# Research Review: A scoping review exploring the needs, barriers, and facilitators to the collection of biological data in adolescence for mental health research

**DOI:** 10.1111/jcpp.70215

**Published:** 2026-08-07

**Authors:** Courtney Worrell, Camila Ribeiro Perez, Sorcha Alford, Rebecca Pollard, Luca Sforzini, Cole Sommeling, James Hotham, Amanda Rodney, Zara Sadiq, Hana Shah, Tyler Weetman, Gillian Brooks, Matthew R. Broome, Niyah Campbell, Nzinga Gardner, Seeromanie Harding, Anna Lavis, Rosemary R.C. McEachan, Valeria Mondelli, Craig Morgan, Kierra Morris, Chiara Nosarti, Talya Porat, David Ryan, Katy Shire, Anthony Woods, Carmine M. Pariante, CELEBRATE Youth Expert Working Group, Paola Dazzan, Rachel Upthegrove

**Affiliations:** 1 Department of Psychological Medicine, Institute of Psychiatry, Psychology, and Neuroscience, King’s College London, London, UK; 2 Institute for Mental Health, University of Birmingham, Birmingham, UK; 3 Birmingham Women’s and Children’s NHS Foundation Trust, Birmingham, UK; 4 Department of Applied Health Sciences, College of Medicine and Health, University of Birmingham, Birmingham, UK; 5 King’s Business School, King’s College London, London, UK; 6 Public Review Member, NIHR, London, UK; 7 Department of Population Health Sciences, Faculty of Life Sciences and Medicine, King’s College London, London, UK; 8 Bradford Institute for Health Research, Bradford, UK; 9 National Institute for Health Research (NIHR) Maudsley Biomedical Research Centre at South London and Maudsley NHS Foundation Trust and King’s College London, London, UK; 10 Health Service and Population Research Department, Institute of Psychiatry, Psychology, and Neuroscience, King’s College London, London, UK; 11 ESRC Centre for Society and Mental Health, Institute of Psychiatry, Psychology, and Neuroscience, King’s College London, London, UK; 12 Department of Child and Adolescent Psychiatry, Institute of Psychiatry, Psychology and Neuroscience, King’s College London, London, UK; 13 Centre for the Developing Brain, Department of Perinatal Imaging and Health, School of Biomedical Engineering and Imaging Sciences, King’s College London, London, UK; 14 Dyson School of Design Engineering, Imperial College London, London, UK; 15 Department of Psychiatry, University of Oxford, Oxford, UK

**Keywords:** Youth, mental health, biological data, research inclusion, barriers and facilitators

## Abstract

**Background:**

There is increasing recognition of the challenge of representativeness in data, including economic, social, and ethnic diversity, in biological mental health research. This is a particular challenge in adolescence, where brain and body development are impacted by the environment. The first step in addressing this is understanding the scale of the challenge. As such, this scoping review aims to explore existing literature to identify and understand the needs, barriers, and facilitators in collecting biological data in adolescent mental health research.

**Methods:**

A systematic search identified papers recruiting participants aged 11–18, collecting biological data, and focussing on mental health/related psychopathology. Screening was performed in duplicate, and data charting was iterative.

**Results:**

The initial search identified 13,752 papers. After removal of duplicates and screening exclusions, 962 papers were included; 892 were explored for recruitment, retention, and engagement. Sample sizes <99 were most common (54.4%), and health settings (45%) were the most frequent recruitment source. As labels for ‘barriers’, ‘facilitators’, and ‘needs’ were rarely used, information on recruitment, retention, and engagement (e.g. strategies for stakeholder engagement) were explored as a proxy. Only 8.6% of papers reported engagement strategies; fewer evaluated their efficacy. Less than half (45.6%) reported retention data, with reasons for lost data mostly relating to the nature of the data collection.

**Conclusions:**

Papers do not adequately report the methods used to ensure sufficient collection of representative data. Limited reporting challenges whether or not facilitators are being implemented. Advancing the field requires detailed guidelines. We present recommendations that serve as a first step towards this development.

## Introduction

Given the importance of adolescence as a critical period in the development of mental health and ill health, there is an urgent need to increase and improve research recruiting samples that are representative of those most at risk, reflecting the economic, social, and ethnic diversity in society. The sparsity of data, particularly biological data, available for research in this population creates barriers to advancing scientific understanding. To facilitate such necessary advancements in our knowledge, a comprehensive understanding of the barriers to the acquisition of these data and potential best practices is required.

Adolescence, the period of life between the ages of 10 and 19 years, is a transitional period marked by distinct vulnerability to developing poor mental health. Poor mental health in adolescents and young adults has been identified as one of the leading causes of disability and death (World Health Organisation (WHO), 2025a). Of lifetime mental health disorders, 50% have been suggested to have onset by 14 years of age, and 75% by 24 years (Kessler et al., 2005), serving to highlight the far-reaching consequences. Globally, it is reported that approximately 1 in 7 adolescents experience a mental health disorder (WHO, 2025b). Concerningly, the situation may be escalating with a significant rise in rates of mental health conditions in young people across numerous countries (McGorry et al., 2024; Patel & Sumathipala, 2001).

To better understand the risk factors and consequences of adolescent mental health difficulties, research needs to look towards prevention and improving treatment, reducing the impact on an individual and societal scale. For this very reason, adolescent mental health is a prominent research focus, with many studies exploring possible risk factors, including environmental, psychosocial, and biological factors, often in silos. Although it is well established that a combination of factors increases the risk for mental health problems (National Institute of Mental Health, 2024), more evidence is required. Systematic reviews have been able to demonstrate associations between biopsychosocial factors in the development of depression in adolescence, for example (Zajkowska et al., 2021), emphasising a clear need to understand how biological mechanisms interact with environmental and psychological risk factors to impact mental health in the adolescent period, looking towards intervention and stratification strategies.

Biological evidence in mental health, thus far has largely been collected in adult samples. Moreover, reports have long suggested that much psychiatric research comes from Western European and North American samples (Patel & Sumathipala, 2001). In adolescent populations, sample sizes tend to be small, or the biological material collected is limited. Key to this present review, existing research may be limited in its applicability as it tends to lack representativeness, specifically in terms of surrounding ethnicity and socioeconomic status. Children and adolescents with socioeconomic disadvantage have been reported to be two to three times more likely to develop mental health difficulties during this period of life (Reiss, 2013). Furthermore, treatment outcomes may differ according to ethnicity, with participants from Asian and mixed-race backgrounds showing less measurable change after treatment for their mental health in comparison to white British participants (Ruphrect-Smith, Davies, Jacob, & Edbrooke-Childs, 2024), making underrepresentation in biological mental health research a pressing issue.

Moreover, significant challenges in representative recruitment and retention lead to further issues such as completion delays, sampling bias, and inadequate statistical power (Parrish, Duron, & Oxhandler, 2017). An emphasis is placed on effective recruitment and retention strategies in the literature, specifically for young samples (Schoeppe, Oliver, Badland, Burke, & Duncan, 2014). Attrition issues may be more likely related to individual factors such as age, ethnic minority status, and socioeconomic status (Karlson & Rapoff, 2009), making some individuals (for example, those from an ethnic minority group) more likely to drop out of research, illustrating the complexity of underrepresentation. While specific to personal barriers such as gender, age, and ethnicity, rather than adolescent participants or biological data collection, a review of representative research samples in mental health identified barriers including distrust in research and language barriers for people from minority ethnic groups (Woodall, Morgan, Sloan, & Howard, 2010).

Therefore, methodological innovation is required to overcome the current limitations in this field and move towards high-quality, representative, and unbiased research. To drive such advancement, a comprehensive understanding of the barriers and facilitators in current research is necessary. In a qualitative piece of research, Jong, Stevenson, Winpenny, Corder, and van Sluijs (2023) reported some barriers to representative data highlighted in focus groups, including stigma, participant age, the perception of the commitment required, and the timing of research. For biological data in younger samples, Rodriguez et al. (2016) theorised unique concerns, such as the influence of potential psychological discomfort, such as feeling embarrassed by the idea of being weighed or giving saliva samples. Facilitators of this type of data collection are less widely reported.

While these insights are important, there is currently little comprehensive knowledge of the barriers and facilitators to collecting biological data in adolescent mental health research. This scoping review aims to address this gap by examining the engagement and retention strategies reported in studies that collected biological data from adolescent participants in mental health research and to identify barriers and facilitators affecting research in this field.

## Methods

The nature of a scoping review allows for the exploration of a broader overview of the literature in this field (Smith & Duncan, 2022), rather than the exploration of findings from a smaller, specific set of publications. This scoping review has been performed using the guidance of Levac, Colquhoun, and O’Brien (2010) and recommendations from the Joanna Briggs Institute Scoping Review Methodology (Joanna Briggs Institute, 2024; Peters et al., 2015). Our protocol was registered in Open Science Framework (https://doi.org/10.17605/OSF.IO/3M9DY) and published (Worrell et al., 2024).

### Review question

What are the needs of the adolescent population and the research itself, and what are the barriers and facilitators in collecting biological data in mental health research from adolescent populations?

We sought to collate details on recruitment and retention methodology from relevant publications describing the collection of biological data in adolescents for mental health research.

### Inclusion criteria

Broad eligibility criteria were implemented to identify relevant papers on this type of work, rather than just publications that explicitly focused on the barriers and facilitators. The full inclusion and exclusion criteria are provided in Table 1. We included studies on recruited adolescent participants between 11 and 18 years. Of note, studies that recruited adolescent participants between 11 and 18 years have been considered regardless of whether they also included younger/older participants. Samples that only recruited children up to the age of 11, or only adults aged 18 and above, were not included. We included all study designs, including reviews and opinion papers, if they specifically discussed the subject matter. Studies conducted globally were included in the review, with no selection based on cultural, ethnic, or sex/gender-based factors; however, only papers published in the English language were included for feasibility reasons.

### Search strategy

A search strategy was developed using the Patient, Exposure, Outcome (PEO) Framework, described in Table 1. The string of search terms can be seen in Appendix S1. The search was conducted in February 2023 and was updated in October 2024 and again in October 2025. Evidence was extracted into the software Zotero, and duplicates were removed.

### Evidence selection

The software ‘Rayyan’ was used to support the process of evidence selection. Screenings of Titles and Abstracts were performed dually and were blinded, with conflicts resolved by an independent reviewer.

Evidence sources included based on initial Title and Abstract screenings were then screened for eligibility based on full text. The full-text screening was again performed in duplicate, with an independent reviewer resolving conflicts. Grey literature identified from reference lists was also screened in duplicate. A team approach was taken to screening, with meetings facilitating the work.

In line with guidance on scoping reviews of published literature, rather than a systematic review, assessments of risk of bias or quality were not performed (Smith & Duncan, 2022).

### Information charting

A Data Extraction form was developed to ensure relevant methodological details, and any information indicative of barriers and facilitators was extracted. The reviewers performed a pilot test of information charting with 10 papers to test the Data Extraction form and the consistency of charting. Charting was iterative, with data collection informed as papers were reviewed. One iterative change was made to the Data Extraction form near the beginning of charting; all reviewers agreed to the change (inclusion of information relating to withdrawals or exclusions following consent) before its implementation. Given the high number of papers included, one reviewer charted data per evidence source. An independent reviewer assessed consistency for all extractions.

### Collating and summarising the data

A descriptive numerical summary of included studies and a narrative synthesis of themes relating to barriers and facilitators is presented. Following charting, reviewers assessed categories, grouping information into wider groups. This included, for example, further categorising papers which reported salivary cortisol, blood or urine samples into ‘fluid-based biomarkers’ to support analysis. Further categories were developed to bring together information on more qualitative factors, such as engagement strategies, which were broken down into (1) engagement with individual young people, (2) engagement with stakeholders, (3) engagement with communities, and (4) feedback strategies.

### Patient and public involvement and engagement (PPIE) consultation

This scoping review was conducted as part of the ‘CELEBRATE’ project (‘Co-producing a framework of guiding principles for Engaging representative and diverse cohorts of young peopLE in Biological ReseArch in menTal hEalth’). Taking a participatory approach, young people (aged 11–18 years) are at the centre of the project and members of our Youth Expert Working Group (YEWG) co-lead the project. We have adopted the additional consultation stage of frameworks as reported by Arksey and O’Malley (2005) and Levac et al. (2010), including members of the YEWG in the review. The review aims, development of the protocol, and preliminary results were discussed with the YEWG members who provided feedback and suggestions, as well as interpretation of the findings and points for discussion.

## Results

**Figure 1:**
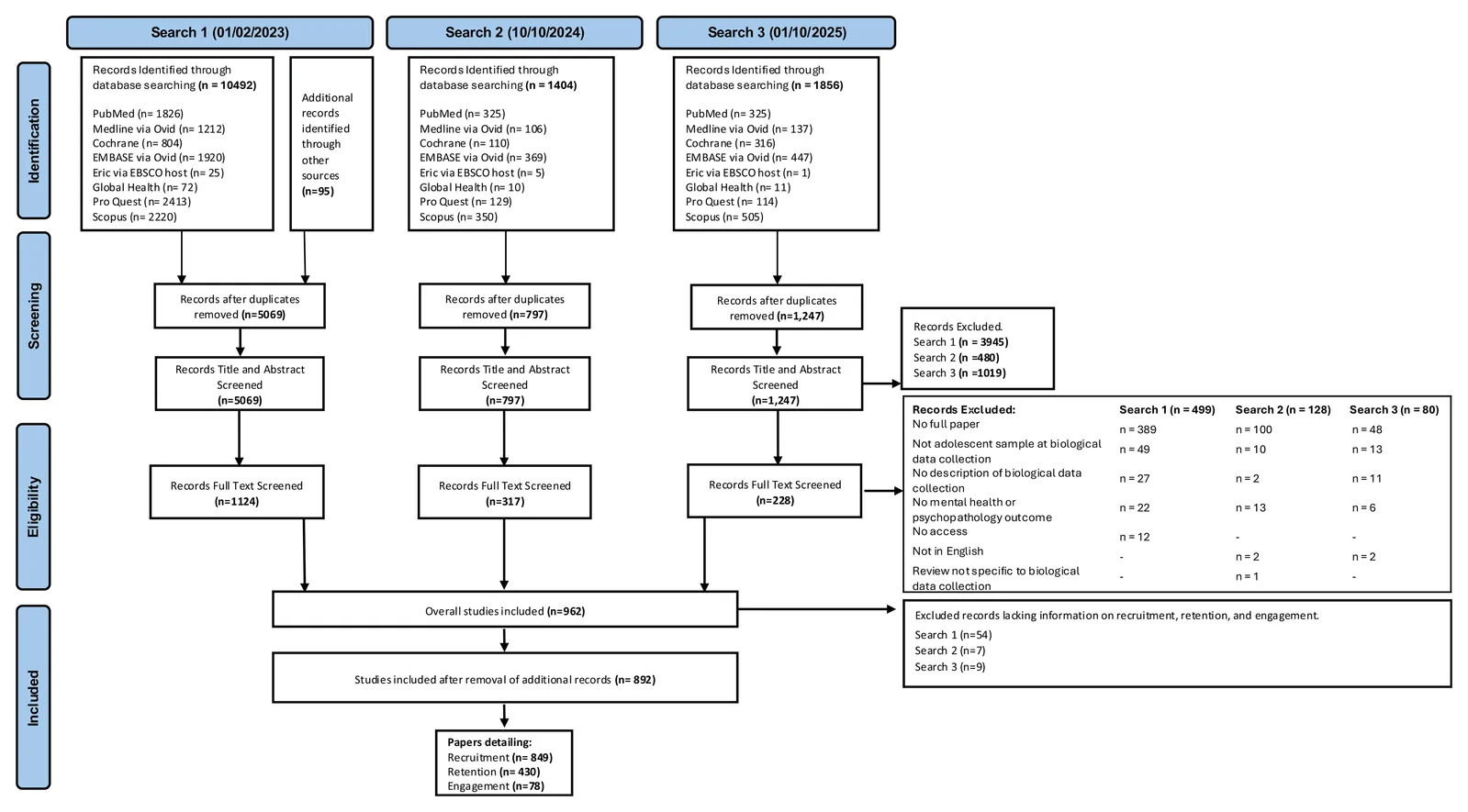
PRISMA flowchart

Following the removal of duplicates, 7,113 papers were screened based on the Titles and Abstracts. Following exclusions, 1,669 papers were screened by full text, and 707 were excluded, leading to the final inclusion of 962 papers, with 892 providing usable data. These are shown in Figure 1, adapted from the PRISMA flowchart by Moher, Liberati, Tetzlaff, and Altman (2010). A full list of the included papers not mentioned in the manuscript can be found in Appendix S2.

### Study characteristics

#### Date of publication

**Figure 2:**
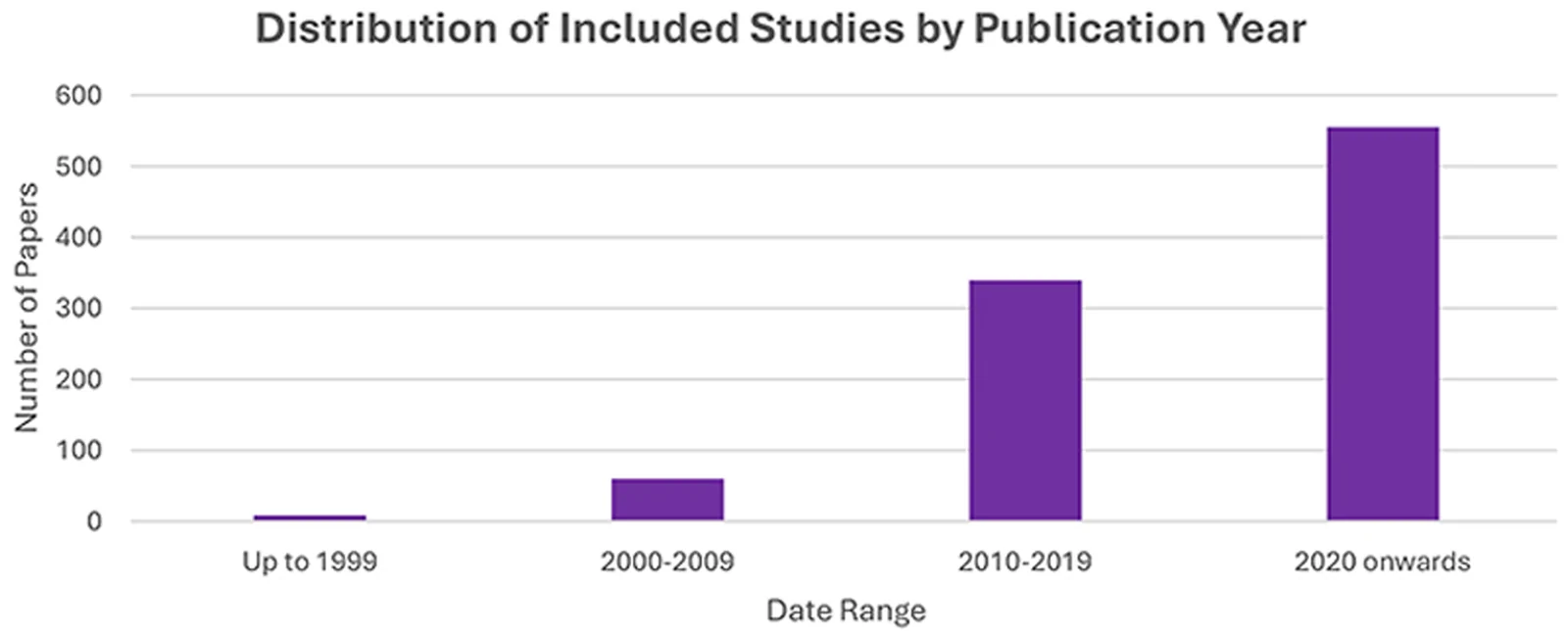
Distribution of included studies by publication yearFigure shows the distribution of total included studies by publication year (*n* = 962).

The 962 papers included (before records lacking relevant information were removed) were published between 1992 and 2025, with half (555, 57.69%) published since 2020, suggesting a growing body of research in this field given the smaller period (Figure 2).

#### Country of origin

Twenty-eight (2.91%) were conducted in Low and Lower-Middle-Income Countries (LMIC), and 219 (22.77%) in Upper-Middle Income Countries (UMIC) (Table 2).

#### Age of participants

Papers recruiting participants 11–18 years old were our focus, but as mentioned, many papers had wider age ranges. Two hundred and eighty-four (29.51%) papers included in the review had a sample which included participants aged 11–18. Three hundred and forty (35.34%) included participants of any age up to 18 years. Two hundred and fifty-one (26.1%) included participants aged 11 and over, and 85 (8.84%) papers included participants who were under 11 years through to over 18 years old.

#### Demographic reporting

**Figure 3:**
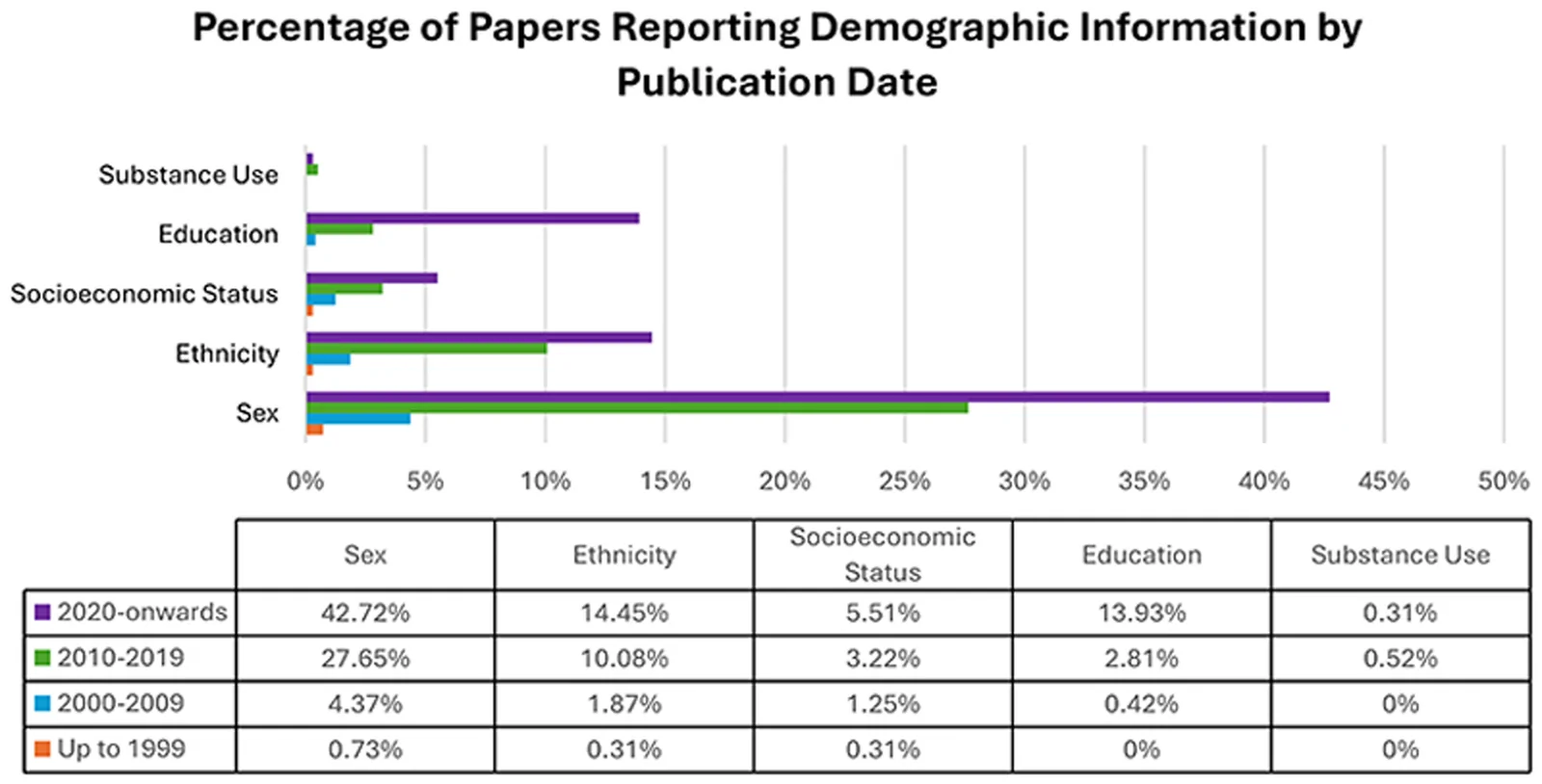
Percentage of papers reporting demographic information by publication dateFigure shows the percentage of papers reporting each demographic from the total included papers (*n* = 962).

All papers included reported age as a demographic factor, and further demographic details are reported in Figure 3. It should be noted that some papers described only research protocols or methodology and therefore did not present any demographics beyond those of the target population and relevant eligibility criteria.

#### Biological data collection methods

The broader categories of biological data collection methods are reported in Table 2. Several (*n* = 173) of the included studies reported multiple methods. When looking at the specific methods used, including where multiple types of data were collected, blood samples (*n* = 222), fMRI (*n* = 275), and MRI (*n* = 210) were most commonly reported. A multitude of other types of biological data were collected, including but not limited to eye movement/tracking (*n* = 9), saliva samples (*n* = 74), heart rate (*n* = 14), EEG (*n* = 115), and anthropometric measures (*n* = 60). The full scope of measures and the frequency of reporting are reported in Table 3.

Seventy papers did not report any recruitment, retention, or engagement methods and were removed from further analysis. Therefore, 892 papers were included in the rest of the analysis.

A summary of the broad findings relevant to recruitment, engagement, and retention approaches of the 892 papers included can be found in Table 4.

### Recruitment

A range of recruitment methods was reported, with the most common source of recruitment being through clinics (such as healthcare settings including primary care clinics) (*n* = 310, 34.75%). One hundred and twenty-seven papers (14.25%) described recruitment through populations or schools. Of those reporting multiple recruitment methods (*n* = 145), 99 included the use of posts and/or advertisements as one of the methods, typically alongside recruiting through clinics (*n* = 87) or population/schools (*n* = 27).

### Engagement strategies

In total, 77 papers (8.62%) reported engagement strategies (with young people, stakeholders, the community, or collecting/providing feedback). Forty-five studies reported one type of engagement, 20 reported two, and 12 studies reported three. None reported strategies which fell across all four types of engagement (young people, stakeholders, community, and feedback). A summary of the engagement strategies reported in the included papers can be seen in Table 5. A more detailed breakdown of the 77 papers describing engagement strategies has been categorised and presented in Table S1. It is important to note that some of these papers were reports on the same study, for example, a protocol paper and a data paper and therefore reflect some of the same strategies and have been reported together. For the purpose of synthesis, we have not distinguished between engagement, which would be considered PPIE (for example, a youth advisory panel), as opposed to engagement with study participants (for example, feedback from participants).

### Engagement with young people

Forty-three studies (4.82%) reported activities considered to be engagement with young people. These included having youth-friendly recruitment procedures (having adapted methods to be suitable for young people, for example, use or development of age-appropriate materials, adapting measures and procedures) (*n* = 18), implementing youth ideas (such as youth advisory panel input in the design of recruitment materials) (*n* = 10) and mock scanner/training for familiarisation of scanning environment deemed to be beyond standard procedure (*n* = 7), specifically in neuroimaging studies. Some of the studies (*n* = 32) implementing these strategies did not evaluate the impact of these strategies. This may be partly due to the number of papers which describe protocols or methodology only (of those reporting engagement, *n* = 51). Of papers evaluating engagement methods, there was a varying degree of detail. For example, Kaufman et al. (1997) described youth-friendly procedures such as adapting the laboratory environment to include age-appropriate entertainment (such as books, art supplies, and video games) and reported that such factors were rated positively and that ‘there were no problems with missing data’ but do not evaluate the strategies further (Kaufman et al., 1997). Leung, Chien, and Weiss (2024) attributed the success of their recruitment and retention specifically to strategies such as brief questionnaires with flexible timelines and giving participants checklists (Leung et al., 2024). One neuroimaging study, conducted by Siless et al. (2020) combined mock scanning/training for familiarisation of the scanning environment beyond standard procedure and offered youth-friendly procedures to ‘maximise participant comfort’, such as choosing a specific coil, shortening tasks and creating multiple opportunities for rest breaks, acknowledging that participants were adolescents, many with anxiety or attention-deficit hyperactivity disorder (ADHD) (Siless et al., 2020). The authors describe that 80% of young people reported feeling good/very good after scans and reported low attrition rates.

### Engagement with stakeholders

Thirty-three papers reported stakeholder engagement strategies, relating to schools and teachers (*n* = 6), or parents/guardians (*n* = 8). Bonhauser et al. (2005) described school or teacher collaboration and facilitation in the development of the study in considerable detail, from the design of the intervention, approval by school directors and teacher committee councils, parent committee councils and student representatives. The authors reported a direct link between the involvement of these stakeholders and high participation and compliance, describing ‘an effective coalition among school authorities, teachers, students and researchers’ (Bonhauser et al., 2005). It is important to note, however, that this was directly linked with the programme explored as opposed to the biological data collection being conducted. Another paper reported that their research team engaged with leaders of school system research departments; however, despite this engagement, the research did not receive approval to recruit through the school system (Liu et al., 2013), leading to the reduction of the anticipated recruitment goal. As a result, the researchers of this study adapted the recruitment approach and made procedures more youth-friendly. Most papers described the involvement of stakeholders in protocol or methodology papers, and therefore, there was little evaluation of the impact overall. One paper, however, described that participants who had completed biological data collection were representative of the overall sample, which the authors attributed, in part, to the input of stakeholders (Dickie et al., 2024).

### Community engagement

Of the 19 papers describing community engagement, 2 reported researcher and community relationships, 3 disseminated findings to the community, 11 community involvement in the research, and 3 piloted the study with the community. The types of community engagement described varied considerably. One protocol paper for the IMAGINE Network’s Mind and Gut Interactions Cohort (MAGIC), described PPIE efforts being made in detail, specifying that communities of patients were involved in identifying funding opportunities and research topics, and in study design and recruitment (Moayyedi et al., 2020). The authors additionally described leveraging a Patient and Community Engagement Research (PaCER) programme to promote roles for patients with inflammatory bowel disease and irritable bowel syndrome and family members in health through engagement in research. The authors describe this as a key strength of the work, ‘making it more patient focussed, and supports the knowledge translation of the findings to patients.’ Similarly, suggesting possible success of community engagement strategies, Tobe et al. (2022) described an approach to ‘provide a hands-on educational neuroscience experience for families.’ Through community partnerships and events, the research team provided mock scan exposure, lectures, faculty tours, and art, to name a few, and reported that ‘promotion of community and scientific partnership became a core tenet of recruitment and retention in this longitudinal cohort’ (Tobe et al., 2022).

### Feedback strategies

Twenty-two papers identified in the review reported the use of feedback strategies, such as collecting feedback from participants or caregivers. The REACT study focused on measuring development to improve the ‘quality, relevance and impact’ of the study. Part of the feedback acquired led to changes in participant documents, questionnaires, and risk protocols (Smith et al., 2021). However, as this was a protocol paper, there was no further evaluation or discussion on this input. In contrast, a paper on the acceptability and feasibility of a protocol reported interesting findings surrounding biological data collection methods. The authors reported that 78.6% of participants would participate in research which involved a blood test, and 85.7% would agree to an MRI scan (Youn et al., 2023). However, this feedback should be interpreted with caution, given the mock nature of the data collection, and feedback was collected as part of the research rather than as feedback on real data collection.

In some cases, feedback was combined with other stakeholder engagement. For example, in a protocol paper published by Hughes et al. (2025), the authors describe extensive engagement and piloting, including focus groups with parents and surveys on important matters such as access to data, attitudes, and decision-making. In addition to this feedback stream, several other stakeholders were included in the consultation, such as professionals in pregnancy and newborns, biological data methods (genetics and omics, for example), epidemiologists, and more, as well as input from ‘groups representing Aboriginal and Torres Strait Islander families, those from culturally and linguistically diverse backgrounds, LGBTQIA + communities, and numerous peak bodies’. The included paper describes a protocol and references that a cohort profile will be published but makes reference to other evaluations from the strategies used, including leveraging funding and collaborators.

### Retention

Four hundred and seven (45.6%) papers reported data on retention. Of these, 233 papers (26.1%) were published from 2020 onwards, and 344 (84.11%) described reasons for dropouts, exclusions, or losses to follow-up. A summary of reasons for dropouts and exclusions relating to mental health and/or biological data collection, categorised according to data collection method, study design, and indication, can be seen in Table 6.

Thirty-four papers (Table S2) explicitly reported using retention methods. Methods utilised included strategies such as short or regular follow-up periods (Abrial et al., 2022; Cavalcante et al., 2024; Lee et al., 2013), weekly/frequent contact with participants/families and reminders (Braun et al., 2020; Cheung et al., 2023; Du et al., 2025; Gindt et al., 2019; Lee et al., 2023; Leung et al., 2024, Lv et al., 2024; Montgomery et al., 2024; Nicol et al., 2018; Pedrini et al., 2021; Piccin et al., 2024; Spark et al., 2025), providing added flexibility to attend visits/intervention through session opportunities or location (Cotton et al., 2019; Leung et al., 2024; Rubia et al., 2024; Trivedi et al., 2020; Wall et al., 2016), mailouts, birthday cards, newsletters, or social media pages (Braun et al., 2020; Schmidt et al., 2013; Tobe et al., 2022; Trickett, Noll, & Putnam, 2011), having visits on nonschool days (Cheung et al., 2023) or at school or home (Pedrini et al., 2021; Piccin et al., 2024; Warner, Behnke, Eyler, & Szabo, 2011), and providing 24 hr hotlines (Kidman et al., 2024). Not all papers describing retention methods reported data on actual retention or attrition figures, and most described the protocol or methodology. One paper described a contingency plan in case of retention issues (Roberts et al., 2021). It is important to note that retention methods have been considered separately from factors relating to recruitment, such as compensation for participants.

### Information in other sources

As expected with large cohorts, we identified several papers that describe the same cohort or use data from overlapping samples. For one such cohort, the Longitudinal Youth-At-Risk Study (LYRIKS) (Mitter, Nah, Bong, Lee, & Chong, 2014), a publication was identified during the grey literature search, describing the community-engaged framework for outreach strategies used for the cohort. This paper was not included nor eligible in our original searches due to the lack of description regarding biological data collection, which was added later to the cohort. Mitter et al. (2014) described a series of outreach approaches, which includes community engagement through educational institutes and health partners through workshops, as one of many examples which were implemented to achieve three objectives, (1) empower the community with knowledge on the ultrahigh risk (UHR) concept, (2) train professionals with appropriate skills to identify UHR, (3) recruit participants into the research. Workshops aimed at training people to identify young people at ultrahigh risk (UHR) of psychosis, which would subsequently feed into recruitment, and figures reported suggest efforts were successful, with over 3,700 referrals to the cohort through this outreach. Further outreach included strategies such as forums, which were also evaluated, showing that 77.4% of attendees (*n* = 155) reported having a better understanding of clinical research. In addition, 58.8% indicated they would help or participate in clinical research in the future. While the engagement strategies appear successful, the authors also reported challenges collaborating with community partners due to individual priorities and reported that working towards a mutual benefit was helpful.

## Discussion

We conducted a large and comprehensive scoping review of 962 studies that gathered biological data collection to explore adolescent mental health or relevant psychopathology and highlighted the representativeness and challenges in this specific field of research. The results indicate that while many studies in this field are being conducted, significant gaps remain, such as a need for larger cohorts and more representative samples. Moreover, reporting of factors such as recruitment, retention, and engagement with stakeholders provided insight into barriers and facilitators; however, details on how the work was conducted were limited. This restricted our understanding of how these factors could influence future research and indicated a possible need for more specific reporting guidelines for research in this field. Barriers reported include limited retention strategies, which could improve the size and completeness of samples; however, it is also unclear as to whether such strategies were used but not reported. Limited research conducted in LMICs is also a barrier to improving representativeness and generalisability in this field. Facilitators may include employing multiple recruitment and retention methods, specifically around keeping contact, adapting to young people’s schedules, and engaging with stakeholders, including young people themselves, in the design and conduct of the research. Notably, the majority of included studies acquired neuroimaging data, which highlights the need for a wider range of biological measures, such as fluid-based biomarkers and EEG, in future research, in addition to neuroimaging.

The limited reporting of retention methods, engagement and feedback strategies, featuring only in 3.5% and 8.12%, respectively, of all included studies, is also a main finding of this review. This raises a question as to whether more studies are implementing new strategies and not reporting them, or whether the lack of reporting is consistent with, and representative of, limited implementation. As noted in our results, one additional paper not eligible for inclusion in the review, as it did not specifically mention the biological data collection methods used in the cohort, presented some relevant information for our research question surrounding engagement strategies used and evaluated (Mitter et al., 2014). Other included work may have reported methodological details in different publications without describing features relevant to our inclusion criteria (for example, describing biological data collection methods). In recent work, on recruitment and retention in longitudinal research with adolescent populations, Jong et al. (2023) included that recruitment strategies, both effective and ineffective, should be reported alongside study results.

Other reviews have similarly identified limited reporting and retention rates in adolescent samples (albeit not specific to biological research) (Tobe et al., 2022), as well as high attrition rates (Karlson & Rapoff, 2009). While we show an increase in reporting of recruitment and retention according to the date of publication, there is a clear need for recruitment and retention strategies and methods to be reported as standard, so that lessons can be shared to influence the success and applicability of the research in adolescent populations (Parrish et al., 2017). We found that where stakeholder engagement and PPIE were reported, there was little evaluation, possibly because these were mostly reported in papers describing protocols and methodology. PPIE specifically has become an increasingly important part of health research, but reviews highlight that it is inconsistently reported (Brett et al., 2012; Mockford, Staniszewska, Griffiths, & Herron-Marx, 2012; Staniszewska, Brett, Mockford, & Barber, 2011). Such inconsistency poses a difficulty for both understanding the context for those at the centre of the individual works and implementing best practices and future developments (Mockford et al., 2012). The discrepancy between increasing encouragement from funding bodies to include PPIE and a lack of reporting highlights a need for more transparency, and journals are indeed moving to promote this. The Lancet Psychiatry, for example, has recently announced that while papers not describing PPIE are still publishable, authors must report any omission as a limitation of the work, and clear guidance on what information is provided, specifically pertaining to distinct stages of design, implementation, and dissemination, is available (Davis et al., 2024).

Our review also highlighted important insights into the current state of the research field, including the lack of representation of the country of origin. We showed that 702 of the 962 papers included originated in HIC, and only 28 were conducted in LMIC. Studies exploring research in LMICs show that although poor mental health affects adolescents globally, research in these locations is scant (Razzouk et al., 2010), specifically around the use of child and adolescent mental health services (Babatunde, van Rensburg, Bhana, & Petersen, 2021) and interventions (Klasen & Crombag, 2013). A trend suggesting that research in LMIC is increasing with time was identified, in line with reports that scientific research (albeit not specific to this field) in LMIC has increased in the last decade (Bezuidenhout & Chakauya, 2018). Biological research, specifically, may be impacted by a lack of resources and the laboratory infrastructures available (de Vries et al., 2011). While collecting samples to export to HICs may be a resource-effective solution, there are considerable practical and ethical challenges in doing so (Matandika et al., 2020). Systemic changes are needed to advance and increase biological research into adolescent mental health in LMIC.

It is also important to highlight results providing insight into the representativeness of recruited samples, beyond the country of origin. We gathered information on the frequency of reporting different sociodemographic variables, such as ethnicity and socioeconomic status. With the exception of reporting age and sex of participants, demographics such as ethnicity and socioeconomic status were reported in less than 30% of papers included, highlighting the need for increased reporting and the implications for the generalisability of findings and reducing disparity. Similar rates of reporting sociodemographic information have been found in other reviews, such as in reviews focussing on representation in eating disorder research (Burnette, Luzier, Weisenmuller, & Boutté, 2022; Egbert, Hunt, Williams, Burke, & Mathis, 2022), although these noted that there is an increasing frequency of reporting which was corroborated in the results of the present work. While examining the demographics reported was beyond the scope of this review, results around recruitment methods used also gave interesting insights on this theme. Our results demonstrated that recruiting participants through clinics was the most common method. Clinical settings have consistently been reported as a common recruitment source in adolescent mental health research (Kilicel, de Crescenzo, Pontrelli, & Armando, 2023) and in randomised controlled trials in mental health (Liu et al., 2018). While several studies use this recruitment method, evidence suggests that certain groups are less likely to access mental health services, posing a potential issue for the representativeness of samples (Oliver, Pearson, Coe, & Gunnell, 2005). Given the population of interest, it is not surprising that around 14% of papers included recruited participants just through schools; a popular recruitment strategy with dedicated literature specifically sharing strategies for school-based research with children (Trapp, Giles-Corti, Martin, Timperio, & Villanueva, 2012), although not specific to biological data collection. A school-based pilot study collecting salivary cortisol samples found that while the protocol had been co-produced with both parents and teachers, only 11.3% of parents consented for their child to participate (Warne et al., 2022), suggesting that the nature of the research, and specifically the biological nature of data collection, may play a role in the success of recruitment via schools.

As discussed, limited reporting of engagement and PPIE work was one of the main results of this review. For engagement with young people specifically, much of the focus was on making biological data collection methods, mainly neuroimaging, youth-friendly. Likely due to the complexity of acquiring high-quality neuroimaging data, the literature has previously focused on key considerations for conducting neuroimaging studies with adolescents, with mock scanning and adapted procedures to facilitate data collection. Information on methods that facilitate collection of other biological data in this population is less readily available (Davis, Garza, & Church, 2022). Beyond adapting research protocols, engaging with and involving young people in research is clearly important but not often published (Viksveen et al., 2022). We are seeing an increase in reports on co-production, such as a recent paper by Sonuga-Barke et al. (2024) reporting a co-production approach and youth involvement processes as a possible model for future research focussing on neurodivergence or mental health in adolescents. Co-production with young people, or any stakeholders, is increasingly important and has the potential to facilitate research. Still, the limited evaluation we found reported varied experiences. Positive experiences were reported by some (Bonhauser et al., 2005), while others reported involvement of schools but with limited success (Liu et al., 2013). Similarly, other literature has shown that when using a parental opt-in approach to consent, there were high levels of nonresponse from parents of invited children despite having co-produced the study, and the protocol having been deemed acceptable by parents as stakeholders (Warne et al., 2022).

### Strengths and limitations

The nature of biological data collection in adolescent mental health research is complex. By gathering available information on the state of the research in this field, as done in this review, we can begin to unpick the needs, the problems and the beneficial strategies that should be implemented to improve work in this field, opening the door to more and increasingly representative and applicable research in the area. Given the broad inclusion criteria and use of seven bibliographic databases, the present scoping review is an extremely comprehensive view of the current state of the literature. In addition, the identification of grey literature increases the breadth and further reduces the impact of publication bias. Following the most up-to-date guidance on scoping review methodology and employing an iterative process for data charting has further increased the robustness of this work, ensuring that relevant information has been captured from as many sources as possible.

It is, however, important to consider the results of this scoping review in the context of its limitations. A full quality and risk of bias assessment for all evidence sources included in our review was not performed as it was beyond the scope of the methodological frameworks we have followed, and is not, as standard, included in the definition of a scoping review. It is important to note that the results of the studies included were not the focus of the review. Rather, we aimed to broadly explore the literature to seek details of the barriers and facilitators in conducting such research, and therefore, such assessments should not have a substantial impact on our ability to achieve this aim. An important limitation to acknowledge is that the breadth of the biological data collection methods included may have been restricted by the search terms we used. While we were able to identify a broad range of methods used across the included works, there was a high proportion of neuroimaging studies, which were directly captured by the search terms. Fewer evidence sources were included using saliva samples, which is likely a popular biological data collection method when conducting research with this population. The review was also restricted to publications written in the English language for feasibility reasons, potentially omitting some relevant data sources. While dual extraction is considered best practice, this was not feasible given the scale of this review. Notably, the literature describes that this is sufficient with an independent reviewer checking accuracy, as was done for this review (Pollock et al., 2023).

## Conclusion

Biological research currently being conducted in adolescents is often based on nonrepresentative data, recruited from participants in high-income countries. Barriers and facilitators for recruitment, which could aid the assessment and development of more representative cohorts, are consistently underreported. However, given the lack of detailed reporting, it is difficult to currently make evidence-based recommendations for future research. The lack of reporting is particularly relevant as it raises questions about the need for standardisation of reporting in specific populations, such as adolescents. The results of this scoping review highlight the need for reporting guidelines and offer the first areas of focus, including the reporting of demographic features and recruitment strategies. Advancing this specific field of research requires even more transparency of methods, including where co-production and engagement are used, which are not consistently being reported at present. A collaborative approach with different groups of stakeholders is needed to identify a shared vision for advancing research in this field and overcoming the barriers identified.

## Supplementary Material

Supplementary Material

## Figures and Tables

**Table 1:** Search strategy, including search terms, search strings and full inclusion and exclusion criteria

Broad search terms based on Patient, Exposure, Outcome (PEO) framework:	
Population: Young people, adolescents, teenagers.	
Exposure: Participation in mental health,	
psychopathology-related research, with biological data collection	
Outcome: Participation, barriers to recruitment, retention, involvement	
Databases searched: PubMed Medline Scopus Cochrane ERIC EMBASE ProQuest EBSCO Global Health Manual exploration of relevant citations from reference lists of sources identified in search	
Search string: (“MRI” OR “fMRI” OR “EEG” OR “PET” OR “neuroimaging” OR “biological research” OR “biological sample*” OR “biologic*” OR “blood sample* “OR “urine sample*”) AND (“mental health” OR “mental” OR “depression” OR “anxiety” OR “self-harm” OR “self-harm” OR “suicide” OR “psychiatric” OR “psychopathology” OR “mood disorder” OR “autism” OR “neurodivergence”) AND (“youth*” OR “adolescent” OR “adolescence” OR “teen*” OR “teenager*” OR “high school student*” OR “AYA”) AND (“retention” OR “recruitment” OR “recruit” OR “recruited” OR “enrol” OR “enrol” OR “enrollment” OR “enrolment” OR “enrolling” OR “enrolled” OR “participation”)	
Inclusion criteria: Recruited participants aged between 11 and 18 years Focused on mental health or psychopathology outcomes and experiences Included biological data collection methods, including but not limited to neuroimaging or biological sample collection	Exclusion criteria: Not written in the English language Did not recruit participants aged between 11 and 18 years Did not focus on mental health or psychopathology outcomes and experiences Did not include biological data collection methods

**Table 2:** An overview of the number of papers according to country income status, sample size, mental health or psychopathology, and broad biological data collection methods

Data	Categories	Number of papers
Income status of country/countries	HIC	687
	UMIC	219
	LMIC	28
	HIC and UMIC Multinational	12
	HIC and LMIC Multinational	3
	Not described/Missing	28
Sample size	<99	485
	100–499	347
	500–999	52
	1,000–4,999	44
	5,000+	23
	Not described/Missing	11
Indication/Mental health or psychopathology-related outcome	ADHD	58
	Anxiety	38
	ASD	140
	Bipolar disorder	62
	Depression	188
	Eating disorder	17
	General psychopathology	54
	OCD	5
	Personality disorder	0
	Psychosis	66
	PTSD	19
	Substance use disorder	23
	Other	225
	Multiple	41
	Not categorised	17
Biological Data Collection Methods	EEG	80
	Fluid-based (e.g. blood, saliva, urine)	161
	Neuroimaging	504
	Other (e.g. BMI, hair, teeth)	43
	Multiple methods	173
	Mock data collection	1

ADHD, attention-deficit hyperactivity disorder; ASD, autism spectrum disorder; BMI, body mass index; EEG, electroencephalogram; HIC, high-income country; LMIC, lower-middle-income country; OCD, obsessive compulsive disorder; PTSD, posttraumatic stress disorder; UMIC, upper-middle-income country.

**Table 3:** An overview of specific biological measures and frequency distribution across included works

Biological measure umbrella/broad term	Specific biological measure	Frequency reported
Behavioural and digital monitoring	Automatic physiology	1
	Biosensors	2
	Eye movement/tracking	9
	Smartphone passive sensing	1
Biospecimens	Fluid-based sampling	
	Blood samples (including blood spot and cord blood)	222
	CSF samples	2
	Lumbar puncture	1
	Mouthwash samples	1
	Saliva samples	74
	Stool samples	18
	Urine samples Genetic-based sampling	54
	Buccal swab	8
	Genetic testing nonspecified	1
	Throat culture Tissue-based sampling	1
	Hair samples	30
	Nail samples	2
	Skin biopsy	1
	Teeth	3
Cardiovascular measures	Blood pressure	20
	Cardiovascular functioning or monitoring or ‘measures’	4
	EDA	1
	Heart rate	14
	HRV	7
	Impedance cardiography	1
	Pulse amplitude or rate	2
	Respiratory sinus arrythmia	1
	Skin conductance	5
Clinical laboratory	Pregnancy testing	1
	Pubertal development	1
	Temperature	2
	Thyroid and liver function	1
Electrophysiology	EEG measurements	
	EEG	115
	ERP	4
	SEEG Magnetic-based	1
	MEG Brain stimulation	9
	rTMS	2
	TMS Other electrophysiological	1
	ECG/EKG	13
	EPSI	1
Neuroimaging	MRI nonspecified (including resting state)	210
	Neuroimagine nonspecified	1
	Functional brain imaging fMRI (including resting state)	275
	fNIRS/NIRS	16
	H-MRS	1
	MRS	9
	PET	8
	rsFC	1
	SPECT	2
	Structural brain imaging	
	CT	3
	DSI	1
	DTI	23
	dMRI	1
	FLAIR	1
	sMRI	1
Other	EMT	1
	OCTA	1
	Physiological signals	1
Other imaging	Ultrasound	2
	X-Ray	3
Physical measures/function	Aerobic capacity	2
	Anthropometric measures (including body composition and BMI)	60
	Physical examination	2
Respiratory	Respiratorty rate/data	4
	Respiratory sinus arrythmia	1
Sleep physiology	Actigraphy	11
	Polysomnography	1
	Polygraphy	1
	Sleep data/monitor	2

Table provides an overview of the more specific biological measures reported and frequency across the included studies. It should be noted that total numbers do not correspond to the number of included papers, as several works included multiple biological measures. BMI, body mass index; CSF, cerebrospinal fluid; CT, computed tomography; dMRI, diffusion magnetic resonance imaging; DSI, diffusion spectrum imaging; DTI, diffusion tensor imaging; ECG/EKG, electrocardiogram; EDA, electrodermal activity; EEG, electroencephalogram; EMT, electromagnetic tomography; EPSI, echo-planar spectroscopic imaging; ERP, event-related potentials; FLAIR, fluid attenuated inversion recovery; fMRI, functional magnetic resonance imaging; fNIRS, functional near-infrared spectroscopy; H-MRS, proton magnetic resonance spectroscopy; HRV, heart rate variability; MEG, magnetoencephalography; MRI, magnetic resonance imaging; MRS, magnetic resonance spectroscopy; NIRS, near-infrared spectroscopy; OCTA, optical coherence tomography angiography; PET, positron emission tomography; rsFC, resting state functional connectivity; rTMS, repetitive transcranial magnetic stimulation; SEEG, Stereo encephalogram; sMRI, structural magnetic resonance imaging; SPECT, single-photon emission computed tomography; TMS, transcranial magnetic stimulation.

**Table 4:** Summary of key recruitment, retention and engagement details based on the type of biological data collection performed

Data type	Number of studies	Age range	Young people engagement strategies	Stakeholder engagement strategies	Community engagement strategies	Feedback strategies	Recruitment methods
Neuroimaging	465	Adolescent (*n* = 148)/adolescent-adult (*n* = 109)/child–adolescent (*n* = 163)/child–adult (*n* = 45)	Noncash Incentives Beyond Retention (*n* = 1), Building Rapport (*n* = 3), Regular Contact Beyond Retention (*n* = 1), Youth Friendly Procedures (e.g. adapting methods/measures for young people, such as age-appropriate materials or protocols) (*n* = 5), Close Attention and Monitoring (*n* = 3), Disseminating Findings to Participants (*n* = 1), Mock Scanner/Familiarising Participants with Scanning Environment Beyond Retention (*n* = 4)/Implementing youth ideas (*n* =1)	Stakeholder Collaboration and Facilitation (*n* = 3)/School and Teacher Collaboration and Facilitation (*n* = 1), Providing Information to Parents and Guardians (*n* =1)	Community Involvement in Study (*n* = 2), Dissemination of Findings to Community (*n* = 1), Piloted Tested Study to Community (*n* = 1), Researcher Community Relationship (*n* = 1)	Participant Feedback (*n* = 5), Participant and Caregiver Feedback (*n* =1)	Clinics (*n* = 208), Other studies (*n* = 131), Posts/Advertisements (*n* = 104), Population/Schools (*n* = 88), Not described (*n* = 23)
Multiple	161	Adolescent (*n* = 40)/adolescent-adult (*n* = 49)/child–adolescent *(n = 56)*/child–adult (*n* = 16)	Building Rapport (*n* = 1), Noncash Incentives Beyond Retention (*n* = 6), Regular Contact Beyond Retention (*n* = 4), Youth Friendly Procedures (e.g. adapting methods/measures for young people, such as age-appropriate materials or protocols) (*n* = 7), Disseminating Findings to Participants (*n* = 3), Educational Opportunities (*n* = 2), Implementing Youth Ideas (*n* = 7), Mock Scanner /Familiarising Participants with Scanning Environment Beyond Retention (*n* = 2)/Close attention and monitoring (*n* = 1)	Stakeholder Collaboration and Facilitation (*n* = 14), School and Teacher Collaboration and Facilitation (*n* = 3), Parent and Guardian Collaboration and facilitation (*n* = 2), Providing Information to Parents and Guardians (*n* = 2), Referral to Mental Health Services and Risk Assessment (*n* = 1)	Community Involvement in Study (*n* = 8), Dissemination of Findings to Community (*n* = 1)	Participant Feedback (*n* = 9)/Caregiver Feedback (*n* =1)	Clinics (*n* = 86), Population/Schools (*n* = 37), Posts/Advertisement (*n* = 33), Other Studies (*n* = 24), Not described (*n* =11)
Fluid-based biomarkers	148	Adolescent (n=37)/adolescent-adult (*n* = 49)/child–adolescent *(n = 56)*/child–adult (*n* = 6)	Building Rapport (*n* = 2), Youth Friendly Procedures (e.g. adapting methods/measures for young people, such as age-appropriate materials or protocols) (*n* = 5), Close Attention and Monitoring (*n* = 2), Implementing Youth Ideas (*n* = 2)/Noncash Incentives Beyond Standard Procedure (*n* = 2)/Educational opportunities (*n* = 1)	Stakeholder Collaboration and Facilitation (*n* = 3), School and Teacher Collaboration and Facilitation (*n* = 2), Parent and Guardian Collaboration and Facilitation (*n* = 1), Providing Information to Parent and Guardian (*n* = 1)	Community Involvement in Study (*n* = 1), Piloted Tested Study to Community (*n* = 1), Researcher Community Relationship (*n* =1)	Participant Feedback (*n* =3), Caregiver Feedback (*n* =1), Participant and Caregiver Feedback (*n* =1)	Clinics (*n* = 80), Population/Schools (*n* = 38), Other Studies (*n* = 28), Posts/Advertisements (*n* = 16), Not described (*n* = 4)
EEG	75	Adolescent (*n* = 24)/adolescent-adult (*n* = 16)/child–adolescent (*n* = 28)/child–adult (*n* = 7)	Noncash Incentives Beyond Retention (*n* = 1), Youth Friendly Procedures (e.g. adapting methods/measures for young people, such as age-appropriate materials protocols) (*n* = 1)/Disseminating Findings to Participants (*n* = 1)	Providing Information to Parents and Guardians (*n* =1)	Dissemination of Findings to Community (*n* = 1)	None	Clinics (*n* = 40), Population/schools (*n* = 20), Posts/advertisements (*n* = 13). Other studies (*n* = 9), Not described (*n* = 6)
Other	42	Adolescent (*n* =11)/adolescent-adult (*n* = 8)/child–adolescent (*n* = 19)/child–adult (*n* = 4)	Mock Scanner /Familiarising Participants with Scanning Environment Beyond Retention (*n* = 1)	None	Piloted tested study to community (*n* = 1)	None	Clinics (*n* = 19), Other studies (*n* =9), Population/schools (*n* = 13), Posts/Advertisements (*n* =7), Not described (*n* = 1)
Mock neuroimaging	1	Adolescent-adult (*n* =1)	None	None	None	Participant feedback (*n* = 1)	Clinics (*n* = 1)
Total =	892	892	70	35	19	22	n/a

**Table 5:** Summary of engagement strategies described

Engagement type	Engagement strategy	Number of papers
Young People Engagement	Dissemination of findings to participants	5
	Building rapport with participants	6
	Noncash incentives beyond standard procedures	10
	Regular contact beyond standard procedure	5
	Youth friendly procedures	18
	Implementing youth Ideas	10
	Mock scanner training for familiarisation of scanning environment beyond standard procedure	7
	Educational opportunities	3
	Close attention and monitoring	5
Stakeholder Engagement	School or teacher collaboration and facilitation in the development of the study	6
	Stakeholder collaboration and facilitation in development of the study	16
	Parent/guardian collaboration and facilitation in the development of the study	3
	Providing information to parents/guardians	5
	Referrals and risk assessments provided	1
Community Engagement	Researcher and community relationship	2
	Dissemination of research to community	3
	Community involvement in research	11
	Pilot tested study to community	3
Feedback Strategies	Caregiver feedback	2
	Participant feedback	18
	Participant and caregiver feedback	2

**Table 6:** Reasons for dropouts and exclusions relating to mental health and/or biological data collection, according to the study design and indication

Data type	Number of studies	Study design	Indications	Reasons for loss of data
Neuroimaging	465	Experimental (*n* = 34), Observational (*n* = 358), Pilot Study (*n* = 2), Protocol (*n* = 21), Not categorised (*n* = 50)	ADHD (*n* = 20), Anxiety (*n* = 20), ASD (*n* = 62), Bipolar Disorder (*n* = 46), Depression (*n* = 87), Eating disorder (*n* = 5), General Psychopathology (*n* = 21), OCD (*n* = 5), Personality Disorder (*n* = 9), Psychosis (*n* = 37), PTSD (*n* = 13), Substance use disorder (*n* = 16), Other (*n* = 96), Multiple (*n* = 24), Not categorised (*n* = 46)	Adverse events (*n* = 2), Adverse reactions or discomfort during scan or procedure (e.g. became claustrophobic, anxious, panic attack) (*n* = 22), Brain abnormalities detected during scan (neurologic, structural, neuroanatomical, anatomical) (*n* = 14), Caregiver/participant found study unhelpful or believed scope was unwarranted (*n* = 1), Declined scan or refusal to participate (*n* = 12), Dropped out or withdrew (*n* = 15), Excessive movement (*n* = 85), Failure to attend session or no-show (*n* = 6), Failure to complete treatment or task (*n* = 10), IQ did not meet level required (*n* = 3), Issues related to data quality (*n* = 58), Lost to follow-up (*n* = 11), Medical reasons (*n* = 12), Missing or incomplete data (*n* = 51), No longer interested or engaged (*n* = 5), Other reasons (*n* = 3), Outliers (*n* = 11), Participant began other treatment (e.g. medication) (*n* = 8), Participant diagnosis changed (*n* = 1), Participant fell asleep during scanning procedure (*n* = 3), Participant found task too complicated (*n* = 1), Participant re-hospitalised (*n* = 3), Poor task performance (*n* = 8), Scanning artefacts (*n* = 17), Scanning contraindications (e.g. metal in body or pregnancy) (*n* = 17), Lack of resources (e.g. researchers, scanning equipment) (*n* = 2), Situational constraints (e.g. travel distance, time constraints, too time-consuming) (*n* = 10), Task noncompliance (*n* = 5), Technical problems or difficulties (*n* = 30), Reasons related to COVID-19 (*n* = 1), Not described or reasons not related to biological data collections/psychopathology (*n* = 271)
Multiple	161	Experimental (*n* = 20), Observational (*n* = 88), Pilot Study (*n* = 3), Protocol (*n* = 33), Not categorised (*n* = 18)	ADHD (*n* = 13), Anxiety (*n* = 6), ASD (*n* = 17), Bipolar Disorder (*n* = 4), Depression (*n* = 30), Eating disorder (*n* = 6), General Psychopathology (*n* = 15), Psychosis (*n* = 9), Substance use disorder (*n* = 3), Other (*n* = 49), Multiple (*n* = 3), Not categorised (*n* = 6)	Adverse events (*n* = 3), Adverse reactions or discomfort during scan or procedure (e.g. became claustrophobic, anxious, panic attack) (*n* = 3), Brain abnormalities detected during scan (neurologic, structural, neuroanatomical, anatomical) (*n* = 2), Caregiver or participant found study unhelpful/believed scope was unwarranted (*n* = 2), Dropped out or withdrew (*n* = 4), Excessive movement (*n* = 8), Failure to attend session or no-show (*n* = 5), Failure to complete task or treatment (*n* = 3), IQ did not meet level required (*n* = 1), Issues related to data quality (*n* = 4), Lost to follow-up (*n* = 8), Medical reasons (*n* = 3), Missing or incomplete data (*n* = 11), No longer interested or engaged (*n* = 3), Outliers (*n* = 1), Participant became anxious about school expectations and developed somatic symptoms (*n* = 1), Participant began other treatment (e.g. medication) (*n* = 1), Participant fell asleep during scan (*n* = 1), Participant found task too complicated (*n* = 1), Participant re-hospitalised (*n* = 2), Poor task performance (*n* = 1), Reasons related to COVID-19 (*n* = 1), Scanning artefacts (*n* = 3), Scanning contraindications (e.g. metal in body or pregnancy) (*n* = 1), Situational constraints (e.g. travel distance, time constraints, too time-consuming) (*n* = 2), Task noncompliance (*n* = 1), Technical problems or difficulties (*n* = 6), Declined scan/refused to participate (*n* = 3), Not described or reasons not related to biological data collections or psychopathology (*n* = 129)
Fluid-based biomarkers	148	Experimental (*n* = 24), Observational (*n* = 97), Pilot Study (*n* = 2), Protocol (*n* = 7), Not categorised (*n* = 18)	ADHD (*n* = 7), Anxiety (*n* = 2), ASD (*n* = 20), Bipolar Disorder (*n* = 7), Depression (*n* = 29), Eating disorder (*n* = 2), General Psychopathology (*n* = 11), Psychosis (*n* = 14), PTSD (*n* = 2), Substance use disorder (*n* = 1), Other (*n* = 44), Multiple (*n* = 7), Not categorised (*n* = 2)	Adverse events (*n* = 1), Adverse reactions or discomfort during scan or procedure (e.g. became claustrophobic, anxious, panic attack) (*n* = 3), Declined scan or refusal to participate (*n* = 2), Dropped out or withdrew (*n* = 6), Failure to complete task or treatment (*n* = 4), Issues related to data quality (*n* = 5), Lost to follow-up (*n* = 1), Medical reasons (*n* = 2), Missing or incomplete data (*n* = 7), Other reasons (*n* = 3), Participant began other treatment (e.g. medication) (*n* = 2), Participant re-hospitalised (*n* = 1), Situational constraints (e.g. travel distance, time constraints, too time-consuming) (*n* = 5), Technical problems or difficulties (*n* = 6), IQ did not meet level required (*n* = 1), Issues related to COVID-19 (*n* = 1), Outliers (*n* = 26), Participant unable to complete task or procedure (*n* = 1), Task noncompliance (*n* = 1), Not described or reasons not related to biological data collections or psychopathology (*n* = 85)
EEG	75	Experimental (*n* = 11), Observational (*n* = 12), Pilot Study (*n* = 4), Protocol (*n* = 3), Not categorised (*n* = 45)	ADHD (*n* = 10), Anxiety (*n* = 7), ASD (*n* = 17), Depression (*n* = 17), General Psychopathology (*n* = 1), Psychosis (*n* = 1), Substance use disorder (*n* = 1), Other (*n* = 17), Multiple (*n* = 1), Not categorised (*n* = 3)	Adverse reactions or discomfort during scan or procedure (e.g. became claustrophobic, anxious, panic attack) (*n* = 1), Began other treatment (e.g. medication) (*n* = 2), Caregiver or participant worried about burden or safety (*n* = 1), Declined scan or refusal to participate (*n* = 5), Dropped out or withdrew (*n* = 6), Excessive movement (*n* = 3), Failure to complete treatment or task (*n* = 1), Failure to attend session or no-show (*n* = 2), IQ did not meet level required (*n* = 1), Issues related to data quality (*n* = 7), Lost to follow-up (*n* = 5), Medical reasons (*n* = 4), Missing or incomplete data (*n* = 6), No longer interested/engaged (*n* = 3), Other reasons (*n* = 4), Participant re-hospitalised (*n* = 2), Reasons related to COVID-19 (*n* = 1), Scanning artefacts (*n* = 3), Scanning contraindications (e.g. metal in body or pregnancy) (*n* = 1), Situational constraints (e.g. travel distance, time constraints, too time-consuming) (*n* = 2), Task noncompliance or disqualified from study (*n* = 3), Technical problems or difficulties (*n* = 2), Not described or reasons not related to biological data collections or psychopathology (*n* = 44)
Other	42	Experimental (*n* = 2), Observational (*n* = 33), Pilot Study (*n* = 2), Not categorised (*n* = 5)	ADHD (*n* = 3), Anxiety (*n* = 1), ASD (*n* = 9), Depression (*n* = 8), Eating disorder (*n* = 3), General Psychopathology (*n* = 2), Psychosis (*n* = 1), PTSD (*n* = 4), Other (*n* = 8), Multiple (*n* = 2), Not categorised (*n* = 1)	Adverse reactions or discomfort during scan or procedure (e.g. became claustrophobic, anxious, panic attack) (*n* = 1), Brain abnormalities detected during scan (neurologic, structural, neuroanatomical, anatomical) (*n* = 1), Declined scan or refusal to participate (*n* = 2), Dropped out or withdrew (*n* = 1), Excessive movement (*n* = 5), IQ did not meet level required (*n* = 1), Issues related to data quality (*n* = 1), Missing or incomplete data (*n* = 4), Outliers (*n* = 1), Poor task performance (*n* = 1), Scanning artefacts (*n* = 2), Situational constraints (e.g. travel distance, time constraints, too time-consuming) (*n* = 3), Technical problems or difficulties (*n* = 2), Failure to attend or no-show (*n* = 3)/Lost to follow-up (*n* = 2), No longer interested or engaged (*n* = 1), Task noncompliance (*n* = 1), Not described or reasons not related to biological data collections or psychopathology (*n* = 25)
Mock Neuroimaging	1	Protocol (*n* = 1)	General psychopathology (*n* = 1)	No longer interested or engaged (*n* = 1)/Lost to follow-up (*n* = 1), Reasons related to COVID-19 (*n* = 1)

ADHD, attention-deficit hyperactivity disorder; ASD, autism spectrum disorder; EEG, electroencephalogram; IQ, intelligence quotient; OCD, obsessive compulsive disorder; PTSD, post traumatic stress disorder.

## Data Availability

Data sharing not applicable to this article as no datasets were generated or analysed during the current study.

## Key points

### What’s known?

- Representativeness in biological mental health research is a growing concern, particularly in adolescence, when development is strongly shaped by economic, social, and ethnic factors.

### What’s new?

- We reviewed 962 studies on biological data collection in youth populations to identify key needs, barriers, and facilitators by looking at recruitment, retention, and engagement methods. Smaller samples were common, recruitment largely occurred in health settings, and reporting was limited; 8.6% described engagement, and fewer than half reported retention data.
- Lack of reporting raises uncertainty about whether facilitators are being implemented, highlighting the need for detailed reporting guidelines.

### What’s relevant?

- As a first step, we provide recommendations for future research, emphasising improved reporting of methods to ensure evidence reflects diverse youth perspectives and informs inclusive, youth-centred clinical care.

## References

[R1] Abrial E, Chalancon B, Leaune E, Brunelin J, Wallon M, Moll F, Poulet E (2022). Investigating predictive factors of suicidal re-attempts in adolescents and young adults after a first suicide attempt, a prospective cohort study. Study Protocol of the SURAYA Project. Frontiers in Psychiatry.

[R2] Arksey H, O’Malley L (2005). Scoping studies: Towards a methodological framework. International Journal of Social Research Methodology.

[R3] Babatunde GB, van Rensburg AJ, Bhana A, Petersen I (2021). Barriers and facilitators to child and adolescent mental health services in low-and-middle-income countries: A scoping review. Global Social Welfare.

[R4] Bezuidenhout L, Chakauya E (2018). Hidden concerns of sharing research data by low/middle-income country scientists. Global Bioethics.

[R5] Bonhauser M, Fernandez G, Püschel K, Yanéz F, Montero J, Thompson B, Coronado G (2005). Improving physical fitness and emotional well-being in adolescents of low socioeconomic status in Chile: Results of a school-based controlled trial. Health Promotion International.

[R6] Braun JM, Buckley JP, Cecil KM, Chen A, Kalkwarf HJ, Lanphear BP, Yolton K (2020). Adolescent follow-up in the Health Outcomes and Measures of the Environment (HOME) study: Cohort profile. BMJ Open.

[R7] Brett J, Staniszewska S, Mockford C, Herron-Marx S, Hughes J, Tysall C, Suleman R (2012). Mapping the impact of patient and public involvement on health and social care research: A systematic review. Health Expectations.

[R8] Burnette CB, Luzier JL, Weisenmuller CM, Boutté RL (2022). A systematic review of sociodemographic reporting and representation in eating disorder psychotherapy treatment trials in the United States. International Journal of Eating Disorders.

[R9] Cavalcante DA, Noto M, Cerqueira RDO, Costa GO, Coutinho L, Malinovski F, Noto C (2024). GAPi: A description of the initiative for early psychosis intervention in Latin America and the short- to medium-term outcomes in early psychosis patients. Asian Journal of Psychiatry.

[R10] Cheung T, Chau B, Fong KH, Lam JYT, Lo H, Li MH, Cheng CPW (2023). Evaluating the efficacy and safety of transcranial pulse stimulation on adolescents with attention deficit hyperactivity disorder: Study protocol of a pilot randomized, double-blind, sham-controlled trial. Frontiers in Neurology.

[R11] Cotton SM, Berk M, Watson A, Wood S, Allott K, Bartholomeusz CF, Dodd S (2019). ENACT: A protocol for a randomised placebo-controlled trial investigating the efficacy and mechanisms of action of adjunctive N-acetylcysteine for first-episode psychosis. Trials.

[R12] Davis BR, Garza A, Church JA (2022). Key considerations for child and adolescent MRI data collection. Frontiers in Neuroimaging.

[R13] Davis S, Pinfold V, Catchpole J, Lovelock C, Senthi B, Kenny A (2024). Reporting lived experience work. The Lancet Psychiatry.

[R14] de Vries J, Bull SJ, Doumbo O, Ibrahim M, Mercereau-Puijalon O, Kwiatkowski D, Parker M (2011). Ethical issues in human genomics research in developing countries. BMC Medical Ethics.

[R15] Dickie EW, Ameis SH, Boileau I, Diaconescu AO, Felsky D, Goldstein BI, Voineskos AN (2024). Neuroimaging and biosample collection in the Toronto Adolescent and Youth Cohort Study: Rationale, methods, and early data. Biological Psychiatry: Cognitive Neuroscience and Neuroimaging.

[R16] Du N, Xiao Y, Li Y, Li C, Li Y, Chen J, Wang P (2025). The role of cytokines in predicting the therapeutic effect of non-suicidal self-injury in adolescents: A longitudinal study. BMC Psychiatry.

[R17] Egbert AH, Hunt RA, Williams KL, Burke NL, Mathis KJ (2022). Reporting racial and ethnic diversity in eating disorder research over the past 20 years. International Journal of Eating Disorders.

[R18] Gindt M, Thümmler S, Soubelet A, Guenolé F, Battista M, Askenazy F (2019). Methodology of “14-7” program: A longitudinal follow-up study of the pediatric population and their families exposed to the terrorist attack of Nice on July 14th, 2016. Frontiers in Psychiatry.

[R19] Hughes EK, Siero W, Gülenç A, Clifford SA, Frugier T, Hall SM, Wake M (2025). Generation Victoria (GenV): Protocol for a longitudinal birth cohort of Victorian children and their parents. BMC Public Health.

[R20] Joanna Briggs Institute (2024). Scoping reviews resources.

[R21] Jong ST, Stevenson R, Winpenny EM, Corder K, van Sluijs EMF (2023). Recruitment and retention into longitudinal health research from an adolescent perspective: A qualitative study. BMC Medical Research Methodology.

[R22] Karlson CW, Rapoff MA (2009). Attrition in randomized controlled trials for pediatric chronic conditions. Journal of Pediatric Psychology.

[R23] Kaufman J, Birmaher B, Perel J, Dahl RE, Moreci P, Nelson B, Ryan ND (1997). The corticotropin-releasing hormone challenge in depressed abused, depressed nonabused, and normal control children. Biological Psychiatry.

[R24] Kessler RC, Berglund P, Demler O, Jin R, Merikangas KR, Walters EE (2005). Lifetime prevalence and age-of-onset distributions of DSM-IV disorders in the National Comorbidity Survey Replication. Archives of General Psychiatry.

[R25] Kidman R, Mwera J, Rui YT, Breton E, Zulu A, Behrman J, Kohler H-P (2024). Cohort profile: The adverse childhood experiences cohort of the Malawi longitudinal study of families and health. BMJ Open.

[R26] Kilicel D, de Crescenzo F, Pontrelli G, Armando M (2023). Participant recruitment issues in child and adolescent psychiatry clinical trials with a focus on prevention programs: A meta-analytic review of the literature. Journal of Clinical Medicine.

[R27] Klasen H, Crombag A-C (2013). What works where? A systematic review of child and adolescent mental health interventions for low and middle income countries. Social Psychiatry and Psychiatric Epidemiology.

[R28] Lee J, Rekhi G, Mitter N, Bong YL, Kraus MS, Lam M, Keefe RSE (2013). The Longitudinal Youth at Risk Study (LYRIKS)—An Asian UHR perspective. Schizophrenia Research.

[R29] Lee KH, Shin J, Lee J, Yoo JH, Kim J-W, Brent DA (2023). Measures of connectivity and dorsolateral prefrontal cortex volumes and depressive symptoms following treatment with selective serotonin reuptake inhibitors in adolescents. JAMA Network Open.

[R30] Leung CY, Chien S, Weiss SJ (2024). Engaging adolescents in research: Home self-collection of biological samples and health questionnaires. Research in Nursing & Health.

[R31] Levac D, Colquhoun H, O’Brien KK (2010). Scoping studies: Advancing the methodology. Implementation Science.

[R32] Liu J, Richmond TS, Raine A, Cheney R, Brodkin ES, Gur RC, Gur RE (2013). The healthy brains and behavior study: Objectives, design, recruitment, and population coverage. International Journal of Methods in Psychiatric Research.

[R33] Liu Y, Pencheon E, Hunter RM, Moncrieff J, Freemantle N (2018). Recruitment and retention strategies in mental health trials – a systematic review. PLoS One.

[R34] Lv R, Cai M, Tang N, Shi Y, Zhang Y, Liu N, Wang H (2024). Active versus sham DLPFC-NAc rTMS for depressed adolescents with anhedonia using resting-state functional magnetic resonance imaging (fMRI): A study protocol for a randomized placebo-controlled trial. Trials.

[R35] Matandika L, Ngóngóla RT, Mita K, Manda-Taylor L, Gooding K, Mwale D, Mfutso-Bengo J (2020). A qualitative study exploring stakeholder perspectives on the use of biological samples for future unspecified research in Malawi. BMC Medical Ethics.

[R36] McGorry PD, Mei C, Dalal N, Alvarez-Jimenez M, Blakemore S-J, Browne V, Killackey E (2024). The Lancet Psychiatry Commission on youth mental health. The Lancet Psychiatry.

[R37] Mitter N, Nah GQR, Bong YL, Lee J, Chong S (2014). Longitudinal Youth-At-Risk Study (LYRIKS): Outreach strategies based on a community-engaged framework. Early Intervention in Psychiatry.

[R38] Moayyedi P, MacQueen G, Bernstein CN, Vanner S, Bercik P, Madsen KL, Fernandes A (2020). IMAGINE Network’s Mind and Gut Interactions Cohort (MAGIC) study: A protocol for a prospective observational multicentre cohort study in inflammatory bowel disease and irritable bowel syndrome. BMJ Open.

[R39] Mockford C, Staniszewska S, Griffiths F, Herron-Marx S (2012). The impact of patient and public involvement on UK NHS health care: A systematic review. International Journal for Quality in Health Care.

[R40] Moher D, Liberati A, Tetzlaff J, Altman DG (2010). Preferred reporting items for systematic reviews and meta-analyses: The PRISMA statement. International Journal of Surgery.

[R41] Montgomery KE, Basha M, Nyholm L, Smith C, Ananiev G, Fedorov A, Kwekkeboom K (2024). Exploring inflammation and stress as biological correlates of symptoms in children with advanced cancer: A longitudinal feasibility study. Journal of Pediatric Hematology/Oncology Nursing.

[R42] National Institute of Mental Health (2024). Looking at my genes: What can they tell me about my mental health?.

[R43] Nicol GE, Yingling MD, Flavin KS, Schweiger JA, Patterson BW, Schechtman KB, Newcomer JW (2018). Metabolic effects of antipsychotics on adiposity and insulin sensitivity in youths. JAMA Psychiatry.

[R44] Oliver MI, Pearson N, Coe N, Gunnell D (2005). Help-seeking behaviour in men and women with common mental health problems: Cross-sectional study. British Journal of Psychiatry.

[R45] Parrish DE, Duron JF, Oxhandler HK (2017). Adolescent recruitment strategies: Lessons learned from a university-based study of social anxiety. Social Work Research.

[R46] Patel V, Sumathipala A (2001). International representation in psychiatric literature. British Journal of Psychiatry.

[R47] Pedrini L, Rossi R, Magni LR, Lanfredi M, Meloni S, Ferrari C, Cattaneo A (2021). Emotional Regulation in Teens and Improvement of Constructive Skills (EmoTI-ConS): Study protocol for a randomized controlled trial. Trials.

[R48] Peters MDJ, Godfrey CM, Khalil H, McInerney P, Parker D, Soares CB (2015). Guidance for conducting systematic scoping reviews. International Journal of Evidence-Based Healthcare.

[R49] Piccin J, Viduani A, Buchweitz C, Pereira RB, Zimerman A, Amando GR, Kieling C (2024). Prospective follow-up of adolescents with and at risk for depression: Protocol and methods of the identifying depression early in adolescence risk stratified cohort longitudinal assessments. JAACAP Open.

[R50] Pollock D, Peters MDJ, Khalil H, McInerney P, Alexander L, Tricco AC, Munn Z (2023). Recommendations for the extraction, analysis, and presentation of results in scoping reviews. JBI Evidence Synthesis.

[R51] Razzouk D, Sharan P, Gallo C, Gureje O, Lamberte EE, de Jesus Mari J, Saxena S (2010). Scarcity and inequity of mental health research resources in low-and-middle income countries: A global survey. Health Policy.

[R52] Reiss F (2013). Socioeconomic inequalities and mental health problems in children and adolescents: A systematic review. Social Science & Medicine.

[R53] Roberts H, Jacobs RH, Bessette KL, Crowell SE, Westlund-Schreiner M, Thomas L, Watkins ER (2021). Mechanisms of rumination change in adolescent depression (RuMeChange): Study protocol for a randomised controlled trial of rumination-focused cognitive behavioural therapy to reduce ruminative habit and risk of depressive relapse in high-ruminating adolescents. BMC Psychiatry.

[R54] Rodriguez J, Huntington-Moskos L, Johnson A, Williams S, Gulledge E, Feeley C, Rice M (2016). Collecting biological measures for research with children and adolescents. Journal of Pediatric Health Care.

[R55] Rubia K, Johansson L, Carter B, Stringer D, Santosh P, Mehta MA, Cortese S (2024). The efficacy of real versus sham external trigeminal nerve stimulation (eTNS) in youth with attention-deficit/hyperactivity disorder (ADHD) over 4 weeks: A protocol for a multi-centre, double-blind, randomized, parallel-group, phase IIb study (ATTENS). BMC Psychiatry.

[R56] Ruphrect-Smith H, Davies S, Jacob J, Edbrooke-Childs J (2024). Ethnic differences in treatment outcome for children and young people accessing mental health support. European Child & Adolescent Psychiatry.

[R57] Schmidt NL, Van Hulle CA, Brooker RJ, Meyer LR, Lemery-Chalfant K, Goldsmith HH (2013). Wisconsin twin research: Early development, childhood psychopathology, autism, and sensory over-responsivity. Twin Research and Human Genetics.

[R58] Schoeppe S, Oliver M, Badland HM, Burke M, Duncan MJ (2014). Recruitment and retention of children in behavioral health risk factor studies: REACH strategies. International Journal of Behavioral Medicine.

[R59] Siless V, Hubbard NA, Jones R, Wang J, Lo N, Bauer CCC, Yendiki A (2020). Image acquisition and quality assurance in the Boston Adolescent Neuroimaging of Depression and Anxiety study. NeuroImage: Clinical.

[R60] Smith AJ, Moreno-López L, Davidson E, Dauvermann M, Orellana S, Soneson E, van Harmelen A-L (2021). REACT study protocol: Resilience after the COVID-19 threat (REACT) in adolescents. BMJ Open.

[R61] Smith SA, Duncan AA (2022). Systematic and scoping reviews: A comparison and overview. Seminars in Vascular Surgery.

[R62] Sonuga-Barke EJS, Chandler S, Lukito S, Kakoulidou M, Moore G, Cooper N, Pavlopoulou G (2024). Participatory translational science of neurodivergence: Model for attention-deficit/hyperactivity disorder and autism research. The British Journal of Psychiatry.

[R63] Spark J, Rowe E, Alvarez-Jimenez M, Bell I, Byrne L, Dzafic I, Nelson B (2025). Integrating virtual reality, neurofeedback, and cognitive behavioral therapy for auditory verbal hallucinations (hybrid): Protocol of a pilot, unblinded, single-arm interventional study. JMIR Research Protocols.

[R64] Staniszewska S, Brett J, Mockford C, Barber R (2011). The GRIPP checklist: Strengthening the quality of patient and public involvement reporting in research. International Journal of Technology Assessment in Health Care.

[R65] Tobe RH, MacKay-Brandt A, Lim R, Kramer M, Breland MM, Tu L, Milham MP (2022). A longitudinal resource for studying connectome development and its psychiatric associations during childhood. Scientific Data.

[R66] Trapp G, Giles-Corti B, Martin K, Timperio A, Villanueva K (2012). Conducting field research in a primary school setting: Methodological considerations for maximizing response rates, data quality and quantity. Health Education Journal.

[R67] Trickett PK, Noll JG, Putnam FW (2011). The impact of sexual abuse on female development: Lessons from a multigenerational, longitudinal research study. Development and Psychopathology.

[R68] Trivedi MH, Chin Fatt CR, Jha MK, Cooper CM, Trombello JM, Mason BL, Greer TL (2020). Comprehensive phenotyping of depression diseasetrajectory and risk: Rationale and design of Texas Resilience Against Depression study (T-RAD). Journal of Psychiatric Research.

[R69] Viksveen P, Cardenas NE, Ibenfeldt M, Meldahl LG, Krijger L, Game JR, Tong M (2022). Involvement of adolescent Representatives and coresearchers in mental health research: Experiences from a research project. Health Expectations.

[R70] Wall CA, Croarkin PE, Maroney-Smith MJ, Haugen LM, Baruth JM, Frye MA, Port JD (2016). Magnetic resonance imaging-guided, open-label, high-frequency repetitive transcranial magnetic stimulation for adolescents with major depressive disorder. Journal of Child and Adolescent Psychopharmacology.

[R71] Warne N, Rook S, Bevan Jones R, Brown R, Bates L, Hopkins-Jones L, Collishaw S (2022). Collecting genetic samples and linked mental health data from adolescents in schools: Protocol coproduction and a mixed-methods pilot of feasibility and acceptability. BMJ Open.

[R72] Warner TD, Behnke M, Eyler FD, Szabo NJ (2011). Early adolescent cocaine use as determined by hair analysis in a prenatal cocaine exposure cohort. Neurotoxicology and Teratology.

[R73] Woodall A, Morgan C, Sloan C, Howard L (2010). Barriers to participation in mental health research: Are there specific gender, ethnicity and age related barriers?. BMC Psychiatry.

[R74] World Health Organization (2025a). Mental health of adolescents.

[R75] World Health Organization (2025b). Over a billion people living with mental health conditions – services require urgent scale-up.

[R76] Worrell C, Pollard R, Weetman T, Sadiq Z, Pieptan M, Brooks G, Upthegrove R (2024). Exploring the research needs, barriers and facilitators to the collection of biological data in adolescence for mental health research: A scoping review protocol paper. BMJ Open.

[R77] Youn S, Mamsa S, Allott K, Berger M, Polari A, Rice S, Lavoie S (2023). Acceptability and feasibility of a multidomain harmonized data collection protocol in youth mental health. Early Intervention in Psychiatry.

[R78] Zajkowska Z, Walsh A, Zonca V, Gullett N, Pedersen GA, Kieling C, Mondelli V (2021). A systematic review of the association between biological markers and environmental stress risk factors for adolescent depression. Journal of Psychiatric Research.

