# Supplementary material for: Research Review: A scoping review exploring the needs, barriers, and facilitators to the collection of biological data in adolescence for mental health research
Source: J Child Psychol Psychiatry. Author manuscript; Available in PMC 2026 Sep 8. (PMC7619467; doi:10.1111/jcpp.70215)
Supplement: Supplementary file 1 [file EMS218152-supplement-Supplementary_Material.docx]

**A scoping review exploring the needs, barriers, and facilitators to the collection of biological data in adolescence for mental health research.**

**Supporting Information**

Table S1: A summary of engagement strategies described in the included papers

| **Citation** | **Type of paper** | **Young people engagement** | **Stakeholder engagement** | **Community engagement** | **Feedback**  **strategies** |
| --- | --- | --- | --- | --- | --- |
| Bent et al., (2018) ‘Identification of urinary metabolites that correlate with clinical improvements in children with autism treated with sulforaphane from broccoli’ (Bent et al., 2018) | Data paper | None described | None described | Researcher and community relationship | Caregiver feedback |
| Bonhauser et al., (2005) ‘Improving physical fitness and emotional well-being in adolescents of low socioeconomic status in Chile: results of a school-based controlled trial’ (Bonhauser et al., 2005) | Data paper | None described | School or teacher collaboration and facilitation in the development of the study | None described | None described |
| Braun et al., (2020) ‘Adolescent follow-up in the Health Outcomes and Measures of the Environment (HOME) Study: cohort profile’ (Braun et al., 2020) | Protocol, methodology or cohort profile | Dissemination of findings to participants | None described | None described | Participant feedback |
| Bray et al., (2024) ‘Puberty and NeuroDevelopment in adolescents (PANDA): a study protocol’ | Protocol, methodology or cohort profile | Non-cash incentives beyond standard procedure | None described | None described | None described |
| Cascio et al., (2023) ‘Effect of Technology and Digital Media Use on Adolescent Health and Development: Protocol for a Multimethod Longitudinal Study’ (Cascio et al., 2023) | Protocol, methodology or cohort profile | Building rapport with participants  Non-cash incentives beyond standard procedure  Regular contact beyond standard procedure | None described | None described | Participant feedback |
| Cavalcante et al., (2024) ‘GAPi: A description of the initiative for early psychosis intervention in Latin America and the short- to medium-term outcomes in early psychosis patients’(Cavalcante et al., 2024) | Data paper | None described | Stakeholder collaboration and facilitation in the development of the study | None described | None described |
| Corrivetti et al., (2024) ‘Optimizing and Predicting Antidepressant Efficacy in Patients with Major Depressive Disorder Using Multi-Omics Analysis and the Opade AI Prediction Tools.’ (Corrivetti et al., 2024) | Protocol, methodology or cohort profile | None described | None described | None described | Participant feedback |
| Cotton et al., (2019) ‘ENACT: a protocol for a randomised placebo-controlled trial investigating the efficacy and mechanisms of action of adjunctive *N-*acetylcysteine for first-episode psychosis’ (Cotton et al., 2019) | Protocol, methodology or cohort profile | Youth friendly procedures  Disseminating findings to participants | None described | None described | None described |
| Crone et al., (2024) Growing ‘Up Together in Society (GUTS): A team science effort to predict societal trajectories in adolescence and young adulthood’ (Crone et al., 2024) | Protocol, methodology or cohort profile | Implementing youth ideas | Stakeholder collaboration and facilitation in the development of the study  Parent/guarding collaboration and facilitation in the development of the study | None described | Participant feedback |
| de la Serna et al., (2023) ‘Effectiveness of enhancing cognitive reserve in children, adolescents and young adults at genetic risk for psychosis: Study protocol for a randomized controlled trial’ (de la Serna et al., 2021) | Protocol, methodology or cohort profile | Youth friendly procedures | None described | None described | None described |
| Dickie et al., (2024) ‘Neuroimaging and Biosample Collection in the Toronto Adolescent and Youth Cohort Study: Rationale, Methods, and Early Data’ (Dickie et al., 2024) | Protocol, methodology or cohort profile | Youth friendly procedures  Non-cash incentives beyond standard procedure  Mock scanner/training for familiarisation of scanning environment beyond standard procedure  Implementing youth ideas | Parent/guardian collaboration and facilitation in the development of the study | None described | None described |
| Ewais et al., (2019) ‘Protocol for a pilot randomised controlled trial of mindfulness-based cognitive therapy in youth with inflammatory bowel disease and depression’ (Ewais et al., 2019)  Ewais et al., (2019) ‘Mindfulness based cognitive therapy for youth with inflammatory bowel disease and depression – Findings from a pilot randomised controlled trial’ (Ewais et al., 2021) | Protocol, methodology or cohort profile  and  Data paper | Implementing youth ideas  Disseminating findings to participants  Building rapport with participants  Youth friendly procedures | None described | None described | Participant feedback |
| Frommelt et al., (2023) ‘Study protocol for a randomized controlled trial investigating the effect of online interpretation bias intervention on stress reactivity in the children of parents with depression: the CoCo study’ (Frommelt et al., 2023) | Protocol, methodology or cohort profile | Youth friendly procedures | None described | None described | Participant feedback |
| Gindt et al., (2019) ‘Methodology of “14-7” Program: A Longitudinal Follow-Up Study of the Pediatric Population and Their Families Exposed to the Terrorist Attack of Nice on July 14^th^, 2016’ (Gindt et al., 2019) | Protocol, methodology or cohort profile | Regular contact beyond standard procedure | None described | None described | None described |
| Goldberg et al., (2002) ‘Deficits in the initiation of eye movements in the absence of a visual target in adolescents with high functioning autism’ (Goldberg et al., 2002) | Data paper | Mock scanner/training for familiarisation of scanning environment beyond standard procedure | None described | None described | None described |
| Grootendorst-van Mil et al., (2021) ‘The iBerry study: a longitudinal cohort study of adolescents at high risk of psychopathology’ (Grootendorst-van Mil et al., 2021) | Protocol, methodology or cohort profile | Non-cash incentives beyond standard procedure  Regular contact beyond standard procedure | None described | None described | None described |
| Häberling et al., (2019) ‘Omega-3 Fatty Acids as a Treatment for Pediatric Depression. A Phase III, 36 Weeks, Multi-Center, Double-Blind, Placebo-Controlled Randomized Superiority Study’ (Häberling et al., 2019) | Protocol, methodology or cohort profile | None described | None described | Dissemination of research to community | None described |
| Hemmings et al., (2024) ‘A randomised controlled feasibility trial of intermittent theta burst stimulation with an open longer-term follow-up for young people with persistent anorexia nervosa (RaISE): study protocol’ (Hemmings et al., 2024) | Protocol, methodology or cohort profile | Youth friendly procedures  Implementing youth ideas | Stakeholder collaboration and facilitation in the development of the study | None described | Participant feedback |
| Hughes et al., (2025) ‘Generation Victoria (GenV): protocol for a longitudinal birth cohort of Victorian children and their parents’ | Protocol, methodology or cohort profile | None described | Stakeholder collaboration and facilitations in the development of the study | Community involvement in research | Caregiver feedback |
| Iadipaolo et al., (2018) ‘Distinct neural correlates of trait resilience within core neurocognitive networks in at-risk children and adolescents’ (Iadipaolo et al., 2018) | Data paper | Mock scanner/training for familiarisation of scanning environment beyond standard procedure | None described | None described | None described |
| Islam et al., (2023) ‘Change in Urinary Inflammatory Biomarkers and Psychological Health with Gut Microbiome Modulation after Six Months of a Lifestyle Modification Program in Children’ (Islam et al., 2023) | Data paper | Educational opportunities | None described | None described | None described |
| Iveson et al., (2024) ‘Cohort profile: The Scottish SHARE Mental Health (SHARE-MH) cohort - linkable survey, genetic and routinely collected data for mental health research’ (Iveson et al., 2024) | Protocol, methodology or cohort profile | None described | Stakeholder collaboration and facilitation in the development of the study | None described | None described |
| Kaufman et al., (1997) ‘The corticotropin-releasing hormone challenge in depressed abused, depressed nonabused, and normal control children’ (Kaufman et al., 1997) | Data paper | Youth friendly procedures  Close attention and monitoring | None described | None described | None described |
| Kidman et al., (2024) ‘Pathways between adverse childhood experiences and viral suppression among male HIV-infected adolescents in South Africa’ (Kidman et al., 2024) | Data paper | Youth friendly procedures  Implementing youth ideas  Close attention and monitoring | Stakeholder collaboration and facilitation in the development of the study | Community involvement in research | None described |
| Kidman et al., (2024) ‘ Cohort profile: the Adverse Childhood Experiences cohort of the Malawi Longitudinal Study of Families and Health’ (Kidman et al., 2024) | Protocol, methodology or cohort profile | None described | Stakeholder collaboration and facilitation in the development of the study | Community involvement in research | None described |
| Kildal et al., (2023) ‘Heart rate monitoring to detect acute pain in non-verbal patients: a study protocol for a randomized controlled clinical trial’ (Kildal et al., 2023) | Protocol, methodology or cohort profile | None described | Stakeholder collaboration and facilitation in the development of the study | None described | None described |
| Larsen et al., (2024) ‘Children at Familial High risk of Schizophrenia and Bipolar Disorder Exhibit Altered Connectivity Patterns During Pre-attentive Processing of an Auditory Prediction Error’ (Larsen et al., 2024) | Data paper | Youth friendly procedures | None described | None described | None described |
| Ledoux et al., (2024) ‘Smartphone App–Delivered Mindfulness-Based Intervention for Mild Traumatic Brain Injury in Adolescents: Protocol for a Feasibility Randomized Controlled Trial’ (Ledoux et al., 2024) | Protocol, methodology or cohort profile | None described | Stakeholder collaboration and facilitation in the development of the study | Pilot tested study to community | None described |
| Lee et al., (2013) ‘The Longitudinal Youth at Risk Study (LYRIKS) – An Asian UHR perspective’ (Lee et al., 2013) | Protocol, methodology or cohort profile | Regular contact beyond standard procedure | None described | None described | None described |
| Leung et al., (2024) ‘Engaging adolescents in research: Home self-collection of biological samples and health questionnaires’ (Leung et al., 2024) | Data paper | Youth friendly procedures  Building rapport with participants | Providing information to parents/guardians | None described | None described |
| Liu et al., (2013) ‘The Healthy Brains and Behavior Study: objectives, design, recruitment, and population coverage’ (J. Liu et al., 2013) | Protocol, methodology or cohort profile | Youth friendly procedures | School or teacher collaboration and facilitation in the development of study | None described | None described |
| Livny & Silberg, (2023) ‘Puberty, brain network connectivity and neuropsychiatric outcomes following pediatric traumatic brain injury in females: A research protocol’ (Livny & Silberg, 2023) | Protocol, methodology or cohort profile | None described | Stakeholder collaboration and facilitation in the development of the study | None described | None described |
| Malhi et al., (2019) ‘Effect of stress gene-by-environment interactions on hippocampal volumes and cortisol secretion in adolescent girls’ (Malhi et al., 2019) | Data paper | None described | School or teacher collaboration and facilitation in the development of study | None described | None described |
| Mamah et al., (2024) ‘Kenya Psychosis-Risk Outcomes Study (KePROS): Development of an Accelerated Medicine Partnership Schizophrenia-Aligned Project in Africa’ (Mamah et al., 2024) | Protocol, methodology or cohort profile | None described | None described | Community involvement in research | None described |
| Man et al., (2023) ‘Long-term safety of methylphenidate in children and adolescents with ADHD: 2-year outcomes of the Attention Deficit Hyperactivity Disorder Drugs Use Chronic Effects (ADDUCE) study’ (Man et al., 2023) | Data paper | None described | Stakeholder collaboration and facilitation in the development of the study | None described | None described |
| Manasse et al., (2024) ‘Evaluating an acceptance-based lifestyle modification program to address cardiovascular disease risk among adolescent girls with overweight and obesity: Protocol for a randomized controlled trial.’ (Manasse et al., 2024) | Protocol, methodology or cohort profile | None described | None described | Community involvement in research | None described |
| May et al., (2024) ‘The role of environmental sensitivity in the mental health of Syrian refugee children: a multi-level analysis’ (May et al., 2024) | Data paper | None described | None described | Pilot tested study to community | None described |
| Moayyedi et al., (2020) ‘IMAGINE Network’s Mind and Gut Interactions Cohort (MAGIC) Study: a protocol for a prospective observational multicentre cohort study in inflammatory bowel disease and irritable bowel syndrome’ (Moayyedi et al., 2020) | Protocol, methodology or cohort profile | None described | None described | Community involvement in research | Participant feedback |
| Montgomery et al., (2024) ‘Exploring Inflammation and Stress as Biological Correlates of Symptoms in Children With Advanced Cancer: A Longitudinal Feasibility Study’ (Montgomery et al., 2024) | Data paper | None described | Stakeholder collaboration and facilitation in the development of the study | None described | Participant and Caregiver Feedback |
| Mortazavi et al., (2022) ‘Moderate-to-vigorous group aerobic exercise versus group leisure activities for mild-to-moderate depression in adolescents: study protocol for a multicentre randomised controlled trial’ (Mortazavi et al., 2022) | Protocol, methodology or cohort profile | Implementing youth ideas | None described | None described | Participant feedback |
| Mouga et al., (2022) ‘Parahippocampal deactivation and hyperactivation of central executive, saliency and social cognition networks in autism spectrum disorder’ (Mouga et al., 2022) | Data paper | Mock scanner/training for familiarisation of scanning environment beyond standard procedure | None described | Community involvement in research | None described |
| Mudra Rakshasa-Loots et al., (2025) ‘Metabolic biomarkers of clinical outcomes in severe mental illness (METPSY): protocol for a prospective observational study in the Hub for metabolic psychiatry’ | Protocol, methodology or cohort profile | Implementing youth ideas | None described | Community involvement in research | Participant feedback |
| Nabwera et al., (2021) ‘Menstrual hygiene management practices and associated health outcomes among school-going adolescents in rural Gambia’ (Nabwera et al., 2021) | Data paper | None described | None described | Pilot tested study to community | None described |
| Nagashima et al., (2014) ‘Neuropharmacological effect of atomoxetine on attention network in children with attention deficit hyperactivity disorder during oddball paradigms as assessed using functional near-infrared spectroscopy’ (Nagashima et al., n.d.) | Data paper | Youth friendly procedures | None described | None described | None described |
| Naim et al., (2021) ‘Across-subjects multiple baseline trial of exposure-based cognitive-behavioural therapy for severe irritability: a study protocol’ (Naim et al., 2021) | Protocol, methodology or cohort profile | None described | None described | Researcher and community relationship | Participant feedback |
| Nicol et al., (2018) ‘Metabolic Effects of Antipsychotics on Adiposity and Insulin Sensitivity in Youths’ (Nicol et al., 2018) | Data paper | Regular contact beyond standard procedure | None described | None described | None described |
| Okada et al., (2023) ‘My Diet Study: protocol for a two-part observational, longitudinal, psycho-biological study of dieting in Australian youth’ (Okada et al., 2023) | Protocol, methodology or cohort profile | Non-cash incentives beyond standard procedure | Stakeholder collaboration and facilitation in the development of the study | None described | None described |
| Özel et al., (2023) ‘Exploring gender dysphoria and related outcomes in a prospective cohort study: protocol for the Swedish Gender Dysphoria Study (SKDS)’ (Özel et al., 2023) | Protocol, methodology or cohort profile | None described | Stakeholder collaboration and facilitation in the development of the study | None described | None described |
| Pedrini et al., (2021) ‘Emotional Regulation in Teens and Improvement for Constructive Skills (EmoTIConS): study protocol for a randomized controlled trial’ (Pedrini et al., 2021) | Protocol, methodology or cohort profile | None described | School or teacher collaboration and facilitation in the development of the study | None described | None described |
| Qiao et al., (2025) ‘The possible effect of inflammation on non-suicidal self-injury in adolescents with depression: a mediator of connectivity within corticostriatal reward circuitry’ | Data paper | Non-cash incentives beyond standard procedure | None described | None described | None described |
| Rohrsetzer et al., (2023) ‘A magnetic resonance imaging-based morphometric and structural covariance network study of Brazilian adolescents stratified by depression risk’ (Rohrsetzer et al., 2023)  Piccin et al., (2024) ‘Prospective Follow-Up of Adolescents with and at Risk for Depression: Protocol and Methods of the Identifying Depression Early in Adolescence Risk Stratified Cohort Longitudinal Assessments’ (Piccin et al., n.d.) | Data paper  And  Protocol, methodology or cohort profile | Youth friendly procedures | None described | None described | Participant feedback |
| Rubia et al., (2024) ‘The efficacy of real versus sham external Trigeminal Nerve Stimulation (eTNS) in youth with Attention-Deficit/Hyperactivity Disorder (ADHD) over 4 weeks: a protocol for a multi-centre, double-blind, randomized, parallel-group, phase IIb study (ATTENS)’ (Rubia et al., 2024) | Protocol, methodology or cohort profile | None described | None described | Dissemination of research to community | None described |
| Salum et al., (2015) ‘High risk cohort study for psychiatric disorders in childhood: rationale, design, methods and preliminary results’ (Salum et al., 2015) | Protocol, methodology or cohort profile | Mock scanner/training for familiarisation of scanning environment beyond standard procedure  Youth friendly procedures  Close attention and monitoring | Providing information to parents/guardians  Referrals and risk assessments provided | None described | None described |
| Scherff et al., (2024) ‘Cohort profile: BioMD-Y (biopsychosocial factors of major depression in youth) - a biobank study on the molecular genetics and environmental factors of depression in children and adolescents in Munich’ (Scherff et al., 2024) | Protocol, methodology or cohort profile | Youth friendly procedures  Implementing youth ideas | Parent or guardian collaboration and facilitation in the development of the study | None described | Participant feedback |
| Schmidt et al., (2013) ‘Wisconsin Twin Research: Early Development, Childhood Psychopathology, Autism, and Sensory Over-responsivity’ (Schmidt et al., 2013)  Schmidt et al., (2019) ‘Wisconsin Twin Project Overview: Temperament and Affective Neuroscience’ (Schmidt et al., 2019) | Protocol, methodology or cohort profile  And  Data Paper | None described | Stakeholder collaboration and facilitation in the development of the study | None described | None described |
| Schwartzman et al., (2025) ‘Study protocol for a multimethod investigation of the development of social and nonsocial reward responsivity and depression in autistic adolescents: Reward and Depression in Autism (RDA)’ | Protocol, methodology or cohort profile | Disseminating findings to participants | Providing information to parents/guardians | Dissemination of research to community | None described |
| Siless et al., (2020) ‘Image acquisition and quality assurance in the Boston Adolescent Neuroimaging of Depression and Anxiety study’ (Siless et al., 2020) | Protocol, methodology or cohort profile | Mock scanner/training for familiarisation of scanning environment beyond standard procedure  Youth friendly procedures  Close attention and monitoring | None described | None described | None described |
| Smith et al., (2021) ‘REACT study protocol: resilience after the COVID-19 threat (REACT) in adolescents’ (A. J. Smith et al., 2021) | Protocol, methodology or cohort profile | None described | None described | None described | Participant feedback |
| Stunden et al., (2021) ‘Comparing a Virtual Reality-Based Simulation App (VR-MRI) With a Standard Preparatory Manual and Child Life Program for Improving Success and Reducing Anxiety During Pediatric Medical Imaging: Randomized Clinical Trial’ (Stunden et al., 2021) | Data paper | Mock scanner/training for familiarisation of scanning environment beyond standard procedure  Building rapport with participant | Stakeholder collaboration and facilitation in the development of the study | None described | Participant and caregiver feedback |
| Tobe et al., (2022) ‘A longitudinal resource for studying connectome development and its psychiatric associations during childhood’ (Tobe et al., 2022a) | Protocol, methodology or cohort profile | Educational opportunities  Disseminating findings to participants | None described | Community involvement in research | Participant feedback |
| Toma et al., (2025) ‘Cerebral Blood Flow and Mood in Adolescents With Bipolar Disorder’ | Data paper | None described | None described | Community involvement in research | None described |
| Trickett et al., (2011) ‘The impact of sexual abuse on female development: Lessons from a multigenerational, longitudinal research study’ (Trickett et al., 2011) | Data paper | Non-cash incentives beyond standard procedure  Educational opportunities | None described | None described | None described |
| Trivedi et al., (2020) ‘Comprehensive phenotyping of depression disease trajectory and risk: Rationale and design of Texas Resilience Against Depression study (T-RAD)’ (Trivedi et al., 2020) | Protocol, methodology or cohort profile | Non-cash incentives beyond standard procedure | None described | None described | None described |
| Tseng et al., (2024) ‘Characterizing Autism Spectrum Disorder Through Fusion of Local Cortical Activation and Global Functional Connectivity Using Game-Based Stimuli and a Mobile EEG System’ (Tseng et al., 2024) | Data paper | Non-cash incentives beyond standard procedure | None described | None described | None described |
| Tweedie-Cullen et al., (2024) ‘Protocol for the Gut Bugs in Autism Trial: a double-blind randomised placebo-controlled trial of faecal microbiome transfer for the treatment of gastrointestinal symptoms in autistic adolescents and adults’ (Tweedie-Cullen et al., 2024) | Protocol, methodology or cohort profile | None described | None described | Community involvement in research | None described |
| Vaquero et al., (2025) ‘Studying the effects of digital arts-based interventions on teenagers’ social media usage, brain connectivity, and mental health: study protocol of the SMART project’ | Protocol, methodology or cohort profile | Youth friendly procedures | School or teacher collaboration in facilitation and development of study | None described | Participant feedback |
| Vreeker et al., (2024) ‘The Mood and Resilience in Offspring (MARIO) project: a longitudinal cohort study among offspring of parents with and without a mood disorder’ (Vreeker et al., 2024) | Protocol, methodology or cohort profile | Implementing youth ideas | Stakeholder collaboration and facilitation in the development of the study | None described | None described |
| Wannan et al., (2024) ‘Accelerating Medicines Partnership® Schizophrenia (AMP® SCZ): Rationale and Study Design of the Largest Global Prospective Cohort Study of Clinical High Risk for Psychosis’ (Wannan et al., 2024) | Protocol, methodology or cohort profile | None described | Stakeholder collaboration and facilitation in the development of the study | None described | None described |
| Weigensberg et al., (2018) ‘Protocol for the Imagine HEALTH Study: Guided imagery lifestyle intervention to improve obesity-related behaviors and salivary cortisol pattens in predominantly Latino adolescents.’ (Weigensberg et al., 2018) | Protocol, methodology or cohort profile | Building rapport with participant  Non-cash incentives beyond standard procedure | School or teacher collaboration and facilitation in the development of the study | None described | None described |
| Wilson et al., (2023) ‘An open-label pilot trial of faecal microbiome transfer to restore the gut microbiome in anorexia nervosa: protocol’ (Wilson et al., 2023) | Protocol, methodology or cohort profile | None described | Stakeholder collaboration and facilitation in the development of the study | None described | None described |
| Yarger et al., (2025) ‘Assessing anxiety in adolescents with autism spectrum disorder (ADORA): developing a health equity protocol for non-invasive biomarkers’ | Protocol, methodology or cohort profile | Youth friendly procedures | None described | None described | None described |
| Yates et al., (2025) ‘Investigative Approaches to Resilient Emotion Regulation in Neurodevelopment in a South African Birth Cohort’ | Protocol, methodology or cohort profile | Close attention and monitoring   Building rapport with participant   Implementing youth ideas | Providing information to parents/guardians | None described | None described |
| Youn et al., (2023) ‘Acceptability and feasibility of a multidomain harmonized data collection protocol in youth mental health’ (Youn et al., 2023) | Protocol, methodology or cohort profile | None described | None described | None described | Participant feedback |
| Zhang et al., (2022) ‘A cohort study of adolescents with depression in China: tracking multidimensional outcomes and early biomarkers for intervention’ (Zhang et al., 2022) | Protocol, methodology or cohort profile | None described | Providing information to parents/guardians | None described | None described |

Table S2: A summary of retention information described in included papers.

| **Citation** | **Type of paper** | **Retention methods reported** | **Reports any retention statistics** |
| --- | --- | --- | --- |
| Abrial et al., (2022) ‘Investigating Predictive Factors of Suicidal Re-attempts in Adolescents and Young Adults After a First Suicide Attempt, a Prospective Cohort Study. Study Protocol of the SURAYA Project’ (Abrial et al., 2022) | Protocol, methodology or cohort profile | Regular follow up period  Phone calls replacing face-to-face visits | No |
| Amatoury et al., (2018) ‘Salivary cortisol reveals overt and hidden anxiety in survivors of childhood cancer attending clinic’ (Amatoury et al., 2018) | Data paper | Reminder phone calls specific to at-home data collection | Yes  55 patients consented, 46 remained after participants who failed to complete minimum tasks were excluded. 3 collected samples ≤ 2 days from start date |
| Braun et al., (2020) ‘Adolescent follow-up in the Health Outcomes and Measures of the Environment (HOME) Study: cohort profile’ (Braun et al., 2020) | Data paper | Newsletters, mailings, and emails  Social media  Publicised study results  Home visits  Small gifts | Yes  30 mothers dropped out during run-in phase of RCT. At 12-year visit, 10 of these children completed visit after being recontacted. Completed follow-up on 256 (58%) of children.  185 did not complete follow-up - 99 (54%) refused, 23 (12%) were lost to follow-up and 63 (34%) were out of age range. |
| Butera et al., (2023) ‘The relationship between alexithymia, interoception, and neural functional connectivity during facial expression processing in autism spectrum disorder’ (Butera et al., 2023) | Data paper | Participants trained in mock scanner before scanning | No |
| Cavalcante et al., (2024) ‘GAPi: A description of the initiative for early psychosis intervention in Latin America and the short- to medium-term outcomes in early psychosis patients’ (Cavalcante et al., 2024) | Data paper | Regular follow-ups | Yes  Dropout rates were approximately 34.05 % at 2 months, 79.31 % at 1 year, and 92.24 % at 5 years |
| Cheung et al., (2023) ‘Evaluating the efficacy and safety of transcranial pulse stimulation on adolescents with attention deficit hyperactivity disorder: study protocol of a pilot randomized, double-blind, sham-controlled trial’ (Cantone et al., n.d.) | Protocol, methodology or cohort profile | Reminders by phone or text | No |
| Cotton et al., (2019) ‘ENACT: a protocol for a randomised placebo-controlled trial investigating the efficacy and mechanisms of action of adjunctive N-acetylcysteine for first-episode psychosis’ (Cotton et al., 2019) | Protocol, methodology or cohort profile | Flexibility applied to timing and location of assessments (e.g home visits, after school hours) | No |
| Du et al., (2025) ‘The role of cytokines in predicting the therapeutic effect of non-suicidal self-injury in afdolescents: a longitudinal study’ | Data paper | Reminders via WeChat group and electronic links to questionnaires on their statuses | Yes  2 participants died by suicide before the first follow-up, and 1 participant dropped out at the second follow-up due to unwillingness to follow doctors’ prescription |
| Gindt et al., (2019) ‘Methodology of “14-7” Program: A Longitudinal Follow-Up Study of the Pediatric Population and Their Families Exposed to the Terrorist Attack of Nice on July 14th, 2016’ (Gindt et al., 2019) | Protocol, methodology or cohort profile | Regular contact with participants and families | No |
| Hegarty II et al, (2024) ‘A Twin Study of Altered White Matter Heritability in Youth With Autism Spectrum Disorder’ (Hegarty et al., n.d.) | Data paper | Participants trained in mock scanner before scanning | Yes  Diffusion-weighted data were available from 84 complete twin pairs (N = 168). Could not obtain scans from both twins for 6 twin pairs |
| Kidman et al., (2024) ‘Cohort profile: the Adverse Childhood Experiences cohort of the Malawi Longitudinal Study of Families and Health’ | Protocol, methodology or cohort profile | Attempts to recontact those who moved location  Provided 24 hour hotline for support | Yes  2061 enrolled. Wave 1 – 2061 (+ 1438 caregivers). Wave 2 – 1878 (+208 offspring).  333 ineligible, 37 died, 193 not found/temporarily absent, 158 not attempted contact, 238 moved out of catchment areas, 13 refused. Lost to follow up – 2 died, 26 temporarily absent, 111 moved out of catchment area, 3 refused, 41 other reasons. |
| Lee et al., (2013) ‘The Longitudinal Youth at Risk Study (LYRIKS) – an Asian UHR perspective’ (Lee et al., 2013) | Protocol, methodology or cohort profile | Regular contact and monitoring of participants by phone/email | Recruitment statistics not retention |
| Leung et al., (2024) ‘Engaging adolescents in research: Home self-collection of biological samples and health questionnaires’ (Leung et al., 2024) | Data paper | Regular contact with participants and families  Flexibility applied to data collection  Reminders by phone | Yes  Screened 116 adolescents, 110 were eligible and enrolled. 21 either withdrew or were lost to follow-up, leaving a total sample of 90.88 completed questionnaires, 80 completed stool samples, 85 completed saliva samples for cytokines and 81 for cortisol (80 awakening, 79 for 45 min, 78 for 4pm, 76 for before bedtime). |
| Lv et al., (2024) ‘Active versus sham DLPFC-NAc rTMS for depressed adolescents with anhedonia using resting-state functional magnetic resonance imaging (fMRI): a study protocol for a randomized placebo-controlled trial’ (Lv et al., 2024) | Protocol, methodology or cohort profile | Reminders for follow-ups | No |
| Monaco et al., (2024) ‘Innova4Health: an integrated approach for prevention of recurrence and personalized treatment of Major Depressive Disorder.’ (Monaco et al., 2024) | Protocol, methodology or cohort profile | Website/mobile app for participant communication | No |
| Montgomery et al., (2024) ‘Exploring Inflammation and Stress as Biological Correlates of Symptoms in Children With Advanced Cancer: A Longitudinal Feasibility Study.’ (Montgomery et al., 2024) | Data paper | Reminders by phone or text | Yes  Across all participants and time points, 97% of blood samples and 83% of saliva samples were collected. Of the 199 saliva samples, 43 specimens for salivary cortisol and 70 for sAA were unanalysable due to insufficient quantity of saliva. Participants completed 84% of saliva surveys. |
| Nicol et al., (2018) ‘Metabolic Effects of Antipsychotics on Adiposity and Insulin Sensitivity in Youths’ (Nicol et al., 2018) | Data paper | Regular contact with participants and families  Reminder phone calls  Newsletters | Yes  13 withdrew consent, 17 were lost to follow-up, 4 were excluded |
| Oberman et al., (2024) ‘Design and methodology for a proof of mechanism study of individualized neuronavigated continuous Theta burst stimulation for auditory processing in adolescents with autism spectrum disorder’ (Oberman et al., 2024) | Protocol, methodology or cohort profile | Participants trained in mock scanner before scanning  Flexibility applied to accommodate schedules | No |
| Pedrini et al., (2021) ‘Emotional Regulation in Teens and Improvement of Constructive Skills (EmoTIConS): study protocol for a randomised controlled trial’ (Pedrini et al., 2021) | Protocol, methodology or cohort profile | Assessments during school time (and planned in agreement with school personnel)  Teachers identified as contact persons  Reminders by phone | No |
| Piccin et al., (2024) ‘Prospective Follow-Up of Adolescents With and at Risk for Depression: Protocol and Methods of the Identifying Depression Early in Adolescence Risk Stratified Cohort Longitudinal Assessments.’(Piccin et al., n.d.) | Protocol, methodology or cohort profile | Regular contact with participants and families  Home visits | Yes  Retention rates at timepoints 1, 2, and 3 were 96% (n = 144), 94% (n = 141), and 88% (n = 132) respectively. 83.3% (n = 125) completed at least 1 data point for each of the 4 data collection timepoints. |
| Portnova et al., (2023) ‘New approaches to clinical electroencephalography analysis in typically developing children and children with autism’ (Portnova et al., 2023) | Data paper | Participants trained in mock scanner before scanning | No |
| Roberts et al., (2021) ‘Mechanisms of Rumination Change in Adolescent Depression (RuMeChange): study protocol for a randomised controlled trial of rumination-focused cognitive behavioural therapy to reduce ruminative habit and risk of depressive relapse in high-ruminating adolescents’ (Roberts et al., 2021) | Protocol, methodology or cohort profile | Set contingency plans in case of retention issues including increased advertisement, outreach resources, and phone calls | No |
| Rubia et al., (2024) ‘The efficacy of real versus sham external Trigeminal Nerve Stimulation (eTNS) in youth with Attention-Deficit/Hyperactivity Disorder (ADHD) over 4 weeks: a protocol for a multi-centre, double-blind, randomized, parallel-group, phase IIb study (ATTENS).’ (Rubia et al., 2024) | Protocol, methodology or cohort profile | Flexibility applied to accommodate schedules  Phone calls to replace face to face visits | No |
| Schmidt et al., (2019) ‘Wisconsin Twin Project Overview: Temperament and Affective Neuroscience’ (Schmidt et al., 2019)  Schmidt et al., (2013) ‘Wisconsin Twin Research: Early Development, Childhood Psychopathology, Autism, and Sensory Over-responsivity’ (Schmidt et al., 2013) | Protocol, methodology or cohort profile  And  Protocol, methodology or cohort profile | Newsletters  Website devoted to participant communication  Participant tracking | No |
| Spark et al., (2025) ‘Integrating Virtual Reality, Neurofeedback, and Cognitive Behavioral Therapy for Auditory Verbal Hallucinations (Hybrid): Protocol of a Pilot Unblinded, Single-Arm Interventional Study’ | Protocol, methodology or cohort profile | Reminders by phone or text | 75 individuals approached, 64 pre-screened (41 eligible, 15 ineligible, 8 declined/lost to follow-up). 5 individuals have been included so far, with 1 withdrawn |
| Tobe et al., (2022) ‘A longitudinal resource for studying connectome development and its psychiatric associations during childhood’ (Tobe et al., 2022b) | Protocol, methodology or cohort profile | Birthday cards  Participants invited to events and talks  Collect and implement participant feedback  Retest scans for missed visits  Maximised availability on non-school days | Yes  326 completed entire baseline, 247 completed midpoint and 177 completed final visit, 207 completed retest visits. |
| Trickett et al., (2011) ‘The impact of sexual abuse on female development: Lessons from a multigenerational, longitudinal research study’ (Trickett et al., 2011) | Data paper | Home visits where contact could not be made by phone  Monetary incentives for phone calls  Participant tracing  Frequent mailings – newsletters, birthday/holiday/milestone cards | Yes  Retention rates across timepoint 2–6 ranged from 84%-88%. Timepoints 4–6 retention rates were 87%, 86%, and 88% respectively. Over 96% of the sample was retained and reassessed at timepoint 4, 5, and/or 6. 166 completed follow-ups. |
| Trivedi et al., (2020) ‘Comprehensive phenotyping of depression disease and risk: Rationale and design of Texas Resilience Against Depression study (T-RAD)’ (Trivedi et al., 2020) | Protocol, methodology or cohort profile | Flexibility applied to assessments (split visits across days and completion by phone/mail/computer) | No |
| Wall et al., (2016) ‘Magnetic Resonance Imaging-Guided, Open-Label, High-Frequency Repetitive Transcranial Magnetic Stimulation for Adolescents with Major Depressive Disorder’ (Wall et al., 2016) | Data paper | Flexibility applied to accommodate variability in schedules (e.g., related to school, illness or family events) | Yes  3 did not complete treatment (1 due to discomfort, 1 was hospitalised, 1 withdrew). |
| Warner et al., (2011) ‘Early adolescent cocaine use as determined by hair analysis in a prenatal cocaine exposure cohort’ (Warner et al., 2011) | Data paper | Assessments outside of the home (in mobile clinic) or at school | Yes  90% participated in the follow-up interviews and/or neuropsychological  evaluations between ages 10½ and 12½. 263 provided hair samples at one or both ages. |
| Weigensberg et al., (2018) ‘Protocol for the Imagine HEALTH Study: guided imagery lifestyle intervention to improve obesity-related behaviors and saliva patterns in predominantly Latino adolescents’ (Weigensberg et al., 2018) | Protocol, methodology or cohort profile | Reminder phone calls and texts  Phone calls and texts for lateness or missed sessions | Recruitment statistics not retention |
| Weissman et al., (2022) ‘Exposure to Violence as an Environmental Pathway Linking Low Socioeconomic Status with Altered Neural Processing of Threat and Adolescent Psychopathology’ (Weissman et al., 2022) | Data paper | Participants trained in mock scanner before scanning  Participants shown motion tracker to indicate head motion in scanner | No |
| You et al., (2023) ‘Mapping access to meaning in adolescents with autism: Atypical lateralization and spatiotemporal patterns as a function of language abilty | Data paper | Mock scanning and familiarisation of environment | No |

Appendix S1: Search Terms

("MRI" OR "fMRI" OR "EEG" OR "PET" OR "neuroimaging" OR "biological research" OR "biological sample*" OR "biologic*" OR "blood sample* " OR "urine sample*") AND ("mental health" OR "mental" OR "depression" OR "anxiety" OR "self-harm" OR "self-harm" OR "suicide" OR "psychiatric" OR "psychopathology" OR "mood disorder" OR "autism" OR "neurodivergence") AND ("youth*" OR "adolescent" OR "adolescence" OR "teen*" OR "teenager*" OR "high school student*" OR "AYA") AND ("retention" OR "recruitment" OR "recruit" OR "recruited" OR "enroll" OR "enrol" OR "enrollment" OR "enrolment" OR "enrolling" OR "enrolled" OR "participation")

Appendix S2: Additional records not listed in the main manuscript

Abbas, M., Gandy, K., Salas, R., Devaraj, S., & Calarge, C. A. (2023). Iron deficiency and internalizing symptom severity in unmedicated adolescents: a pilot study. *Psychological Medicine*, *53*(6), 2274–2284. https://doi.org/10.1017/S0033291721004098

Abbas, M., Gandy, K., Salas, R., Devaraj, S., & Calarge, C. A. (2024). Investigation of Carnitine Levels in some Biological Fluids. *Tropical Journal of Natural Product Research*, *8*(1). https://doi.org/10.26538/tjnpr/v8i1.41

Abbo, N., Mitchell, T., Tonekaboni, S. H., Anagnostou, E., Andrade, B. F., Thorpe, K., & Beal, D. S. (2025). Feasibility and usability of remote transcranial direct current stimulation (tDCS) for self-regulation in children with autism: protocol for a randomized controlled pilot study. *Pilot and Feasibility Studies*, *11*(1), 57. https://doi.org/10.1186/s40814-025-01650-4

Abdulamir, H. A., Abdul-Rasheed, O. F., & Abdulghani, E. A. (2018). Serotonin and serotonin transporter levels in autistic children. *Saudi Medical Journal*, *39*(5), 487–494. https://doi.org/10.15537/smj.2018.5.21751

Abe, J., Jafarpour, S., Vu, M. H., O’Brien, D., Boyd, N. K., Vogel, B. N., Nguyen, L., Paulsen, K. C., Saucier, L. E., Ahsan, N., Mitchell, W. G., & Santoro, J. D. (2023). Impact of endocrine dysregulation on disability and non-motor symptoms in pediatric onset multiple sclerosis. *Frontiers in Neurology*, *14*. https://doi.org/10.3389/fneur.2023.1304610

Acuff, H. E., Versace, A., Bertocci, M. A., Ladouceur, C. D., Hanford, L. C., Manelis, A., Monk, K., Bonar, L., McCaffrey, A., Goldstein, B. I., Goldstein, T. R., Sakolsky, D., Axelson, D., Birmaher, B., & Phillips, M. L. (2018). Association of Neuroimaging Measures of Emotion Processing and Regulation Neural Circuitries With Symptoms of Bipolar Disorder in Offspring at Risk for Bipolar Disorder. *JAMA Psychiatry*, *75*(12), 1241. https://doi.org/10.1001/jamapsychiatry.2018.2318

Addington, J., Goldstein, B. I., Wang, J. L., Kennedy, S. H., Bray, S., Lebel, C., Hassel, S., Marshall, C., & MacQueen, G. (2018). Youth at-risk for serious mental illness: methods of the PROCAN study. *BMC Psychiatry*, *18*(1), 219. https://doi.org/10.1186/s12888-018-1801-0

Adler, C. M., DelBello, M. P., Mills, N. P., Schmithorst, V., Holland, S., & Strakowski, S. M. (2005). Comorbid ADHD is associated with altered patterns of neuronal activation in adolescents with bipolar disorder performing a simple attention task. *Bipolar Disorders*, *7*(6), 577–588. https://doi.org/10.1111/j.1399-5618.2005.00257.x

Aggarwal, N., Williams, L. E., Tromp, D. P. M., Pine, D. S., & Kalin, N. H. (2022). A dynamic relation between whole-brain white matter microstructural integrity and anxiety symptoms in preadolescent females with pathological anxiety. *Translational Psychiatry*, *12*(1), 57. https://doi.org/10.1038/s41398-022-01827-y

Aghamohammadi-Sereshki, A., McMorris, C. A., Gibbard, W. ben, Tortorelli, C., Pike, G. B., & Lebel, C. (2025). Properties of the prefrontal tracts and cingulum bundle in children with prenatal alcohol exposure. *Journal of Affective Disorders*, *369*, 164–173. https://doi.org/10.1016/J.JAD.2024.09.165

Alegria, A. A., Wulff, M., Brinson, H., Barker, G. J., Norman, L. J., Brandeis, D., Stahl, D., David, A. S., Taylor, E., Giampietro, V., & Rubia, K. (2017). Real‐time f <scp>MRI</scp> neurofeedback in adolescents with attention deficit hyperactivity disorder. *Human Brain Mapping*, *38*(6), 3190–3209. https://doi.org/10.1002/hbm.23584

Alexander-Bloch, A., Huguet, G., Schultz, L. M., Huffnagle, N., Jacquemont, S., Seidlitz, J., Saci, Z., Moore, T. M., Bethlehem, R. A. I., Mollon, J., Knowles, E. K., Raznahan, A., Merikangas, A., Chaiyachati, B. H., Raman, H., Schmitt, J. E., Barzilay, R., Calkins, M. E., Shinohara, R. T., … Glessner, J. (2022). Copy Number Variant Risk Scores Associated With Cognition, Psychopathology, and Brain Structure in Youths in the Philadelphia Neurodevelopmental Cohort. *JAMA Psychiatry*, *79*(7), 699. https://doi.org/10.1001/jamapsychiatry.2022.1017

Alghadir, A. H., Gabr, S. A., & Al-Eisa, E. (2016). Effects of Physical Activity on Trace Elements and Depression Related Biomarkers in Children and Adolescents. *Biological Trace Element Research*, *172*(2), 299–306. https://doi.org/10.1007/s12011-015-0601-3

Ali, E., Embaby, A., Arafa, S. M., Elbana, A. K., Ghazala, M., & Ibrahim, D. (2024). Electroconvulsive therapy improves hematological inflammatory markers in bipolar disorder. *Psychopharmacology*, *241*(2), 351–357. https://doi.org/10.1007/s00213-023-06491-8

Allen, P., Baldwin, H., Bartholomeusz, C. F., Chee, M. W., Chen, X., Cooper, R. E., de Haan, L., Hamilton, H. K., He, Y., Hegelstad, W. ten V., Horton, L. E., Hubl, D., Klaunig, M. J., Koppel, A., Kwak, Y. bin, León-Ortiz, P., Loewy, R. L., McGorry, P., Ouyang, L., … Frangou, S. (2024). Normative Modeling of Brain Morphometry in Clinical High Risk for Psychosis. *JAMA Psychiatry*, *81*(1), 77. https://doi.org/10.1001/jamapsychiatry.2023.3850

Allison, G. O., Freeman, C., Renault, H., Banica, I., Ethridge, P., Sandre, A., & Weinberg, A. (2023). Risk factors for the intergenerational transmission of depression in women and girls: Understanding neural correlates of major depressive disorder and the role of early-onset maternal depression. *Cognitive, Affective, & Behavioral Neuroscience*, *23*(2), 400–414. https://doi.org/10.3758/s13415-023-01063-x

Amatoury, M., Maguire, A. M., Olivier, J., Barton, B., Gabriel, M., Dalla-Pozza, L., Steinbeck, K. S., & Battisti, R. A. (2018). Salivary cortisol reveals overt and hidden anxiety in survivors of childhood cancer attending clinic. *Journal of Affective Disorders*, *240*, 105–112. https://doi.org/10.1016/J.JAD.2018.07.035

Arnold, L. E., Lofthouse, N., Hersch, S., Pan, X., Hurt, E., Bates, B., Kassouf, K., Moone, S., & Grantier, C. (2013). EEG Neurofeedback for ADHD. *Journal of Attention Disorders*, *17*(5), 410–419. https://doi.org/10.1177/1087054712446173

Arora, P., Sagar, R., Mehta, M., Pallavi, P., Sharma, S., & Mukhopadhyay, A. K. (2019). Serum S100B levels in patients with depression. *Indian Journal of Psychiatry*, *61*(1), 70–76. https://doi.org/10.4103/psychiatry.IndianJPsychiatry_391_16

Arruda, J. M., Bogetz, A. L., Vellanki, S., Wren, A., & Yeh, A. M. (2018). Yoga as adjunct therapy for adolescents with inflammatory bowel disease: A pilot clinical trial. *Complementary Therapies in Medicine*, *41*, 99–104. https://doi.org/10.1016/j.ctim.2018.09.007

Assaf, R., Ouellet, J., Bourque, J., Stip, E., Leyton, M., Conrod, P., & Potvin, S. (2022). A functional neuroimaging study of self-other processing alterations in atypical developmental trajectories of psychotic-like experiences. *Scientific Reports*, *12*(1), 16324. https://doi.org/10.1038/s41598-022-20129-3

Asta, L., Ricciardello, A., Cucinotta, F., Turriziani, L., Boncoddo, M., Bellomo, F., Angelini, J., Gnazzo, M., Scandolo, G., Pisanò, G., Pelagatti, F., Chehbani, F., Camia, M., & Persico, A. M. (2024). Clinical, developmental and serotonemia phenotyping of a sample of 70 Italian patients with Phelan-McDermid Syndrome. *Journal of Neurodevelopmental Disorders*, *16*(1), 57. https://doi.org/10.1186/s11689-024-09572-7

Auerbach, R. P., Pagliaccio, D., Allison, G. O., Alqueza, K. L., & Alonso, M. F. (2021). Neural Correlates Associated With Suicide and Nonsuicidal Self-injury in Youth. *Biological Psychiatry*, *89*(2), 119–133. https://doi.org/10.1016/j.biopsych.2020.06.002

Badura Brack, A. S., Marklin, M., Embury, C. M., Picci, G., Frenzel, M., Klanecky Earl, A., Stephen, J., Wang, Y.-P., Calhoun, V., & Wilson, T. W. (2022). Neurostructural brain imaging study of trait dissociation in healthy children. *BJPsych Open*, *8*(5), e172. https://doi.org/10.1192/bjo.2022.576

Bahn, G. H., Hong, M., Lee, K. M., Lee, C., Ryu, C.-W., Lee, J.-A., Park, S., Kim, E. J., & Jahng, G.-H. (2018). The relationship between microstructural alterations of the brain and clinical measurements in children and adolescents with hair pulling disorder. *Brain Imaging and Behavior*, *12*(2), 477–487. https://doi.org/10.1007/s11682-017-9716-9

Bai, J., Eldridge, R., Houser, M., Martin, M., Powell, C., Sutton, K. S., Noh, H. I., Wu, Y., Olson, T., Konstantinidis, K. T., & Bruner, D. W. (2024). Multi-omics analysis of the gut microbiome and metabolites associated with the psychoneurological symptom cluster in children with cancer receiving chemotherapy. *Journal of Translational Medicine*, *22*(1), 256. https://doi.org/10.1186/s12967-024-05066-1

Baker, A. E., Padgaonkar, N. T., Galván, A., & Peris, T. S. (2024). Anxiety may alter the role of fronto-striatal circuitry in adolescent risky decision-making. *Journal of Affective Disorders*, *348*, 238–247. https://doi.org/10.1016/j.jad.2023.12.063

Baker, E., Veytsman, E., Choy, T., Blacher, J., & Stavropoulos, K. K. M. (2021). Investigating Changes in Reward-Related Neural Correlates After PEERS Intervention in Adolescents With ASD: Preliminary Evidence of a “Precision Medicine” Approach. *Frontiers in Psychiatry*, *12*. https://doi.org/10.3389/fpsyt.2021.742280

Barbosa, A. G., Pratesi, R., Paz, G. S. C., dos Santos, M. A. A. L., Uenishi, R. H., Nakano, E. Y., Gandolfi, L., & Pratesi, C. B. (2020). Assessment of BDNF serum levels as a diagnostic marker in children with autism spectrum disorder. *Scientific Reports*, *10*(1), 17348. https://doi.org/10.1038/s41598-020-74239-x

Barch, D. M., Donohue, M. R., Elsayed, N. M., Gilbert, K., Harms, M. P., Hennefield, L., Herzberg, M., Kandala, S., Karcher, N. R., Jackson, J. J., Luking, K. R., Rappaport, B. I., Sanders, A., Taylor, R., Tillman, R., Vogel, A. C., Whalen, D., & Luby, J. L. (2022). Early Childhood Socioeconomic Status and Cognitive and Adaptive Outcomes at the Transition to Adulthood: The Mediating Role of Gray Matter Development Across Five Scan Waves. *Biological Psychiatry: Cognitive Neuroscience and Neuroimaging*, *7*(1), 34–44. https://doi.org/10.1016/j.bpsc.2021.07.002

Barendse, M. E. A., Vijayakumar, N., Byrne, M. L., Flannery, J. E., Cheng, T. W., Flournoy, J. C., Nelson, B. W., Cosme, D., Mobasser, A., Chavez, S. J., Hval, L., Brady, B., Nadel, H., Helzer, A., Shirtcliff, E. A., Allen, N. B., & Pfeifer, J. H. (2020). Study Protocol: Transitions in Adolescent Girls (TAG). *Frontiers in Psychiatry*, *10*. https://doi.org/10.3389/fpsyt.2019.01018

Bartholdy, S., O’Daly, O. G., Campbell, I. C., Banaschewski, T., Barker, G., Bokde, A. L. W., Bromberg, U., Büchel, C., Quinlan, E. B., Desrivières, S., Flor, H., Frouin, V., Garavan, H., Gowland, P., Heinz, A., Ittermann, B., Martinot, J.-L., Paillère Martinot, M.-L., Nees, F., … Stedman, A. (2019). Neural Correlates of Failed Inhibitory Control as an Early Marker of Disordered Eating in Adolescents. *Biological Psychiatry*, *85*(11), 956–965. https://doi.org/10.1016/j.biopsych.2019.01.027

Bartholomeusz, C. F., Ganella, E. P., Whittle, S., Allott, K., Thompson, A., Abu-Akel, A., Walter, H., McGorry, P., Killackey, E., Pantelis, C., & Wood, S. J. (2018). An fMRI study of theory of mind in individuals with first episode psychosis. *Psychiatry Research: Neuroimaging*, *281*, 1–11. https://doi.org/10.1016/J.PSCYCHRESNS.2018.08.011

Bashford‐Largo, J., Aloi, J., Lukoff, J., Johnson, K., White, S. F., Dobbertin, M., Blair, R. J., & Blair, K. S. (2021). Reduced top‐down attentional control in adolescents with generalized anxiety disorder. *Brain and Behavior*, *11*(2). https://doi.org/10.1002/brb3.1994

Bat-Pitault, F., da Fonseca, D., Cortese, S., le Strat, Y., Kocher, L., Rey, M., Adrien, J., Deruelle, C., & Franco, P. (2013). The sleep macroarchitecture of children at risk for depression recruited in sleep centers. *European Psychiatry*, *28*(3), 168–173. https://doi.org/10.1016/j.eurpsy.2012.02.007

Battaglia, A., & Carey, J. C. (2006). Etiologic yield of autistic spectrum disorders: A prospective study. *American Journal of Medical Genetics Part C: Seminars in Medical Genetics*, *142C*(1), 3–7. https://doi.org/10.1002/ajmg.c.30076

Bednarz, H. M., Maximo, J. O., Murdaugh, D. L., O’Kelley, S., & Kana, R. K. (2017). “Decoding versus comprehension”: Brain responses underlying reading comprehension in children with autism. *Brain and Language*, *169*, 39–47. https://doi.org/10.1016/j.bandl.2017.01.002

Beesdo‐Baum, K., Voss, C., Venz, J., Hoyer, J., Berwanger, J., Kische, H., Ollmann, T. M., & Pieper, L. (2020). The Behavior and Mind Health (BeMIND) study: Methods, design and baseline sample characteristics of a cohort study among adolescents and young adults. *International Journal of Methods in Psychiatric Research*, *29*(1). https://doi.org/10.1002/mpr.1804

Belem da Silva, C. T., de Abreu Costa, M., Kapczinski, F., de Aguiar, B. W., Salum, G. A., & Manfro, G. G. (2017). Inflammation and internalizing disorders in adolescents. *Progress in Neuro-Psychopharmacology and Biological Psychiatry*, *77*, 133–137. https://doi.org/10.1016/j.pnpbp.2017.03.023

Bellis, M. D., Narasimhan, A., Thatcher, D. L., Keshavan, M. S., Soloff, P., & Clark, D. B. (2005). Prefrontal Cortex, Thalamus, and Cerebellar Volumes in Adolescents and Young Adults with Adolescent-Onset Alcohol Use Disorders and Comorbid Mental Disorders. *Alcoholism: Clinical and Experimental Research*, *29*(9), 1590–1600. https://doi.org/10.1097/01.alc.0000179368.87886.76

Beneria, A., Motger-Albertí, A., Quesada-Franco, M., Arteaga-Henríquez, G., Santesteban-Echarri, O., Ibáñez, P., Parramon-Puig, G., Sanz-Correcher, P., Galynker, I., Ramos-Quiroga, J. A., Pintor, L., Bruguera, P., & Braquehais, M. D. (2024). A Suicide Attempt Multicomponent Intervention Treatment (SAMIT Program): study protocol for a multicentric randomised controlled trial. *BMC Psychiatry*, *24*(1), 666. https://doi.org/10.1186/s12888-024-06113-3

Bent, S., Lawton, B., Warren, T., Widjaja, F., Dang, K., Fahey, J. W., Cornblatt, B., Kinchen, J. M., Delucchi, K., & Hendren, R. L. (2018). Identification of urinary metabolites that correlate with clinical improvements in children with autism treated with sulforaphane from broccoli. *Molecular Autism*, *9*(1), 35. https://doi.org/10.1186/s13229-018-0218-4

Berg, L. M., Gurr, C., Leyhausen, J., Seelemeyer, H., Bletsch, A., Schaefer, T., Pretzsch, C. M., Oakley, B., Loth, E., Floris, D. L., Buitelaar, J. K., Beckmann, C. F., Banaschewski, T., Charman, T., Jones, E. J. H., Tillmann, J., Chatham, C. H., Bourgeron, T., Ahmad, J., … Ecker, C. (2023). The neuroanatomical substrates of autism and ADHD and their link to putative genomic underpinnings. *Molecular Autism*, *14*(1), 36. https://doi.org/10.1186/s13229-023-00568-z

Berger, M., Taylor, S., Harriss, L., Campbell, S., Thompson, F., Jones, S., Sushames, A., Amminger, G. P., Sarnyai, Z., & McDermott, R. (2019). Hair cortisol, allostatic load, and depressive symptoms in Australian Aboriginal and Torres Strait Islander people. *Stress*, *22*(3), 312–320. https://doi.org/10.1080/10253890.2019.1572745

Berk, L., Stewart, J. L., May, A. C., Wiers, R. W., Davenport, P. W., Paulus, M. P., & Tapert, S. F. (2015). Under pressure: adolescent substance users show exaggerated neural processing of aversive interoceptive stimuli. *Addiction*, *110*(12), 2025–2036. https://doi.org/10.1111/add.13090

Bezek, J. L., Tillem, S., Suarez, G. L., Burt, S. A., Vazquez, A. Y., Michael, C., Sripada, C., Kump, K. L., & Hyde, L. W. (2024). Functional brain network organization and multidomain resilience to neighborhood disadvantage in youth. *American Psychologist*, *79*(8), 1123–1138. https://doi.org/10.1037/amp0001279

Bhargava, S., Zundel, C. G., Shampine, M., Ely, S., Carpenter, C., Losiowski, J., Chanamolu, S., Jande, J., Tamimi, R., Sharma, K., O’Mara, E., Jaster, A. M., & Marusak, H. A. (2025). Puberty, sex, and fear extinction retention: A neuroimaging study in youth. *Developmental Cognitive Neuroscience*, *75*, 101595. https://doi.org/10.1016/j.dcn.2025.101595

Bharthi, K., Zuberi, R., Maruf, A. al, Shaheen, S. M., McCloud, R., Heintz, M., McAusland, L., Arnold, P. D., & Bousman, C. A. (2024). Impact of Cytochrome P450 Genetic Variation on Patient-Reported Symptom Improvement and Side Effects Among Children and Adolescents Treated with Fluoxetine. *Journal of Child and Adolescent Psychopharmacology*, *34*(1), 21–27. https://doi.org/10.1089/cap.2023.0039

Bilgiç, A., Toker, A., Işık, Ü., & Kılınç, İ. (2017). Serum brain-derived neurotrophic factor, glial-derived neurotrophic factor, nerve growth factor, and neurotrophin-3 levels in children with attention-deficit/hyperactivity disorder. *European Child & Adolescent Psychiatry*, *26*(3), 355–363. https://doi.org/10.1007/s00787-016-0898-2

Bioulac, S., Purper-Ouakil, D., Ros, T., Blasco-Fontecilla, H., Prats, M., Mayaud, L., & Brandeis, D. (2019). Personalized at-home neurofeedback compared with long-acting methylphenidate in an european non-inferiority randomized trial in children with ADHD. *BMC Psychiatry*, *19*(1), 237. https://doi.org/10.1186/s12888-019-2218-0

BIRMAHER, B., RABIN, B. S., GARCIA, M. R., JAIN, U., WHITESIDE, T. L., WILLIAMSON, D. E., AL-SHABBOUT, M., NELSON, B. C., DAHL, R. E., & RYAN, N. D. (1994). Cellular Immunity in Depressed, Conduct Disorder, and Normal Adolescents: Role of Adverse Life Events. *Journal of the American Academy of Child & Adolescent Psychiatry*, *33*(5), 671–678. https://doi.org/10.1097/00004583-199406000-00008

Bjork, J. M., Chen, G., Smith, A. R., & Hommer, D. W. (2010). Incentive‐elicited mesolimbic activation and externalizing symptomatology in adolescents. *Journal of Child Psychology and Psychiatry*, *51*(7), 827–837. https://doi.org/10.1111/j.1469-7610.2009.02201.x

Bjork, J. M., Knutson, B., & Hommer, D. W. (2008). Incentive‐elicited striatal activation in adolescent children of alcoholics. *Addiction*, *103*(8), 1308–1319. https://doi.org/10.1111/j.1360-0443.2008.02250.x

Blackwell, C. K., Cella, D., Adair, L., Cordero, J. F., Das, S. R., Elliott, A. J., Hipwell, A. E., Jacobson, L. P., Neiderhiser, J. M., Stanford, J. B., Wright, R. J., & Gershon, R. (2024). Extending the Environmental influences on Child Health Outcomes (ECHO) Cohort through 2030: Rationale and study protocol. *PLOS ONE*, *19*(12), e0312677. https://doi.org/10.1371/journal.pone.0312677

Blardi, P., de Lalla, A., Ceccatelli, L., Vanessa, G., Auteri, A., & Hayek, J. (2010). Variations of plasma leptin and adiponectin levels in autistic patients. *Neuroscience Letters*, *479*(1), 54–57. https://doi.org/10.1016/J.NEULET.2010.05.027

Bloom, P. A., Pagliaccio, D., Zhang, J., Bauer, C. C. C., Kyler, M., Greene, K. D., Treves, I., Morfini, F., Durham, K., Cherner, R., Bajwa, Z., Wool, E., Olafsson, V., Lee, R. F., Bidmead, F., Cardona, J., Kirshenbaum, J. S., Ghosh, S., Hinds, O., … Auerbach, R. P. (2023). Mindfulness-based real-time fMRI neurofeedback: a randomized controlled trial to optimize dosing for depressed adolescents. *BMC Psychiatry*, *23*(1), 757. https://doi.org/10.1186/s12888-023-05223-8

Blumenthal, H., Leen-Feldner, E. W., Knapp, A. A., Bunaciu, L., & Zamboanga, B. L. (2012). Alcohol use history and panic-relevant responding among adolescents: A test using a voluntary hyperventilation challenge. *Psychology of Addictive Behaviors*, *26*(4), 683–692. https://doi.org/10.1037/a0027364

Boes, A. D., McCormick, L. M., Coryell, W. H., & Nopoulos, P. (2008). Rostral Anterior Cingulate Cortex Volume Correlates with Depressed Mood in Normal Healthy Children. *Biological Psychiatry*, *63*(4), 391–397. https://doi.org/10.1016/J.BIOPSYCH.2007.07.018

Bogdan, R., Williamson, D. E., & Hariri, A. R. (2012). Mineralocorticoid Receptor Iso/Val (rs5522) Genotype Moderates the Association Between Previous Childhood Emotional Neglect and Amygdala Reactivity. *American Journal of Psychiatry*, *169*(5), 515–522. https://doi.org/10.1176/appi.ajp.2011.11060855

Bölte, S., Willfors, C., Berggren, S., Norberg, J., Poltrago, L., Mevel, K., Coco, C., Fransson, P., Borg, J., Sitnikov, R., Toro, R., Tammimies, K., Anderlid, B.-M., Nordgren, A., Falk, A., Meyer, U., Kere, J., Landén, M., Dalman, C., … Lichtenstein, P. (2014). The Roots of Autism and ADHD Twin Study in Sweden (RATSS). *Twin Research and Human Genetics*, *17*(3), 164–176. https://doi.org/10.1017/thg.2014.12

Bolton, T. A. W., Freitas, L. G. A., Jochaut, D., Giraud, A.-L., & van de Ville, D. (2020). Neural responses in autism during movie watching: Inter-individual response variability co-varies with symptomatology. *NeuroImage*, *216*, 116571. https://doi.org/10.1016/j.neuroimage.2020.116571

Boomsma, D. I., Vink, J. M., van Beijsterveldt, T. C. E. . M., Geus, E. J. C. de, Beem, A. L., Mulder, E. J. C. M., Derks, E. M., Riese, H., Willemsen, G. A. H. M., Bartels, M., van den Berg, M., Kupper, N. H. M., Polderman, T. J. C., Posthuma, D., Rietveld, M. J. H., Stubbe, J. H., Knol, L. I., Stroet, T., & van Baal, G. C. M. (2002). Netherlands Twin Register: A Focus on Longitudinal Research. *Twin Research*, *5*(05), 401–406. https://doi.org/10.1375/twin.5.5.401

Bos, D. J., Oranje, B., Veerhoek, E. S., van Diepen, R. M., Weusten, J. M., Demmelmair, H., Koletzko, B., de Sain-van der Velden, M. G., Eilander, A., Hoeksma, M., & Durston, S. (2015). Reduced Symptoms of Inattention after Dietary Omega-3 Fatty Acid Supplementation in Boys with and without Attention Deficit/Hyperactivity Disorder. *Neuropsychopharmacology*, *40*(10), 2298–2306. https://doi.org/10.1038/npp.2015.73

Böttinger, B. W., Aggensteiner, P.-M., Hohmann, S., Heintz, S., Ruf, M., Glennon, J., Holz, N. E., Banaschewski, T., Brandeis, D., & Baumeister, S. (2024). Exploring real-time functional magnetic resonance imaging neurofeedback in adolescents with disruptive behavior disorder and callous unemotional traits. *Journal of Affective Disorders*, *345*, 32–42. https://doi.org/10.1016/j.jad.2023.10.036

Bounoua, N., Church, L. D., Matyi, M. A., Rudoler, J., Wieand, K., & Spielberg, J. M. (2023). Assessing the utility of a novel cortical marker of delay discounting (C-DD) in two independent samples of early adolescents: Links with externalizing pathology. *PLOS ONE*, *18*(9), e0291868. https://doi.org/10.1371/journal.pone.0291868

Bounoua, N., Joseph, J. E., Adams, Z. W., Crum, K. I., Sege, C. T., McTeague, L. M., Hajcak, G., Halliday, C. A., & Danielson, C. K. (2025). Interpersonal violence moderates sustained-transient threat co-activation in the vmPFC and amygdala in a community sample of youth. *Development and Psychopathology*, *37*(4), 2151–2160. https://doi.org/10.1017/S0954579424001743

Boyes, A., Schwenn, P. E., Shan, Z. Y., Korgaonkar, M., Pang, J., Prince, T., Mills, L., Kennedy, M. F., & Hermens, D. F. (2024). *Geometric eigenmode brain fingerprinting and its longitudinal associations with adolescent mental health and wellbeing*. https://doi.org/10.1101/2024.08.08.607260

Bradley, K. A. L., Colcombe, S., Henderson, S. E., Alonso, C. M., Milham, M. P., & Gabbay, V. (2016). Neural correlates of self-perceptions in adolescents with major depressive disorder. *Developmental Cognitive Neuroscience*, *19*, 87–97. https://doi.org/10.1016/j.dcn.2016.02.007

Bray, K. O., Durbin, O., Hartanto, S., Khetan, M., Liontos, D., Manuele, S. J., Zwaan, I., Ganella, D., Herting, M. M., Kim, J. H., O’Connell, M., Pozzi, E., Schwartz, O., Seal, M., Simmons, J., Vijayakumar, N., & Whittle, S. (2024). Puberty and NeuroDevelopment in adolescents (PANDA): a study protocol. *BMC Pediatrics*, *24*(1), 768. https://doi.org/10.1186/s12887-024-05197-w

Brennan, C., Cini, E., Illingworth, S., Chapman, S., Simic, M., Nicholls, D., Chapman, V., Simms, C., Hayes, E., Fuller, S., Orpwood, J., Tweedy, N., Baksh, T., Astaire, E., Bhakta, D., Saglio, E., Stevenson, A., & Buchbinder, M. (2024). A prospective observational study comparing rates of medical instability between adolescents with typical and atypical anorexia nervosa. *Journal of Human Nutrition and Dietetics*, *37*(4), 1100–1108. https://doi.org/10.1111/jhn.13328

Brewe, A. M., Antezana, L., Carlton, C. N., Gracanin, D., Richey, J. A., Kim, I., & White, S. W. (2025). A Randomized Trial Utilizing EEG Brain Computer Interface to Improve Facial Emotion Recognition in Autistic Adults. *Journal of Autism and Developmental Disorders*, *55*(9), 3217–3230. https://doi.org/10.1007/s10803-024-06436-w

Broadhouse, K. M., Boyes, A., Winks, N., Dokonal, T., McLoughlin, L., Parker, M., Beaudequin, D., Simcock, G., Lagopoulos, J., & Hermens, D. F. (2019). Subcortical Volume Correlates of Psychological Distress in Early Adolescence. *Developmental Neuroscience*, *41*(3–4), 193–202. https://doi.org/10.1159/000502339

Brown, M. R. G., Benoit, J. R. A., Juhás, M., Dametto, E., Tse, T. T., MacKay, M., Sen, B., Carroll, A. M., Hodlevskyy, O., Silverstone, P. H., Dolcos, F., Dursun, S. M., & Greenshaw, A. J. (2015). fMRI investigation of response inhibition, emotion, impulsivity, and clinical high-risk behavior in adolescents. *Frontiers in Systems Neuroscience*, *9*. https://doi.org/10.3389/fnsys.2015.00124

Brown, S. A., Brumback, T., Tomlinson, K., Cummins, K., Thompson, W. K., Nagel, B. J., de Bellis, M. D., Hooper, S. R., Clark, D. B., Chung, T., Hasler, B. P., Colrain, I. M., Baker, F. C., Prouty, D., Pfefferbaum, A., Sullivan, E. v., Pohl, K. M., Rohlfing, T., Nichols, B. N., … Tapert, S. F. (2015). The National Consortium on Alcohol and NeuroDevelopment in Adolescence (NCANDA): A Multisite Study of Adolescent Development and Substance Use. *Journal of Studies on Alcohol and Drugs*, *76*(6), 895–908. https://doi.org/10.15288/jsad.2015.76.895

Bryn, V., Halvorsen, B., Ueland, T., Isaksen, J., Kolkova, K., Ravn, K., & Skjeldal, O. H. (2015). Brain derived neurotrophic factor (BDNF) and autism spectrum disorders (ASD) in childhood. *European Journal of Paediatric Neurology*, *19*(4), 411–414. https://doi.org/10.1016/J.EJPN.2015.03.005

Bui, H. T. P., Huy Do, D., Ly, H. T. T., Tran, K. T., Le, H. T. T., Nguyen, K. T., Pham, L. T. D., Le, H. D., Le, V. S., Mukhopadhyay, A., & Nguyen, L. T. (2024). De novo copy number variations in candidate genomic regions in patients of severe autism spectrum disorder in Vietnam. *PLOS ONE*, *19*(3), e0290936. https://doi.org/10.1371/journal.pone.0290936

Burkhouse, K. L., Jacobs, R. H., Peters, A. T., Ajilore, O., Watkins, E. R., & Langenecker, S. A. (2017). Neural correlates of rumination in adolescents with remitted major depressive disorder and healthy controls. *Cognitive, Affective, & Behavioral Neuroscience*, *17*(2), 394–405. https://doi.org/10.3758/s13415-016-0486-4

Burkhouse, K. L., Kujawa, A., Hosseini, B., Klumpp, H., Fitzgerald, K. D., Langenecker, S. A., Monk, C. S., & Phan, K. L. (2018). Anterior cingulate activation to implicit threat before and after treatment for pediatric anxiety disorders. *Progress in Neuro-Psychopharmacology and Biological Psychiatry*, *84*, 250–256. https://doi.org/10.1016/j.pnpbp.2018.03.013

Burkhouse, K. L., Kujawa, A., Keenan, K., Klumpp, H., Fitzgerald, K. D., Monk, C. S., & Phan, K. L. (2017). The relation between parent depressive symptoms and neural correlates of attentional control in offspring: A preliminary study. *Psychiatry Research: Neuroimaging*, *263*, 26–31. https://doi.org/10.1016/J.PSCYCHRESNS.2017.03.002

Burkhouse, K. L., Kujawa, A., Klumpp, H., Fitzgerald, K. D., Monk, C. S., & Phan, K. L. (2017). Neural correlates of explicit and implicit emotion processing in relation to treatment response in pediatric anxiety. *Journal of Child Psychology and Psychiatry*, *58*(5), 546–554. https://doi.org/10.1111/jcpp.12658

Burt, S. A., & Klump, K. L. (2013). The Michigan State University Twin Registry (MSUTR): An Update. *Twin Research and Human Genetics*, *16*(1), 344–350. https://doi.org/10.1017/thg.2012.87

Bussing, R., Grudnik, J., Mason, D., Wasiak, M., & Leonard, C. (2002). ADHD and Conduct Disorder: An MRI Study in a Community Sample. *The World Journal of Biological Psychiatry*, *3*(4), 216–220. https://doi.org/10.3109/15622970209150624

Butcher, T. J., Dzemidzic, M., Harezlak, J., Hulvershorn, L. A., & Oberlin, B. G. (2021). Brain responses during delay discounting in youth at high-risk for substance use disorders. *NeuroImage: Clinical*, *32*, 102772. https://doi.org/10.1016/j.nicl.2021.102772

Butera, C., Kaplan, J., Kilroy, E., Harrison, L., Jayashankar, A., Loureiro, F., & Aziz-Zadeh, L. (2023). The relationship between alexithymia, interoception, and neural functional connectivity during facial expression processing in autism spectrum disorder. *Neuropsychologia*, *180*, 108469. https://doi.org/10.1016/J.NEUROPSYCHOLOGIA.2023.108469

Byrne, M. L., Sheeber, L., Simmons, J. G., Davis, B., Shortt, J. W., Katz, L. F., & Allen, N. B. (2010). Autonomic cardiac control in depressed adolescents. *Depression and Anxiety*, *27*(11), 1050–1056. https://doi.org/10.1002/da.20717

Cahart, M.-S., Amad, A., Draper, S. B., Lowry, R. G., Marino, L., Carey, C., Ginestet, C. E., Smith, M. S., & Williams, S. C. R. (2022). The effect of learning to drum on behavior and brain function in autistic adolescents. *Proceedings of the National Academy of Sciences*, *119*(23). https://doi.org/10.1073/pnas.2106244119

Cahart, M.-S., Giampietro, V., & O’Daly, O. (2025). Atypical attentional network dynamics in adolescent depression during emotional movie viewing. *Social Cognitive and Affective Neuroscience*, *20*(1). https://doi.org/10.1093/scan/nsaf011

Cai, J., Xie, M., Liang, S., Gong, J., Deng, W., Guo, W., Ma, X., Sham, P. C., Wang, Q., & Li, T. (2024). Dysfunction of thalamocortical circuits in early-onset schizophrenia. *Cerebral Cortex*, *34*(8). https://doi.org/10.1093/cercor/bhae313

Cakar, M. E., Cummings, K. K., Bookheimer, S. Y., Dapretto, M., & Green, S. A. (2023). Age-related changes in neural responses to sensory stimulation in autism: a cross-sectional study. *Molecular Autism*, *14*(1), 38. https://doi.org/10.1186/s13229-023-00571-4

Calkins, M. E., Moore, T. M., Satterthwaite, T. D., Wolf, D. H., Turetsky, B. I., Roalf, D. R., Merikangas, K. R., Ruparel, K., Kohler, C. G., Gur, R. C., & Gur, R. E. (2017). Persistence of psychosis spectrum symptoms in the Philadelphia Neurodevelopmental Cohort: a prospective two‐year follow‐up. *World Psychiatry*, *16*(1), 62–76. https://doi.org/10.1002/wps.20386

CAMPBELL, M. J., SCHMIDT, L. A., SANTESSO, D. L., van AMERINGEN, M., MANCINI, C. L., & OAKMAN, J. M. (2007). Behavioral and psychophysiological characteristics of children of parents with social phobia: a pilot study. *International Journal of Neuroscience*, *117*(5), 605–616. https://doi.org/10.1080/00207450600773780

Cao, L., Feng, R., Gao, Y., Bao, W., Zhou, Z., Liang, K., Hu, X., Li, H., Zhang, L., Li, Y., Zhuo, L., Huang, G., & Huang, X. (2023). Suprachiasmatic nucleus functional connectivity related to insomnia symptoms in adolescents with major depressive disorder. *Frontiers in Psychiatry*, *14*. https://doi.org/10.3389/fpsyt.2023.1154095

Capitão, L. P., Chapman, R., Filippini, N., Wright, L., Murphy, S. E., James, A., Cowen, P. J., & Harmer, C. J. (2023). Acute neural effects of fluoxetine on emotional regulation in depressed adolescents. *Psychological Medicine*, *53*(10), 4799–4810. https://doi.org/10.1017/S0033291722001805

Cardinale, E. M., Bezek, J., Morales, S., Filippi, C., Smith, A. R., Haller, S., Valadez, E. A., Harrewijn, A., Phillips, D., Chronis-Tuscano, A., Brotman, M. A., Fox, N. A., Pine, D. S., Leibenluft, E., & Kircanski, K. (2023). Cross-sectional and Longitudinal Associations of Anxiety and Irritability With Adolescents’ Neural Responses to Cognitive Conflict. *Biological Psychiatry: Cognitive Neuroscience and Neuroimaging*, *8*(4), 436–444. https://doi.org/10.1016/j.bpsc.2022.03.007

Carper, R. A., Solders, S., Treiber, J. M., Fishman, I., & Müller, R.-A. (2015). Corticospinal Tract Anatomy and Functional Connectivity of Primary Motor Cortex in Autism. *Journal of the American Academy of Child & Adolescent Psychiatry*, *54*(10), 859–867. https://doi.org/10.1016/j.jaac.2015.07.007

Carrion, V. G., Garrett, A., Menon, V., Weems, C. F., & Reiss, A. L. (2008). Posttraumatic stress symptoms and brain function during a response-inhibition task: an fMRI study in youth. *Depression and Anxiety*, *25*(6), 514–526. https://doi.org/10.1002/da.20346

Carrion, V. G., Weems, C. F., & Reiss, A. L. (2007). Stress Predicts Brain Changes in Children: A Pilot Longitudinal Study on Youth Stress, Posttraumatic Stress Disorder, and the Hippocampus. *Pediatrics*, *119*(3), 509–516. https://doi.org/10.1542/peds.2006-2028

Carter, E. J., Williams, D. L., Minshew, N. J., & Lehman, J. F. (2012). Is He Being Bad? Social and Language Brain Networks during Social Judgment in Children with Autism. *PLoS ONE*, *7*(10), e47241. https://doi.org/10.1371/journal.pone.0047241

Carucci, S., Romaniello, R., Demuru, G., Curatolo, P., Grelloni, C., Masi, G., Liboni, F., Mereu, A., Contu, P., Lamberti, M., Gagliano, A., & Zuddas, A. (2022). Omega-3/6 supplementation for mild to moderate inattentive ADHD: a randomised, double-blind, placebo-controlled efficacy study in Italian children. *European Archives of Psychiatry and Clinical Neuroscience*, *272*(8), 1453–1467. https://doi.org/10.1007/s00406-022-01428-2

Cascio, C. N., Selkie, E., & Moreno, M. A. (2023). Effect of Technology and Digital Media Use on Adolescent Health and Development: Protocol for a Multimethod Longitudinal Study. *JMIR Research Protocols*, *12*, e50984. https://doi.org/10.2196/50984

Caswell, C. B., Hosseini-Kamkar, N., Cox, S. M., Prada, N. P., Iqbal, M., Nikolic, M., Banaschewski, T., Barker, G. J., Bokde, A. L. W., Brühl, R., Desrivières, S., Flor, H., Garavan, H., Gowland, P., Grigis, A., Heinz, A., Martinot, J.-L., Martinot, M.-L. P., Artiges, E., … Leyton, M. (2025). *A Novel Diathesis-Stress Model of Comorbid Early Onset Psychiatric Disorders*. https://doi.org/10.1101/2025.04.08.25325481

Cetin, F. H., Cetin, F., Isik, Y., Guney, E., Alp, F., & Aksoy, A. (2019). Attention deficit hyperactivity disorder and anti-Purkinje autoantibodies: no link? *Psychiatry and Clinical Psychopharmacology*, *29*(4), 435–440. https://doi.org/10.1080/24750573.2018.1517467

Cevher Binici, N., Inal Emiroglu, F. N., Resmi, H., & Ellidokuz, H. (2016). Serum Brain-derived Neurotrophic Factor Levels among Euthymic Adolescents with Bipolar Disorder Type I. *Noro Psikiyatri Arsivi*, *53*(3), 267–271. https://doi.org/10.5152/npa.2015.8832

Chai, R., Guo, J., Geng, Y., & Yao, X. (2025). Seasonal biological social and cognitive mechanisms of mental health among Chinese adolescents. *Scientific Reports*, *15*(1), 32642. https://doi.org/10.1038/s41598-025-18486-w

Chan, A. S., Sze, S. L., Siu, N. Y., Lau, E. M., & Cheung, M. (2013). A Chinese Mind-Body Exercise Improves Self-Control of Children with Autism: A Randomized Controlled Trial. *PLoS ONE*, *8*(7), e68184. https://doi.org/10.1371/journal.pone.0068184

Chang, K., DelBello, M., Garrett, A., Kelley, R., Howe, M., Adler, C., Welge, J., Strakowski, S. M., & Singh, M. (2018). Neurofunctional Correlates of Response to Quetiapine in Adolescents with Bipolar Depression. *Journal of Child and Adolescent Psychopharmacology*, *28*(6), 379–386. https://doi.org/10.1089/cap.2017.0030

Chatterjee, M., Saha, S., Sinha, S., & Mukhopadhyay, K. (2022). A three-pronged analysis confirms the association of the serotoninergic system with attention deficit hyperactivity disorder. *World Journal of Pediatrics*, *18*(12), 825–834. https://doi.org/10.1007/s12519-022-00614-5

Chattopadhyay, S., Tait, R., Simas, T., van Nieuwenhuizen, A., Hagan, C. C., Holt, R. J., Graham, J., Sahakian, B. J., Wilkinson, P. O., Goodyer, I. M., & Suckling, J. (2017). Cognitive Behavioral Therapy Lowers Elevated Functional Connectivity in Depressed Adolescents. *eBioMedicine*, *17*, 216–222. https://doi.org/10.1016/j.ebiom.2017.02.010

Cheetham, A., Allen, N. B., Whittle, S., Simmons, J. G., Yücel, M., & Lubman, D. I. (2012). Orbitofrontal Volumes in Early Adolescence Predict Initiation of Cannabis Use: A 4-Year Longitudinal and Prospective Study. *Biological Psychiatry*, *71*(8), 684–692. https://doi.org/10.1016/j.biopsych.2011.10.029

Cheetham, A., Allen, N. B., Whittle, S., Simmons, J., Yücel, M., & Lubman, D. I. (2014). Volumetric differences in the anterior cingulate cortex prospectively predict alcohol-related problems in adolescence. *Psychopharmacology*, *231*(8), 1731–1742. https://doi.org/10.1007/s00213-014-3483-8

Cheetham, A., Allen, N. B., Whittle, S., Simmons, J., Yücel, M., & Lubman, D. I. (2018). Amygdala volume mediates the relationship between externalizing symptoms and daily smoking in adolescence: A prospective study. *Psychiatry Research: Neuroimaging*, *276*, 46–52. https://doi.org/10.1016/j.pscychresns.2018.03.007

Chen, C., Wang, Z., Cao, X., & Zhu, J. (2023). Exploring the association between early exposure to material hardship and psychopathology through indirect effects of fronto-limbic functional connectivity during fear learning. *Cerebral Cortex*, *33*(20), 10702–10710. https://doi.org/10.1093/cercor/bhad320

Chen, J., Li, X., Zhang, Q., Zhou, Y., Wang, R., Tian, C., & Xiang, H. (2021). Impulsivity and Response Inhibition Related Brain Networks in Adolescents With Internet Gaming Disorder: A Preliminary Study Utilizing Resting-State fMRI. *Frontiers in Psychiatry*, *11*. https://doi.org/10.3389/fpsyt.2020.618319

Chen, J., Wei, Y., Xue, K., Gao, X., Zhang, M., Han, S., Wen, B., Wu, G., & Cheng, J. (2024). Static and temporal dynamic changes of intrinsic brain activity in early-onset and adult-onset schizophrenia: a fMRI study of interaction effects. *Frontiers in Neurology*, *15*. https://doi.org/10.3389/fneur.2024.1445599

Chen, J., Wei, Y., Xue, K., Han, S., Wang, C., Wen, B., & Cheng, J. (2023). The interaction between first-episode drug-naïve schizophrenia and age based on gray matter volume and its molecular analysis: a multimodal magnetic resonance imaging study. *Psychopharmacology*, *240*(4), 813–826. https://doi.org/10.1007/s00213-023-06323-9

Chen, M., & Cao, C. (2024). The mediation effect of glucocorticoid receptor gene ( *NR3C1* ) methylation between childhood maltreatment and depressive symptoms in Chinese adolescents: A 2-year longitudinal study. *Child Development*, *95*(1), 144–159. https://doi.org/10.1111/cdev.13972

Chen, M., Liu, D., Feng, J., & Tian, T. (2025). Insights into structural deviations in attention deficit hyperactivity disorder (ADHD) and comorbidities using big data-derived brain charts: a cross-sectional study. *Quantitative Imaging in Medicine and Surgery*, *15*(9), 8320–8332. https://doi.org/10.21037/qims-2024-2707

Chen, S., Lopez-Quintero, C., & Elton, A. (2026). Perceived Racism, Brain Development, and Internalizing and Externalizing Symptoms: Findings From the ABCD Study. *Journal of the American Academy of Child & Adolescent Psychiatry*, *65*(4), 552–563. https://doi.org/10.1016/j.jaac.2025.04.005

Chen, T., Jiang, J., Xu, M., Dai, Y., Gao, X., & Jiang, C. (2024). Atypical prefrontal neural activity during an emotional interference control task in adolescents with autism spectrum disorder: A functional near-infrared spectroscopy study. *NeuroImage*, *302*, 120907. https://doi.org/10.1016/j.neuroimage.2024.120907

Chen, X., Chen, H., Liu, J., Tang, H., Zhou, J., Liu, P., Tian, Y., Wang, X., Lu, F., & Zhou, J. (2023). Functional connectivity alterations in reward-related circuits associated with non-suicidal self-injury behaviors in drug-naïve adolescents with depression. *Journal of Psychiatric Research*, *163*, 270–277. https://doi.org/10.1016/j.jpsychires.2023.05.068

Chen, X., Zeng, C., Gong, C., Zhang, L., Wan, Y., Tao, F., & Sun, Y. (2019). Associations between early life parent-child separation and shortened telomere length and psychopathological outcomes during adolescence. *Psychoneuroendocrinology*, *103*, 195–202. https://doi.org/10.1016/J.PSYNEUEN.2019.01.021

Chen, Y., Xia, X., Zhou, Z., Yuan, M., Peng, Y., Liu, Y., Tang, J., & Fu, Y. (2024). Interleukin-6 is correlated with amygdala volume and depression severity in adolescents and young adults with first-episode major depressive disorder. *Brain Imaging and Behavior*, *18*(4), 773–782. https://doi.org/10.1007/s11682-024-00871-0

Cheng, Y., Chou, K.-H., Fan, Y.-T., & Lin, C.-P. (2011). ANS: Aberrant Neurodevelopment of the Social Cognition Network in Adolescents with Autism Spectrum Disorders. *PLoS ONE*, *6*(4), e18905. https://doi.org/10.1371/journal.pone.0018905

Cheung, C., McAlonan, G. M., Fung, Y. Y., Fung, G., Yu, K. K., Tai, K.-S., Sham, P. C., & Chua, S. E. (2011). MRI Study of Minor Physical Anomaly in Childhood Autism Implicates Aberrant Neurodevelopment in Infancy. *PLoS ONE*, *6*(6), e20246. https://doi.org/10.1371/journal.pone.0020246

Cheung, T., Ho, Y. S., Fong, K. H., Lam, Y. T. J., Li, M. H., Tse, A. C.-Y., Li, C.-T., Cheng, C. P.-W., & Beisteiner, R. (2022). Evaluating the Safety and Efficacy of Transcranial Pulse Stimulation on Autism Spectrum Disorder: A Double-Blinded, Randomized, Sham-Controlled Trial Protocol. *International Journal of Environmental Research and Public Health*, *19*(23), 15614. https://doi.org/10.3390/ijerph192315614

Chi, S., Mok, Y. E., Lee, J., Suh, S., Han, C., & Lee, M.-S. (2023). Functional connectivity and network analysis in adolescents with major depressive disorder showing suicidal behavior. *Journal of Affective Disorders*, *343*, 42–49. https://doi.org/10.1016/j.jad.2023.09.027

Chiang, J. J., Park, H., Almeida, D. M., Bower, J. E., Cole, S. W., Irwin, M. R., McCreath, H., Seeman, T. E., & Fuligni, A. J. (2019). Psychosocial stress and C-reactive protein from mid-adolescence to young adulthood. *Health Psychology*, *38*(3), 259–267. https://doi.org/10.1037/hea0000701

Chien, H., Gau, S. S., Hsu, Y., Chen, Y., Lo, Y., Shih, Y., & Tseng, W. I. (2015). Altered Cortical Thickness and Tract Integrity of the Mirror Neuron System and Associated Social Communication in Autism Spectrum Disorder. *Autism Research*, *8*(6), 694–708. https://doi.org/10.1002/aur.1484

Chin Fatt, C., Ayvaci, E. R., Jha, M. K., Emslie, G., Gibson, S., Minhajuddin, A. T., Mayes, T. L., Farrar, J. D., & Trivedi, M. H. (2023). Characterizing inflammatory profiles of suicidal behavior in adolescents: Rationale and design. *Journal of Affective Disorders*, *325*, 55–61. https://doi.org/10.1016/J.JAD.2022.12.114

Chin Fatt, C. R., Ballard, E. D., Minhajuddin, A. T., Toll, R., Mayes, T. L., Foster, J. A., & Trivedi, M. H. (2024). Active suicidal ideation associated with dysfunction in default mode network using resting-state EEG and functional MRI - Findings from the T-RAD Study. *Journal of Psychiatric Research*, *176*, 240–247. https://doi.org/10.1016/J.JPSYCHIRES.2024.06.016

Chin Fatt, C. R., Jha, M. K., Minhajuddin, A., Mayes, T., Ballard, E. D., & Trivedi, M. H. (2021). Dysfunction of default mode network is associated with active suicidal ideation in youths and young adults with depression: Findings from the T-RAD study. *Journal of Psychiatric Research*, *142*, 258–262. https://doi.org/10.1016/j.jpsychires.2021.07.047

Chin, W.-C., Huang, S.-Y., Liu, F.-Y., Wang, C.-H., Tang, I., Hsiao, I.-T., & Huang, Y.-S. (2024). The application of machine learning on brain imaging features of different narcolepsy subtypes. *SLEEP*, *47*(2). https://doi.org/10.1093/sleep/zsad328

Chiu, S., Widjaja, F., Bates, M. E., Voelbel, G. T., Pandina, G., Marble, J., Blank, J. A., Day, J., Brule, N., & Hendren, R. L. (2008). Anterior cingulate volume in pediatric bipolar disorder and autism. *Journal of Affective Disorders*, *105*(1–3), 93–99. https://doi.org/10.1016/J.JAD.2007.04.019

Chopra, P., Sagar, R., & Mukhopadhyay, A. K. (2021). Oxidative Stress Markers (8-Isoprostane and 8-Hydroxy-2-Deoxyguanosine) in Major Depression: A Case-control Study. *JOURNAL OF CLINICAL AND DIAGNOSTIC RESEARCH*. https://doi.org/10.7860/JCDR/2021/47766.14982

Chopra, S., Francey, S. M., O’Donoghue, B., Sabaroedin, K., Arnatkeviciute, A., Cropley, V., Nelson, B., Graham, J., Baldwin, L., Tahtalian, S., Yuen, H. P., Allott, K., Alvarez-Jimenez, M., Harrigan, S., Pantelis, C., Wood, S. J., McGorry, P., & Fornito, A. (2021). Functional Connectivity in Antipsychotic-Treated and Antipsychotic-Naive Patients With First-Episode Psychosis and Low Risk of Self-harm or Aggression. *JAMA Psychiatry*, *78*(9), 994. https://doi.org/10.1001/jamapsychiatry.2021.1422

Chu, K.-C., Lu, H.-K., Huang, M.-C., Lin, S.-J., Liu, W.-I., Huang, Y.-S., Hsu, J.-F., & Wang, C.-H. (2020). Using Mobile Electroencephalography and Actigraphy to Diagnose Attention-Deficit/Hyperactivity Disorder: Case-Control Comparison Study. *JMIR Mental Health*, *7*(6), e12158. https://doi.org/10.2196/12158

Chu, S.-H., Lenglet, C., Schreiner, M. W., Klimes-Dougan, B., Cullen, K., & Parhi, K. K. (2018a). Anatomical Biomarkers for Adolescent Major Depressive Disorder from Diffusion Weighted Imaging using SVM Classifier. *2018 40th Annual International Conference of the IEEE Engineering in Medicine and Biology Society (EMBC)*, 2740–2743. https://doi.org/10.1109/EMBC.2018.8512852

Chu, S.-H., Lenglet, C., Schreiner, M. W., Klimes-Dougan, B., Cullen, K., & Parhi, K. K. (2018b). Biomarkers for Adolescent MDD from Anatomical Connectivity and Network Topology Using Diffusion MRI. *2018 40th Annual International Conference of the IEEE Engineering in Medicine and Biology Society (EMBC)*, 1152–1155. https://doi.org/10.1109/EMBC.2018.8512505

Chuang, J.-Y., J Whitaker, K., Murray, G. K., Elliott, R., Hagan, C. C., Graham, J. M., Ooi, C., Tait, R., Holt, R. J., van Nieuwenhuizen, A. O., Reynolds, S., Wilkinson, P. O., Bullmore, E. T., Lennox, B. R., Sahakian, B. J., Goodyer, I., & Suckling, J. (2016). Aberrant brain responses to emotionally valent words is normalised after cognitive behavioural therapy in female depressed adolescents. *Journal of Affective Disorders*, *189*, 54–61. https://doi.org/10.1016/j.jad.2015.09.008

Chumpitazi, B. P., Weidler, E. M., & Shulman, R. J. (2017). Lactulose Breath Test Gas Production in Childhood IBS Is Associated With Intestinal Transit and Bowel Movement Frequency. *Journal of Pediatric Gastroenterology and Nutrition*, *64*(4), 541–545. https://doi.org/10.1097/MPG.0000000000001295

Chung, S. A., Hwang, H., Kim, H. J., Hong, J. S., Kim, S. M., & Han, D. H. (2025). Changes in heart rate variability and hemodynamics of adolescents within the frontal cortex in response to face emotional stimulation. *PLOS One*, *20*(7), e0326204. https://doi.org/10.1371/journal.pone.0326204

Chung, T., & Clark, D. B. (2014). Insula White Matter Volume Linked to Binge Drinking Frequency Through Enhancement Motives in Treated Adolescents. *Alcoholism: Clinical and Experimental Research*, *38*(7), 1932–1940. https://doi.org/10.1111/acer.12461

Chung, Y., Allswede, D., Addington, J., Bearden, C. E., Cadenhead, K., Cornblatt, B., Mathalon, D. H., McGlashan, T., Perkins, D., Seidman, L. J., Tsuang, M., Walker, E., Woods, S. W., McEwen, S., van Erp, T. G. M., & Cannon, T. D. (2019). Cortical abnormalities in youth at clinical high-risk for psychosis: Findings from the NAPLS2 cohort. *NeuroImage: Clinical*, *23*, 101862. https://doi.org/10.1016/j.nicl.2019.101862

Cisler, J. M., Sigel, B. A., Kramer, T. L., Smitherman, S., Vanderzee, K., Pemberton, J., & Kilts, C. D. (2015). Amygdala response predicts trajectory of symptom reduction during Trauma-Focused Cognitive-Behavioral Therapy among adolescent girls with PTSD. *Journal of Psychiatric Research*, *71*, 33–40. https://doi.org/10.1016/j.jpsychires.2015.09.011

Cohen, J. W., Ramphal, B., DeSerisy, M., Zhao, Y., Pagliaccio, D., Colcombe, S., Milham, M. P., & Margolis, A. E. (2022). *Relative Brain Age Is Associated with Socioeconomic Status and Anxiety/Depression Problems in Youth*. https://doi.org/10.1101/2022.09.15.505331

Cordola Hsu, A. R., Nguyen-Rodriguez, S., & Spruijt-Metz, D. (2019). Age, Physical Activity Motivation and Perceived Stress in Minority Girls. *Californian Journal of Health Promotion*, *17*(2), 01–12. https://doi.org/10.32398/cjhp.v17i2.2285

Cordova, M., Shada, K., Demeter, D. v, Doyle, O., Miranda-Dominguez, O., Perrone, A., Schifsky, E., Graham, A., Fombonne, E., Langhorst, B., Nigg, J., Fair, D. A., & Feczko, E. (2020). Heterogeneity of executive function revealed by a functional random forest approach across ADHD and ASD. *NeuroImage: Clinical*, *26*, 102245. https://doi.org/10.1016/j.nicl.2020.102245

Corrivetti, G., Monaco, F., Vignapiano, A., Marenna, A., Palm, K., Fernández-Arroyo, S., Frigola-Capell, E., Leen, V., Ibarrola, O., Amil, B., Caruson, M. M., Chiariotti, L., Palacios-Ariza, M. A., Hoekstra, P. J., Chiang, H.-Y., Floareș, A., Fagiolini, A., & Fasano, A. (2024). Optimizing and Predicting Antidepressant Efficacy in Patients with Major Depressive Disorder Using Multi-Omics Analysis and the Opade AI Prediction Tools. *Brain Sciences*, *14*(7), 658. https://doi.org/10.3390/brainsci14070658

Couvy-Duchesne, B., Strike, L. T., de Zubicaray, G. I., McMahon, K. L., Thompson, P. M., Hickie, I. B., Martin, N. G., & Wright, M. J. (2018). Lingual Gyrus Surface Area Is Associated with Anxiety-Depression Severity in Young Adults: A Genetic Clustering Approach. *Eneuro*, *5*(1), ENEURO.0153-17.2017. https://doi.org/10.1523/ENEURO.0153-17.2017

Croarkin, P. E., Elmaadawi, A. Z., Aaronson, S. T., Schrodt, G. R., Holbert, R. C., Verdoliva, S., Heart, K. L., Demitrack, M. A., & Strawn, J. R. (2021). Left prefrontal transcranial magnetic stimulation for treatment-resistant depression in adolescents: a double-blind, randomized, sham-controlled trial. *Neuropsychopharmacology*, *46*(2), 462–469. https://doi.org/10.1038/s41386-020-00829-y

Crone, E. A., Bol, T., Braams, B. R., de Rooij, M., Franke, B., Franken, I., Gazzola, V., Güroğlu, B., Huizenga, H., Hulshoff Pol, H., Keijsers, L., Keysers, C., Krabbendam, L., Jansen, L., Popma, A., Stulp, G., van Atteveldt, N., van Duijvenvoorde, A., & Veenstra, R. (2024). Growing Up Together in Society (GUTS): A team science effort to predict societal trajectories in adolescence and young adulthood. *Developmental Cognitive Neuroscience*, *67*, 101403. https://doi.org/10.1016/J.DCN.2024.101403

Crosby, R., Salazar, L. F., DiClemente, R. J., Yarber, W. L., Caliendo, A. M., & Staples-Horne, M. (2004). Health risk factors among detained adolescent females. *American Journal of Preventive Medicine*, *27*(5), 404–410. https://doi.org/10.1016/J.AMEPRE.2004.07.017

Crum, K. I., Aloi, J., Blair, K. S., Bashford-Largo, J., Bajaj, S., Zhang, R., Hwang, S., Schwartz, A., Elowsky, J., Filbey, F. M., Dobbertin, M., & Blair, R. J. (2023). Latent profiles of substance use, early life stress, and attention/externalizing problems and their association with neural correlates of reinforcement learning in adolescents. *Psychological Medicine*, *53*(15), 7358–7367. https://doi.org/10.1017/S0033291723000971

Cucchiara, F., Frumento, P., Banfi, T., Sesso, G., di Galante, M., D’Ascanio, P., Valvo, G., Sicca, F., & Faraguna, U. (2020). Electrophysiological features of sleep in children with Kir4.1 channel mutations and Autism–Epilepsy phenotype: a preliminary study. *Sleep*, *43*(4). https://doi.org/10.1093/sleep/zsz255

Cullen, A. E., Fisher, H. L., Gullet, N., Fraser, E. R., Roberts, R. E., Zahid, U., To, M., Yap, N. H., Zunszain, P. A., Pariante, C. M., Wood, S. J., McGuire, P., Murray, R. M., Mondelli, V., & Laurens, K. R. (2022). Cortisol Levels in Childhood Associated With Emergence of Attenuated Psychotic Symptoms in Early Adulthood. *Biological Psychiatry*, *91*(2), 226–235. https://doi.org/10.1016/j.biopsych.2021.08.009

Cullen, A. E., Tappin, B. M., Zunszain, P. A., Dickson, H., Roberts, R. E., Nikkheslat, N., Khondoker, M., Pariante, C. M., Fisher, H. L., & Laurens, K. R. (2017). The relationship between salivary C-reactive protein and cognitive function in children aged 11–14 years: Does psychopathology have a moderating effect? *Brain, Behavior, and Immunity*, *66*, 221–229. https://doi.org/10.1016/j.bbi.2017.07.002

Cullen, K. R., Schreiner, M. W., Klimes-Dougan, B., Eberly, L. E., LaRiviere, L. L., Lim, K. O., Camchong, J., & Mueller, B. A. (2020). Neural correlates of clinical improvement in response to N-acetylcysteine in adolescents with non-suicidal self-injury. *Progress in Neuro-Psychopharmacology and Biological Psychiatry*, *99*, 109778. https://doi.org/10.1016/j.pnpbp.2019.109778

Cullen, K. R., Westlund, M. K., Klimes-Dougan, B., Mueller, B. A., Houri, A., Eberly, L. E., & Lim, K. O. (2014). Abnormal Amygdala Resting-State Functional Connectivity in Adolescent Depression. *JAMA Psychiatry*, *71*(10), 1138. https://doi.org/10.1001/jamapsychiatry.2014.1087

Cunha, G. R., Asevedo, E., Mansur, R. B., Zugman, A., Pan, P. M., Gadelha, A., Belangero, S. I., Rizzo, L. B., Coelho, R., Stertz, L., Cogo‐Moreira, H., Grassi‐Oliveira, R., Teixeira, A. L., Kauer‐Sant’Anna, M., Mari, J. J., Miguel, E. C., Bressan, R. A., & Brietzke, E. (2016). Inflammation, neurotrophism and oxidative stress and childhood psychopathology in a large community sample. *Acta Psychiatrica Scandinavica*, *133*(2), 122–132. https://doi.org/10.1111/acps.12453

Curzio, O., Billeci, L., Belmonti, V., Colantonio, S., Cotrozzi, L., de Pasquale, C. F., Morales, M. A., Nali, C., Pascali, M. A., Venturi, F., Tonacci, A., Zannoni, N., & Maestro, S. (2022). Horticultural Therapy May Reduce Psychological and Physiological Stress in Adolescents with Anorexia Nervosa: A Pilot Study. *Nutrients*, *14*(24), 5198. https://doi.org/10.3390/nu14245198

Curzio, O., Calderoni, S., Maestro, S., Rossi, G., de Pasquale, C. F., Belmonti, V., Apicella, F., Muratori, F., & Retico, A. (2020). Lower gray matter volumes of frontal lobes and insula in adolescents with anorexia nervosa restricting type: Findings from a Brain Morphometry Study. *European Psychiatry*, *63*(1), e27. https://doi.org/10.1192/j.eurpsy.2020.19

Dai, J., Lin, X.-T., Shen, L.-L., Zhang, X.-W., Ding, Z.-W., Wang, J., Fan, X.-W., & Ning, W.-D. (2025). Immune indicators and depression in adolescents: Associations with monocytes, lymphocytes, and direct bilirubin. *World Journal of Psychiatry*, *15*(2). https://doi.org/10.5498/wjp.v15.i2.101818

Dal Canto, G., Pellacani, S., Valvo, G., Masi, G., Ferrari, A. R., & Sicca, F. (2018). Internalizing and externalizing symptoms in preschool and school-aged children with epilepsy: Focus on clinical and EEG features. *Epilepsy & Behavior*, *79*, 68–74. https://doi.org/10.1016/j.yebeh.2017.10.004

Dalwani, M., Sakai, J. T., Mikulich-Gilbertson, S. K., Tanabe, J., Raymond, K., McWilliams, S. K., Thompson, L. L., Banich, M. T., & Crowley, T. J. (2011). Reduced cortical gray matter volume in male adolescents with substance and conduct problems. *Drug and Alcohol Dependence*, *118*(2–3), 295–305. https://doi.org/10.1016/j.drugalcdep.2011.04.006

D’Angiulli, A., Weinberg, J., Oberlander, T. F., Grunau, R. E., Hertzman, C., & Maggi, S. (2012). Frontal EEG/ERP correlates of attentional processes, cortisol and motivational states in adolescents from lower and higher socioeconomic status. *Frontiers in Human Neuroscience*, *6*. https://doi.org/10.3389/fnhum.2012.00306

Danielsen, Y. S., Årdal Rekkedal, G., Frostad, S., & Kessler, U. (2016). Effectiveness of enhanced cognitive behavioral therapy (CBT-E) in the treatment of anorexia nervosa: a prospective multidisciplinary study. *BMC Psychiatry*, *16*(1), 342. https://doi.org/10.1186/s12888-016-1056-6

DASTAMOOZ, S., HO, R. T. H., ARBOUR-NICITOPOULOS, K., WONG, S. H. S., & SIT, C. H. P. (2025). The Association between Movement Behaviors and Mental Health Issues in Adolescents with Neurodevelopmental Disorders. *Medicine & Science in Sports & Exercise*, *57*(5), 932–941. https://doi.org/10.1249/MSS.0000000000003628

de Bellis, M. D., & Keshavan, M. S. (2003). Sex differences in brain maturation in maltreatment-related pediatric posttraumatic stress disorder. *Neuroscience & Biobehavioral Reviews*, *27*(1–2), 103–117. https://doi.org/10.1016/S0149-7634(03)00013-7

de Bellis, M. D., van Voorhees, E., Hooper, S. R., Gibler, N., Nelson, L., Hege, S. G., Payne, M. E., & MacFall, J. (2008). Diffusion Tensor Measures of the Corpus Callosum in Adolescents With Adolescent Onset Alcohol Use Disorders. *Alcoholism: Clinical and Experimental Research*, *32*(3), 395–404. https://doi.org/10.1111/j.1530-0277.2007.00603.x

de Brito, S. A., Viding, E., Sebastian, C. L., Kelly, P. A., Mechelli, A., Maris, H., & McCrory, E. J. (2013). Reduced orbitofrontal and temporal grey matter in a community sample of maltreated children. *Journal of Child Psychology and Psychiatry*, *54*(1), 105–112. https://doi.org/10.1111/j.1469-7610.2012.02597.x

de la Serna, E., Montejo, L., Solé, B., Castro-Fornieles, J., Camprodon-Boadas, P., Sugranyes, G., Rosa-Justicia, M., Martinez-Aran, A., Vieta, E., Vicent-Gil, M., Serra-Blasco, M., Cardoner, N., & Torrent, C. (2023). Effectiveness of enhancing cognitive reserve in children, adolescents and young adults at genetic risk for psychosis: Study protocol for a randomized controlled trial. *Spanish Journal of Psychiatry and Mental Health*, *16*(3), 184–191. https://doi.org/10.1016/j.rpsm.2021.02.003

de Pasquale, C., Pistorio, M. L., Tornatore, E., de Berardis, D., & Fornaro, M. (2013). The relationship between drive to thinness, conscientiousness and bulimic traits during adolescence: a comparison between younger and older cases in 608 healthy volunteers. *Annals of General Psychiatry*, *12*(1), 34. https://doi.org/10.1186/1744-859X-12-34

de Wit, S., Ziermans, T. B., Nieuwenhuis, M., Schothorst, P. F., van Engeland, H., Kahn, R. S., Durston, S., & Schnack, H. G. (2017). Individual prediction of long-term outcome in adolescents at ultra-high risk for psychosis: Applying machine learning techniques to brain imaging data. *Human Brain Mapping*, *38*(2), 704–714. https://doi.org/10.1002/hbm.23410

Demirlek, C., Verim, B., Zorlu, N., Demir, M., Yalincetin, B., Eyuboglu, M. S., Cesim, E., Uzman-Özbek, S., Süt, E., Öngür, D., & Bora, E. (2024). Functional brain networks in clinical high-risk for bipolar disorder and psychosis. *Psychiatry Research*, *342*, 116251. https://doi.org/10.1016/j.psychres.2024.116251

Deng, W.-X., Liu, X.-B., Guo, T., Shang, L.-F., Li, Y., Zeng, K., & Long, J.-Y. (2025). Metabolomic Changes in Major Depressive Disorder Adolescent Females with or without Suicide Attempts. *Current Neuropharmacology*, *23*(7), 787–799. https://doi.org/10.2174/1570159X23666250122093451

Dennis, E. L., Gotlib, I. H., Thompson, P. M., & Thomason, M. E. (2011). Anxiety Modulates Insula Recruitment in Resting-State Functional Magnetic Resonance Imaging in Youth and Adults. *Brain Connectivity*, *1*(3), 245–254. https://doi.org/10.1089/brain.2011.0030

Derome, M., Fonseca-Pedrero, E., Badoud, D., Morosan, L., van de Ville, D., Lazeyras, F., Eliez, S., Chan, R., Rudrauf, D., Schwartz, S., & Debbane, M. (2018). Resting-State Networks of Adolescents Experiencing Depersonalization-Like Illusions: Cross-sectional and Longitudinal Findings. *Schizophrenia Bulletin*, *44*(suppl_2), S501–S511. https://doi.org/10.1093/schbul/sby031

Deveney, C. M., Connolly, M. E., Jenkins, S. E., Kim, P., Fromm, S. J., Pine, D. S., & Leibenluft, E. (2012). Neural recruitment during failed motor inhibition differentiates youths with bipolar disorder and severe mood dysregulation. *Biological Psychology*, *89*(1), 148–155. https://doi.org/10.1016/j.biopsycho.2011.10.003

Dhawan, A., Baitamouni, S., Liu, D., Busch, R., Klaas, P., Frazier, T. W., Srivastava, S., Parikh, S., Hsich, G. E., Friedman, N. R., Ritter, D. M., Hardan, A. Y., Martinez‐Agosto, J. A., Sahin, M., & Eng, C. (2024). Exploring the neurological features of individuals with germline <scp>PTEN</scp> variants: A multicenter study. *Annals of Clinical and Translational Neurology*, *11*(5), 1301–1309. https://doi.org/10.1002/acn3.52046

di Martino, A., Yan, C.-G., Li, Q., Denio, E., Castellanos, F. X., Alaerts, K., Anderson, J. S., Assaf, M., Bookheimer, S. Y., Dapretto, M., Deen, B., Delmonte, S., Dinstein, I., Ertl-Wagner, B., Fair, D. A., Gallagher, L., Kennedy, D. P., Keown, C. L., Keysers, C., … Milham, M. P. (2014). The autism brain imaging data exchange: towards a large-scale evaluation of the intrinsic brain architecture in autism. *Molecular Psychiatry*, *19*(6), 659–667. https://doi.org/10.1038/mp.2013.78

Díaz, D. E., Tseng, W.-L., & Michalska, K. J. (2024). Pre-scan state anxiety is associated with greater right amygdala-hippocampal response to fearful versus happy faces among trait-anxious Latina girls. *BMC Psychiatry*, *24*(1), 1. https://doi.org/10.1186/s12888-023-05403-6

Dickstein, D. P., Finger, E. C., Skup, M., Pine, D. S., Blair, J. R., & Leibenluft, E. (2010). Altered neural function in pediatric bipolar disorder during reversal learning. *Bipolar Disorders*, *12*(7), 707–719. https://doi.org/10.1111/j.1399-5618.2010.00863.x

Dietz, L. J., & Matthews, K. A. (2011). Depressive Symptoms and Subclinical Markers of Cardiovascular Disease in Adolescents. *Journal of Adolescent Health*, *48*(6), 579–584. https://doi.org/10.1016/J.JADOHEALTH.2010.09.001

Diler, R. S., de Almeida, J. R. C., Ladouceur, C., Birmaher, B., Axelson, D., & Phillips, M. (2013). Neural activity to intense positive versus negative stimuli can help differentiate bipolar disorder from unipolar major depressive disorder in depressed adolescents: A pilot fMRI study. *Psychiatry Research: Neuroimaging*, *214*(3), 277–284. https://doi.org/10.1016/j.pscychresns.2013.06.013

Diler, R. S., Pan, L. A., Segreti, A., Ladouceur, C. D., Forbes, E., Cela, S. R., Almeida, J. R. C., Birmaher, B., Axelson, D. A., & Phillips, M. L. (2014). Differential Anterior Cingulate Activity during Response Inhibition in Depressed Adolescents with Bipolar and Unipolar Major Depressive Disorder. *Journal of the Canadian Academy of Child and Adolescent Psychiatry = Journal de l’Academie Canadienne de Psychiatrie de l’enfant et de l’adolescent*, *23*(1), 10–19.

Dimick, M. K., Toma, S., MacIntosh, B. J., Grigorian, A., Fiksenbaum, L., Youngstrom, E. A., Robertson, A. D., & Goldstein, B. I. (2022). Cerebral Blood Flow and Core Mood Symptoms in Youth Bipolar Disorder: Evidence for Region–Symptom Specificity. *Journal of the American Academy of Child & Adolescent Psychiatry*, *61*(12), 1455–1465. https://doi.org/10.1016/J.JAAC.2022.04.010

Diwadkar, V. A. (2012). Disordered Corticolimbic Interactions During Affective Processing in Children and Adolescents at Risk for Schizophrenia Revealed by Functional Magnetic Resonance Imaging and Dynamic Causal Modeling. *Archives of General Psychiatry*, *69*(3), 231. https://doi.org/10.1001/archgenpsychiatry.2011.1349

do Prado, C. H., Grassi-Oliveira, R., Wieck, A., Zaparte, A., Filho, L. D., da Silva Morrone, M., Moreira, J. C., & Bauer, M. E. (2016). The impact of childhood maltreatment on redox state: Relationship with oxidative damage and antioxidant defenses in adolescents with no psychiatric disorder. *Neuroscience Letters*, *617*, 173–177. https://doi.org/10.1016/j.neulet.2016.01.062

Dobbertin, M., Blair, K. S., Aloi, J., Bajaj, S., Bashford-Largo, J., Mathur, A., Zhang, R., Carollo, E., Schwartz, A., Elowsky, J., Ringle, J. L., Tyler, P., & Blair, R. J. (2024). Neural correlates of automatic emotion regulation and their association with suicidal ideation in adolescents during the first 90-days of residential care. *Translational Psychiatry*, *14*(1), 54. https://doi.org/10.1038/s41398-023-02723-9

Doruk Camsari, D., Lewis, C. P., Sonmez, A. I., Ozger, C., Fatih, P., Yuruk, D., Shekunov, J., vande Voort, J. L., & Croarkin, P. E. (2023). Event-Related Potential Markers of Suicidality in Adolescents. *International Journal of Neuropsychopharmacology*, *26*(8), 566–575. https://doi.org/10.1093/ijnp/pyad039

dos Santos, P. M. N., Mendes, S. L., Biazoli, C., Gadelha, A., Salum, G. A., Miguel, E. C., Rohde, L. A., & Sato, J. R. (2023). Assessing atypical brain functional connectivity development: An approach based on generative adversarial networks. *Frontiers in Neuroscience*, *16*. https://doi.org/10.3389/fnins.2022.1025492

Doulalas, A. D., Rallidis, L. S., Gialernios, T., Moschonas, D. N., Kougioulis, M. N., Rizos, I., Tselegaridis, T. S., & Kremastinos, D. T. (2006). Association of depressive symptoms with coagulation factors in young healthy individuals. *Atherosclerosis*, *186*(1), 121–125. https://doi.org/10.1016/j.atherosclerosis.2005.06.030

Duffy, F. H., Shankardass, A., McAnulty, G. B., & Als, H. (2017). A unique pattern of cortical connectivity characterizes patients with attention deficit disorders: a large electroencephalographic coherence study. *BMC Medicine*, *15*(1), 51. https://doi.org/10.1186/s12916-017-0805-9

Dziembowska, I., Izdebski, P., Rasmus, A., Brudny, J., Grzelczak, M., & Cysewski, P. (2016). Effects of Heart Rate Variability Biofeedback on EEG Alpha Asymmetry and Anxiety Symptoms in Male Athletes: A Pilot Study. *Applied Psychophysiology and Biofeedback*, *41*(2), 141–150. https://doi.org/10.1007/s10484-015-9319-4

Edgar, E. v., Waugh, A., Wu, J., Castagna, P., Potenza, M. N., Mayes, L. C., & Crowley, M. J. (2024). Risk avoidance and social anxiety in adolescence: Examination of event-related potentials and theta-dynamics on the Balloon Risk Avoidance Task. *Brain and Cognition*, *180*, 106209. https://doi.org/10.1016/j.bandc.2024.106209

Edmiston, E. E. (2011). Corticostriatal-Limbic Gray Matter Morphology in Adolescents With Self-reported Exposure to Childhood Maltreatment. *Archives of Pediatrics & Adolescent Medicine*, *165*(12), 1069. https://doi.org/10.1001/archpediatrics.2011.565

Ehrler, M., O’Gorman, R., Wehrle, F. M., Speckert, A., Jakab, A., Kretschmar, O., & Latal, B. (2025). Learning from those who thrive: protective factors and neuroimaging markers in adolescents with complex congenital heart disease and with a favorable neurodevelopmental profile. *Child Neuropsychology*, *31*(4), 613–634. https://doi.org/10.1080/09297049.2024.2419048

Eigsti, I.-M., Schuh, J., Mencl, E., Schultz, R. T., & Paul, R. (2012). The neural underpinnings of prosody in autism. *Child Neuropsychology*, *18*(6), 600–617. https://doi.org/10.1080/09297049.2011.639757

Eigsti, I.-M., Stevens, M. C., Schultz, R. T., Barton, M., Kelley, E., Naigles, L., Orinstein, A., Troyb, E., & Fein, D. A. (2016). Language comprehension and brain function in individuals with an optimal outcome from autism. *NeuroImage: Clinical*, *10*, 182–191. https://doi.org/10.1016/j.nicl.2015.11.014

el Fotoh, W. M. M. A., el Naby, S. A. A., & Abd El Hady, N. M. S. (2019). Autism Spectrum Disorders: The Association with Inherited Metabolic Disorders and Some Trace Elements. A Retrospective Study. *CNS & Neurological Disorders - Drug Targets*, *18*(5), 413–420. https://doi.org/10.2174/1871527318666190430162724

Elalfy, M. S., Ibrahim, A. S., Ibrahim, G. S., Hussein, H. M. A. G., Mohammed, H. G. E., & Ebeid, F. S. E. (2021). Hidden brain iron content in sickle cell disease: impact on neurocognitive functions. *European Journal of Pediatrics*, *180*(8), 2677–2686. https://doi.org/10.1007/s00431-021-04189-7

El-Ansary, A., Alhakbany, M., Aldbass, A., Qasem, H., Al-Mazidi, S., Bhat, R. S., & Al-Ayadhi, L. (2021). Alpha-Synuclein, cyclooxygenase-2 and prostaglandins-EP2 receptors as neuroinflammatory biomarkers of autism spectrum disorders: Use of combined ROC curves to increase their diagnostic values. *Lipids in Health and Disease*, *20*(1), 155. https://doi.org/10.1186/s12944-021-01578-7

El-Ansary, A., Bjørklund, G., Chirumbolo, S., & Alnakhli, O. M. (2017). Predictive value of selected biomarkers related to metabolism and oxidative stress in children with autism spectrum disorder. *Metabolic Brain Disease*, *32*(4), 1209–1221. https://doi.org/10.1007/s11011-017-0029-x

Ellis, A., Salgari, G., Miklowitz, D. J., & Loo, S. K. (2016). 2.18 SEMANTIC PROCESSING OF EMOTIONAL WORDS IN ADOLESCENTS WITH MOOD DISORDERS: AN EVENT-RELATED POTENTIAL STUDY. *Journal of the American Academy of Child & Adolescent Psychiatry*, *55*(10), S126. https://doi.org/10.1016/j.jaac.2016.09.084

Ellis, R., Seal, M. L., Adamson, C., Beare, R., Simmons, J. G., Whittle, S., & Allen, N. B. (2017). Brain connectivity networks and longitudinal trajectories of depression symptoms in adolescence. *Psychiatry Research: Neuroimaging*, *260*, 62–69. https://doi.org/10.1016/J.PSCYCHRESNS.2016.12.010

Elsayed, N. M., Kim, M. J., Fields, K. M., Olvera, R. L., Hariri, A. R., & Williamson, D. E. (2018). Trajectories of Alcohol Initiation and Use During Adolescence: The Role of Stress and Amygdala Reactivity. *Journal of the American Academy of Child & Adolescent Psychiatry*, *57*(8), 550–560. https://doi.org/10.1016/j.jaac.2018.05.011

Emond, P., Mavel, S., Aïdoud, N., Nadal-Desbarats, L., Montigny, F., Bonnet-Brilhault, F., Barthélémy, C., Merten, M., Sarda, P., Laumonnier, F., Vourc’h, P., Blasco, H., & Andres, C. R. (2013). GC-MS-based urine metabolic profiling of autism spectrum disorders. *Analytical and Bioanalytical Chemistry*, *405*(15), 5291–5300. https://doi.org/10.1007/s00216-013-6934-x

English, J. A., Lopez, L. M., O’Gorman, A., Föcking, M., Hryniewiecka, M., Scaife, C., Sabherwal, S., Wynne, K., Dicker, P., Rutten, B. P. F., Lewis, G., Zammit, S., Cannon, M., Cagney, G., & Cotter, D. R. (2018). Blood-Based Protein Changes in Childhood Are Associated With Increased Risk for Later Psychotic Disorder: Evidence From a Nested Case–Control Study of the ALSPAC Longitudinal Birth Cohort. *Schizophrenia Bulletin*, *44*(2), 297–306. https://doi.org/10.1093/schbul/sbx075

Erata, M. C., Kasap Ustundag, D., Yerlikaya Oral, E., Uslu, O., Erdogan, Y., Tonyali, A., Karacetin, G., & Gonul, A. S. (2024). Erken Başlangıçlı Psikoz ile İlişkili Beyin Bölgelerinin İncelenmesi: Ailesel Yük ve Çevresel Risk Faktörlerinin Etkisi Üzerine bir Voksel Tabanlı Morfometri Çalışması. *Archives of Neuropsychiatry*. https://doi.org/10.29399/npa.28885

Ercan Dogan, A., Aslan Genc, H., Balaç, S., Hun Senol, S., Ayas, G., Dogan, Z., Bora, E., Ceylan, D., & Şar, V. (2025). DMN network and neurocognitive changes associated with dissociative symptoms in major depressive disorder: a research protocol. *Frontiers in Psychiatry*, *16*. https://doi.org/10.3389/fpsyt.2025.1516920

Erickson, C. A., Tessier, C. R., Gross, C., Pedapati, E. v., Wink, L. K., Dominick, K. C., Shaffer, R. C., Rosselot, H., Hong, M. P., Bantel, A. P., Berry-Kravis, E., Horn, P. S., Adams, R., & Sweeney, J. A. (2023). Lymphocytic Extracellular Signal–Regulated Kinase Dysregulation in Autism Spectrum Disorder. *Journal of the American Academy of Child & Adolescent Psychiatry*, *62*(5), 582-592.e2. https://doi.org/10.1016/j.jaac.2022.09.437

Ewais, T., Begun, J., Kenny, M., Chuang, K.-H., Barclay, J., Hay, K., & Kisely, S. (2019). Protocol for a pilot randomised controlled trial of mindfulness-based cognitive therapy in youth with inflammatory bowel disease and depression. *BMJ Open*, *9*(4), e025568. https://doi.org/10.1136/bmjopen-2018-025568

Ewais, T., Begun, J., Kenny, M., Hay, K., Houldin, E., Chuang, K.-H., Tefay, M., & Kisely, S. (2021). Mindfulness based cognitive therapy for youth with inflammatory bowel disease and depression - Findings from a pilot randomised controlled trial. *Journal of Psychosomatic Research*, *149*, 110594. https://doi.org/10.1016/j.jpsychores.2021.110594

Fairchild, G., Passamonti, L., Hurford, G., Hagan, C. C., von dem Hagen, E. A. H., van Goozen, S. H. M., Goodyer, I. M., & Calder, A. J. (2011). Brain Structure Abnormalities in Early-Onset and Adolescent-Onset Conduct Disorder. *American Journal of Psychiatry*, *168*(6), 624–633. https://doi.org/10.1176/appi.ajp.2010.10081184

Fan, L., Booth, J. R., Liu, M., Chou, T., & Gau, S. S. (2021). Developmental differences in neural connectivity for semantic processing in youths with autism. *Journal of Child Psychology and Psychiatry*, *62*(9), 1090–1099. https://doi.org/10.1111/jcpp.13373

Fan, L., Zhang, Z., Ma, X., Liang, L., Yuan, L., Ouyang, L., Wang, Y., Li, Z., Chen, X., He, Y., & Palaniyappan, L. (2025). Brain Age Gap as a Predictor of Early Treatment Response and Functional Outcomes in First-Episode Schizophrenia: A Longitudinal Study: L’écart d’âge cérébral comme prédicteur de la réponse en début de traitement et des résultats fonctionnels dans un premier épisode de schizophrénie : une étude longitudinale. *The Canadian Journal of Psychiatry*, *70*(3), 240–250. https://doi.org/10.1177/07067437241293981

Fassett-Carman, A. N., Moser, A. D., Ruzic, L., Neilson, C., Jones, J., Barnes-Horowitz, S., Schneck, C. D., & Kaiser, R. H. (2023). Amygdala and nucleus accumbens activation during reward anticipation moderates the association between life stressor frequency and depressive symptoms. *Journal of Affective Disorders*, *330*, 309–318. https://doi.org/10.1016/j.jad.2023.02.149

Ferro, M. A., Chan, C. K. Y., Rahman, F. A., Quadrilatero, J., & Timmons, B. W. (2025). Inflammatory Biomarkers Predictive of Psychopathology in Children With Physical Illness. *Brain and Behavior*, *15*(8). https://doi.org/10.1002/brb3.70726

Feurer, C., Granros, M., Calentino, A. E., Suor, J. H., Patel, K., & Burkhouse, K. L. (2021). The interplay of stress and electrocortical reactivity to reward in the prospective prediction of depression symptoms during COVID-19. *Journal of Psychiatric Research*, *140*, 124–131. https://doi.org/10.1016/J.JPSYCHIRES.2021.05.034

Findling, R. L., Goldman, R., Chiu, Y.-Y., Silva, R., Jin, F., Pikalov, A., & Loebel, A. (2015). Pharmacokinetics and Tolerability of Lurasidone in Children and Adolescents With Psychiatric Disorders. *Clinical Therapeutics*, *37*(12), 2788–2797. https://doi.org/10.1016/j.clinthera.2015.11.001

Finger, E. C., Marsh, A. A., Blair, K. S., Reid, M. E., Sims, C., Ng, P., Pine, D. S., & Blair, R. J. R. (2011). Disrupted Reinforcement Signaling in the Orbitofrontal Cortex and Caudate in Youths With Conduct Disorder or Oppositional Defiant Disorder and a High Level of Psychopathic Traits. *American Journal of Psychiatry*, *168*(2), 152–162. https://doi.org/10.1176/appi.ajp.2010.10010129

Fink, E., Reichl, C., Lerch, S., Koenig, J., & Kaess, M. (2025). A biological phenotype of suicide attempt in adolescents with nonsuicidal self-injury: a machine-based learning approach. *Neuropsychopharmacology*, *50*(12), 1817–1826. https://doi.org/10.1038/s41386-025-02176-2

Forbes, E. E., Ryan, N. D., Phillips, M. L., Manuck, S. B., Worthman, C. M., Moyles, D. L., Tarr, J. A., Sciarrillo, S. R., & Dahl, R. E. (2010). Healthy Adolescentsʼ Neural Response to Reward: Associations With Puberty, Positive Affect, and Depressive Symptoms. *Journal of the American Academy of Child & Adolescent Psychiatry*, *49*(2), 162-172e5. https://doi.org/10.1097/00004583-201002000-00010

Forte, M. F., Clougher, D., Segura, À. G., Mezquida, G., Sánchez‐Torres, A. M., Vieta, E., Garriga, M., Lobo, A., González‐Pinto, A. M., Diaz‐Caneja, C. M., Roldan, A., Martínez‐Arán, A., de la Serna, E., Mané, A., Mas, S., Torrent, C., Allott, K., Bernardo, M., & Amoretti, S. (2025). From Genetics to Psychosocial Functioning: Unraveling the Mediating Roles of Cognitive Reserve, Cognition, and Negative Symptoms in First‐Episode Psychosis. *Acta Psychiatrica Scandinavica*, *151*(5), 600–612. https://doi.org/10.1111/acps.13779

Fradkin, Y., Khadka, S., Bessette, K. L., & Stevens, M. C. (2017). The relationship of impulsivity and cortical thickness in depressed and non-depressed adolescents. *Brain Imaging and Behavior*, *11*(5), 1515–1525. https://doi.org/10.1007/s11682-016-9612-8

Francis, H. M., Stevenson, R. J., Chambers, J. R., Gupta, D., Newey, B., & Lim, C. K. (2019). A brief diet intervention can reduce symptoms of depression in young adults – A randomised controlled trial. *PLOS ONE*, *14*(10), e0222768. https://doi.org/10.1371/journal.pone.0222768

Frank, G. K. W., DeGuzman, M. C., Shott, M. E., Laudenslager, M. L., Rossi, B., & Pryor, T. (2018). Association of Brain Reward Learning Response With Harm Avoidance, Weight Gain, and Hypothalamic Effective Connectivity in Adolescent Anorexia Nervosa. *JAMA Psychiatry*, *75*(10), 1071–1080. https://doi.org/10.1001/jamapsychiatry.2018.2151

Frommelt, T., Bäumler, L., Rohleder, N., Schulte-Körne, G., & Platt, B. (2023). Study protocol for a randomized controlled trial investigating the effect of online interpretation bias intervention on stress reactivity in the children of parents with depression: the CoCo study. *BMC Psychiatry*, *23*(1), 378. https://doi.org/10.1186/s12888-023-04803-y

Fuentes-Albero, M., Mafla-España, M. A., Martínez-Raga, J., & Cauli, O. (2024). Autistic Children/Adolescents Have Lower Adherence to the Mediterranean Diet and Higher Salivary IL-6 Concentration: Potential Diet-Inflammation Links? *Pathophysiology : The Official Journal of the International Society for Pathophysiology*, *31*(3), 376–387. https://doi.org/10.3390/pathophysiology31030028

Fujioka, T., Fujisawa, T. X., Inohara, K., Okamoto, Y., Matsumura, Y., Tsuchiya, K. J., Katayama, T., Munesue, T., Tomoda, A., Wada, Y., & Kosaka, H. (2020). Attenuated relationship between salivary oxytocin levels and attention to social information in adolescents and adults with autism spectrum disorder: a comparative study. *Annals of General Psychiatry*, *19*(1), 38. https://doi.org/10.1186/s12991-020-00287-2

Fujioka, T., Tsuchiya, K. J., Saito, M., Hirano, Y., Matsuo, M., Kikuchi, M., Maegaki, Y., Choi, D., Kato, S., Yoshida, T., Yoshimura, Y., Ooba, S., Mizuno, Y., Takiguchi, S., Matsuzaki, H., Tomoda, A., Shudo, K., Ninomiya, M., Katayama, T., & Kosaka, H. (2020). Developmental changes in attention to social information from childhood to adolescence in autism spectrum disorders: a comparative study. *Molecular Autism*, *11*(1), 24. https://doi.org/10.1186/s13229-020-00321-w

Furman, D. J., Hamilton, J. P., Joormann, J., & Gotlib, I. H. (2011). Altered timing of amygdala activation during sad mood elaboration as a function of 5-HTTLPR. *Social Cognitive and Affective Neuroscience*, *6*(3), 270–276. https://doi.org/10.1093/scan/nsq029

Furrer, M., Jaramillo, V., Volk, C., Ringli, M., Aellen, R., Wehrle, F. M., Pugin, F., Kurth, S., Brandeis, D., Schmid, M., Jenni, O. G., & Huber, R. (2019). Sleep EEG slow-wave activity in medicated and unmedicated children and adolescents with attention-deficit/hyperactivity disorder. *Translational Psychiatry*, *9*(1), 324. https://doi.org/10.1038/s41398-019-0659-3

Gabbay, V., Hess, D. A., Liu, S., Babb, J. S., Klein, R. G., & Gonen, O. (2007). Lateralized Caudate Metabolic Abnormalities in Adolescent Major Depressive Disorder: A Proton MR Spectroscopy Study. *American Journal of Psychiatry*, *164*(12), 1881–1889. https://doi.org/10.1176/appi.ajp.2007.06122032

Gabbay, V., Liebes, L., Katz, Y., Liu, S., Mendoza, S., Babb, J. S., Klein, R. G., & Gonen, O. (2010). The kynurenine pathway in adolescent depression: Preliminary findings from a proton MR spectroscopy study. *Progress in Neuro-Psychopharmacology and Biological Psychiatry*, *34*(1), 37–44. https://doi.org/10.1016/J.PNPBP.2009.09.015

Galván, A., & Peris, T. S. (2014). NEURAL CORRELATES OF RISKY DECISION MAKING IN ANXIOUS YOUTH AND HEALTHY CONTROLS. *Depression and Anxiety*, *31*(7), 591–598. https://doi.org/10.1002/da.22276

Gandhi Karunanithi Balaji, Preethi John, & Gokulakannan Kandasamy. (2021). A Single-center, Single Blinded, Randomized Controlled Trial Protocol of Therapeutic Listening Programme Versus Traditional Music Therapy on Depression and Quality of Life in Institutionalized Adolescents. *Medico Legal Update*, *21*(2), 120–125. https://doi.org/10.37506/mlu.v21i2.2657

Gao, W., Jiao, Q., Lu, S., Zhong, Y., Qi, R., Lu, D., Xiao, Q., Yang, F., Lu, G., & Su, L. (2014). Alterations of regional homogeneity in pediatric bipolar depression: a resting-state fMRI study. *BMC Psychiatry*, *14*(1), 222. https://doi.org/10.1186/s12888-014-0222-y

Gao, W., Mu, Q., Cui, D., Zhu, C., Jiao, Q., Su, L., Lu, S., & Yang, R. (2025). Alterations of subcortical structural volume in pediatric bipolar disorder patients with and without psychotic symptoms. *Psychiatry Research: Neuroimaging*, *347*, 111948. https://doi.org/10.1016/j.pscychresns.2025.111948

Gao, W., Wu, J., Liu, Y., Wang, J., Yang, J., & Deng, H. (2025). The relationship between psychological resilience and hair cortisol in adolescents: The influence of family and school environments. *Psychoneuroendocrinology*, *177*, 107471. https://doi.org/10.1016/j.psyneuen.2025.107471

Gao, W., Zhang, K., Jiao, Q., Su, L., Cui, D., Lu, S., & Yang, R. (2025). Machine learning for classification of pediatric bipolar disorder with and without psychotic symptoms based on thalamic subregional structural volume. *BMC Psychiatry*, *25*(1), 574. https://doi.org/10.1186/s12888-025-07018-5

Gao, Y., Feng, R., Ouyang, X., Zhou, Z., Bao, W., Li, Y., Zhuo, L., Hu, X., Li, H., Zhang, L., Huang, G., & Huang, X. (2024). Multivariate association between psychosocial environment, behaviors, and brain functional networks in adolescent depression. *Asian Journal of Psychiatry*, *95*, 104009. https://doi.org/10.1016/J.AJP.2024.104009

Gao, Y., Linke, A., Jao Keehn, R. J., Punyamurthula, S., Jahedi, A., Gates, K., Fishman, I., & Müller, R. (2019). The language network in autism: Atypical functional connectivity with default mode and visual regions. *Autism Research*, *12*(9), 1344–1355. https://doi.org/10.1002/aur.2171

Gariup, M., Gonzalez, A., Lázaro, L., Torres, F., Serra-Pagès, C., & Morer, A. (2015). IL-8 and the innate immunity as biomarkers in acute child and adolescent psychopathology. *Psychoneuroendocrinology*, *62*, 233–242. https://doi.org/10.1016/j.psyneuen.2015.08.017

Garrett, A., Cohen, J. A., Zack, S., Carrion, V., Jo, B., Blader, J., Rodriguez, A., Vanasse, T. J., Reiss, A. L., & Agras, W. S. (2019). Longitudinal changes in brain function associated with symptom improvement in youth with PTSD. *Journal of Psychiatric Research*, *114*, 161–169. https://doi.org/10.1016/j.jpsychires.2019.04.021

Garrett, A. S., Chang, K. D., Singh, M. K., Armstrong, C. C., Walshaw, P. D., & Miklowitz, D. J. (2021). Neural changes in youth at high risk for bipolar disorder undergoing family‐focused therapy or psychoeducation. *Bipolar Disorders*, *23*(6), 604–614. https://doi.org/10.1111/bdi.13045

Garrett, A. S., Miklowitz, D. J., Howe, M. E., Singh, M. K., Acquaye, T. K., Hawkey, C. G., Glover, G. H., Reiss, A. L., & Chang, K. D. (2015). Changes in brain activation following psychotherapy for youth with mood dysregulation at familial risk for bipolar disorder. *Progress in Neuro-Psychopharmacology and Biological Psychiatry*, *56*, 215–220. https://doi.org/10.1016/j.pnpbp.2014.09.007

Garvey, M. A., Perlmutter, S. J., Allen, A. J., Hamburger, S., Lougee, L., Leonard, H. L., Witowski, M. E., Dubbert, B., & Swedo, S. E. (1999). A pilot study of penicillin prophylaxis for neuropsychiatric exacerbations triggered by streptococcal infections. *Biological Psychiatry*, *45*(12), 1564–1571. https://doi.org/10.1016/S0006-3223(99)00020-7

Gaughan, C., Nasa, A., Roman, E., Cullinane, D., Kelly, L., Riaz, S., Brady, C., Browne, C., Sooknarine, V., Mosley, O., Almulla, A., Alsehli, A., Kelliher, A., Murphy, C., O’Hanlon, E., Cannon, M., & Roddy, D. W. (2023). A Pilot Study of Adolescents with Psychotic Experiences: Potential Cerebellar Circuitry Disruption Early Along the Psychosis Spectrum. *The Cerebellum*, *23*(5), 1772–1782. https://doi.org/10.1007/s12311-023-01579-5

Ge, L.-K., Gao, P., Chang, D., Nie, J.-J., Wang, Y.-S., Zuo, X.-N., & Wei, G.-X. (2024). An open data for imaging acute aerobic exercise effects on brain and mind in emerging adulthood. *Scientific Data*, *11*(1), 1422. https://doi.org/10.1038/s41597-024-04270-4

Gehlert, S., & Hartlage, S. (1997). A design for studying the DSM-IV research criteria of premenstrual dysphoric disorder. *Journal of Psychosomatic Obstetrics & Gynecology*, *18*(1), 36–44. https://doi.org/10.3109/01674829709085567

George, G. C., Keding, T. J., Heyn, S. A., & Herringa, R. J. (2022). Longitudinal hippocampal circuit change differentiates persistence and remission of pediatric posttraumatic stress disorder. *Depression and Anxiety*, *39*(1), 8–18. https://doi.org/10.1002/da.23229

Gerin, M. I., Puetz, V. B., Blair, R. J. R., White, S., Sethi, A., Hoffmann, F., Palmer, A. L., Viding, E., & McCrory, E. J. (2017). A neurocomputational investigation of reinforcement-based decision making as a candidate latent vulnerability mechanism in maltreated children. *Development and Psychopathology*, *29*(5), 1689–1705. https://doi.org/10.1017/S095457941700133X

Gershon, E. S., Lee, S. H., Zhou, X., Sweeney, J. A., Tamminga, C., Pearlson, G. A., Clementz, B. A., Keshavan, M. S., Alliey-Rodriguez, N., Hudgens-Haney, M., Keedy, S. K., Glahn, D. C., Asif, H., Lencer, R., & Hill, S. K. (2022). An opportunity for primary prevention research in psychotic disorders. *Schizophrenia Research*, *243*, 433–439. https://doi.org/10.1016/J.SCHRES.2021.07.001

Goldberg, M. C., Lasker, A. G., Zee, D. S., Garth, E., Tien, A., & Landa, R. J. (2002). Deficits in the initiation of eye movements in the absence of a visual target in adolescents with high functioning autism. *Neuropsychologia*, *40*(12), 2039–2049. https://doi.org/10.1016/S0028-3932(02)00059-3

Goldstein, B. I., Lotrich, F., Axelson, D. A., Gill, M. K., Hower, H., Goldstein, T. R., Fan, J., Yen, S., Diler, R., Dickstein, D., Strober, M. A., Iyengar, S., Ryan, N. D., Keller, M. B., & Birmaher, B. (2015). Inflammatory markers among adolescents and young adults with bipolar spectrum disorders. *The Journal of Clinical Psychiatry*, *76*(11), 1556–1563. https://doi.org/10.4088/JCP.14m09395

Gollier-Briant, F., Paillère-Martinot, M.-L., Lemaitre, H., Miranda, R., Vulser, H., Goodman, R., Penttilä, J., Struve, M., Fadai, T., Kappel, V., Poustka, L., Grimmer, Y., Bromberg, U., Conrod, P., Banaschewski, T., Barker, G. J., Bokde, A. L. W., Büchel, C., Flor, H., … Artiges, E. (2016). Neural correlates of three types of negative life events during angry face processing in adolescents. *Social Cognitive and Affective Neuroscience*, *11*(12), 1961–1969. https://doi.org/10.1093/scan/nsw100

González-Domenech, P. J., Díaz Atienza, F., García Pablos, C., Fernández Soto, M. L., Martínez-Ortega, J. M., & Gutiérrez-Rojas, L. (2020). Influence of a Combined Gluten-Free and Casein-Free Diet on Behavior Disorders in Children and Adolescents Diagnosed with Autism Spectrum Disorder: A 12-Month Follow-Up Clinical Trial. *Journal of Autism and Developmental Disorders*, *50*(3), 935–948. https://doi.org/10.1007/s10803-019-04333-1

Gordon, C. T., Frazier, J. A., McKenna, K., Giedd, J., Zametkin, A., Kaysen, D., Albus, K. E., Rapoport, J. L., Zahn, T., Hong, W., & Hommer, D. (1994). Childhood-Onset Schizophrenia: An NIMH Study in Progress. *Schizophrenia Bulletin*, *20*(4), 697–712. https://doi.org/10.1093/schbul/20.4.697

Gorham, L. S., Jernigan, T., Hudziak, J., & Barch, D. M. (2019). Involvement in Sports, Hippocampal Volume, and Depressive Symptoms in Children. *Biological Psychiatry: Cognitive Neuroscience and Neuroimaging*, *4*(5), 484–492. https://doi.org/10.1016/j.bpsc.2019.01.011

Gorka, S. M., Radoman, M., Jimmy, J., Kreutzer, K. A., Manzler, C., & Culp, S. (2023). Behavioral and brain reactivity to uncertain stress prospectively predicts binge drinking in youth. *Neuropsychopharmacology*, *48*(8), 1194–1200. https://doi.org/10.1038/s41386-023-01571-x

Granros, M., Burkhouse, K. L., & Feurer, C. (2024). Relations Between Neighborhood Disadvantage and Electrocortical Reward Processing in Youth at High and Low Risk for Depression. *Research on Child and Adolescent Psychopathology*, *52*(7), 1037–1045. https://doi.org/10.1007/s10802-024-01180-2

Greene, R. K., Spanos, M., Alderman, C., Walsh, E., Bizzell, J., Mosner, M. G., Kinard, J. L., Stuber, G. D., Chandrasekhar, T., Politte, L. C., Sikich, L., & Dichter, G. S. (2018). The effects of intranasal oxytocin on reward circuitry responses in children with autism spectrum disorder. *Journal of Neurodevelopmental Disorders*, *10*(1), 12. https://doi.org/10.1186/s11689-018-9228-y

Grigorian, A., Zou, Y., Kennedy, K. G., Dimick, M. K., MacIntosh, B. J., & Goldstein, B. I. (2025). Association of grey matter cerebral blood flow with white matter integrity in relation to youth bipolar disorder. *Journal of Psychiatry and Neuroscience*, *50*(4), E210–E217. https://doi.org/10.1503/jpn.250019

Groen, W. B., Tesink, C., Petersson, K. M., van Berkum, J., van der Gaag, R. J., Hagoort, P., & Buitelaar, J. K. (2010). Semantic, Factual, and Social Language Comprehension in Adolescents with Autism: An FMRI Study. *Cerebral Cortex*, *20*(8), 1937–1945. https://doi.org/10.1093/cercor/bhp264

Grogans, S. E., Hur, J., Barstead, M. G., Anderson, A. S., Islam, S., Kim, H. C., Kuhn, M., Tillman, R. M., Fox, A. S., Smith, J. F., DeYoung, K. A., & Shackman, A. J. (2024). Neuroticism/Negative Emotionality Is Associated with Increased Reactivity to Uncertain Threat in the Bed Nucleus of the Stria Terminalis, Not the Amygdala. *The Journal of Neuroscience*, *44*(32), e1868232024. https://doi.org/10.1523/JNEUROSCI.1868-23.2024

Grootendorst-van Mil, N. H., Bouter, D. C., Hoogendijk, W. J. G., van Jaarsveld, S. F. L. M., Tiemeier, H., Mulder, C. L., & Roza, S. J. (2021). The iBerry study: a longitudinal cohort study of adolescents at high risk of psychopathology. *European Journal of Epidemiology*, *36*(4), 453–464. https://doi.org/10.1007/s10654-021-00740-w

Grover, S. A., Aubert-Broche, B., Fetco, D., Collins, D. L., Arnold, D. L., Finlayson, M., Banwell, B. L., Motl, R. W., & Yeh, E. A. (2015). Lower physical activity is associated with higher disease burden in pediatric multiple sclerosis. *Neurology*, *85*(19), 1663–1669. https://doi.org/10.1212/WNL.0000000000001939

Gudbrandsen, M., Bletsch, A., Mann, C., Daly, E., Murphy, C. M., Stoencheva, V., Blackmore, C. E., Rogdaki, M., Kushan, L., Bearden, C. E., Murphy, D. G. M., Craig, M. C., & Ecker, C. (2020). Neuroanatomical underpinnings of autism symptomatology in carriers and non-carriers of the 22q11.2 microdeletion. *Molecular Autism*, *11*(1), 46. https://doi.org/10.1186/s13229-020-00356-z

Gulati, S., Narayan, C. L., Mahesan, A., Kamila, G., Kapoor, S., Chaturvedi, P. K., Scaria, V., Velpandian, T., Jauhari, P., Chakrabarty, B., Datta, S. K. R., & Pandey, R. M. (2026). Transmethylation and Oxidative Biomarkers in Children with Autism Spectrum Disorder: A Cross Sectional Study. *Journal of Autism and Developmental Disorders*, *56*(1), 269–277. https://doi.org/10.1007/s10803-024-06542-9

Guldner, S., Sarvasmaa, A. S., Lemaître, H., Massicotte, J., Vulser, H., Miranda, R., Bezivin – Frère, P., Filippi, I., Penttilä, J., Banaschewski, T., Barker, G. J., Bokde, A. L., Bromberg, U., Büchel, C., Conrod, P. J., Desrivières, S., Flor, H., Frouin, V., Gallinat, J., … Martinot, J.-L. (2023). Longitudinal associations between adolescent catch-up sleep, white-matter maturation and internalizing problems. *Developmental Cognitive Neuroscience*, *59*, 101193. https://doi.org/10.1016/j.dcn.2022.101193

Gunde, E., Novak, T., Kopecek, M., Schmidt, M., Propper, L., Stopkova, P., Höschl, C., Duffy, A., Alda, M., & Hajek, T. (2011). White matter hyperintensities in affected and unaffected late teenage and early adulthood offspring of bipolar parents: A two-center high-risk study. *Journal of Psychiatric Research*, *45*(1), 76–82. https://doi.org/10.1016/j.jpsychires.2010.04.019

Guo, J., Zhang, T., Chen, W., Tan, J., Li, X., Zheng, A., Fu, Y., & Qiu, T. (2024). The relationship between serum resolvin D1, NLRP3, cytokine levels, and adolescents with first-episode medication-naïve major depressive disorder. *BMC Psychiatry*, *24*(1), 285. https://doi.org/10.1186/s12888-024-05724-0

Guo, Q., Qiao, D., Zhai, P., Zhang, R., Wen, Y., Liu, P., Li, G., & Liu, Z. (2025). The relationship between the circadian protein PER and adolescent depression: the mediating effect of aberrant functional connectivity of suprachiasmatic nucleus-orbitofrontal cortex. *Journal of Psychiatric Research*, *190*, 225–234. https://doi.org/10.1016/j.jpsychires.2025.08.001

Gur, R. E., Moore, T. M., Rosen, A. F. G., Barzilay, R., Roalf, D. R., Calkins, M. E., Ruparel, K., Scott, J. C., Almasy, L., Satterthwaite, T. D., Shinohara, R. T., & Gur, R. C. (2019). Burden of Environmental Adversity Associated With Psychopathology, Maturation, and Brain Behavior Parameters in Youths. *JAMA Psychiatry*, *76*(9), 966. https://doi.org/10.1001/jamapsychiatry.2019.0943

Haag, K., Hiller, R., Peyk, P., Michael, T., Meiser-Stedman, R., Fearon, P., Ehlers, A., & Halligan, S. L. (2019). A Longitudinal Examination of Heart-Rate and Heart Rate Variability as Risk Markers for Child Posttraumatic Stress Symptoms in an Acute Injury Sample. *Journal of Abnormal Child Psychology*, *47*(11), 1811–1820. https://doi.org/10.1007/s10802-019-00553-2

Haartsen, R., Mason, L., Garces, P., Gui, A., Charman, T., Tillmann, J., Johnson, M. H., Buitelaar, J. K., Loth, E., Murphy, D., Jones, E. J. H., Ahmad, J., Ambrosino, S., Banaschewski, T., Baron-Cohen, S., Bast, N., Baumeister, S., Beckmann, C., Bölte, S., … Rhein, D. von. (2022). Qualitative differences in the spatiotemporal brain states supporting configural face processing emerge in adolescence in autism. *Cortex*, *155*, 13–29. https://doi.org/10.1016/j.cortex.2022.06.010

Häberling, I., Berger, G., Schmeck, K., Held, U., & Walitza, S. (2019). Omega-3 Fatty Acids as a Treatment for Pediatric Depression. A Phase III, 36 Weeks, Multi-Center, Double-Blind, Placebo-Controlled Randomized Superiority Study. *Frontiers in Psychiatry*, *10*. https://doi.org/10.3389/fpsyt.2019.00863

Hadjulis, M., Pipe, R., & Frangou, S. (2004). Normal cerebral volume asymmetries in early onset schizophrenia. *Biological Psychiatry*, *55*(2), 148–153. https://doi.org/10.1016/S0006-3223(02)01905-4

Hafeman, D., Bebko, G., Bertocci, M. A., Fournier, J. C., Chase, H. W., Bonar, L., Perlman, S. B., Travis, M., Gill, M. K., Diwadkar, V. A., Sunshine, J. L., Holland, S. K., Kowatch, R. A., Birmaher, B., Axelson, D., Horwitz, S. M., Arnold, L. E., Fristad, M. A., Frazier, T. W., … Phillips, M. L. (2017). Amygdala-prefrontal cortical functional connectivity during implicit emotion processing differentiates youth with bipolar spectrum from youth with externalizing disorders. *Journal of Affective Disorders*, *208*, 94–100. https://doi.org/10.1016/j.jad.2016.09.064

Hafeman, D. M., Bebko, G., Bertocci, M. A., Fournier, J. C., Bonar, L., Perlman, S. B., Travis, M., Gill, M. K., Diwadkar, V. A., Sunshine, J. L., Holland, S. K., Kowatch, R. A., Birmaher, B., Axelson, D., Horwitz, S. M., Arnold, L. E., Fristad, M. A., Frazier, T. W., Youngstrom, E. A., … Phillips, M. L. (2014). Abnormal deactivation of the inferior frontal gyrus during implicit emotion processing in youth with bipolar disorder: Attenuated by medication. *Journal of Psychiatric Research*, *58*, 129–136. https://doi.org/10.1016/j.jpsychires.2014.07.023

Hafeman, D. M., Chase, H. W., Monk, K., Bonar, L., Hickey, M. B., McCaffrey, A., Graur, S., Manelis, A., Ladouceur, C. D., Merranko, J., Axelson, D. A., Goldstein, B. I., Goldstein, T. R., Birmaher, B., & Phillips, M. L. (2019). Intrinsic functional connectivity correlates of person-level risk for bipolar disorder in offspring of affected parents. *Neuropsychopharmacology : Official Publication of the American College of Neuropsychopharmacology*, *44*(3), 629–634. https://doi.org/10.1038/s41386-018-0264-9

Hafeman, D. M., Ostroff, A. N., Feldman, J., Hickey, M. B., Phillips, M. L., Creswell, D., Birmaher, B., & Goldstein, T. R. (2020). Mindfulness-based intervention to decrease mood lability in at-risk youth: Preliminary evidence for changes in resting state functional connectivity. *Journal of Affective Disorders*, *276*, 23–29. https://doi.org/10.1016/j.jad.2020.06.042

Hagan, C. C., Graham, J. M. E., Tait, R., Widmer, B., van Nieuwenhuizen, A. O., Ooi, C., Whitaker, K. J., Simas, T., Bullmore, E. T., Lennox, B. R., Sahakian, B. J., Goodyer, I. M., & Suckling, J. (2015). Adolescents with current major depressive disorder show dissimilar patterns of age-related differences in ACC and thalamus. *NeuroImage: Clinical*, *7*, 391–399. https://doi.org/10.1016/J.NICL.2014.12.019

Hagan, C. C., Graham, J. M., Widmer, B., Holt, R. J., Ooi, C., van Nieuwenhuizen, A. O., Fonagy, P., Reynolds, S., Target, M., Kelvin, R., Wilkinson, P. O., Bullmore, E. T., Lennox, B. R., Sahakian, B. J., Goodyer, I., & Suckling, J. (2013). Magnetic resonance imaging of a randomized controlled trial investigating predictors of recovery following psychological treatment in adolescents with moderate to severe unipolar depression: study protocol for Magnetic Resonance-Improving Mood with Psychoanalytic and Cognitive Therapies (MR-IMPACT). *BMC Psychiatry*, *13*(1), 247. https://doi.org/10.1186/1471-244X-13-247

Hagler, D. J., Hatton, SeanN., Cornejo, M. D., Makowski, C., Fair, D. A., Dick, A. S., Sutherland, M. T., Casey, B. J., Barch, D. M., Harms, M. P., Watts, R., Bjork, J. M., Garavan, H. P., Hilmer, L., Pung, C. J., Sicat, C. S., Kuperman, J., Bartsch, H., Xue, F., … Dale, A. M. (2019). Image processing and analysis methods for the Adolescent Brain Cognitive Development Study. *NeuroImage*, *202*, 116091. https://doi.org/10.1016/j.neuroimage.2019.116091

Haile, S. R., Peralta, G. P., Raineri, A., Rueegg, S., Ulyte, A., Puhan, M. A., Radtke, T., & Kriemler, S. (2023). *Determinants of health-related quality of life in healthy children and adolescents during the COVID-19 pandemic: results from a prospective longitudinal cohort study*. https://doi.org/10.1101/2023.08.25.23294563

Haile, S. R., Peralta, G. P., Raineri, A., Rueegg, S., Ulytė, A., Puhan, M. A., Radtke, T., & Kriemler, S. (2024). Determinants of health-related quality of life in healthy children and adolescents during the COVID-19 pandemic: Results from a prospective longitudinal cohort study. *European Journal of Pediatrics*, *183*(5), 2273–2283. https://doi.org/10.1007/s00431-024-05459-w

Hajek, T., Bernier, D., Slaney, C., Propper, L., Schmidt, M., Carrey, N., MacQueen, G., Duffy, A., & Alda, M. (2008). A comparison of affected and unaffected relatives of patients with bipolar disorder using proton magnetic resonance spectroscopy. *Journal of Psychiatry & Neuroscience : JPN*, *33*(6), 531–540.

Hajek, T., Cullis, J., Novak, T., Kopecek, M., Höschl, C., Blagdon, R., O’Donovan, C., Bauer, M., Young, L. T., MacQueen, G., & Alda, M. (2012). Hippocampal volumes in bipolar disorders: opposing effects of illness burden and lithium treatment. *Bipolar Disorders*, *14*(3), 261–270. https://doi.org/10.1111/j.1399-5618.2012.01013.x

Hajek, T., Franke, K., Kolenic, M., Capkova, J., Matejka, M., Propper, L., Uher, R., Stopkova, P., Novak, T., Paus, T., Kopecek, M., Spaniel, F., & Alda, M. (2019). Brain Age in Early Stages of Bipolar Disorders or Schizophrenia. *Schizophrenia Bulletin*, *45*(1), 190–198. https://doi.org/10.1093/schbul/sbx172

Hajek, T., Gunde, E., Bernier, D., Slaney, C., Propper, L., MacQueen, G., Duffy, A., & Alda, M. (2008). Pituitary volumes in relatives of bipolar patients. *European Archives of Psychiatry and Clinical Neuroscience*, *258*(6), 357–362. https://doi.org/10.1007/s00406-008-0804-0

Hajek, T., Gunde, E., Slaney, C., Propper, L., MacQueen, G., Duffy, A., & Alda, M. (2009). Amygdala and Hippocampal Volumes in Relatives of Patients with Bipolar Disorder: A High—Risk Study. *The Canadian Journal of Psychiatry*, *54*(11), 726–733. https://doi.org/10.1177/070674370905401102

Halari, R., Simic, M., Pariante, C. M., Papadopoulos, A., Cleare, A., Brammer, M., Fombonne, E., & Rubia, K. (2009). Reduced activation in lateral prefrontal cortex and anterior cingulate during attention and cognitive control functions in medication‐naïve adolescents with depression compared to controls. *Journal of Child Psychology and Psychiatry*, *50*(3), 307–316. https://doi.org/10.1111/j.1469-7610.2008.01972.x

Halldorsdottir, T., Kristjansson, A. L., Asgeirsdottir, B. B., Thorisdottir, I. E., Sigfusson, J., Tolgyes, E. M. J., Valdimarsdottir, H. B., Allegrante, J., & Sigfusdottir, I. D. (2021). A multi-level developmental approach towards understanding adolescent mental health and behaviour: rationale, design and methods of the LIFECOURSE study in Iceland. *Social Psychiatry and Psychiatric Epidemiology*, *56*(3), 519–529. https://doi.org/10.1007/s00127-020-01995-6

Han, D. H., Chung, U., Shin, Y. J., & Renshaw, P. (2016). A prosocial online game for social cognition training in adolescents with high-functioning autism: an fMRI study. *Neuropsychiatric Disease and Treatment*, 651. https://doi.org/10.2147/NDT.S94669

Han, D. H., Lee, Y. S., Shi, X., & Renshaw, P. F. (2014). Proton magnetic resonance spectroscopy (MRS) in on-line game addiction. *Journal of Psychiatric Research*, *58*, 63–68. https://doi.org/10.1016/J.JPSYCHIRES.2014.07.007

Han, D. H., Yoo, H. J., Kim, B. N., McMahon, W., & Renshaw, P. F. (2014). Brain Activity of Adolescents with High Functioning Autism in Response to Emotional Words and Facial Emoticons. *PLoS ONE*, *9*(3), e91214. https://doi.org/10.1371/journal.pone.0091214

Han, G.-Q., Kat, S.-C., Wang, H., Yang, Y.-L., Ma, Z.-H., Yin, T.-N., Sun, Y.-J., Tang, X.-Z., Gong, X.-Y., Wang, D., Li, L., Sun, B.-X., Zhao, L.-Y., Su, X., Chen, J.-L., Chen, X., Wang, H.-L., Li, X.-Y., Liu, H.-L., … Liu, J. (2025). Alterations in whole-brain white matter fiber networks in individuals with autism spectrum disorder after social skills training. *Brain Research Bulletin*, *229*, 111466. https://doi.org/10.1016/j.brainresbull.2025.111466

Hanaie, R., Mohri, I., Kagitani-Shimono, K., Tachibana, M., Azuma, J., Matsuzaki, J., Watanabe, Y., Fujita, N., & Taniike, M. (2013). Altered Microstructural Connectivity of the Superior Cerebellar Peduncle is Related to Motor Dysfunction in Children with Autistic Spectrum Disorders. *The Cerebellum*, *12*(5), 645–656. https://doi.org/10.1007/s12311-013-0475-x

Haney-Caron, E., Caprihan, A., & Stevens, M. C. (2014). DTI-measured white matter abnormalities in adolescents with Conduct Disorder. *Journal of Psychiatric Research*, *48*(1), 111–120. https://doi.org/10.1016/j.jpsychires.2013.09.015

Harlalka, V., Bapi, R. S., Vinod, P. K., & Roy, D. (2018). Age, Disease, and Their Interaction Effects on Intrinsic Connectivity of Children and Adolescents in Autism Spectrum Disorder Using Functional Connectomics. *Brain Connectivity*, *8*(7), 407–419. https://doi.org/10.1089/brain.2018.0616

Hassan, A. H., Salem, A. M., Shehab, A. A., & el Hossiny, R. M. (2025). Diagnostic and severity correlation of serum zinc, copper, and anti-myelin basic protein antibodies in children with autism spectrum disorder: a cross-sectional controlled study. *Egyptian Pediatric Association Gazette*, *73*(1), 62. https://doi.org/10.1186/s43054-025-00406-2

Hassan, Z. R., Zekry, K. M., Heikal, E. A., Ibrahim, H. F., Khirala, S. K., Abd El-Hamid, S. M., Amin, D. R., Seliem, N., El-Aal, G. N. A., Alkherkhisy, M. M., Elhamid, S. A. A., Mahgoub, E. A., Hefny, M. E. N., el Nady, G. H., & Badr, M. S. (2023). Toxoplasmosis and cytomegalovirus infection and their role in Egyptian autistic children. *Parasitology Research*, *122*(5), 1177–1187. https://doi.org/10.1007/s00436-023-07818-2

Haugen, T., Halvorsen, J. Ø., Friborg, O., Mork, P. J., Mikkelsen, G., Schei, B., & Hagemann, C. (2024). Early Intervention after Rape to prevent post-traumatic stress symptoms (the EIR-study): an internal pilot study of a randomized controlled trial. *Pilot and Feasibility Studies*, *10*(1), 118. https://doi.org/10.1186/s40814-024-01541-0

He, X., Song, J., Gao, H., Li, Z., Wang, X., Zeng, Q., Xiao, Y., Feng, J., Zhou, D., & Wang, G. (2023). Serum brain-derived neurotrophic factor and glial cell-derived neurotrophic factor in patients with first-episode depression at different ages. *International Journal of Psychiatry in Clinical Practice*, *27*(2), 118–126. https://doi.org/10.1080/13651501.2022.2107938

He, X.-Q., Hu, J.-H., Peng, X.-Y., Zhao, L., Zhou, D.-D., Ma, L.-L., Zhang, Z.-Y., Tao, W.-Q., Liu, X.-Y., Kuang, L., & Wang, W. (2024). EEG microstate analysis reveals large-scale brain network alterations in depressed adolescents with suicidal ideation. *Journal of Affective Disorders*, *346*, 57–63. https://doi.org/10.1016/j.jad.2023.11.018

He, X.-Y., Huang, Z.-H., Wang, F., Chen, Z.-L., Wang, S.-B., Jia, F.-J., & Hou, C.-L. (2023). Gene Polymorphisms and Expression of NRG1, DAOA, and DISC1 Genes in a Chinese Han Population with an Ultra-High Risk for Psychosis. *Neuropsychiatric Disease and Treatment*, *Volume 19*, 2521–2533. https://doi.org/10.2147/NDT.S434856

He, Y., Yu, Q., Yang, T., Zhang, Y., Zhang, K., Jin, X., Wu, S., Gao, X., Huang, C., Cui, X., & Luo, X. (2021). Abnormalities in Electroencephalographic Microstates Among Adolescents With First Episode Major Depressive Disorder. *Frontiers in Psychiatry*, *12*. https://doi.org/10.3389/fpsyt.2021.775156

Hegarty, J. P., Monterrey, J. C., Tian, Q., Cleveland, S. C., Gong, X., Phillips, J. M., Wolke, O. N., McNab, J. A., Hallmayer, J. F., Reiss, A. L., Hardan, A. Y., & Lazzeroni, L. C. (2024). A Twin Study of Altered White Matter Heritability in Youth With Autism Spectrum Disorder. *Journal of the American Academy of Child & Adolescent Psychiatry*, *63*(1), 65–79. https://doi.org/10.1016/j.jaac.2023.05.030

Hegarty, J. P., Pegoraro, L. F. L., Lazzeroni, L. C., Raman, M. M., Hallmayer, J. F., Monterrey, J. C., Cleveland, S. C., Wolke, O. N., Phillips, J. M., Reiss, A. L., & Hardan, A. Y. (2020). Genetic and environmental influences on structural brain measures in twins with autism spectrum disorder. *Molecular Psychiatry*, *25*(10), 2556–2566. https://doi.org/10.1038/s41380-018-0330-z

Helgadóttir, H., Gudmundsson, Ó. Ó., Baldursson, G., Magnússon, P., Blin, N., Brynjólfsdóttir, B., Emilsdóttir, Á., Gudmundsdóttir, G. B., Lorange, M., Newman, P. K., Jóhannesson, G. H., & Johnsen, K. (2015). Electroencephalography as a clinical tool for diagnosing and monitoring attention deficit hyperactivity disorder: a cross-sectional study. *BMJ Open*, *5*(1), e005500. https://doi.org/10.1136/bmjopen-2014-005500

Hemmings, A., Gallop, L., İnce, B., Cutinha, D., Kan, C., Simic, M., Zadeh, E., Malvisi, I., McKenzie, K., Zocek, L., Sharpe, H., O’Daly, O., Campbell, I. C., & Schmidt, U. (2024). A randomised controlled feasibility trial of intermittent theta burst stimulation with an open longer‐term follow‐up for young people with persistent anorexia nervosa (RaISE): Study protocol. *European Eating Disorders Review*, *32*(3), 575–588. https://doi.org/10.1002/erv.3073

Herd, T., & Kim-Spoon, J. (2021). A Systematic Review of Associations Between Adverse Peer Experiences and Emotion Regulation in Adolescence. *Clinical Child and Family Psychology Review*, *24*(1), 141–163. https://doi.org/10.1007/s10567-020-00337-x

Hernandez, L. M., Green, S. A., Lawrence, K. E., Inada, M., Liu, J., Bookheimer, S. Y., & Dapretto, M. (2020). Social Attention in Autism: Neural Sensitivity to Speech Over Background Noise Predicts Encoding of Social Information. *Frontiers in Psychiatry*, *11*. https://doi.org/10.3389/fpsyt.2020.00343

Herpertz, S. C., Mueller, B., Qunaibi, M., Lichterfeld, C., Konrad, K., & Herpertz-Dahlmann, B. (2005). Response to Emotional Stimuli in Boys With Conduct Disorder. *American Journal of Psychiatry*, *162*(6), 1100–1107. https://doi.org/10.1176/appi.ajp.162.6.1100

Heyn, S. A., Bailowitz, S., Russell, J. D., & Herringa, R. J. (2022). Sex‐based variations of prefrontal structure and longitudinal symptoms in pediatric posttraumatic stress disorder. *Depression and Anxiety*, *39*(12), 902–912. https://doi.org/10.1002/da.23296

Heyn, S. A., Keding, T. J., Cisler, J., McLaughlin, K., & Herringa, R. J. (2025). Differential gray matter correlates and machine learning prediction of abuse and internalizing psychopathology in adolescent females. *Scientific Reports*, *15*(1), 651. https://doi.org/10.1038/s41598-024-84616-5

Hillegers, M. H. J., Reichart, C. G., Wals, M., Verhulst, F. C., Ormel, J., Nolen, W. A., & Drexhage, H. A. (2007). Signs of a higher prevalence of autoimmune thyroiditis in female offspring of bipolar parents. *European Neuropsychopharmacology*, *17*(6–7), 394–399. https://doi.org/10.1016/J.EURONEURO.2006.10.005

Ho, R. A., Hall, G. B., Noseworthy, M. D., & DeMatteo, C. (2018). An Emotional Go/No-Go fMRI study in adolescents with depressive symptoms following concussion. *International Journal of Psychophysiology*, *132*, 62–73. https://doi.org/10.1016/J.IJPSYCHO.2017.09.021

Ho, T. C., Teresi, G. I., Segarra, J. R., Ojha, A., Walker, J. C., Gu, M., Spielman, D. M., Sacchet, M. D., Jiang, F., Rosenberg-Hasson, Y., Maecker, H., & Gotlib, I. H. (2021). Higher Levels of Pro-inflammatory Cytokines Are Associated With Higher Levels of Glutamate in the Anterior Cingulate Cortex in Depressed Adolescents. *Frontiers in Psychiatry*, *12*. https://doi.org/10.3389/fpsyt.2021.642976

Hogeveen, J., Campbell, E. M., Mullins, T. S., Robertson-Benta, C. R., Quinn, D. K., Mayer, A. R., & Cavanagh, J. F. (2024). Neural response to monetary incentives in acquired adolescent depression after mild traumatic brain injury: Stage 2 Registered Report. *Brain Communications*, *6*(5). https://doi.org/10.1093/braincomms/fcae250

Holmes, J., Bryant, A., & Gathercole, S. E. (2019). Protocol for a transdiagnostic study of children with problems of attention, learning and memory (CALM). *BMC Pediatrics*, *19*(1), 10. https://doi.org/10.1186/s12887-018-1385-3

Höper, S., Kröller, F., Heinze, A.-L., Bardtke, K. F., Kaess, M., & Koenig, J. (2024). Prefrontal cortex activation under stress as a function of borderline personality disorder in female adolescents engaging in non-suicidal self-injury. *BJPsych Open*, *10*(5), e142. https://doi.org/10.1192/bjo.2024.728

Horn, S. R., Leve, L. D., Levitt, P., & Fisher, P. A. (2019). Childhood adversity, mental health, and oxidative stress: A pilot study. *PLOS ONE*, *14*(4), e0215085. https://doi.org/10.1371/journal.pone.0215085

Horton, M. K., Hsu, L., Henn, B. C., Margolis, A., Austin, C., Svensson, K., Schnaas, L., Gennings, C., Hu, H., Wright, R., Rojo, M. M. T., & Arora, M. (2018). Dentine biomarkers of prenatal and early childhood exposure to manganese, zinc and lead and childhood behavior. *Environment International*, *121*, 148–158. https://doi.org/10.1016/J.ENVINT.2018.08.045

Hsu, J.-W., Chen, L.-C., Lin, W.-C., Bai, Y.-M., Tsai, S.-J., & Chen, M.-H. (2025). Reduced levels of vascular endothelial growth factor were associated with psychopathology in youth with first-episode schizophrenia. *Schizophrenia Research*, *284*, 269–274. https://doi.org/10.1016/j.schres.2025.08.020

Hu, D., Wang, Y.-X., Chen, W.-J., Zhang, Y., Li, H.-H., Xiong, L., Zhu, H.-P., Chen, H.-Y., Peng, S.-X., Wan, Z.-H., Zhang, Y., & Du, Y.-K. (2017). Associations of phthalates exposure with attention deficits hyperactivity disorder: A case-control study among Chinese children. *Environmental Pollution*, *229*, 375–385. https://doi.org/10.1016/j.envpol.2017.05.089

Hu, S., Sun, H., Bao, C., Shen, N., Yan, R., Hua, L., Chen, Z., Lin, P., Wang, X., Yao, Z., & Lu, Q. (2025). Functional brain abnormalities in adolescents and young adults with bipolar depression with mixed features: Insights from resting-state fMRI. *Journal of Psychiatric Research*, *190*, 490–498. https://doi.org/10.1016/j.jpsychires.2025.09.001

Hu, S., Zhu, L., & Zhang, X.-Y. (2025). Structural brain alterations in patients with anxious depression: evidence from the REST-meta-MDD project. *Frontiers in Psychiatry*, *16*. https://doi.org/10.3389/fpsyt.2025.1589040

Hu, W., Ran, X., Wu, Z., Zhu, H., Kou, Y., Zhang, S., Yang, G., Li, W., Yang, Y., Lv, L., & Zhang, Y. (2024). Short-term antipsychotic treatment reduces functional connectivity of the striatum in first-episode drug-naïve early-onset schizophrenia. *Schizophrenia Research*, *270*, 281–288. https://doi.org/10.1016/j.schres.2024.06.016

Huang, H., Gundapuneedi, T., & Rao, U. (2012). White Matter Disruptions in Adolescents Exposed to Childhood Maltreatment and Vulnerability to Psychopathology. *Neuropsychopharmacology*, *37*(12), 2693–2701. https://doi.org/10.1038/npp.2012.133

Huang, X.-C.-C., Guan, Q.-Y., Jiang, M.-J., Yu, Q.-T., Ou, W.-Q., Wang, Y.-Y., Xiao, Z., Zhang, J.-F., Liu, X.-C., Hou, C.-L., & Chen, M. (2025). Abnormal eye movement patterns in adolescent and early adulthood MDD patients with and without psychotic symptoms: A multi-paradigm feature-based study. *Psychiatry Research: Neuroimaging*, *353*, 112064. https://doi.org/10.1016/j.pscychresns.2025.112064

Huang, Y., Xu, L., Kuang, L., Wang, W., Cao, J., & Xiao, M.-N. (2020). Abnormal brain activity in adolescents with Internet addiction who attempt suicide: an assessment using functional magnetic resonance imaging. *Neural Regeneration Research*, *15*(8), 1554–1559. https://doi.org/10.4103/1673-5374.274346

Hur, J., Tillman, R. M., Kim, H. C., Didier, P., Anderson, A. S., Islam, S., Stockbridge, M. D., de Los Reyes, A., DeYoung, K. A., Smith, J. F., & Shackman, A. J. (2023). *Adolescent social anxiety is associated with diminished discrimination of anticipated threat and safety in the bed nucleus of the stria terminalis*. https://doi.org/10.1101/2023.10.30.564701

Hur, J., Tillman, R. M., Kim, H. C., Didier, P., Anderson, A. S., Islam, S., Stockbridge, M. D., de Los Reyes, A., DeYoung, K. A., Smith, J. F., & Shackman, A. J. (2025). Adolescent social anxiety is associated with diminished discrimination of anticipated threat and safety in the bed nucleus of the stria terminalis. *Journal of Psychopathology and Clinical Science*, *134*(1), 41–56. https://doi.org/10.1037/abn0000940

Huyser, C., Veltman, D. J., Wolters, L. H., de Haan, E., & Boer, F. (2010). Functional Magnetic Resonance Imaging During Planning Before and After Cognitive-Behavioral Therapy in Pediatric Obsessive-Compulsive Disorder. *Journal of the American Academy of Child & Adolescent Psychiatry*, *49*(12), 1238-1248.e5. https://doi.org/10.1016/J.JAAC.2010.08.007

Hwang, S., Chung, U., Chang, Y., Kim, E., Suk, J.-W., Meffert, H., Kratochvil, C., Leibenluft, E., & Blair, J. (2021). Neural Responses to Fluoxetine in Youths with Disruptive Behavior and Trauma Exposure: A Pilot Study. *Journal of Child and Adolescent Psychopharmacology*, *31*(8), 562–571. https://doi.org/10.1089/cap.2020.0174

Hwang, S., Suk, J.-W., Meffert, H., Lerdahl, A., Garvey, W. F., Edwards, R., Delizza, A., Soltis-Vaughan, B., Cordts, K., Leibenluft, E., & Blair, R. J. R. (2024). Neural Responses to Intranasal Oxytocin in Youths With Severe Irritability. *American Journal of Psychiatry*, *181*(4), 291–298. https://doi.org/10.1176/appi.ajp.20230174

Hwang, S., White, S. F., Nolan, Z. T., Craig Williams, W., Sinclair, S., & Blair, R. J. R. (2015). Executive attention control and emotional responding in attention-deficit/hyperactivity disorder — A functional MRI study. *NeuroImage: Clinical*, *9*, 545–554. https://doi.org/10.1016/j.nicl.2015.10.005

Hyatt, C. J., Haney-Caron, E., & Stevens, M. C. (2012). Cortical Thickness and Folding Deficits in Conduct-Disordered Adolescents. *Biological Psychiatry*, *72*(3), 207–214. https://doi.org/10.1016/J.BIOPSYCH.2011.11.017

Iadipaolo, A. S., Marusak, H. A., Paulisin, S. M., Sala-Hamrick, K., Crespo, L. M., Elrahal, F., Peters, C., Brown, S., & Rabinak, C. A. (2018). Distinct neural correlates of trait resilience within core neurocognitive networks in at-risk children and adolescents. *NeuroImage: Clinical*, *20*, 24–34. https://doi.org/10.1016/j.nicl.2018.06.026

Iftene, F., Predescu, E., Stefan, S., & David, D. (2015). Rational-emotive and cognitive-behavior therapy (REBT/CBT) versus pharmacotherapy versus REBT/CBT plus pharmacotherapy in the treatment of major depressive disorder in youth; A randomized clinical trial. *Psychiatry Research*, *225*(3), 687–694. https://doi.org/10.1016/j.psychres.2014.11.021

Imbiriba, T., Demirkaya, A., Singh, A., Erdogmus, D., & Goodwin, M. S. (2023). Wearable Biosensing to Predict Imminent Aggressive Behavior in Psychiatric Inpatient Youths With Autism. *JAMA Network Open*, *6*(12), e2348898. https://doi.org/10.1001/jamanetworkopen.2023.48898

Insel, C., Glenn, C. R., Nock, M. K., & Somerville, L. H. (2019). Aberrant striatal tracking of reward magnitude in youth with current or past-year depression. *Journal of Abnormal Psychology*, *128*(1), 44–56. https://doi.org/10.1037/abn0000389

Introne, W. J., Westbroek, W., Groden, C. A., Bhambhani, V., Golas, G. A., Baker, E. H., Lehky, T. J., Snow, J., Ziegler, S. G., Malicdan, M. C. v., Adams, D. R., Dorward, H. M., Hess, R. A., Huizing, M., Gahl, W. A., & Toro, C. (2017). Neurologic involvement in patients with atypical Chediak-Higashi disease. *Neurology*, *88*(7). https://doi.org/10.1212/WNL.0000000000003622

Iorfino, F., Hickie, I. B., Lee, R. S. C., Lagopoulos, J., & Hermens, D. F. (2016). The underlying neurobiology of key functional domains in young people with mood and anxiety disorders: a systematic review. *BMC Psychiatry*, *16*(1), 156. https://doi.org/10.1186/s12888-016-0852-3

Iqbal, M., Cox, S. M. L., Jaworska, N., Tippler, M., Castellanos-Ryan, N., Parent, S., Dagher, A., Vitaro, F., Brendgen, M. R., Boivin, M., Pihl, R. O., Côté, S. M., Tremblay, R. E., Séguin, J. R., & Leyton, M. (2022). A three-factor model of common early onset psychiatric disorders: temperament, adversity, and dopamine. *Neuropsychopharmacology*, *47*(3), 752–758. https://doi.org/10.1038/s41386-021-01187-z

Islam, M. S., Page-Hefley, S., Hernandez, A. P., Whelchel, L., Crasto, C., Viator, W., Money, T., Awosile, B., Howard, N., & Vasylyeva, T. L. (2023). Change in Urinary Inflammatory Biomarkers and Psychological Health with Gut Microbiome Modulation after Six Months of a Lifestyle Modification Program in Children. *Nutrients*, *15*(19), 4243. https://doi.org/10.3390/nu15194243

Iveson, M. H., Ball, E. L., Doherty, J., Pugh, C., Vashishta, S., Palmer, C. N. A., & McIntosh, A. (2024). Cohort profile: The Scottish SHARE Mental Health (SHARE-MH) cohort – linkable survey, genetic and routinely collected data for mental health research. *BMJ Open*, *14*(1), e078246. https://doi.org/10.1136/bmjopen-2023-078246

Jack, A., & Morris, J. P. (2014). Neocerebellar contributions to social perception in adolescents with autism spectrum disorder. *Developmental Cognitive Neuroscience*, *10*, 77–92. https://doi.org/10.1016/j.dcn.2014.08.001

Jackowski, A. P., Douglas-Palumberi, H., Jackowski, M., Win, L., Schultz, R. T., Staib, L. W., Krystal, J. H., & Kaufman, J. (2008). Corpus callosum in maltreated children with posttraumatic stress disorder: A diffusion tensor imaging study. *Psychiatry Research: Neuroimaging*, *162*(3), 256–261. https://doi.org/10.1016/j.pscychresns.2007.08.006

Jacobs, R. H., Watkins, E. R., Peters, A. T., Feldhaus, C. G., Barba, A., Carbray, J., & Langenecker, S. A. (2016). Targeting Ruminative Thinking in Adolescents at Risk for Depressive Relapse: Rumination-Focused Cognitive Behavior Therapy in a Pilot Randomized Controlled Trial with Resting State fMRI. *PLOS ONE*, *11*(11), e0163952. https://doi.org/10.1371/journal.pone.0163952

Jankowski, K. F., Batres, J., Scott, H., Smyda, G., Pfeifer, J. H., & Quevedo, K. (2018). Feeling left out: depressed adolescents may atypically recruit emotional salience and regulation networks during social exclusion. *Social Cognitive and Affective Neuroscience*, *13*(8), 863–876. https://doi.org/10.1093/scan/nsy055

Janssen, T. W. P., Bink, M., Weeda, W. D., Geladé, K., van Mourik, R., Maras, A., & Oosterlaan, J. (2017). Learning curves of theta/beta neurofeedback in children with ADHD. *European Child & Adolescent Psychiatry*, *26*(5), 573–582. https://doi.org/10.1007/s00787-016-0920-8

Jaspers-Fayer, F., Lin, S. Y., Best, J. R., Thorsen, A. L., Negreiros, J., Chan, E., Ellwyn, R., Lin, B., de Wit, S., van den Heuvel, O. A., & Stewart, S. E. (2022). An fMRI study of cognitive planning before and after symptom provocation in pediatric obsessive–compulsive disorder. *Journal of Psychiatry and Neuroscience*, *47*(6), E409–E420. https://doi.org/10.1503/jpn.220064

Jaspers-Fayer, F., Lin, S. Y., Chan, E., Ellwyn, R., Lim, R., Best, J., Belschner, L., Lang, D., Heran, M. K. M., Woodward, T. S., & Stewart, S. E. (2019). Neural correlates of symptom provocation in pediatric obsessive-compulsive disorder. *NeuroImage: Clinical*, *24*, 102034. https://doi.org/10.1016/j.nicl.2019.102034

Jaster, A. M., Ely, S. L., Zundel, C. G., Gowatch, L. C., Shampine, M., Carpenter, C., O’Mara, E., Bhogal, A., Tamimi, R., Lewis, C., Sharma, K., Losiowski, J., & Marusak, H. A. (2026). Relationship between circulating plasma endocannabinoids in adolescents and maternal depressive symptoms. *Psychopharmacology*, *243*(2), 369–381. https://doi.org/10.1007/s00213-025-06831-w

Jenness, J. L., Peverill, M., Miller, A. B., Heleniak, C., Robertson, M. M., Sambrook, K. A., Sheridan, M. A., & McLaughlin, K. A. (2021). Alterations in neural circuits underlying emotion regulation following child maltreatment: a mechanism underlying trauma-related psychopathology. *Psychological Medicine*, *51*(11), 1880–1889. https://doi.org/10.1017/S0033291720000641

Jeong, E., Park, J., Kim, Y., & Woo, M. (2021). Aerobic Exercise Mediates Neural Inefficiency Among Adolescents At Risk for Suicide. *Perceptual and Motor Skills*, *128*(3), 1130–1149. https://doi.org/10.1177/0031512521998281

Jewkes, R., Nduna, M., Levin, J., Jama, N., Dunkle, K., Puren, A., & Duvvury, N. (2008). Impact of Stepping Stones on incidence of HIV and HSV-2 and sexual behaviour in rural South Africa: cluster randomised controlled trial. *BMJ*, *337*(aug07 1), a506–a506. https://doi.org/10.1136/bmj.a506

Jha, M. K., Cai, L., Minhajuddin, A., Fatt, C. C., Furman, J. L., Gadad, B. S., Mason, B. L., Greer, T. L., Hughes, J. L., Xiao, G., Emslie, G., Kennard, B., Mayes, T., & Trivedi, M. H. (2020). Dysfunctional adaptive immune response in adolescents and young adults with suicide behavior. *Psychoneuroendocrinology*, *111*, 104487. https://doi.org/10.1016/j.psyneuen.2019.104487

Jiang, X., Zai, C. C., Dimick, M. K., Kennedy, J. L., Young, L. T., Birmaher, B., & Goldstein, B. I. (2024). Psychiatric Polygenic Risk Scores Across Youth With Bipolar Disorder, Youth at High Risk for Bipolar Disorder, and Controls. *Journal of the American Academy of Child & Adolescent Psychiatry*, *63*(11), 1149–1157. https://doi.org/10.1016/j.jaac.2023.12.009

Jiang, Y., Qin, M., Teng, T., Li, X., Yu, Y., Wang, J., Wu, H., He, Y., Zhou, X., & Xie, P. (2022). Identification of Sex-Specific Plasma Biomarkers Using Metabolomics for Major Depressive Disorder in Children and Adolescents. *Frontiers in Psychiatry*, *13*. https://doi.org/10.3389/fpsyt.2022.929207

Jiao, Q., Ding, J., Lu, G., Su, L., Zhang, Z., Wang, Z., Zhong, Y., Li, K., Ding, M., & Liu, Y. (2011). Increased Activity Imbalance in Fronto-Subcortical Circuits in Adolescents with Major Depression. *PLoS ONE*, *6*(9), e25159. https://doi.org/10.1371/journal.pone.0025159

Jin, C., Zhang, T., Cai, C., Bi, Y., Li, Y., Yu, D., Zhang, M., & Yuan, K. (2016). Abnormal prefrontal cortex resting state functional connectivity and severity of internet gaming disorder. *Brain Imaging and Behavior*, *10*(3), 719–729. https://doi.org/10.1007/s11682-015-9439-8

Jirsaraie, R. J., Kaczkurkin, A. N., Rush, S., Piiwia, K., Adebimpe, A., Bassett, D. S., Bourque, J., Calkins, M. E., Cieslak, M., Ciric, R., Cook, P. A., Davila, D., Elliott, M. A., Leibenluft, E., Murtha, K., Roalf, D. R., Rosen, A. F. G., Ruparel, K., Shinohara, R. T., … Satterthwaite, T. D. (2019). Accelerated cortical thinning within structural brain networks is associated with irritability in youth. *Neuropsychopharmacology*, *44*(13), 2254–2262. https://doi.org/10.1038/s41386-019-0508-3

Ju, Y., Zhao, T., Gao, Z., Hu, W., Luo, J., Cheng, N., Liu, C., Jiang, Y., Hong, B., Ji, T., & Yan, Y. (2025). Brain age prediction model based on electroencephalogram signal and its application in children with autism spectrum disorders. *Frontiers in Neurology*, *16*. https://doi.org/10.3389/fneur.2025.1605291

Juarez-Martinez, E. L., Sprengers, J. J., Cristian, G., Oranje, B., van Andel, D. M., Avramiea, A.-E., Simpraga, S., Houtman, S. J., Hardstone, R., Gerver, C., Jan van der Wilt, G., Mansvelder, H. D., Eijkemans, M. J. C., Linkenkaer-Hansen, K., & Bruining, H. (2023). Prediction of Behavioral Improvement Through Resting-State Electroencephalography and Clinical Severity in a Randomized Controlled Trial Testing Bumetanide in Autism Spectrum Disorder. *Biological Psychiatry: Cognitive Neuroscience and Neuroimaging*, *8*(3), 251–261. https://doi.org/10.1016/j.bpsc.2021.08.009

Juliá-Palacios, N., Olivella, M., Sigatullina Bondarenko, M., Ibáñez-Micó, S., Muñoz-Cabello, B., Alonso-Luengo, O., Soto-Insuga, V., García-Navas, D., Cuesta-Herraiz, L., Andreo-Lillo, P., Aguilera-Albesa, S., Hedrera-Fernández, A., González Alguacil, E., Sánchez-Carpintero, R., Martín del Valle, F., Jiménez González, E., Cean Cabrera, L., Medina-Rivera, I., Perez-Ordoñez, M., … García-Cazorla, Á. (2024). L-serine treatment in patients with *GRIN* -related encephalopathy: a phase 2A, non-randomized study. *Brain*, *147*(5), 1653–1666. https://doi.org/10.1093/brain/awae041

Kaess, M., Parzer, P., Mehl, L., Weil, L., Strittmatter, E., Resch, F., & Koenig, J. (2017). Stress vulnerability in male youth with Internet Gaming Disorder. *Psychoneuroendocrinology*, *77*, 244–251. https://doi.org/10.1016/j.psyneuen.2017.01.008

Kaligis, F., Ismail, R. I., Wiguna, T., Prasetyo, S., Gunardi, H., Indriatmi, W., Pasaribu, M. M., Pandia, V., Minayati, K., Magdalena, C. C., Nurraga, G. W., Pramatirta, B., Calvin, N., & Sourander, A. (2023). Effectiveness of an online mental health strengthening module to build resilience and overcome stress for transitional aged medical students. *Frontiers in Digital Health*, *5*. https://doi.org/10.3389/fdgth.2023.1207583

Kalita, J., Agarwal, R., Chandra, S., & Misra, U. K. (2013). A study of neurobehavioral, clinical psychometric, and P3 changes in vitamin B12 deficiency neurological syndrome. *Nutritional Neuroscience*, *16*(1), 39–46. https://doi.org/10.1179/1476830512Y.0000000028

Kaliukhovich, D. A., Manyakov, N. v., Bangerter, A., Ness, S., Skalkin, A., Boice, M., Goodwin, M. S., Dawson, G., Hendren, R., Leventhal, B., Shic, F., & Pandina, G. (2021). Visual Preference for Biological Motion in Children and Adults with Autism Spectrum Disorder: An Eye-Tracking Study. *Journal of Autism and Developmental Disorders*, *51*(7), 2369–2380. https://doi.org/10.1007/s10803-020-04707-w

Kalman, J. L., Papiol, S., Forstner, A. J., Heilbronner, U., Degenhardt, F., Strohmaier, J., Adli, M., Adorjan, K., Akula, N., Alda, M., Anderson‐Schmidt, H., Andlauer, T. F., Anghelescu, I., Ardau, R., Arias, B., Arolt, V., Aubry, J., Backlund, L., Bartholdi, K., … Schulze, T. G. (2019). Investigating polygenic burden in age at disease onset in bipolar disorder: Findings from an international multicentric study. *Bipolar Disorders*, *21*(1), 68–75. https://doi.org/10.1111/bdi.12659

Kang, E., Clarkson, T., Keifer, C. M., Rosen, T. E., & Lerner, M. D. (2019). Discrete electrocortical predictors of anxiety and anxiety-related treatment response in youth with autism spectrum disorder. *Biological Psychology*, *146*, 107710. https://doi.org/10.1016/j.biopsycho.2019.05.010

Karavallil Achuthan, S., Coburn, K. L., Beckerson, M. E., & Kana, R. K. (2023). Amplitude of low frequency fluctuations during resting state <scp>fMRI</scp> in autistic children. *Autism Research*, *16*(1), 84–98. https://doi.org/10.1002/aur.2846

Karcher, N. R., & Barch, D. M. (2021). The ABCD study: understanding the development of risk for mental and physical health outcomes. *Neuropsychopharmacology*, *46*(1), 131–142. https://doi.org/10.1038/s41386-020-0736-6

Karlsgodt, K. H., van Erp, T. G. M., Bearden, C. E., & Cannon, T. D. (2014). Altered relationships between age and functional brain activation in adolescents at clinical high risk for psychosis. *Psychiatry Research: Neuroimaging*, *221*(1), 21–29. https://doi.org/10.1016/j.pscychresns.2013.08.004

Karthikeyan, S., Dimick, M. K., Fiksenbaum, L., Jeong, H., Birmaher, B., Kennedy, J. L., Lanctôt, K., Levitt, A. J., Miller, G. E., Schaffer, A., Young, L. T., Youngstrom, E. A., Andreazza, A. C., & Goldstein, B. I. (2022). Inflammatory markers, brain-derived neurotrophic factor, and the symptomatic course of adolescent bipolar disorder: A prospective repeated-measures study. *Brain, Behavior, and Immunity*, *100*, 278–286. https://doi.org/10.1016/j.bbi.2021.11.020

Karthikeyan, S., Fiksenbaum, L., Grigorian, A., Lu, H., MacIntosh, B. J., & Goldstein, B. I. (2019). Normal Cerebral Oxygen Consumption Despite Elevated Cerebral Blood Flow in Adolescents With Bipolar Disorder: Putative Neuroimaging Evidence of Anomalous Energy Metabolism. *Frontiers in Psychiatry*, *10*. https://doi.org/10.3389/fpsyt.2019.00739

Kavanaugh, B. C., Vigne, M. M., Gamble, I., Legere, C., DePamphilis, G., Acuff, W. L., Tirrell, E., Vaughan, N., Thorpe, R., Spirito, A., Jones, S. R., & Carpenter, L. L. (2024). *Dysfunctional oscillatory bursting patterns underlie working memory deficits in adolescents with ADHD*. https://doi.org/10.1101/2024.12.09.627520

Kaymaz, N., Uzun, M. E., Şirin, H., & Kara, Ö. (2025). Evaluation of biochemical markers in relation to psychological well-being in adolescents with polycystic ovary syndrome. *Trends in Pediatrics*, *6*(1), 15–24. https://doi.org/10.59213/TP.2025.155

Keding, T. J., & Herringa, R. J. (2016). Paradoxical Prefrontal–Amygdala Recruitment to Angry and Happy Expressions in Pediatric Posttraumatic Stress Disorder. *Neuropsychopharmacology*, *41*(12), 2903–2912. https://doi.org/10.1038/npp.2016.104

Kehrer-Sawatzki, H., Kluwe, L., Salamon, J., Well, L., Farschtschi, S., Rosenbaum, T., & Mautner, V.-F. (2020). Clinical characterization of children and adolescents with NF1 microdeletions. *Child’s Nervous System*, *36*(10), 2297–2310. https://doi.org/10.1007/s00381-020-04717-0

Kember, J., Stepien, L., Panda, E., & Tekok-Kilic, A. (2023). Resting-state EEG dynamics help explain differences in response control in ADHD: Insight into electrophysiological mechanisms and sex differences. *PLOS ONE*, *18*(10), e0277382. https://doi.org/10.1371/journal.pone.0277382

Kerr, K. L., Ratliff, E. L., Cohen, Z. P., Fuller, S., Cosgrove, K. T., DeVille, D. C., Misaki, M., Morris, A. S., & Bodurka, J. (2022). Real-Time Functional Magnetic Resonance Imaging Dyadic Neurofeedback for Emotion Regulation: A Proof-of-Concept Study. *Frontiers in Human Neuroscience*, *16*. https://doi.org/10.3389/fnhum.2022.910951

Khan, A. J., Nair, A., Keown, C. L., Datko, M. C., Lincoln, A. J., & Müller, R.-A. (2015). Cerebro-cerebellar Resting-State Functional Connectivity in Children and Adolescents with Autism Spectrum Disorder. *Biological Psychiatry*, *78*(9), 625–634. https://doi.org/10.1016/j.biopsych.2015.03.024

Kidman, R., Hossain, S., Hou, W., & Violari, A. (2024). Pathways between adverse childhood experiences and viral suppression among male HIV-infected adolescents in South Africa. *AIDS Care*, *36*(sup1), 36–44. https://doi.org/10.1080/09540121.2024.2332451

Kieling, C., Buchweitz, C., Caye, A., Manfro, P., Pereira, R., Viduani, A., Anés, M., Battel, L., Benetti, S., Fisher, H. L., Karmacharya, R., Kohrt, B. A., Martini, T., Petresco, S., Piccin, J., Rocha, T., Rohde, L. A., Rohrsetzer, F., Souza, L., … Mondelli, V. (2021). The Identifying Depression Early in Adolescence Risk Stratified Cohort (IDEA-RiSCo): Rationale, Methods, and Baseline Characteristics. *Frontiers in Psychiatry*, *12*. https://doi.org/10.3389/fpsyt.2021.697144

Kildal, E. S. M., Quintana, D. S., Szabo, A., Tronstad, C., Andreassen, O., Nærland, T., & Hassel, B. (2023). Heart rate monitoring to detect acute pain in non-verbal patients: a study protocol for a randomized controlled clinical trial. *BMC Psychiatry*, *23*(1), 252. https://doi.org/10.1186/s12888-023-04757-1

Killgore, W. D. S., Gruber, S. A., & Yurgelun-Todd, D. A. (2007). Depressed mood and lateralized prefrontal activity during a Stroop task in adolescent children. *Neuroscience Letters*, *416*(1), 43–48. https://doi.org/10.1016/j.neulet.2007.01.081

Kim, D., Kang, P., Kim, J., Kim, C. Y., Lee, J.-H., Suh, S., & Lee, M.-S. (2019). Machine Learning Classification of First-Onset Drug-Naive MDD Using Structural MRI. *IEEE Access*, *7*, 153977–153985. https://doi.org/10.1109/ACCESS.2019.2949128

Kim, E., Kyeong, S., Cheon, K. A., Park, B., Oh, M. K., Chun, J. W., Park, H. J., Kim, J. J., & Song, D. H. (2016). Neural responses to affective and cognitive theory of mind in children and adolescents with autism spectrum disorder. *Neuroscience Letters*, *621*, 117–125. https://doi.org/10.1016/J.NEULET.2016.04.026

Kim, J., Kim, N., Kim, Y., Park, B., & Park, M. H. (2025). The association between fronto-insular interaction and executive function during childhood and adolescence in a resting state fMRI study. *Scientific Reports*, *15*(1), 30096. https://doi.org/10.1038/s41598-025-14736-z

Kim, J. Y., Minnes, S., Min, M. O., Kim, S. K., Lang, A., Weishampel, P., Short, E. J., Powers, G., & Singer, L. T. (2022). Self-reported mental health outcomes in prenatally cocaine exposed adolescents at 17 years of age. *Neurotoxicology and Teratology*, *94*, 107132. https://doi.org/10.1016/J.NTT.2022.107132

Kim, S., Yoon, H., Shin, J., & Yang, C.-M. (2023). Classification of fNIRS signals from adolescents with MDD in suicide high- and low-risk groups using machine learning. *Journal of Affective Disorders*, *340*, 379–386. https://doi.org/10.1016/j.jad.2023.07.118

Kirkland, A. E., Green, R., Browning, B. D., Aghamoosa, S., Meyerhoff, D. J., Ferguson, P. L., Tomko, R. L., Gray, K. M., & Squeglia, L. M. (2024). Multi-modal neuroimaging reveals differences in alcohol-cue reactivity but not neurometabolite concentrations in adolescents who drink alcohol. *Drug and Alcohol Dependence*, *257*, 111254. https://doi.org/10.1016/j.drugalcdep.2024.111254

Kirshenbaum, J. S., Chahal, R., Ho, T. C., King, L. S., Gifuni, A. J., Mastrovito, D., Coury, S. M., Weisenburger, R. L., & Gotlib, I. H. (2022). Correlates and predictors of the severity of suicidal ideation in adolescence: an examination of brain connectomics and psychosocial characteristics. *Journal of Child Psychology and Psychiatry*, *63*(6), 701–714. https://doi.org/10.1111/jcpp.13512

Kizito, S., Ssewamala, F. M., Nabayinda, J., Namuwonge, F., Neilands, T. B., Nabunya, P., Bahar, O. S., Ssentumbwe, V., & Nattabi, J. (2025). The long-term impact of family economic empowerment on viral suppression and mental health outcomes among adolescents living with HIV in low-income settings: A cluster-randomized controlled trial in Southern Uganda. *Social Science & Medicine*, *364*, 117546. https://doi.org/10.1016/j.socscimed.2024.117546

Klauser, P., Zhou, J., Lim, J. K. W., Poh, J. S., Zheng, H., Tng, H. Y., Krishnan, R., Lee, J., Keefe, R. S. E., Adcock, R. A., Wood, S. J., Fornito, A., & Chee, M. W. L. (2015). Lack of Evidence for Regional Brain Volume or Cortical Thickness Abnormalities in Youths at Clinical High Risk for Psychosis: Findings From the Longitudinal Youth at Risk Study: Table 1. *Schizophrenia Bulletin*, *41*(6), 1285–1293. https://doi.org/10.1093/schbul/sbv012

Klimes-Dougan, B., Eberly, L. E., Westlund Schreiner, M., Kurkiewicz, P., Houri, A., Schlesinger, A., Thomas, K. M., Mueller, B. A., Lim, K. O., & Cullen, K. R. (2014). Multilevel assessment of the neurobiological threat system in depressed adolescents: Interplay between the limbic system and hypothalamic–pituitary–adrenal axis. *Development and Psychopathology*, *26*(4pt2), 1321–1335. https://doi.org/10.1017/S0954579414001059

Klöbl, M., Prillinger, K., Diehm, R., Doganay, K., Lanzenberger, R., Poustka, L., Plener, P., & Konicar, L. (2023). Individual brain regulation as learned via neurofeedback is related to affective changes in adolescents with autism spectrum disorder. *Child and Adolescent Psychiatry and Mental Health*, *17*(1), 6. https://doi.org/10.1186/s13034-022-00549-9

Klosowska, J. C., Verbeken, S., Braet, C., Wijnant, K., Debeuf, T., de Henauw, S., & Michels, N. (2020). The Moderating Role of Emotion Regulation in the Association Between Stressors With Psychological and Biological Measures in Adolescence. *Psychosomatic Medicine*, *82*(5), 495–507. https://doi.org/10.1097/PSY.0000000000000804

Koc, D., Besenek, M., Ulas, G., Ildız, A., Yılmaz, I. T., Guleryuz, H., Guney, S. A., & Emiroglu, N. I. (2020). Investigation of structure-function correlation among the young offspring of patients with bipolar disorder. *Psychiatry Research: Neuroimaging*, *301*, 111103. https://doi.org/10.1016/j.pscychresns.2020.111103

Kochhar, P., Arora, I., Bellato, A., Ropar, D., Hollis, C., & Groom, M. (Maddie) J. (2024). A comparison of visual attention to pictures in the Autism Diagnostic Observation Schedule in children and adolescents with ADHD and/or autism. *Frontiers in Psychiatry*, *15*. https://doi.org/10.3389/fpsyt.2024.1378593

Koenig, J., Thaler, V., Parzer, P., Resch, F., & Kaess, M. (2019). Hair hormones in male youth with internet gaming disorder. *The World Journal of Biological Psychiatry*, *20*(4), 333–338. https://doi.org/10.1080/15622975.2018.1511921

Kondo, D. G., Sung, Y.-H., Hellem, T. L., Delmastro, K. K., Jeong, E.-K., Kim, N., Shi, X., & Renshaw, P. F. (2011). Open-Label Uridine for Treatment of Depressed Adolescents with Bipolar Disorder. *Journal of Child and Adolescent Psychopharmacology*, *21*(2), 171–175. https://doi.org/10.1089/cap.2010.0054

Kong, Y., Liu, C., Zhang, C., Wang, W., Li, Y., Qiu, H., Wang, G., Li, D., Chen, X., Lv, Z., Zhou, D., Wan, L., Ai, M., Chen, J., Ran, L., & Kuang, L. (2022). Association Between Serum Uric Acid Levels and Suicide Attempts in Adolescents and Young Adults with Major Depressive Disorder: A Retrospective Study. *Neuropsychiatric Disease and Treatment*, *Volume 18*, 1469–1477. https://doi.org/10.2147/NDT.S368471

Konicar, L., Radev, S., Prillinger, K., Klöbl, M., Diehm, R., Birbaumer, N., Lanzenberger, R., Plener, P. L., & Poustka, L. (2021). Volitional modification of brain activity in adolescents with Autism Spectrum Disorder: A Bayesian analysis of Slow Cortical Potential neurofeedback. *NeuroImage: Clinical*, *29*, 102557. https://doi.org/10.1016/j.nicl.2021.102557

Korisky, A., Gordon, I., & Goldstein, A. (2022). Oxytocin impacts top-down and bottom-up social perception in adolescents with ASD: a MEG study of neural connectivity. *Molecular Autism*, *13*(1), 36. https://doi.org/10.1186/s13229-022-00513-6

Kribakaran, S., DeCross, S. N., Odriozola, P., McLaughlin, K. A., & Gee, D. G. (2025). Developmental Differences in a Hippocampal–Cingulate Pathway Involved in Learned Safety Following Interpersonal Trauma Exposure. *Journal of the American Academy of Child & Adolescent Psychiatry*, *64*(7), 835–850. https://doi.org/10.1016/j.jaac.2024.07.928

Kruesi, M. J. P., Casanova, M. F., Mannheim, G., & Johnson-Bilder, A. (2004). Reduced temporal lobe volume in early onset conduct disorder. *Psychiatry Research: Neuroimaging*, *132*(1), 1–11. https://doi.org/10.1016/j.pscychresns.2004.07.002

Ku, B. S., Aberizk, K., Addington, J., Bearden, C. E., Cadenhead, K. S., Cannon, T. D., Carrión, R. E., Compton, M. T., Cornblatt, B. A., Druss, B. G., Mathalon, D. H., Perkins, D. O., Tsuang, M. T., Woods, S. W., & Walker, E. F. (2022). The Association Between Neighborhood Poverty and Hippocampal Volume Among Individuals at Clinical High-Risk for Psychosis: The Moderating Role of Social Engagement. *Schizophrenia Bulletin*, *48*(5), 1032–1042. https://doi.org/10.1093/schbul/sbac055

Kujawa, A., Weinberg, A., Bunford, N., Fitzgerald, K. D., Hanna, G. L., Monk, C. S., Kennedy, A. E., Klumpp, H., Hajcak, G., & Phan, K. L. (2016). Error-related brain activity in youth and young adults before and after treatment for generalized or social anxiety disorder. *Progress in Neuro-Psychopharmacology and Biological Psychiatry*, *71*, 162–168. https://doi.org/10.1016/J.PNPBP.2016.07.010

Labrenz, O., Waedel, L., Kölch, M., Lezius, S., Wacker, C., Fröhlich, A., Paschke, K., Thomasius, R., & Reis, O. (2025). Blended digital health intervention for adolescents at high risk with digital media use disorders: protocol for a randomised controlled trial within the Res@t-Consortium. *Frontiers in Psychiatry*, *15*. https://doi.org/10.3389/fpsyt.2024.1478012

Laney, S., & Nooner, K. (2025). Functional brain changes related to adverse childhood experiences and the presence of psychopathology. *Discover Mental Health*, *5*(1), 72. https://doi.org/10.1007/s44192-025-00202-9

Lange, I., Goossens, L., Bakker, J., Michielse, S., van Winkel, R., Lissek, S., Leibold, N., Marcelis, M., Wichers, M., van Os, J., van Amelsvoort, T., & Schruers, K. (2019). Neurobehavioural mechanisms of threat generalization moderate the link between childhood maltreatment and psychopathology in emerging adulthood. *Journal of Psychiatry and Neuroscience*, *44*(3), 185–194. https://doi.org/10.1503/jpn.180053

Laporta-Hoyos, O., Ballester-Plané, J., Póo, P., Macaya, A., Meléndez-Plumed, M., Vázquez, E., Delgado, I., Zubiaurre-Elorza, L., Botellero, V. L., Narberhaus, A., Toro-Tamargo, E., Segarra, D., & Pueyo, R. (2017). Proxy-reported quality of life in adolescents and adults with dyskinetic cerebral palsy is associated with executive functions and cortical thickness. *Quality of Life Research*, *26*(5), 1209–1222. https://doi.org/10.1007/s11136-016-1433-0

Larsen, K. M., Madsen, K. S., ver Loren van Themaat, A. H., Thorup, A. A. E., Plessen, K. J., Mors, O., Nordentoft, M., & Siebner, H. R. (2024). Children at Familial High risk of Schizophrenia and Bipolar Disorder Exhibit Altered Connectivity Patterns During Pre-attentive Processing of an Auditory Prediction Error. *Schizophrenia Bulletin*, *50*(1), 166–176. https://doi.org/10.1093/schbul/sbad092

Latham, R. M., Kieling, C., Arseneault, L., Kohrt, B. A., Moffitt, T. E., Rasmussen, L. J. H., Rocha, T. B.-M., Mondelli, V., & Fisher, H. L. (2022). Longitudinal associations between adolescents’ individualised risk for depression and inflammation in a UK cohort study. *Brain, Behavior, and Immunity*, *101*, 78–83. https://doi.org/10.1016/j.bbi.2021.12.027

Lazarevic, N., Smurthwaite, K. S., Batterham, P. J., Lane, J., Trevenar, S. M., D’Este, C., Clements, A. C. A., Joshy, A. L., Hosking, R., Gad, I., Lal, A., Law, H. di, Banwell, C., Randall, D. A., Miller, A., Housen, T., Korda, R. J., & Kirk, M. D. (2023). Psychological distress in three Australian communities living with environmental per- and polyfluoroalkyl substances contamination. *Science of The Total Environment*, *874*, 162503. https://doi.org/10.1016/J.SCITOTENV.2023.162503

Leahy, A. A., Eather, N., Smith, J. J., Hillman, C., Morgan, P. J., Nilsson, M., Lonsdale, C., Plotnikoff, R. C., Noetel, M., Holliday, E., Shigeta, T. T., Costigan, S. A., Walker, F. R., Young, S., Valkenborghs, S. R., Gyawali, P., Harris, N., Kennedy, S. G., & Lubans, D. R. (2019). School-based physical activity intervention for older adolescents: rationale and study protocol for the Burn 2 Learn cluster randomised controlled trial. *BMJ Open*, *9*(5), e026029. https://doi.org/10.1136/bmjopen-2018-026029

Ledoux, A.-A., Zemek, R., Cairncross, M., Silverberg, N., Sicard, V., Barrowman, N., Goldfield, G., Gray, C., Harris, A. D., Jaworska, N., Reed, N., Saab, B. J., Smith, A., & Walker, L. (2024). Smartphone App–Delivered Mindfulness-Based Intervention for Mild Traumatic Brain Injury in Adolescents: Protocol for a Feasibility Randomized Controlled Trial. *JMIR Research Protocols*, *13*, e57226. https://doi.org/10.2196/57226

Lee, B. L., Gano, D., Rogers, E. E., Xu, D., Cox, S., James Barkovich, A., Li, Y., Ferriero, D. M., & Glass, H. C. (2022). Long-term cognitive outcomes in term newborns with watershed injury caused by neonatal encephalopathy. *Pediatric Research*, *92*(2), 505–512. https://doi.org/10.1038/s41390-021-01526-2

Lee, J. A., Kim, C. K., Jahng, G. H., Hwang, L. K., Cho, Y. W., Kim, Y. J., Lee, W. H., Moon, S. J., Cho, A. R., & Bahn, G. H. (2010). A pilot study of brain activation in children with trichotillomania during a visual-tactile symptom provocation task: A functional magnetic resonance imaging study. *Progress in Neuro-Psychopharmacology and Biological Psychiatry*, *34*(7), 1250–1258. https://doi.org/10.1016/j.pnpbp.2010.06.031

Lee, J., Pavuluri, M. N., Kim, J. H., Suh, S., Kim, I., & Lee, M. S. (2019). Resting-state functional connectivity in medication-naïve adolescents with major depressive disorder. *Psychiatry Research: Neuroimaging*, *288*, 37–43. https://doi.org/10.1016/J.PSCYCHRESNS.2019.04.008

Lee, M.-J., Chou, M.-C., Chou, W.-J., Huang, C.-W., Kuo, H.-C., Lee, S.-Y., & Wang, L.-J. (2018). Heavy Metals’ Effect on Susceptibility to Attention-Deficit/Hyperactivity Disorder: Implication of Lead, Cadmium, and Antimony. *International Journal of Environmental Research and Public Health*, *15*(6), 1221. https://doi.org/10.3390/ijerph15061221

Lee, Y., Yuan, J. P., Winkler, A. M., Kircanski, K., Pine, D. S., & Gotlib, I. H. (2025). Task–Rest Reconfiguration Efficiency of the Reward Network Across Adolescence and Its Association With Early Life Stress and Depressive Symptoms. *Journal of the American Academy of Child & Adolescent Psychiatry*, *64*(2), 290–300. https://doi.org/10.1016/j.jaac.2024.04.018

Lefebvre, A., Traut, N., Pedoux, A., Maruani, A., Beggiato, A., Elmaleh, M., Germanaud, D., Amestoy, A., Ly-Le Moal, M., Chatham, C., Murtagh, L., Bouvard, M., Alisson, M., Leboyer, M., Bourgeron, T., Toro, R., Dumas, G., Moreau, C., & Delorme, R. (2023). Exploring the multidimensional nature of repetitive and restricted behaviors and interests (RRBI) in autism: neuroanatomical correlates and clinical implications. *Molecular Autism*, *14*(1), 45. https://doi.org/10.1186/s13229-023-00576-z

Lei, D., Li, W., Qin, K., Ai, Y., Tallman, M. J., Patino, L. R., Welge, J. A., Blom, T. J., Klein, C. C., Fleck, D. E., Gong, Q., Adler, C. M., Strawn, J. R., Sweeney, J. A., & DelBello, M. P. (2023). Effects of short-term quetiapine and lithium therapy for acute manic or mixed episodes on the limbic system and emotion regulation circuitry in youth with bipolar disorder. *Neuropsychopharmacology*, *48*(4), 615–622. https://doi.org/10.1038/s41386-022-01463-6

Lei, D., Qin, K., Li, W., Pinaya, W. H. L., Tallman, M. J., Patino, L. R., Strawn, J. R., Fleck, D., Klein, C. C., Lui, S., Gong, Q., Adler, C. M., Mechelli, A., Sweeney, J. A., & DelBello, M. P. (2023). Brain morphometric features predict medication response in youth with bipolar disorder: a prospective randomized clinical trial. *Psychological Medicine*, *53*(9), 4083–4093. https://doi.org/10.1017/S0033291722000757

Leibenluft, E., Rich, B. A., Vinton, D. T., Nelson, E. E., Fromm, S. J., Berghorst, L. H., Joshi, P., Robb, A., Schachar, R. J., Dickstein, D. P., McClure, E. B., & Pine, D. S. (2007). Neural Circuitry Engaged During Unsuccessful Motor Inhibition in Pediatric Bipolar Disorder. *American Journal of Psychiatry*, *164*(1), 52–60. https://doi.org/10.1176/ajp.2007.164.1.52

Lennox, B. R., Palmer-Cooper, E. C., Pollak, T., Hainsworth, J., Marks, J., Jacobson, L., Lang, B., Fox, H., Ferry, B., Scoriels, L., Crowley, H., Jones, P. B., Harrison, P. J., & Vincent, A. (2017). Prevalence and clinical characteristics of serum neuronal cell surface antibodies in first-episode psychosis: a case-control study. *The Lancet Psychiatry*, *4*(1), 42–48. https://doi.org/10.1016/S2215-0366(16)30375-3

Lenow, J. K., Scott Steele, J., Smitherman, S., Kilts, C. D., & Cisler, J. M. (2014). Attenuated behavioral and brain responses to trust violations among assaulted adolescent girls. *Psychiatry Research: Neuroimaging*, *223*(1), 1–8. https://doi.org/10.1016/J.PSCYCHRESNS.2014.04.005

Leonards, C. A., Harrison, B. J., Jamieson, A. J., Agathos, J., Steward, T., & Davey, C. G. (2024). Altered task-related decoupling of the rostral anterior cingulate cortex in depression. *NeuroImage: Clinical*, *41*, 103564. https://doi.org/10.1016/j.nicl.2024.103564

Leone, C., Galosi, S., Mollica, C., Fortunato, M., Possidente, C., Milone, V., Misuraca, S., Berillo, L., Truini, A., Cruccu, G., Ferrara, M., & Terrinoni, A. (2021). Dissecting pain processing in adolescents with Non‐Suicidal Self Injury: Could suicide risk lurk among the electrodes? *European Journal of Pain*, *25*(8), 1815–1828. https://doi.org/10.1002/ejp.1793

Leung, R. C., Pang, E. W., Cassel, D., Brian, J. A., Smith, M. lou, & Taylor, M. J. (2015). Early neural activation during facial affect processing in adolescents with Autism Spectrum Disorder. *NeuroImage: Clinical*, *7*, 203–212. https://doi.org/10.1016/j.nicl.2014.11.009

Leung, R. C., Ye, A. X., Wong, S. M., Taylor, M. J., & Doesburg, S. M. (2014). Reduced beta connectivity during emotional face processing in adolescents with autism. *Molecular Autism*, *5*(1), 51. https://doi.org/10.1186/2040-2392-5-51

Levenstein, J. M., Driver, C., Boyes, A., Parker, M., Shan, Z., Lagopoulos, J., & Hermens, D. F. (2023). Sex differences in brain volumes and psychological distress: The first hundred brains cohort of the longitudinal adolescent brain study. *Neuroimage: Reports*, *3*(2), 100167. https://doi.org/10.1016/J.YNIRP.2023.100167

Li, J. C., Hall, M. A., Shalev, I., Schreier, H. M. C., Zarzar, T. G., Marcovici, I., Putnam, F. W., & Noll, J. G. (2021). Hypothalamic-pituitary-adrenal axis attenuation and obesity risk in sexually abused females. *Psychoneuroendocrinology*, *129*, 105254. https://doi.org/10.1016/j.psyneuen.2021.105254

Li, J., Zhang, Y., Yang, N., Du, J., Liu, P., Dai, W., & Dong, Q. (2025). Differences Between Adolescent Depression and Healthy Controls in Biomarkers Associated With Immune or Inflammatory Processes: A Systematic Review and Meta-Analysis. *Psychiatry Investigation*, *22*(2), 119–129. https://doi.org/10.30773/pi.2024.0295

Li, S., Chen, X., Qiu, Y., Teng, Z., Chen, J., Xu, B., Mo, J., Liu, H., Cai, H., & Wu, H. (2025). High allostatic status impairs cognitive function in patients with first-episode drug-naive bipolar disorder. *Progress in Neuro-Psychopharmacology and Biological Psychiatry*, *140*, 111434. https://doi.org/10.1016/j.pnpbp.2025.111434

Li, X., Chen, X., Zhou, Y., Dai, L., Cui, L.-B., Yu, R., Ai, M., Huang, Q., Tian, Y., Ming, M., & Kuang, L. (2022). Altered Regional Homogeneity and Amplitude of Low-Frequency Fluctuations Induced by Electroconvulsive Therapy for Adolescents with Depression and Suicidal Ideation. *Brain Sciences*, *12*(9), 1121. https://doi.org/10.3390/brainsci12091121

Li, X., Huang, Y., Liu, M., Zhang, M., Liu, Y., Teng, T., Liu, X., Yu, Y., Jiang, Y., Ouyang, X., Xu, M., Lv, F., Long, Y., & Zhou, X. (2023). Childhood trauma is linked to abnormal static-dynamic brain topology in adolescents with major depressive disorder. *International Journal of Clinical and Health Psychology*, *23*(4), 100401. https://doi.org/10.1016/j.ijchp.2023.100401

Li, X., & Wang, J. (2021). Abnormal neural activities in adults and youths with major depressive disorder during emotional processing: a meta-analysis. *Brain Imaging and Behavior*, *15*(2), 1134–1154. https://doi.org/10.1007/s11682-020-00299-2

Li, Y., Yuan, M., Zhang, C., Zhang, X., Hao, J., Tao, F., Wang, G., & Su, P. (2024). Bisphenol AF exposure synergistically increases the risk for suicidality among early adolescents with child maltreatment: A prospective cohort study. *Ecotoxicology and Environmental Safety*, *279*, 116511. https://doi.org/10.1016/j.ecoenv.2024.116511

Liang, Y., Shao, R., Xia, Y., Li, Y., & Guo, S. (2024). Investigating amplitude of low-frequency fluctuation and possible links with cognitive impairment in childhood and adolescence onset schizophrenia: a correlation study. *Frontiers in Psychiatry*, *15*. https://doi.org/10.3389/fpsyt.2024.1288955

Lima Santos, J. P., Soehner, A. M., Biernesser, C. L., Ladouceur, C. D., & Versace, A. (2025). Role of Sleep and White Matter in the Link Between Screen Time and Depression in Childhood and Early Adolescence. *JAMA Pediatrics*, *179*(9), 1000. https://doi.org/10.1001/jamapediatrics.2025.1718

Lin, J.-W., Fan, Z.-C., Tzou, S.-C., Wang, L.-J., & Ko, L.-W. (2024). Temporal Alpha Dissimilarity of ADHD Brain Network in Comparison With CPT and CATA. *IEEE Transactions on Neural Systems and Rehabilitation Engineering*, *32*, 1333–1343. https://doi.org/10.1109/TNSRE.2024.3360137

Lin, K., Shao, R., Lu, R., Chen, K., Lu, W., Li, T., Kong, J., So, K.-F., & Xu, G. (2018). Resting-state fMRI signals in offspring of parents with bipolar disorder at the high-risk and ultra-high-risk stages and their relations with cognitive function. *Journal of Psychiatric Research*, *98*, 99–106. https://doi.org/10.1016/j.jpsychires.2018.01.001

Lin, K., Stubbs, B., Zou, W., Zheng, W., Lu, W., Gao, Y., Chen, K., Wang, S., Liu, J., Huang, Y., Guan, L., Wong, M. N. K., Wang, R., Lam, B. Y.-H., & Xu, G. (2020). Aerobic exercise impacts the anterior cingulate cortex in adolescents with subthreshold mood syndromes: a randomized controlled trial study. *Translational Psychiatry*, *10*(1), 155. https://doi.org/10.1038/s41398-020-0840-8

Lin, P.-I., John, J. R., Masi, A., Ong, L. K., Mathew, N. E., Moni, M. A., Eapen, V., & Walker, A. K. (2024). The relationship between hair cortisol concentration and autism diagnosis. *Journal of Psychiatric Research*, *176*, 68–76. https://doi.org/10.1016/j.jpsychires.2024.05.052

Linke, J. O., Jones, E., Pagliaccio, D., Swetlitz, C., Lewis, K. M., Silverman, W. K., Bar-Haim, Y., Pine, D. S., & Brotman, M. A. (2019). Efficacy and mechanisms underlying a gamified attention bias modification training in anxious youth: protocol for a randomized controlled trial. *BMC Psychiatry*, *19*(1), 246. https://doi.org/10.1186/s12888-019-2224-2

Linke, J. O., Stavish, C., Adleman, N. E., Sarlls, J., Towbin, K. E., Leibenluft, E., & Brotman, M. A. (2020). White matter microstructure in youth with and at risk for bipolar disorder. *Bipolar Disorders*, *22*(2), 163–173. https://doi.org/10.1111/bdi.12885

Littler, E. A. L., Butt, Z. A., Gonzalez, A., & Ferro, M. A. (2025). Association Between Hair Cortisol and Psychopathology in Children With a Chronic Physical Illness. *Stress and Health*, *41*(4). https://doi.org/10.1002/smi.70087

Liu, G.-X., Li, Z.-L., Lin, S.-Y., Luo, Z.-Y., Yin, Y.-N., Zhou, Y.-L., & Ning, Y.-P. (2024). NEFA can serve as good biological markers for the diagnosis of depression in adolescents. *Journal of Affective Disorders*, *352*, 342–348. https://doi.org/10.1016/j.jad.2024.01.274

Liu, H., Liu, S., Yu, M., Xin, B., & Liu, Y. (2026). Real-time EEG-triggered transcranial magnetic stimulation combined with group therapy for adolescents with depression and self-harm behavior: A randomized controlled trial. *Journal of Affective Disorders*, *392*, 120144. https://doi.org/10.1016/j.jad.2025.120144

Liu, J., Liu, Q.-R., Wu, Z.-M., Chen, Q.-R., Chen, J., Wang, Y., Cao, X.-L., Dai, M.-X., Dong, C., Liu, Q., Zhu, J., Zhang, L.-L., Li, Y., Wang, Y.-F., Liu, L., & Yang, B.-R. (2023). Specific brain imaging alterations underlying autistic traits in children with attention-deficit/hyperactivity disorder. *Behavioral and Brain Functions*, *19*(1), 20. https://doi.org/10.1186/s12993-023-00222-x

Liu, N., Jia, G., Li, H., Zhang, S., Wang, Y., Niu, H., Liu, L., & Qian, Q. (2022). The potential shared brain functional alterations between adults with ADHD and children with ADHD co-occurred with disruptive behaviors. *Child and Adolescent Psychiatry and Mental Health*, *16*(1), 54. https://doi.org/10.1186/s13034-022-00486-7

Liu, Q., Feng, Y., Chen, W., Zhu, Y., Preece, D. A., Gao, Y., Luo, X., Dang, C., Wang, Y., Sun, L., & Liu, L. (2025). Emotion regulation strategy and its relationship with emotional dysregulation in children with attention-deficit/hyperactivity disorder: behavioral and brain findings. *European Child & Adolescent Psychiatry*, *34*(7), 2241–2252. https://doi.org/10.1007/s00787-025-02643-7

Liu, S., Tang, F., Dou, H., & Zhang, W. (2023). The relationship between autistic traits and empathy in adolescents: An ERP study. *Neuroscience Letters*, *802*, 137173. https://doi.org/10.1016/J.NEULET.2023.137173

Liu, W., Zheng, H., Wen, X., Liu, L., Yang, Y., & Zhong, H. (2025). Associations of pro-inflammatory factors and IL-10 levels with degree of suicide risk in adolescents with depression. *Frontiers in Psychiatry*, *15*. https://doi.org/10.3389/fpsyt.2024.1491555

Liu, X., Chen, Q., Cheng, F., Zhuang, W., Zhang, W., Tang, Y., & Zhou, D. (2024). The abnormal brain activation pattern of adolescents with major depressive disorder based on working memory tasks: A fNIRS study. *Journal of Psychiatric Research*, *169*, 31–37. https://doi.org/10.1016/j.jpsychires.2023.10.054

Liu, Z., Lu, W., Zou, W., Gao, Y., Li, X., Xu, G., So, K.-F., McIntyre, R. S., Lin, K., & Shao, R. (2025). A Preliminary Study of Brain Developmental Features of Bipolar Disorder Familial Risk and Subthreshold Symptoms. *Biological Psychiatry: Cognitive Neuroscience and Neuroimaging*, *10*(7), 769–780. https://doi.org/10.1016/j.bpsc.2024.06.005

Livny, A., & Silberg, T. (2023). Puberty, brain network connectivity and neuropsychiatric outcomes following pediatric traumatic brain injury in females: A research protocol. *PLOS ONE*, *18*(12), e0296325. https://doi.org/10.1371/journal.pone.0296325

Lo, Y., Chou, T., Fan, L., Gau, S. S., Chiu, Y., & Tseng, W. I. (2013). Altered Structure–Function Relations of Semantic Processing in Youths with High‐Functioning Autism: A Combined Diffusion and Functional <scp>MRI</scp> Study. *Autism Research*, *6*(6), 561–570. https://doi.org/10.1002/aur.1315

Loney, B. R., Butler, M. A., Lima, E. N., Counts, C. A., & Eckel, L. A. (2006). The relation between salivary cortisol, callous‐unemotional traits, and conduct problems in an adolescent non‐referred sample. *Journal of Child Psychology and Psychiatry*, *47*(1), 30–36. https://doi.org/10.1111/j.1469-7610.2005.01444.x

Lopes-Júnior, L. C., Pereira-da-Silva, G., Silveira, D. S. C., Veronez, L. C., Santos, J. C., Alonso, J. B., & Lima, R. A. G. (2018). The Effect of Clown Intervention on Self-Report and Biomarker Measures of Stress and Fatigue in Pediatric Osteosarcoma Inpatients: A Pilot Study. *Integrative Cancer Therapies*, *17*(3), 928–940. https://doi.org/10.1177/1534735418781725

Lopez-Larson, M. P., Shah, L. M., Weeks, H. R., King, J. B., Mallik, A. K., Yurgelun-Todd, D. A., & Anderson, J. S. (2017). Abnormal Functional Connectivity Between Default and Salience Networks in Pediatric Bipolar Disorder. *Biological Psychiatry: Cognitive Neuroscience and Neuroimaging*, *2*(1), 85–93. https://doi.org/10.1016/j.bpsc.2016.10.001

Lotspeich, L. J., Kwon, H., Schumann, C. M., Fryer, S. L., Goodlin-Jones, B. L., Buonocore, M. H., Lammers, C. R., Amaral, D. G., & Reiss, A. L. (2004). Investigation of Neuroanatomical Differences Between Autism and AspergerSyndrome. *Archives of General Psychiatry*, *61*(3), 291. https://doi.org/10.1001/archpsyc.61.3.291

Lowet, D. S., Kolan, A., Vaida, F., Hesselink, J. R., Levin, H. S., Ewing-Cobbs, L., Schachar, R. J., Chapman, S. B., Bigler, E. D., Wilde, E. A., Saunders, A. E., Yang, T. T., Tymofiyeva, O., Arif, H., & Max, J. E. (2022). Novel Oppositional Defiant Disorder 6 Months After Traumatic Brain Injury in Children and Adolescents. *The Journal of Neuropsychiatry and Clinical Neurosciences*, *34*(1), 68–76. https://doi.org/10.1176/appi.neuropsych.21020052

Lowet, D. S., Vaida, F., Hesselink, J. R., Ewing-Cobbs, L., Schachar, R. J., Chapman, S. B., Bigler, E. D., Wilde, E. A., Saunders, A. E., Yang, T. T., Tymofiyeva, O., Huang, M., & Max, J. E. (2024). Novel Oppositional Defiant Disorder or Conduct Disorder 24 Months After Traumatic Brain Injury in Children and Adolescents. *The Journal of Neuropsychiatry and Clinical Neurosciences*, *36*(1), 53–62. https://doi.org/10.1176/appi.neuropsych.20220094

Lowet, D. S., Vaida, F., Hesselink, J. R., Levin, H. S., Ewing-Cobbs, L., Schachar, R. J., Chapman, S. B., Bigler, E. D., Wilde, E. A., Saunders, A. E., Yang, T. T., Tymofiyeva, O., & Max, J. E. (2022). Novel Oppositional Defiant Disorder 12 Months After Traumatic Brain Injury in Children and Adolescents. *The Journal of Neuropsychiatry and Clinical Neurosciences*, *34*(2), 149–157. https://doi.org/10.1176/appi.neuropsych.21060149

Lu, Q., Chen, J., Wang, Y., Huang, L., Jiang, Z., Nguchu, B. A., Chen, S., Qiu, B., & Wang, X. (2023). Cerebellar Structural Abnormality in Autism Spectrum Disorder: A Magnetic Resonance Imaging Study. *Psychiatry Investigation*, *20*(4), 334–340. https://doi.org/10.30773/pi.2022.0254

Luby, J. L., Barch, D., Whalen, D., Tillman, R., & Belden, A. (2017). Association Between Early Life Adversity and Risk for Poor Emotional and Physical Health in Adolescence. *JAMA Pediatrics*, *171*(12), 1168. https://doi.org/10.1001/jamapediatrics.2017.3009

Luckhardt, C., Schütz, M., Mühlherr, A., Mössinger, H., Boxhoorn, S., Dempfle, A., Salvador, R., Ruffini, G., Pereira, H. C., Castelo-Branco, M., Latinus, M., Bonnet-Brilhault, F., Siemann, J., Siniatchkin, M., Ecker, C., & Freitag, C. M. (2021). Phase-IIa randomized, double-blind, sham-controlled, parallel group trial on anodal transcranial direct current stimulation (tDCS) over the left and right tempo-parietal junction in autism spectrum disorder—StimAT: study protocol for a clinical trial. *Trials*, *22*(1), 248. https://doi.org/10.1186/s13063-021-05172-1

Lunsford-Avery, J. R., Carskadon, M. A., Kollins, S. H., & Krystal, A. D. (2025). Sleep Physiology and Neurocognition Among Adolescents With Attention-Deficit/Hyperactivity Disorder. *Journal of the American Academy of Child & Adolescent Psychiatry*, *64*(2), 276–289. https://doi.org/10.1016/j.jaac.2024.03.005

Luo, H., Hu, X., Liu, X., Ma, X., Guo, W., Qiu, C., Wang, Y., Wang, Q., Zhang, X., Zhang, W., Hannum, G., Zhang, K., Liu, X., & Li, T. (2012). Hair Cortisol Level as a Biomarker for Altered Hypothalamic-Pituitary-Adrenal Activity in Female Adolescents with Posttraumatic Stress Disorder After the 2008 Wenchuan Earthquake. *Biological Psychiatry*, *72*(1), 65–69. https://doi.org/10.1016/j.biopsych.2011.12.020

Luo, J., Ping, J., Wan, J., Zhu, J., Zhang, Y., Zhang, J., & Jiang, T. (2025). Association of Serum SOCS3 and Inflammatory Marker Levels With Cognitive Function in First‐Episode Schizophrenia. *International Journal of Developmental Neuroscience*, *85*(4). https://doi.org/10.1002/jdn.70027

Luo, Q., Zhang, L., Huang, C.-C., Zheng, Y., Kanen, J. W., Zhao, Q., Yao, Y., Quinlan, E. B., Jia, T., Banaschewski, T., Bokde, A. L. W., Bromberg, U., Büchel, C., Flor, H., Frouin, V., Garavan, H., Gowland, P., Heinz, A., Ittermann, B., … Robbins, T. W. (2020). Association between childhood trauma and risk for obesity: a putative neurocognitive developmental pathway. *BMC Medicine*, *18*(1), 278. https://doi.org/10.1186/s12916-020-01743-2

Luo, S., Lai, S., Liao, X., Wang, Y., Chen, P., Pei, Y., Zhang, J., Zhang, Y., Zhong, S., & Jia, Y. (2025). Neuroimaging biomarkers distinguishing medication-naïve adolescents with major depressive disorder and bipolar disorder during depressive episodes. *Asian Journal of Psychiatry*, *111*, 104660. https://doi.org/10.1016/j.ajp.2025.104660

Luo, Y., Duan, Y., Zhou, Y., Yi, S., Dai, C., Luo, X., Kang, Y., Wan, Z., Qin, N., Zhou, X., Liu, X., Xie, J., & Cheng, A. S. K. (2025). The Level of Psychological Distress Is Associated With Circadian Rhythm, Sleep Quality, and Inflammatory Markers in Adolescent and Young Adults With Gynecological Cancer. *Cancer Nursing*, *48*(5), E289–E295. https://doi.org/10.1097/NCC.0000000000001325

Lynch, C. J., Breeden, A. L., You, X., Ludlum, R., Gaillard, W. D., Kenworthy, L., & Vaidya, C. J. (2017). Executive Dysfunction in Autism Spectrum Disorder Is Associated With a Failure to Modulate Frontoparietal-insular Hub Architecture. *Biological Psychiatry: Cognitive Neuroscience and Neuroimaging*, *2*(6), 537–545. https://doi.org/10.1016/j.bpsc.2017.03.008

Lynch, C. J., Uddin, L. Q., Supekar, K., Khouzam, A., Phillips, J., & Menon, V. (2013). Default Mode Network in Childhood Autism: Posteromedial Cortex Heterogeneity and Relationship with Social Deficits. *Biological Psychiatry*, *74*(3), 212–219. https://doi.org/10.1016/J.BIOPSYCH.2012.12.013

Lyu, H., Jiao, J., Feng, G., Wang, X., Sun, B., Zhao, Z., Shang, D., Pan, F., Xu, W., Duan, J., Zhou, Q., Hu, S., Xu, Y., Xu, D., & Huang, M. (2021). Abnormal causal connectivity of left superior temporal gyrus in drug-naïve first- episode adolescent-onset schizophrenia: A resting-state fMRI study. *Psychiatry Research: Neuroimaging*, *315*, 111330. https://doi.org/10.1016/j.pscychresns.2021.111330

M. Sokhadze, E., & Daniels, R. (2017). Effects of Prefrontal 40 Hz-Centered EEG Band Neurofeedback on Emotional State and Cognitive Functions in Adolescents. *Adolescent Psychiatry*, *6*(2), 116–129. https://doi.org/10.2174/2210676606666161025115616

Ma, H., Zhang, D., Sun, D., Wang, H., & Yang, J. (2022). Gray and white matter structural examination for diagnosis of major depressive disorder and subthreshold depression in adolescents and young adults: a preliminary radiomics analysis. *BMC Medical Imaging*, *22*(1), 164. https://doi.org/10.1186/s12880-022-00892-5

Ma, H., Zhang, D., Wang, Y., Ding, Y., Yang, J., & Li, K. (2023). Prediction of early improvement of major depressive disorder to antidepressant medication in adolescents with radiomics analysis after ComBat harmonization based on multiscale structural MRI. *BMC Psychiatry*, *23*(1), 466. https://doi.org/10.1186/s12888-023-04966-8

Ma, L., Zhou, D.-D., Zhao, L., Hu, J., Peng, X., Jiang, Z., He, X., Wang, W., Hong, S., & Kuang, L. (2023). Impaired behavioral inhibitory control of self-injury cues between adolescents with depression with self-injury behavior and those without during a two-choice oddball task: an event-related potential study. *Frontiers in Psychiatry*, *14*. https://doi.org/10.3389/fpsyt.2023.1165210

Ma, Y., Zhou, H., Li, C., Zou, X., Luo, X., Wu, L., Li, T., Chen, X., Mao, M., Huang, Y., Li, E., An, Y., Zhang, L., Wang, T., Xu, X., Yan, W., Jiang, Y., & Wang, Y. (2021). Differential Metabolites in Chinese Autistic Children: A Multi-Center Study Based on Urinary 1H-NMR Metabolomics Analysis. *Frontiers in Psychiatry*, *12*. https://doi.org/10.3389/fpsyt.2021.624767

mac Giollabhui, N., Alloy, L. B., & Hartman, C. A. (2021). Investigating whether depressed youth exhibiting elevated C reactive protein perform worse on measures of executive functioning, verbal fluency and episodic memory in a large, population based sample of Dutch adolescents. *Brain, Behavior, and Immunity*, *94*, 369–380. https://doi.org/10.1016/j.bbi.2020.08.030

MacIntosh, B. J., Shirzadi, Z., Scavone, A., Metcalfe, A. W., Islam, A. H., Korczak, D., & Goldstein, B. I. (2017). Increased cerebral blood flow among adolescents with bipolar disorder at rest is reduced following acute aerobic exercise. *Journal of Affective Disorders*, *208*, 205–213. https://doi.org/10.1016/j.jad.2016.08.060

Mackin, D. M., Goldstein, B. L., Mumper, E., Kujawa, A., Kessel, E. M., Olino, T. M., Nelson, B. D., Hajcak, G., & Klein, D. N. (2023). Longitudinal Associations Between Reward Responsiveness and Depression Across Adolescence. *Journal of the American Academy of Child & Adolescent Psychiatry*, *62*(7), 816–828. https://doi.org/10.1016/j.jaac.2022.11.013

MacMillan, H. L., Georgiades, K., Duku, E. K., Shea, A., Steiner, M., Niec, A., Tanaka, M., Gensey, S., Spree, S., Vella, E., Walsh, C. A., de Bellis, M. D., van der Meulen, J., Boyle, M. H., & Schmidt, L. A. (2009). Cortisol Response to Stress in Female Youths Exposed to Childhood Maltreatment: Results of the Youth Mood Project. *Biological Psychiatry*, *66*(1), 62–68. https://doi.org/10.1016/j.biopsych.2008.12.014

Mai, C. L., Burns, S., August, D. A., Bhattacharya, S. T., Mueller, A., Houle, T. T., Anderson, T. A., & Peck, J. (2024). Cardiac index as a surrogate marker for anxiety in pediatric patients undergoing ambulatory endoscopy: a prospective cohort study. *Physiological Measurement*, *45*(10), 105003. https://doi.org/10.1088/1361-6579/ad805e

Malhi, G. S., Das, P., Outhred, T., Dobson-Stone, C., Irwin, L., Gessler, D., Bryant, R., & Mannie, Z. (2019). Effect of stress gene-by-environment interactions on hippocampal volumes and cortisol secretion in adolescent girls. *Australian & New Zealand Journal of Psychiatry*, *53*(4), 316–325. https://doi.org/10.1177/0004867419827649

Mamah, D., Mutiso, V., Musyimi, C., Harms, M. P., Anokhin, A. P., Chen, S., Torous, J., Muyela, L., Nashed, J., Al-Hosni, Y., Odera, A., Yarber, A., Golosheykin, S., Faghankhani, M., Sneed, M., & Ndetei, D. M. (2024). Kenya Psychosis-Risk Outcomes Study (KePROS): Development of an Accelerated Medicine Partnership Schizophrenia-Aligned Project in Africa. *Schizophrenia Bulletin Open*, *5*(1). https://doi.org/10.1093/schizbullopen/sgae009

Mamat, M., Chen, Y., Shen, W., & Li, L. (2025). Molecular architecture of the altered cortical complexity in autism. *Molecular Autism*, *16*(1), 1. https://doi.org/10.1186/s13229-024-00632-2

Man, K. K. C., Häge, A., Banaschewski, T., Inglis, S. K., Buitelaar, J., Carucci, S., Danckaerts, M., Dittmann, R. W., Falissard, B., Garas, P., Hollis, C., Konrad, K., Kovshoff, H., Liddle, E., McCarthy, S., Neubert, A., Nagy, P., Rosenthal, E., Sonuga-Barke, E. J. S., … Uebel-von Sandersleben, H. (2023). Long-term safety of methylphenidate in children and adolescents with ADHD: 2-year outcomes of the Attention Deficit Hyperactivity Disorder Drugs Use Chronic Effects (ADDUCE) study. *The Lancet Psychiatry*, *10*(5), 323–333. https://doi.org/10.1016/S2215-0366(23)00042-1

Mandia, D., Plaze, M., le Ber, I., Ewenczyk, C., Morin, A., Carle, G., Consoli, A., Degardin, A., Amad, A., Moreau, C., Anheim, M., Tranchant, C., Mélé, N., Roue-Jagot, C., Lagarde, J., Sarazin, M., Hamelin, L., Ellul, P., Pagan, C., … Nadjar, Y. (2020). High diagnostic value of plasma Niemann-Pick type C biomarkers in adults with selected neurological and/or psychiatric disorders. *Journal of Neurology*, *267*(11), 3371–3377. https://doi.org/10.1007/s00415-020-10020-4

Marsh, A. A., Finger, E. C., Fowler, K. A., Adalio, C. J., Jurkowitz, I. T. N., Schechter, J. C., Pine, D. S., Decety, J., & Blair, R. J. R. (2013). Empathic responsiveness in amygdala and anterior cingulate cortex in youths with psychopathic traits. *Journal of Child Psychology and Psychiatry*, *54*(8), 900–910. https://doi.org/10.1111/jcpp.12063

Martínez, K., Merchán-Naranjo, J., Pina-Camacho, L., Alemán-Gómez, Y., Boada, L., Fraguas, D., Moreno, C., Arango, C., Janssen, J., & Parellada, M. (2017). Atypical age-dependency of executive function and white matter microstructure in children and adolescents with autism spectrum disorders. *European Child & Adolescent Psychiatry*, *26*(11), 1361–1376. https://doi.org/10.1007/s00787-017-0990-2

Masi, A., Breen, E. J., Alvares, G. A., Glozier, N., Hickie, I. B., Hunt, A., Hui, J., Beilby, J., Ravine, D., Wray, J., Whitehouse, A. J. O., & Guastella, A. J. (2017). Cytokine levels and associations with symptom severity in male and female children with autism spectrum disorder. *Molecular Autism*, *8*(1), 63. https://doi.org/10.1186/s13229-017-0176-2

Mathur, A., Bashford-Largo, J., Elowsky, J., Zhang, R., Dobbertin, M., Tyler, P. M., Bajaj, S., Blair, K. S., & Blair, R. J. R. (2023). Association Between Aggression and Differential Functional Activity of Neural Regions Implicated in Retaliation. *Journal of the American Academy of Child & Adolescent Psychiatry*, *62*(7), 805–815. https://doi.org/10.1016/j.jaac.2023.01.021

Matsushima, K., Matsubayashi, J., Toichi, M., Funabiki, Y., Kato, T., Awaya, T., Tsuchida, R., & Kato, T. (2022). Atypical Physiological Response to Less Controllable Sensory Stimulation in Children with ASD. *Research on Child and Adolescent Psychopathology*, *50*(10), 1363–1377. https://doi.org/10.1007/s10802-022-00927-z

Max, J. E., Drake, I., Vaida, F., Hesselink, J. R., Ewing-Cobbs, L., Schachar, R. J., Chapman, S. B., Bigler, E. D., Wilde, E. A., Saunders, A. E., Yang, T. T., Tymofiyeva, O., & Levin, H. S. (2023). Novel Psychiatric Disorder 6 Months After Traumatic Brain Injury in Children and Adolescents. *The Journal of Neuropsychiatry and Clinical Neurosciences*, *35*(2), 141–150. https://doi.org/10.1176/appi.neuropsych.21120301

Max, J. E., Keatley, E., Wilde, E. A., Bigler, E. D., Levin, H. S., Schachar, R. J., Saunders, A., Ewing-Cobbs, L., Chapman, S. B., Dennis, M., & Yang, T. T. (2011). Anxiety Disorders in Children and Adolescents in the First Six Months After Traumatic Brain Injury. *The Journal of Neuropsychiatry and Clinical Neurosciences*, *23*(1), 29–39. https://doi.org/10.1176/jnp.23.1.jnp29

Max, J. E., Keatley, E., Wilde, E. A., Bigler, E. D., Schachar, R. J., Saunders, A. E., Ewing‐Cobbs, L., Chapman, S. B., Dennis, M., Yang, T. T., & Levin, H. S. (2012). Depression in children and adolescents in the first 6 months after traumatic brain injury. *International Journal of Developmental Neuroscience*, *30*(3), 239–245. https://doi.org/10.1016/j.ijdevneu.2011.12.005

Max, J. E., Robin, D. A., Lindgren, S. D., Smith, W. L., Sato, Y., Mattheis, P. J., Stierwalt, J. A. G., & Castillo, C. S. (1997). Traumatic Brain Injury in Children and Adolescents: Psychiatric Disorders at Two Years. *Journal of the American Academy of Child & Adolescent Psychiatry*, *36*(9), 1278–1285. https://doi.org/10.1097/00004583-199709000-00021

Max, J. E., Schachar, R. J., Landis, J., Bigler, E. D., Wilde, E. A., Saunders, A. E., Ewing-Cobbs, L., Chapman, S. B., Dennis, M., Hanten, G., & Levin, H. S. (2013). Psychiatric Disorders in Children and Adolescents in the First Six Months After Mild Traumatic Brain Injury. *The Journal of Neuropsychiatry and Clinical Neurosciences*, *25*(3), 187–197. https://doi.org/10.1176/appi.neuropsych.12010011

MAX, J. E., SMITH, W. L., SATO, Y., MATTHEIS, P. J., CASTILLO, C. S., LINDGREN, S. D., ROBIN, D. A., & STIERWALT, J. A. G. (1997). Traumatic Brain Injury in Children and Adolescents: Psychiatric Disorders in the First Three Months. *Journal of the American Academy of Child & Adolescent Psychiatry*, *36*(1), 94–102. https://doi.org/10.1097/00004583-199701000-00022

May, A. K., Smeeth, D., McEwen, F., Karam, E., & Pluess, M. (2025). Predictors of Environmental Sensitivity in Syrian refugee children. *Journal of Child Psychology and Psychiatry*, *66*(11), 1688–1702. https://doi.org/10.1111/jcpp.14178

May, A. K., Smeeth, D., McEwen, F., Karam, E., Rieder, M. J., Elzagallaai, A. A., van Uum, S., Lionetti, F., & Pluess, M. (2024). The role of environmental sensitivity in the mental health of Syrian refugee children: a multi-level analysis. *Molecular Psychiatry*, *29*(10), 3170–3179. https://doi.org/10.1038/s41380-024-02573-x

McBurnett, K., Raine, A., Stouthamer-Loeber, M., Loeber, R., Kumar, A. M., Kumar, M., & Lahey, B. B. (2005). Mood and Hormone Responses to Psychological Challenge in Adolescent Males with Conduct Problems. *Biological Psychiatry*, *57*(10), 1109–1116. https://doi.org/10.1016/j.biopsych.2005.01.041

McCabe, C., Woffindale, C., Harmer, C. J., & Cowen, P. J. (2012). Neural Processing of Reward and Punishment in Young People at Increased Familial Risk of Depression. *Biological Psychiatry*, *72*(7), 588–594. https://doi.org/10.1016/j.biopsych.2012.04.034

McClure, E. B., Adler, A., Monk, C. S., Cameron, J., Smith, S., Nelson, E. E., Leibenluft, E., Ernst, M., & Pine, D. S. (2007). fMRI predictors of treatment outcome in pediatric anxiety disorders. *Psychopharmacology*, *191*(1), 97–105. https://doi.org/10.1007/s00213-006-0542-9

McGuire, J. L., Kempen, J. H., Localio, R., Ellenberg, J. H., & Douglas, S. D. (2015). Immune Markers Predictive of Neuropsychiatric Symptoms in HIV-Infected Youth. *Clinical and Vaccine Immunology*, *22*(1), 27–36. https://doi.org/10.1128/CVI.00463-14

McKenna, S., Shin, M., Park, S. H., Nichles, A., Zmicerevska, N., Crouse, J., Janiszewski, C., Park, M., Phung, E., Iorfino, F., Varidel, M., Scott, E. M., Carpenter, J. S., & Hickie, I. B. (2025). Identifying mood disorder subgroups at early risk of metabolic dysfunction: a cross-sectional cohort study in young people at early intervention services. *BMJ Open*, *15*(9), e097140. https://doi.org/10.1136/bmjopen-2024-097140

Medda, E., Toccaceli, V., Fagnani, C., Nisticò, L., Brescianini, S., Salemi, M., Ferri, M., D’Ippolito, C., Alviti, S., Arnofi, A., & Stazi, M. A. (2019). The Italian Twin Registry: An Update at 18 Years From Its Inception. *Twin Research and Human Genetics*, *22*(6), 572–578. https://doi.org/10.1017/thg.2019.75

Melamed, I. R., Heffron, M., Testori, A., & Lipe, K. (2018). A pilot study of high‐dose intravenous immunoglobulin 5% for autism: Impact on autism spectrum and markers of neuroinflammation. *Autism Research*, *11*(3), 421–433. https://doi.org/10.1002/aur.1906

Meng, X., Zhang, S., Xu, Y., Ma, Z., Zhou, S., Ma, Y., Ma, H., Yu, X., & Guan, L. (2025). Exosomal microRNA signatures in youth at clinical high risk for bipolar disorder. *Frontiers in Psychiatry*, *16*. https://doi.org/10.3389/fpsyt.2025.1589374

Meossi, C., Carrer, A., Ciaccio, C., Estienne, M., Silipigni, R., Sciacca, F. L., Pantaleoni, C., D’Arrigo, S., & Milani, D. (2023). Clinical features and magnesium levels: Novel insights in 15q11.2 BP1–BP2 copy number variants. *Journal of Intellectual Disability Research*, *67*(7), 679–689. https://doi.org/10.1111/jir.13038

Metcalfe, A. W. S., MacIntosh, B. J., Scavone, A., Ou, X., Korczak, D., & Goldstein, B. I. (2016). Effects of acute aerobic exercise on neural correlates of attention and inhibition in adolescents with bipolar disorder. *Translational Psychiatry*, *6*(5), e814–e814. https://doi.org/10.1038/tp.2016.85

Metzak, P. D., Addington, J., Hassel, S., Goldstein, B. I., MacIntosh, B. J., Lebel, C., Wang, J. L., Kennedy, S. H., MacQueen, G. M., & Bray, S. (2021). Functional imaging in youth at risk for transdiagnostic serious mental illness: Initial results from the <scp>PROCAN</scp> study. *Early Intervention in Psychiatry*, *15*(5), 1276–1291. https://doi.org/10.1111/eip.13078

Meyer, A., Weinberg, A., Klein, D. N., & Hajcak, G. (2012). The development of the error-related negativity (ERN) and its relationship with anxiety: Evidence from 8 to 13 year-olds. *Developmental Cognitive Neuroscience*, *2*(1), 152–161. https://doi.org/10.1016/J.DCN.2011.09.005

Miano, S., Esposito, M., Foderaro, G., Ramelli, G. P., Pezzoli, V., & Manconi, M. (2016). Sleep‐Related Disorders in Children with Attention‐Deficit Hyperactivity Disorder: Preliminary Results of a Full Sleep Assessment Study. *CNS Neuroscience & Therapeutics*, *22*(11), 906–914. https://doi.org/10.1111/cns.12573

Michalska, K. J., Feldman, J. S., Ivie, E. J., Shechner, T., Sequeira, S., Averbeck, B., Degnan, K. A., Chronis-Tuscano, A., Leibenluft, E., Fox, N. A., & Pine, D. S. (2019). Early-childhood social reticence predicts SCR-BOLD coupling during fear extinction recall in preadolescent youth. *Developmental Cognitive Neuroscience*, *36*, 100605. https://doi.org/10.1016/J.DCN.2018.12.003

Miklowitz, D. J., Schneck, C. D., Walshaw, P. D., Garrett, A. S., Singh, M. K., Sugar, C. A., & Chang, K. D. (2019). Early intervention for youth at high risk for bipolar disorder: A multisite randomized trial of family‐focused treatment. *Early Intervention in Psychiatry*, *13*(2), 208–216. https://doi.org/10.1111/eip.12463

Miller, A. B., McLaughlin, K. A., Busso, D. S., Brueck, S., Peverill, M., & Sheridan, M. A. (2018). Neural Correlates of Emotion Regulation and Adolescent Suicidal Ideation. *Biological Psychiatry: Cognitive Neuroscience and Neuroimaging*, *3*(2), 125–132. https://doi.org/10.1016/j.bpsc.2017.08.008

Modabbernia, A., Reichenberg, A., Ing, A., Moser, D. A., Doucet, G. E., Artiges, E., Banaschewski, T., Barker, G. J., Becker, A., Bokde, A. L. W., Quinlan, E. B., Desrivières, S., Flor, H., Fröhner, J. H., Garavan, H., Gowland, P., Grigis, A., Grimmer, Y., Heinz, A., … Frangou, S. (2021). Linked patterns of biological and environmental covariation with brain structure in adolescence: a population-based longitudinal study. *Molecular Psychiatry*, *26*(9), 4905–4918. https://doi.org/10.1038/s41380-020-0757-x

Modarelli, R. E., Molsberry, S. A., Malave-Ortiz, S., Calvert, M., Lucien, J., Denslow, S., Zaccaro, D., Kamoun, C., & Shaw, N. D. (2025). Natural History of Menstrual Pain and Associated Risk Factors in Early Adolescence. *Journal of Pediatric and Adolescent Gynecology*, *38*(1), 52–59. https://doi.org/10.1016/j.jpag.2024.10.006

Molina-Carballo, A., Cubero-Millán, I., Fernández-López, L., Checa-Ros, A., Machado-Casas, I., Jerez-Calero, A., Blanca-Jover, E., Cantarero-Malagón, A.-M., Uberos, J., & Muñoz-Hoyos, A. (2021). Methylphenidate ameliorates the homeostatic balance between levels of kynurenines in ADHD children. *Psychiatry Research*, *303*, 114060. https://doi.org/10.1016/j.psychres.2021.114060

Molina-Carballo, A., Naranjo-Gómez, A., Uberos, J., Justicia-Martínez, F., Ruiz-Ramos, M.-J., Cubero-Millán, I., Contreras-Chova, F., Augustin-Morales, M.-C., Khaldy-Belkadi, H., & Muñoz-Hoyos, A. (2013). Methylphenidate effects on blood serotonin and melatonin levels may help to synchronise biological rhythms in children with ADHD. *Journal of Psychiatric Research*, *47*(3), 377–383. https://doi.org/10.1016/j.jpsychires.2012.09.020

Molteni, S., Filosi, E., Mensi, M. M., Spada, G., Zandrini, C., Ferro, F., Paoletti, M., Pichiecchio, A., Bonoldi, I., & Balottin, U. (2019). Predictors of Outcomes in Adolescents With Clinical High Risk for Psychosis, Other Psychiatric Symptoms, and Psychosis: A Longitudinal Protocol Study. *Frontiers in Psychiatry*, *10*. https://doi.org/10.3389/fpsyt.2019.00787

Monaco, F., Vignapiano, A., Piacente, M., Farina, F., Pagano, C., Marenna, A., Leo, S., Vecchi, C., Mancuso, C., Prisco, V., Iodice, D., Auricchio, A., Cavaliere, R., D’Agosto, A., Fornaro, M., Solmi, M., Corrivetti, G., & Fasano, A. (2024). Innova4Health: an integrated approach for prevention of recurrence and personalized treatment of Major Depressive Disorder. *Frontiers in Artificial Intelligence*, *7*. https://doi.org/10.3389/frai.2024.1366055

Mooney, M. A., Bhatt, P., Hermosillo, R. J. M., Ryabinin, P., Nikolas, M., Faraone, S. v., Fair, D. A., Wilmot, B., & Nigg, J. T. (2021). Smaller total brain volume but not subcortical structure volume related to common genetic risk for ADHD. *Psychological Medicine*, *51*(8), 1279–1288. https://doi.org/10.1017/S0033291719004148

Moore, A., Lewis, B., Elton, A., Squeglia, L. M., & Nixon, S. J. (2024). An investigation of multimodal predictors of adolescent alcohol initiation. *Drug and Alcohol Dependence*, *265*, 112491. https://doi.org/10.1016/j.drugalcdep.2024.112491

Moore, S. K., Marsch, L. A., Badger, G. J., Solhkhah, R., & Hofstein, Y. (2011). Improvement in Psychopathology Among Opioid-dependent Adolescents During Behavioral-pharmacological Treatment. *Journal of Addiction Medicine*, *5*(4), 264–271. https://doi.org/10.1097/ADM.0b013e3182191099

Morath, J., Gola, H., Sommershof, A., Hamuni, G., Kolassa, S., Catani, C., Adenauer, H., Ruf-Leuschner, M., Schauer, M., Elbert, T., Groettrup, M., & Kolassa, I.-T. (2014). The effect of trauma-focused therapy on the altered T cell distribution in individuals with PTSD: Evidence from a randomized controlled trial. *Journal of Psychiatric Research*, *54*, 1–10. https://doi.org/10.1016/j.jpsychires.2014.03.016

Morcel, J., Béghin, L., Michels, N., Vanhelst, J., Labreuche, J., Drumez, E., Polito, A., Ferrari, M., Censi, L., Deplanque, D., Miguel-Berges, M. L., de Ruyter, T., de Henauw, S., Moreno, L. A., & Gottrand, F. (2022). Identification of Lifestyle Risk Factors in Adolescence Influencing Cardiovascular Health in Young Adults: The BELINDA Study. *Nutrients*, *14*(10), 2089. https://doi.org/10.3390/nu14102089

Morfini, F., Kucyi, A., Zhang, J., Bauer, C. C. C., Bloom, P. A., Pagliaccio, D., Hubbard, N. A., Rosso, I. M., Yendiki, A., Ghosh, S. S., Pizzagalli, D. A., Gabrieli, J. D. E., Whitfield-Gabrieli, S., & Auerbach, R. P. (2025). Brain functional connectivity predicts depression and anxiety during childhood and adolescence: A connectome-based predictive modeling approach. *Imaging Neuroscience*, *3*. https://doi.org/10.1162/IMAG.a.145

Mortazavi, R., Lalouni, M., Grudin, R., Serlachius, E., Sundberg, C. J., Norrbom, J., Larsson, I., Haglund, E., Ivarsson, A., Lenhard, F., Cronqvist, T., Ingemarsson, K., Mårsell, Å., Rask, O., & Jarbin, H. (2022). Moderate-to-vigorous group aerobic exercise versus group leisure activities for mild-to-moderate depression in adolescents: study protocol for a multicentre randomised controlled trial. *BMJ Open*, *12*(7), e060159. https://doi.org/10.1136/bmjopen-2021-060159

Mouga, S., Duarte, I. C., Café, C., Sousa, D., Duque, F., Oliveira, G., & Castelo-Branco, M. (2022). Parahippocampal deactivation and hyperactivation of central executive, saliency and social cognition networks in autism spectrum disorder. *Journal of Neurodevelopmental Disorders*, *14*(1), 9. https://doi.org/10.1186/s11689-022-09417-1

Mueller, S. C., Maheu, F. S., Dozier, M., Peloso, E., Mandell, D., Leibenluft, E., Pine, D. S., & Ernst, M. (2010). Early-life stress is associated with impairment in cognitive control in adolescence: An fMRI study. *Neuropsychologia*, *48*(10), 3037–3044. https://doi.org/10.1016/j.neuropsychologia.2010.06.013

Muglia, P., Filosi, M., da Ros, L., Kam-Thong, T., Nardocci, F., Trabetti, E., Ratti, E., Rizzini, P., Zuddas, A., Bernardina, B. D., & Domenici, E. (2018). The Italian autism network (ITAN): a resource for molecular genetics and biomarker investigations. *BMC Psychiatry*, *18*(1), 369. https://doi.org/10.1186/s12888-018-1937-y

Murage, D., Nazarova, A., Drobinin, V., Adepalli, N., Helmick, C. A., Schmidt, M. H., Newman, A. J., Bowen, C. v., & Uher, R. (2025). Familial risk of major mood disorders and brain functional connectivity in the default mode, cognitive executive, and salience networks. *Journal of Psychiatry and Neuroscience*, *50*(3), E181–E193. https://doi.org/10.1503/jpn.250002

Mürner-Lavanchy, I., Josi, J., Koenig, J., Reichl, C., Brunner, R., & Kaess, M. (2023). Resting-state functional connectivity predicting clinical improvement following treatment in female adolescents with non-suicidal self-injury. *Journal of Affective Disorders*, *327*, 79–86. https://doi.org/10.1016/J.JAD.2023.01.117

Murray, L., Israel, E. S., Balkind, E. G., Pastro, B., Lovell-Smith, N., Lukas, S. E., Forbes, E. E., Pizzagalli, D. A., & Webb, C. A. (2023). Multi-modal assessment of reward functioning in adolescent anhedonia. *Psychological Medicine*, *53*(10), 4424–4433. https://doi.org/10.1017/S0033291722001222

Muscatello, R. A., Cola, M., Vandekar, S., & Corbett, B. A. (2025). Pubertal developmental, body mass index, and cardiovascular autonomic function in children and adolescents with and without autism spectrum disorder: a four-time point accelerated longitudinal study. *Journal of Neurodevelopmental Disorders*, *17*(1), 14. https://doi.org/10.1186/s11689-025-09602-y

Nabwera, H. M., Shah, V., Neville, R., Sosseh, F., Saidykhan, M., Faal, F., Sonko, B., Keita, O., Schmidt, W.-P., & Torondel, B. (2021). Menstrual hygiene management practices and associated health outcomes among school-going adolescents in rural Gambia. *PLOS ONE*, *16*(2), e0247554. https://doi.org/10.1371/journal.pone.0247554

Nagamitsu, S., Sakurai, R., Matsuoka, M., Chiba, H., Ozono, S., Tanigawa, H., Yamashita, Y., Kaida, H., Ishibashi, M., Kakuma, T., Croarkin, P. E., & Matsuishi, T. (2016). Altered SPECT 123I-iomazenil Binding in the Cingulate Cortex of Children with Anorexia Nervosa. *Frontiers in Psychiatry*, *7*. https://doi.org/10.3389/fpsyt.2016.00016

Nagashima, M., Monden, Y., Dan, I., Dan, H., Mizutani, T., Tsuzuki, D., Kyutoku, Y., Gunji, Y., Hirano, D., Taniguchi, T., Shimoizumi, H., Momoi, M. Y., Yamagata, T., & Watanabe, E. (2014). Neuropharmacological effect of atomoxetine on attention network in children with attention deficit hyperactivity disorder during oddball paradigms as assessed using functional near-infrared spectroscopy. *Neurophotonics*, *1*(2), 025007. https://doi.org/10.1117/1.NPh.1.2.025007

Nagy, M. R., Gill, A., Adams, T., Gerras, J., Mazin, L., Leung, C., & Hasson, R. E. (2019). Stress-Induced Suppression of Food Intake in Overweight and Obese Adolescents. *Psychosomatic Medicine*, *81*(9), 814–820. https://doi.org/10.1097/PSY.0000000000000732

Naim, R., Kircanski, K., Gold, A., German, R. E., Davis, M., Perlstein, S., Clayton, M., Revzina, O., & Brotman, M. A. (2021). Across-subjects multiple baseline trial of exposure-based cognitive-behavioral therapy for severe irritability: a study protocol. *BMJ Open*, *11*(3), e039169. https://doi.org/10.1136/bmjopen-2020-039169

Naranjo-Galvis, C. A., Trejos-Gallego, D. M., Correa-Salazar, C., Triviño-Valencia, J., Valencia-Buitrago, M., Ruiz-Pulecio, A. F., Méndez-Ramírez, L. F., Zabaleta, J., Meñaca-Puentes, M. A., Ruiz-Villa, C. A., Orjuela-Rodriguez, M., Carmona-Hernández, J. C., & Salamanca-Duque, L. M. (2025). Anti-Inflammatory Diet and Probiotic Supplementation as Strategies to Modulate Immune Dysregulation in Autism Spectrum Disorder. *Nutrients*, *17*(16), 2664. https://doi.org/10.3390/nu17162664

Nasstasia, Y., Baker, A. L., Halpin, S. A., Hides, L., Lewin, T. J., Kelly, B. J., & Callister, R. (2018). Evaluating the efficacy of an integrated motivational interviewing and multi-modal exercise intervention for youth with major depression: Healthy Body, Healthy Mind randomised controlled trial protocol. *Contemporary Clinical Trials Communications*, *9*, 13–22. https://doi.org/10.1016/J.CONCTC.2017.11.007

Naumann, S., Senftleben, U., Santhosh, M., McPartland, J., & Webb, S. J. (2018). Neurophysiological correlates of holistic face processing in adolescents with and without autism spectrum disorder. *Journal of Neurodevelopmental Disorders*, *10*(1), 27. https://doi.org/10.1186/s11689-018-9244-y

Nelson, E. A., Kraguljac, N. v., Bashir, A., Cofield, S. S., Maximo, J. O., Armstrong, W., & Lahti, A. C. (2025). A longitudinal study of hippocampal subfield volumes and hippocampal glutamate levels in antipsychotic-naïve first episode psychosis patients. *Molecular Psychiatry*, *30*(5), 2017–2026. https://doi.org/10.1038/s41380-024-02812-1

Nerland, S., Barth, C., Jørgensen, K. N., Wortinger, L. A., Castro-Fornieles, J., Díaz-Caneja, C. M., Janssen, J., Arango, C., Sugranyes, G., Baeza, I., Piras, F., Banaj, N., Piras, F., Elia, S., Vecchio, D., Lundberg, M., Bohman, H., Rund, B. R., de la Serna, E., … Agartz, I. (2025). *Cerebral cortical alterations in adolescent early-onset psychosis: a surface-based morphometry mega-analysis*. https://doi.org/10.1101/2025.05.24.25328176

Nguyen, C. B., Alsøe, L., Lindvall, J. M., Sulheim, D., Fagermoen, E., Winger, A., Kaarbø, M., Nilsen, H., & Wyller, V. B. (2017). Whole blood gene expression in adolescent chronic fatigue syndrome: an exploratory cross-sectional study suggesting altered B cell differentiation and survival. *Journal of Translational Medicine*, *15*(1), 102. https://doi.org/10.1186/s12967-017-1201-0

Nielsen, J. D., Case, J. A. C., Divers, R. M., Kautz, M. M., Alloy, L. B., & Olino, T. M. (2020). Trajectories of depressive symptoms through adolescence as predictors of cortical thickness in the orbitofrontal cortex: An examination of sex differences. *Psychiatry Research: Neuroimaging*, *303*, 111132. https://doi.org/10.1016/J.PSCYCHRESNS.2020.111132

Nielsen, N. O., Rafn, A. V., Andersen, S., Rafn, J., Kjærsgaard, H., Petersen, B., Sønderskov, A. J., Dahl, M., Benedikz, E., & Lindahl, M. (2025). Effect of a multi-component, participatory social and health promoting intervention on health and wellbeing in refugee families from Syria resettled in Denmark. *Journal of Migration and Health*, *12*, 100338. https://doi.org/10.1016/j.jmh.2025.100338

Niendam, T. A., Ray, K. L., Iosif, A.-M., Lesh, T. A., Ashby, S. R., Patel, P. K., Smucny, J., Ferrer, E., Solomon, M., Ragland, J. D., & Carter, C. S. (2018). Association of Age at Onset and Longitudinal Course of Prefrontal Function in Youth With Schizophrenia. *JAMA Psychiatry*, *75*(12), 1252. https://doi.org/10.1001/jamapsychiatry.2018.2538

Noll, J. G., Zeller, M. H., Trickett, P. K., & Putnam, F. W. (2007). Obesity Risk for Female Victims of Childhood Sexual Abuse: A Prospective Study. *Pediatrics*, *120*(1), e61–e67. https://doi.org/10.1542/peds.2006-3058

Norman, A. L., Pulido, C., Squeglia, L. M., Spadoni, A. D., Paulus, M. P., & Tapert, S. F. (2011). Neural activation during inhibition predicts initiation of substance use in adolescence. *Drug and Alcohol Dependence*, *119*(3), 216–223. https://doi.org/10.1016/j.drugalcdep.2011.06.019

Nugent, N. R., Armey, M., Boker, S., Brick, L., Knopik, V., McGeary, J. E., Spirito, A., & Mehl, M. R. (2022). Adolescents hospitalised for suicidality: biomarkers, social and affective predictors: a cohort study. *BMJ Open*, *12*(10), e056063. https://doi.org/10.1136/bmjopen-2021-056063

Oberman, L. M., Francis, S. M., Beynel, L., Hynd, M., Jaime, M., Robins, P. L., Deng, Z.-D., Stout, J., van der Veen, J. W., & Lisanby, S. H. (2024). Design and methodology for a proof of mechanism study of individualized neuronavigated continuous Theta burst stimulation for auditory processing in adolescents with autism spectrum disorder. *Frontiers in Psychiatry*, *15*. https://doi.org/10.3389/fpsyt.2024.1304528

O’Donoghue, B., Francey, S. M., Nelson, B., Ratheesh, A., Allott, K., Graham, J., Baldwin, L., Alvarez‐Jimenez, M., Thompson, A., Fornito, A., Polari, A., Berk, M., Macneil, C., Crisp, K., Pantelis, C., Yuen, H. P., Harrigan, S., & McGorry, P. (2019). Staged treatment and acceptability guidelines in early psychosis study (STAGES): A randomized placebo controlled trial of intensive psychosocial treatment plus or minus antipsychotic medication for first‐episode psychosis with low‐risk of self‐harm or aggression. Study protocol and baseline characteristics of participants. *Early Intervention in Psychiatry*, *13*(4), 953–960. https://doi.org/10.1111/eip.12716

Okada, M., Pehlivan, M. J., Miskovic-Wheatley, J., Barakat, S., Griffiths, K. R., Touyz, S. W., Simpson, S. J., Maguire, S., & Holmes, A. J. (2023). My Diet Study: protocol for a two-part observational, longitudinal, psycho-biological study of dieting in Australian youth. *Frontiers in Public Health*, *11*. https://doi.org/10.3389/fpubh.2023.1281855

Okada, N., Ando, S., Sanada, M., Hirata‐Mogi, S., Iijima, Y., Sugiyama, H., Shirakawa, T., Yamagishi, M., Kanehara, A., Morita, M., Yagi, T., Hayashi, N., Koshiyama, D., Morita, K., Sawada, K., Ikegame, T., Sugimoto, N., Toriyama, R., Masaoka, M., … Kasai, K. (2019). Population‐neuroscience study of the Tokyo TEEN Cohort (pn‐TTC): Cohort longitudinal study to explore the neurobiological substrates of adolescent psychological and behavioral development. *Psychiatry and Clinical Neurosciences*, *73*(5), 231–242. https://doi.org/10.1111/pcn.12814

Oluyemi, K., Rechtman, E., Invernizzi, A., Gennings, C., Renzetti, S., Patrono, A., Cagna, G., Reichenberg, A., Smith, D. R., Lucchini, R. G., Wright, R. O., Placidi, D., & Horton, M. K. (2024). Sex-specific associations between co-exposure to multiple metals and externalizing symptoms in adolescence and young adulthood. *Environmental Research*, *250*, 118443. https://doi.org/10.1016/j.envres.2024.118443

O’Neill, J., Joshi, S., Alger, J. R., Leuchter, A. F., Schneider, B. N., & O’Connor, M. J. (2025). Trigeminal nerve stimulation (TNS) for children with attention deficit/hyperactivity disorder and fetal alcohol spectrum disorder: Feasibility study protocol. *PLOS One*, *20*(8), e0330986. https://doi.org/10.1371/journal.pone.0330986

Ono, Y., Kikuchi, M., Nakatani, H., Murakami, M., Nishisaka, M., Matsumura, T., Munesue, T., & Minabe, Y. (2017). Prefrontal oxygenation during verbal fluency and cognitive function in adolescents with bipolar disorder type II. *Asian Journal of Psychiatry*, *25*, 147–153. https://doi.org/10.1016/J.AJP.2016.11.001

ORTIZ, J., & RAINE, A. (2004). Heart Rate Level and Antisocial Behavior in Children and Adolescents: A Meta-Analysis. *Journal of the American Academy of Child & Adolescent Psychiatry*, *43*(2), 154–162. https://doi.org/10.1097/00004583-200402000-00010

Oshodi, Y., Ojewunmi, O., Oshodi, T., Ijarogbe, G., Ogun, O., Aina, O., & Lesi, F. (2017). Oxidative stress markers and genetic polymorphisms of glutathione S-transferase T1, M1, and P1 in a subset of children with autism spectrum disorder in Lagos, Nigeria. *Nigerian Journal of Clinical Practice*, *20*(9), 1161. https://doi.org/10.4103/njcp.njcp_282_16

Osika, W., Friberg, P., & Wahrborg, P. (2007). A new short self-rating questionnaire to assess stress in children. *International Journal of Behavioral Medicine*, *14*(2), 108–117. https://doi.org/10.1007/BF03004176

Ostrover, R., Freed, R. D., Stadterman, J., Case, J. A., & Gabbay, V. (2016). 5.31 ANTERIOR CINGULATE CORTEX GAMMA-AMINOBUTYRIC ACID IN DEPRESSED ADOLESCENTS: A RELATIONSHIP TO ANHEDONIA. *Journal of the American Academy of Child & Adolescent Psychiatry*, *55*(10), S193–S194. https://doi.org/10.1016/j.jaac.2016.09.290

Ouisselsat, M., Maidoumi, S., Elmaouaki, A., Lekouch, N., Pineau, A., & Sedki, A. (2023). Hair Trace Elements and Mineral Content in Moroccan Children with Autism Spectrum Disorder: a Case–Control Study. *Biological Trace Element Research*, *201*(6), 2701–2710. https://doi.org/10.1007/s12011-022-03365-6

Oyama, N., Vaneynde, P., Reynhout, S., Pao, E. M., Timms, A., Fan, X., Foss, K., Derua, R., Janssens, V., Chung, W., & Mirzaa, G. M. (2023). Clinical, neuroimaging and molecular characteristics of *PPP2R5D* -related neurodevelopmental disorders: an expanded series with functional characterisation and genotype–phenotype analysis. *Journal of Medical Genetics*, *60*(5), 511–522. https://doi.org/10.1136/jmg-2022-108713

Özel, F., Indremo, M., Karamanis, G., Elofsson, U., Beckman, U., Fazekas, A., Frisén, L., Isaksson, M., Sandström, L., Thelin, N., Tivesten, Å., Wahlberg, J., Skalkidou, A., Bodlund, O., & Papadopoulos, F. C. (2023). Exploring gender dysphoria and related outcomes in a prospective cohort study: protocol for the Swedish Gender Dysphoria Study (SKDS). *BMJ Open*, *13*(4), e066571. https://doi.org/10.1136/bmjopen-2022-066571

Ozger, C., Chumachenko, S., McVoy, M., Croarkin, P. E., & Doruk Camsari, D. (2023). Evidence for Altered Electroencephalography Coherence in Depressed Adolescents with Suicidal Ideation and Behaviors. *Journal of Child and Adolescent Psychopharmacology*, *33*(7), 287–293. https://doi.org/10.1089/cap.2023.0033

Oztop, D., Altun, H., Baskol, G., & Ozsoy, S. (2012). Oxidative stress in children with attention deficit hyperactivity disorder. *Clinical Biochemistry*, *45*(10–11), 745–748. https://doi.org/10.1016/J.CLINBIOCHEM.2012.03.027

Padmanabhan, A., Garver, K., O’Hearn, K., Nawarawong, N., Liu, R., Minshew, N., Sweeney, J., & Luna, B. (2015). Developmental Changes in Brain Function Underlying Inhibitory Control in Autism Spectrum Disorders. *Autism Research*, *8*(2), 123–135. https://doi.org/10.1002/aur.1398

Paillère Martinot, M.-L., Lemaitre, H., Artiges, E., Miranda, R., Goodman, R., Penttilä, J., Struve, M., Fadai, T., Kappel, V., Poustka, L., Conrod, P., Banaschewski, T., Barbot, A., Barker, G. J., Büchel, C., Flor, H., Gallinat, J., Garavan, H., Heinz, A., … Martinot, J.-L. (2014). White-matter microstructure and gray-matter volumes in adolescents with subthreshold bipolar symptoms. *Molecular Psychiatry*, *19*(4), 462–470. https://doi.org/10.1038/mp.2013.44

Pan, L. A., Batezati-Alves, S. C., Almeida, J. R. C., Segreti, A., Akkal, D., Hassel, S., Lakdawala, S., Brent, D. A., & Phillips, M. L. (2011). Dissociable Patterns of Neural Activity During Response Inhibition in Depressed Adolescents With and Without Suicidal Behavior. *Journal of the American Academy of Child & Adolescent Psychiatry*, *50*(6), 602-611.e3. https://doi.org/10.1016/j.jaac.2011.03.018

Pan, L. A., Hassel, S., Segreti, A. M., Nau, S. A., Brent, D. A., & Phillips, M. L. (2013). Differential patterns of activity and functional connectivity in emotion processing neural circuitry to angry and happy faces in adolescents with and without suicide attempt. *Psychological Medicine*, *43*(10), 2129–2142. https://doi.org/10.1017/S0033291712002966

Panagiotou, E., Agouropoulos, A., Vadiakas, G., Pervanidou, P., Chouliaras, G., & Kanaka-Gantenbein, C. (2021). Oral health of overweight and obese children and adolescents: a comparative study with a multivariate analysis of risk indicators. *European Archives of Paediatric Dentistry*, *22*(5), 861–868. https://doi.org/10.1007/s40368-021-00643-0

Panda, P. K., Badal, S., Sirolia, V., Sharawat, I. K., Chakrabarty, B., Jauhari, P., & Gulati, S. (2024). Efficacy of verbal suggestion, verbal suggestion with a tuning fork, and verbal suggestion with a cotton swab for inducing the paroxysmal event during video-EEG recording in children with suspected psychogenic nonepileptic seizures. *Epilepsy & Behavior*, *156*, 109818. https://doi.org/10.1016/j.yebeh.2024.109818

Pannekoek, J. N., van der Werff, S. J. A., Meens, P. H. F., van den Bulk, B. G., Jolles, D. D., Veer, I. M., van Lang, N. D. J., Rombouts, S. A. R. B., van der Wee, N. J. A., & Vermeiren, R. R. J. M. (2014). Aberrant resting‐state functional connectivity in limbic and salience networks in treatment‐naïve clinically depressed adolescents. *Journal of Child Psychology and Psychiatry*, *55*(12), 1317–1327. https://doi.org/10.1111/jcpp.12266

Papanastasiou, E., Mouchlianitis, E., Joyce, D. W., McGuire, P., Boussebaa, C., Banaschewski, T., Bokde, A. L. W., Büchel, C., Quinlan, E., Desrivières, S., Flor, H., Grigis, A., Garavan, H., Spechler, P., Gowland, P., Heinz, A., Ittermann, B., Martinot, M.-L. P., Artiges, E., … Shergill, S. S. (2020). Examination of the neural basis of psychotic-like experiences in adolescence during processing of emotional faces. *Scientific Reports*, *10*(1), 5164. https://doi.org/10.1038/s41598-020-62026-7

Paquola, C., Bennett, M. R., Hatton, S. N., Hermens, D. F., & Lagopoulos, J. (2017). Utility of the cumulative stress and mismatch hypotheses in understanding the neurobiological impacts of childhood abuse and recent stress in youth with emerging mental disorder. *Human Brain Mapping*, *38*(5), 2709–2721. https://doi.org/10.1002/hbm.23554

Paraskevopoulou, M., van Rooij, D., Batalla, A., Chauvin, R., Luijten, M., Schene, A. H., Buitelaar, J. K., & Schellekens, A. F. A. (2021). Effects of substance misuse on reward-processing in patients with attention-deficit/hyperactivity disorder. *Neuropsychopharmacology*, *46*(3), 622–631. https://doi.org/10.1038/s41386-020-00896-1

Park, S. won, Lee, J. ha, Kim, J., Suh, S., & Lee, M. S. (2018). Changes in heart rate variability in first-episode drug-naïve adolescents with major depressive disorder: A 12-week prospective study. *Journal of Affective Disorders*, *238*, 250–255. https://doi.org/10.1016/J.JAD.2018.05.068

Parker, N., Patel, Y., Jackowski, A. P., Pan, P. M., Salum, G. A., Pausova, Z., & Paus, T. (2020). Assessment of Neurobiological Mechanisms of Cortical Thinning During Childhood and Adolescence and Their Implications for Psychiatric Disorders. *JAMA Psychiatry*, *77*(11), 1127. https://doi.org/10.1001/jamapsychiatry.2020.1495

Passarotti, A. M., Sweeney, J. A., & Pavuluri, M. N. (2010). Neural correlates of response inhibition in pediatric bipolar disorder and attention deficit hyperactivity disorder. *Psychiatry Research: Neuroimaging*, *181*(1), 36–43. https://doi.org/10.1016/j.pscychresns.2009.07.002

Pathmasiri, W., Pratt, K. J., Collier, D. N., Lutes, L. D., McRitchie, S., & Sumner, S. C. J. (2012). Integrating metabolomic signatures and psychosocial parameters in responsivity to an immersion treatment model for adolescent obesity. *Metabolomics*, *8*(6), 1037–1051. https://doi.org/10.1007/s11306-012-0404-x

Paul, R. H., Cho, K. S., Belden, A. C., Mellins, C. A., Malee, K. M., Robbins, R. N., Salminen, L. E., Kerr, S. J., Adhikari, B., Garcia-Egan, P. M., Sophonphan, J., Aurpibul, L., Thongpibul, K., Kosalaraksa, P., Kanjanavanit, S., Ngampiyaskul, C., Wongsawat, J., Vonthanak, S., Suwanlerk, T., … Puthanakit, T. (2020). Machine-learning classification of neurocognitive performance in children with perinatal HIV initiating de novo antiretroviral therapy. *AIDS*, *34*(5), 737–748. https://doi.org/10.1097/QAD.0000000000002471

Pausova, Z., Paus, T., Abrahamowicz, M., Almerigi, J., Arbour, N., Bernard, M., Gaudet, D., Hanzalek, P., Hamet, P., Evans, A. C., Kramer, M., Laberge, L., Leal, S. M., Leonard, G., Lerner, J., Lerner, R. M., Mathieu, J., Perron, M., Pike, B., … Watkins, K. (2007). Genes, maternal smoking, and the offspring brain and body during adolescence: Design of the Saguenay Youth Study. *Human Brain Mapping*, *28*(6), 502–518. https://doi.org/10.1002/hbm.20402

Paveenakiattikhun, S., Likhitweerawong, N., & Sanguansermsri, C. (2025). EEG findings and clinical severity and quality of life in non-epileptic patients with autism spectrum disorders. *Child Neuropsychology*, *31*(2), 255–265. https://doi.org/10.1080/09297049.2024.2360651

Pawełczyk, T., Grancow, M., Kotlicka-Antczak, M., Trafalska, E., Gębski, P., Szemraj, J., Żurner, N., & Pawełczyk, A. (2015). Omega-3 fatty acids in first-episode schizophrenia - a randomized controlled study of efficacy and relapse prevention (OFFER): rationale, design, and methods. *BMC Psychiatry*, *15*(1), 97. https://doi.org/10.1186/s12888-015-0473-2

Pawełczyk, T., Grancow-Grabka, M., Trafalska, E., Szemraj, J., Żurner, N., & Pawełczyk, A. (2019). An increase in plasma brain derived neurotrophic factor levels is related to n-3 polyunsaturated fatty acid efficacy in first episode schizophrenia: secondary outcome analysis of the OFFER randomized clinical trial. *Psychopharmacology*, *236*(9), 2811–2822. https://doi.org/10.1007/s00213-019-05258-4

Penberthy, J. K., Cox, D., Breton, M., Robeva, R., Kalbfleisch, M. L., Loboschefski, T., & Kovatchev, B. (2005). Calibration of ADHD Assessments Across Studies: A Meta-Analysis Tool. *Applied Psychophysiology and Biofeedback*, *30*(1), 31–51. https://doi.org/10.1007/s10484-005-2172-0

Peris, T. S., Piacentini, J., Vreeland, A., Salgari, G., Levitt, J. G., Alger, J. R., Posse, S., McCracken, J. T., & O’Neill, J. (2020). Neurochemical correlates of behavioral treatment of pediatric trichotillomania. *Journal of Affective Disorders*, *273*, 552–561. https://doi.org/10.1016/J.JAD.2020.04.061

Plana, M. T., Flamarique, I., Julià, L., Tasa-Vinyals, E., Citoler, B., Díaz, C., Moreno, E., Andrés-Perpiñá, S., Martínez, E., Lázaro, L., & Castro-Fornieles, J. (2024). Accuracy of estimating self and other body size among adolescent girls with anorexia nervosa. *Eating Disorders*, *32*(1), 29–42. https://doi.org/10.1080/10640266.2023.2243425

Pollard, R., Chen, P. J., Mackes, N., Lawrence, A. J., Ma, X., Matter, M., Kretzer, S., Morgan, C., Harding, S., Schumann, G., Pariante, C., Mehta, M., Montana, G., Nosarti, C., Desrivieres, S., Rodriguez-Mateos, A., & Dazzan, P. (2022). The eBRAIN study: The impact of early adversity on trajectories of brain maturation and mental health in young adolescents – A prospective cohort study. *Brain, Behavior, & Immunity - Health*, *26*, 100539. https://doi.org/10.1016/J.BBIH.2022.100539

Poon, J. A., Thompson, J. C., Forbes, E. E., & Chaplin, T. M. (2019). Adolescents’ Reward‐related Neural Activation: Links to Thoughts of Nonsuicidal Self‐Injury. *Suicide and Life-Threatening Behavior*, *49*(1), 76–89. https://doi.org/10.1111/sltb.12418

Portnova, G. v., Nekrashevich, M. v., Morozova, M. v., Martynova, O. v., & Sharaev, M. G. (2023). New approaches to clinical electroencephalography analysis in typically developing children and children with autism. *Cognitive Systems Research*, *78*, 23–32. https://doi.org/10.1016/J.COGSYS.2022.11.003

Qi, W., Wen, Z., Chen, J., Capichioni, G., Ando, F., Chen, Z. S., Wang, J., Yoncheva, Y., Castellanos, F. X., Milad, M., & Goff, D. C. (2023). Aberrant resting-state functional connectivity of the globus pallidus interna in first-episode schizophrenia. *Schizophrenia Research*, *261*, 100–106. https://doi.org/10.1016/j.schres.2023.09.018

Qi, X., Du, X., Yang, Y., Du, G., Gao, P., Zhang, Y., Qin, W., Li, X., & Zhang, Q. (2015). Decreased modulation by the risk level on the brain activation during decision making in adolescents with internet gaming disorder. *Frontiers in Behavioral Neuroscience*, *9*. https://doi.org/10.3389/fnbeh.2015.00296

Qiao, D., Qi, Y., Zhang, X., Wen, Y., Huang, Y., Li, Y., Liu, P., Li, G., & Liu, Z. (2025). The possible effect of inflammation on non-suicidal self-injury in adolescents with depression: a mediator of connectivity within corticostriatal reward circuitry. *European Child & Adolescent Psychiatry*, *34*(9), 2871–2885. https://doi.org/10.1007/s00787-025-02709-6

Qin, N., Yi, S., Dai, C., Liu, X., Duan, Y., Zhou, Y., Wan, Z., Kang, Y., Zhou, X., Xie, J., & Cheng, A. S. K. (2023). Associations of serum cortisol and inflammatory marker features with depression and anxiety in young women with gynecologic cancer. *Supportive Care in Cancer*, *31*(12), 674. https://doi.org/10.1007/s00520-023-08145-z

Qiu, T., Li, X., Chen, W., He, J., Shi, L., Zhou, C., Zheng, A., Lei, Z., Tang, C., Yu, Q., Du, L., & Guo, J. (2023). Prospective study on Maresin-1 and cytokine levels in medication-naïve adolescents with first-episode major depressive disorder. *Frontiers in Psychiatry*, *14*. https://doi.org/10.3389/fpsyt.2023.1132791

Qiu, Y., Dou, H., Becker, B., He, Z., Mei, Y., & Lei, Y. (2025). Behavioral and neural dysfunctions in reward-related cognitive control among adolescents with major depressive disorder. *Psychological Medicine*, *55*, e298. https://doi.org/10.1017/S003329172510202X

Quevedo, K., Liu, G., Teoh, J. Y., Ghosh, S., Zeffiro, T., Ahrweiler, N., Zhang, N., Wedan, R., Oh, S., Guercio, G., & Paret, C. (2019). Neurofeedback and neuroplasticity of visual self-processing in depressed and healthy adolescents: A preliminary study. *Developmental Cognitive Neuroscience*, *40*, 100707. https://doi.org/10.1016/J.DCN.2019.100707

Quinlan, E. B., Cattrell, A., Jia, T., Artiges, E., Banaschewski, T., Barker, G., Bokde, A. L. W., Bromberg, U., Büchel, C., Brühl, R., Conrod, P. J., Desrivieres, S., Flor, H., Frouin, V., Gallinat, J., Garavan, H., Gowland, P., Heinz, A., Martinot, J.-L., … Schumann, G. (2017). Psychosocial Stress and Brain Function in Adolescent Psychopathology. *American Journal of Psychiatry*, *174*(8), 785–794. https://doi.org/10.1176/appi.ajp.2017.16040464

Rabaya, S., Nairat, S., Bader, K., Herzallah, M. M., & Darwish, H. M. (2023). Iron metabolism in autism spectrum disorder; inference through single nucleotide polymorphisms in key iron metabolism genes. *Journal of the Neurological Sciences*, *453*, 120817. https://doi.org/10.1016/j.jns.2023.120817

Rahdar, A., & Galván, A. (2014). The cognitive and neurobiological effects of daily stress in adolescents. *NeuroImage*, *92*, 267–273. https://doi.org/10.1016/J.NEUROIMAGE.2014.02.007

Rahko, J. S., Vuontela, V. A., Carlson, S., Nikkinen, J., Hurtig, T. M., Kuusikko-Gauffin, S., Mattila, M.-L., Jussila, K. K., Remes, J. J., Jansson-Verkasalo, E. M., Aronen, E. T., Pauls, D. L., Ebeling, H. E., Tervonen, O., Moilanen, I. K., & Kiviniemi, V. J. (2016). Attention and Working Memory in Adolescents with Autism Spectrum Disorder: A Functional MRI Study. *Child Psychiatry & Human Development*, *47*(3), 503–517. https://doi.org/10.1007/s10578-015-0583-6

Rakshasa-Loots, A. M., Steyn, C., Swiffen, D., Marwick, K. F. M., Semple, R. K., Reynolds, R. M., Burgess, K., Lawrie, S. M., Lightman, S. L., Luz, S., & Smith, D. J. (2025). Metabolic biomarkers of clinical outcomes in severe mental illness (METPSY): protocol for a prospective observational study in the Hub for metabolic psychiatry. *BMC Psychiatry*, *25*(1), 122. https://doi.org/10.1186/s12888-025-06579-9

Ramage, A. E., Lin, A. L., Olvera, R. L., Fox, P. T., & Williamson, D. E. (2015). Resting-state regional cerebral blood flow during adolescence: Associations with initiation of substance use and prediction of future use disorders. *Drug and Alcohol Dependence*, *149*, 40–48. https://doi.org/10.1016/J.DRUGALCDEP.2015.01.012

Rao, U., Hammen, C. L., & Poland, R. E. (2009). Risk Markers for Depression in Adolescents: Sleep and HPA Measures. *Neuropsychopharmacology*, *34*(8), 1936–1945. https://doi.org/10.1038/npp.2009.27

Rauschenbach, A.-L., Hauffe, V., Fink-Lamotte, J., Tuschen-Caffier, B., & Schmitz, J. (2024). Reduced early neural processing of faces in children and adolescents with social anxiety disorder. *Biological Psychology*, *191*, 108827. https://doi.org/10.1016/j.biopsycho.2024.108827

Ravagnani Salto, A. B., Santoro, M. L., Hoexter, M. Q., Jackowski, A. P., Pan, P. M., Rosário, M. C., Belangero, S. I., Alvarenga, P. G., Doretto, V. F., Fumo, A. M. T., Batistuzzo, M. C., Macul Ferreira de Barros, P., Timpano, K. R., Ota, V. K., Rohde, L. A., Miguel, E. C., Leckman, J. F., & Zugman, A. (2021). Obsessive-Compulsive Symptoms, Polygenic Risk Score, and Thalamic Development in Children From the Brazilian High-Risk Cohort for Mental Conditions (BHRCS). *Frontiers in Psychiatry*, *12*. https://doi.org/10.3389/fpsyt.2021.673595

Raz, A., Zhu, H., Yu, S., Bansal, R., Wang, Z., Alexander, G. M., Royal, J., & Peterson, B. S. (2009). Neural Substrates of Self-Regulatory Control in Children and Adults with Tourette Syndrome. *The Canadian Journal of Psychiatry*, *54*(9), 579–588. https://doi.org/10.1177/070674370905400902

Reasoner, E. E., van der Plas, E., Al‐Kaylani, H. M., Langbehn, D. R., Conrad, A. L., Schultz, J. L., Epping, E. A., Magnotta, V. A., & Nopoulos, P. C. (2022). Behavioral features in child and adolescent huntingtin gene‐mutation carriers. *Brain and Behavior*, *12*(7). https://doi.org/10.1002/brb3.2630

Rempel, S., Backhausen, L. L., McDonald, M., Roessner, V., Vetter, N. C., Beste, C., & Wolff, N. (2023). App-Based Mindfulness Meditation Training and an Audiobook Intervention Reduce Symptom Severity but Do Not Modify Backward Inhibition in Adolescent Obsessive-Compulsive Disorder: Evidence from an EEG Study. *Journal of Clinical Medicine*, *12*(7), 2486. https://doi.org/10.3390/jcm12072486

Ren, W., Liu, X., Zhang, Z., Zhang, X., Wu, W., Wang, X., Yu, Z., Liu, C., & Yu, R. (2025). Dynamics of cerebral blood flow following sertraline treatment in adolescent depression. *Frontiers in Psychiatry*, *16*. https://doi.org/10.3389/fpsyt.2025.1521565

Richert, K. A., Carrion, V. G., Karchemskiy, A., & Reiss, A. L. (2006). Regional differences of the prefrontal cortex in pediatric PTSD: an MRI study. *Depression and Anxiety*, *23*(1), 17–25. https://doi.org/10.1002/da.20131

Rnic, K., Jopling, E., Tracy, A., & LeMoult, J. (2022). Emotion regulation and diurnal cortisol: A longitudinal study of early adolescents. *Biological Psychology*, *167*, 108212. https://doi.org/10.1016/j.biopsycho.2021.108212

Roberts, C. M., Gamwell, K. L., Baudino, M. N., Edwards, C. S., Jacobs, N. J., Tung, J., Grunow, J. E., Mullins, L. L., & Chaney, J. M. (2022). Illness stigma, body image dissatisfaction, thwarted belongingness and depressive symptoms in youth with inflammatory bowel disease. *European Journal of Gastroenterology & Hepatology*, *34*(9), 919–924. https://doi.org/10.1097/MEG.0000000000002420

Roberts, S., Keers, R., Lester, K. J., Coleman, J. R. I., Breen, G., Arendt, K., Blatter‐Meunier, J., Cooper, P., Creswell, C., Fjermestad, K., Havik, O. E., Herren, C., Hogendoorn, S. M., Hudson, J. L., Krause, K., Lyneham, H. J., Morris, T., Nauta, M., Rapee, R. M., … Wong, C. C. Y. (2015). HPA AXIS RELATED GENES AND RESPONSE TO PSYCHOLOGICAL THERAPIES: GENETICS AND EPIGENETICS. *Depression and Anxiety*, *32*(12), 861–870. https://doi.org/10.1002/da.22430

Rodman, A. M., Jenness, J. L., Weissman, D. G., Pine, D. S., & McLaughlin, K. A. (2019). Neurobiological Markers of Resilience to Depression Following Childhood Maltreatment: The Role of Neural Circuits Supporting the Cognitive Control of Emotion. *Biological Psychiatry*, *86*(6), 464–473. https://doi.org/10.1016/j.biopsych.2019.04.033

Rodriguez-Thompson, A. M., Meyer, K. M., Davidow, J. Y., van Dijk, K. R. A., Santillana, R. M., Snyder, J., Vidal Bustamante, C. M., Hollinshead, M. O., Rosen, B. R., Somerville, L. H., & Sheridan, M. A. (2020). Examining cognitive control and reward interactions in adolescent externalizing symptoms. *Developmental Cognitive Neuroscience*, *45*, 100813. https://doi.org/10.1016/j.dcn.2020.100813

Roetner, J., van Doren, J., Maschke, J., Kulke, L., Pontones, C., Fasching, P. A., Beckmann, M. W., Lenz, B., Kratz, O., Moll, G. H., Kornhuber, J., & Eichler, A. (2024). Effects of prenatal alcohol exposition on cognitive outcomes in childhood and youth: a longitudinal analysis based on meconium ethyl glucuronide. *European Archives of Psychiatry and Clinical Neuroscience*, *274*(2), 343–352. https://doi.org/10.1007/s00406-023-01657-z

Rohrsetzer, F., Balardin, J. B., Picon, F., Sato, J. R., Battel, L., Viduani, A., Manfro, P. H., Yoon, L., Kohrt, B. A., Fisher, H. L., Mondelli, V., Swartz, J. R., & Kieling, C. (2023). A magnetic resonance imaging-based morphometric and structural covariance network study of Brazilian adolescents stratified by depression risk. *Brazilian Journal of Psychiatry*. https://doi.org/10.47626/1516-4446-2023-3037

Rommel, A.-S., James, S.-N., McLoughlin, G., Brandeis, D., Banaschewski, T., Asherson, P., & Kuntsi, J. (2017). Altered EEG spectral power during rest and cognitive performance: a comparison of preterm-born adolescents to adolescents with ADHD. *European Child & Adolescent Psychiatry*, *26*(12), 1511–1522. https://doi.org/10.1007/s00787-017-1010-2

Rosa-Neto, P., Lou, H. C., Cumming, P., Pryds, O., Karrebaek, H., Lunding, J., & Gjedde, A. (2005). Methylphenidate-evoked changes in striatal dopamine correlate with inattention and impulsivity in adolescents with attention deficit hyperactivity disorder. *NeuroImage*, *25*(3), 868–876. https://doi.org/10.1016/j.neuroimage.2004.11.031

Rosen, M. L., Sheridan, M. A., Sambrook, K. A., Dennison, M. J., Jenness, J. L., Askren, M. K., Meltzoff, A. N., & McLaughlin, K. A. (2018). Salience network response to changes in emotional expressions of others is heightened during early adolescence: relevance for social functioning. *Developmental Science*, *21*(3). https://doi.org/10.1111/desc.12571

Rosenau, P. T., van den Hoofdakker, B. J., Matthijssen, A.-F. M., van de Loo-Neus, G. H. H., Buitelaar, J. K., Hoekstra, P. J., & Dietrich, A. (2022). Withdrawing methylphenidate in relation to serum levels of ferritin and zinc in children and adolescents with attention-deficit/hyperactivity disorder. *Journal of Psychiatric Research*, *152*, 31–37. https://doi.org/10.1016/j.jpsychires.2022.06.014

Ross, A. J., Roule, A. L., Deveney, C. M., Towbin, K. E., Brotman, M. A., Leibenluft, E., & Tseng, W. (2021). A preliminary study on functional activation and connectivity during frustration in youths with bipolar disorder. *Bipolar Disorders*, *23*(3), 263–273. https://doi.org/10.1111/bdi.12985

Roybal, D. J., Barnea-Goraly, N., Kelley, R., Bararpour, L., Howe, M. E., Reiss, A. L., & Chang, K. D. (2015). Widespread white matter tract aberrations in youth with familial risk for bipolar disorder. *Psychiatry Research: Neuroimaging*, *232*(2), 184–192. https://doi.org/10.1016/j.pscychresns.2015.02.007

Rudå, D., Jensen, K. G., Decara, M. S., Klauber, D. G., Fagerlund, B., Møllegaard, J. R., Linnet, K., Werge, T., Correll, C. U., Fink-Jensen, A., Jürgens, G., & Pagsberg, A. K. (2021). CYP2D6 Genotyping and Antipsychotic-Associated Extrapyramidal Adverse Effects in a Randomized Trial of Aripiprazole Versus Quetiapine Extended Release in Children and Adolescents, Aged 12–17 Years, With First Episode Psychosis. *Journal of Clinical Psychopharmacology*, *41*(6), 667–672. https://doi.org/10.1097/JCP.0000000000001490

Rzepa, E., & McCabe, C. (2016). Decreased anticipated pleasure correlates with increased salience network resting state functional connectivity in adolescents with depressive symptomatology. *Journal of Psychiatric Research*, *82*, 40–47. https://doi.org/10.1016/j.jpsychires.2016.07.013

Saito, S., Watanabe, K., Hashimoto, E., & Saito, T. (2009). Low serum BDNF and food intake regulation: A possible new explanation of the pathophysiology of eating disorders. *Progress in Neuro-Psychopharmacology and Biological Psychiatry*, *33*(2), 312–316. https://doi.org/10.1016/j.pnpbp.2008.12.009

Saitoh, O. (2001). Development of the hippocampal formation from2 to 42 years: MRI evidence of smaller area dentata in autism. *Brain*, *124*(7), 1317–1324. https://doi.org/10.1093/brain/124.7.1317

Sakuma, A., Obara, C., Katsura, M., Ito, F., Ohmuro, N., Iizuka, K., Kikuchi, T., Miyakoshi, T., Matsuoka, H., & Matsumoto, K. (2018). No regional gray matter volume reduction observed in young Japanese people at ultra-high risk for psychosis: A voxel-based morphometry study. *Asian Journal of Psychiatry*, *37*, 167–171. https://doi.org/10.1016/J.AJP.2018.09.009

Salum, G. A., Gadelha, A., Pan, P. M., Moriyama, T. S., Graeff‐Martins, A. S., Tamanaha, A. C., Alvarenga, P., Krieger, F. V., Fleitlich‐Bilyk, B., Jackowski, A., Sato, J. R., Brietzke, E., Polanczyk, G. V., Brentani, H., de Jesus Mari, J., do Rosário, M. C., Manfro, G. G., Bressan, R. A., Mercadante, M. T., … Rohde, L. A. (2015). High risk cohort study for psychiatric disorders in childhood: rationale, design, methods and preliminary results. *International Journal of Methods in Psychiatric Research*, *24*(1), 58–73. https://doi.org/10.1002/mpr.1459

Salum, G. A., Isolan, L. R., Bosa, V. L., Tocchetto, A. G., Teche, S. P., Schuch, I., Costa, J. R., Costa, M. de A., Jarros, R. B., Mansur, M. A., Knijnik, D., Silva, E. A., Kieling, C., Oliveira, M. H., Medeiros, E., Bortoluzzi, A., Toazza, R., Blaya, C., Leistner-Segal, S., … Manfro, G. G. (2011). The multidimensional evaluation and treatment of anxiety in children and adolescents: rationale, design, methods and preliminary findings. *Revista Brasileira de Psiquiatria*, *33*(2), 181–195. https://doi.org/10.1590/S1516-44462011000200015

Sandhya Joseph, J., & Chinnathambi, K. (2024). Adolescent Focused Intervention Bundle for Institutionalized Adolescents’ Health Promotion: A Study Protocol. *Journal of Client-Centered Nursing Care*, *10*(4), 277–286. https://doi.org/10.32598/JCCNC.10.4.653.1

Santoro, J., Spinazzi, N., Filipink, R., Hayati-Rezvan, P., Kammeyer, R., Patel, L., Sannar, E., Dwyer, L., Banerjee, A., Khoshnood, M., Jafarpour, S., Boyd, N., Partridge, R., Gombolay, G., Christy, A., de Asua, D. R., Ortega, M. del C., Manning, M., Mater, H. van, … Rafii, M. (2023). *Immunotherapy Responsiveness and Risk of Relapse in Down Syndrome Regression Disorder*. https://doi.org/10.21203/rs.3.rs-2521595/v1

Saxena, K., Simonetti, A., Verrico, C. D., Janiri, D., Nicola, M. di, Catinari, A., Kurian, S., Saxena, J., Mwangi, B., & Soares, J. C. (2023). Neurocognitive Correlates of Cerebellar Volumetric Alterations in Youth with Pediatric Bipolar Spectrum Disorders and Bipolar Offspring. *Current Neuropharmacology*, *21*(6), 1367–1378. https://doi.org/10.2174/1570159X21666221014120332

Scherff, A. D., Feldmann, L., Piechaczek, C., Pehl, V., Wagenbüchler, P., Wermuth, I., Ghotbi, N., Allgaier, A.-K., Freisleder, F. J., Beins, E. C., Forstner, A. J., Nöthen, M. M., Czamara, D., Rex-Haffner, M., Ising, M., Binder, E., Greimel, E., & Schulte-Körne, G. (2024). Cohort profile: BioMD-Y (biopsychosocial factors of major depression in youth) – a biobank study on the molecular genetics and environmental factors of depression in children and adolescents in Munich. *BMJ Open*, *14*(3), e074925. https://doi.org/10.1136/bmjopen-2023-074925

Schloesser, L., Lotter, L. D., Offermann, J., Borucki, K., Biemann, R., Seitz, J., Konrad, K., & Herpertz‐Dahlmann, B. (2023). Sex‐dependent clinical presentation, body image, and endocrine status in long‐term remitted anorexia nervosa. *European Eating Disorders Review*, *31*(5), 696–708. https://doi.org/10.1002/erv.2997

Schmaal, L., Hibar, D. P., Sämann, P. G., Hall, G. B., Baune, B. T., Jahanshad, N., Cheung, J. W., van Erp, T. G. M., Bos, D., Ikram, M. A., Vernooij, M. W., Niessen, W. J., Tiemeier, H., Hofman, A., Wittfeld, K., Grabe, H. J., Janowitz, D., Bülow, R., Selonke, M., … Veltman, D. J. (2017). Cortical abnormalities in adults and adolescents with major depression based on brain scans from 20 cohorts worldwide in the ENIGMA Major Depressive Disorder Working Group. *Molecular Psychiatry*, *22*(6), 900–909. https://doi.org/10.1038/mp.2016.60

Schmidt, N. L., Lemery-Chalfant, K., & Goldsmith, H. H. (2019). Wisconsin Twin Project Overview: Temperament and Affective Neuroscience. *Twin Research and Human Genetics*, *22*(6), 794–799. https://doi.org/10.1017/thg.2019.108

Schmidt, N. L., van Hulle, C. A., Brooker, R. J., Meyer, L. R., Lemery-Chalfant, K., & Goldsmith, H. H. (2013). Wisconsin Twin Research: Early Development, Childhood Psychopathology, Autism, and Sensory Over-responsivity. *Twin Research and Human Genetics*, *16*(1), 376–384. https://doi.org/10.1017/thg.2012.105

Schnall, R., Liu, J., Cordoba, E., Brin, M., Garofalo, R., Kuhns, L. M., Jandev, V., Careem, F. R., Hidoyatov, M., Padilla, J. J., Pearson, C., Batey, D. S., Norful, A. A., & Bendinskas, K. (2024). Differences in Self-Reported Stress Versus Hair and Nail Cortisol Among Adolescent and Young Adult Males. *Nursing Research*, *73*(6), 442–449. https://doi.org/10.1097/NNR.0000000000000757

Schreiber, A. M., Martinez, M., Li, S., Williams, T. F., Alexander, N., Mittal, V. A., & Haase, C. M. (2026). Disrupted physiological coregulation in youth at clinical high risk for psychosis: Insights from a dyadic interaction study. *Emotion*, *26*(2), 420–432. https://doi.org/10.1037/emo0001578

Schreiber, J. M., Lanham, D. C., Trescher, W. H., Sparks, S. E., Wassif, C. A., Caffo, B. S., Porter, F. D., Tierney, E., Gropman, A. L., & Ewen, J. B. (2014). Variations in EEG discharges predict ADHD severity within individual Smith-Lemli-Opitz patients. *Neurology*, *83*(2), 151–159. https://doi.org/10.1212/WNL.0000000000000565

Schumann, G., Loth, E., Banaschewski, T., Barbot, A., Barker, G., Büchel, C., Conrod, P. J., Dalley, J. W., Flor, H., Gallinat, J., Garavan, H., Heinz, A., Itterman, B., Lathrop, M., Mallik, C., Mann, K., Martinot, J.-L., Paus, T., Poline, J.-B., … Struve, M. (2010). The IMAGEN study: reinforcement-related behaviour in normal brain function and psychopathology. *Molecular Psychiatry*, *15*(12), 1128–1139. https://doi.org/10.1038/mp.2010.4

Schvey, N. A., Shank, L. M., Tanofsky‐Kraff, M., Ramirez, S., Altman, D. R., Swanson, T., Rubin, A. G., Kelly, N. R., LeMay‐Russell, S., Byrne, M. E., Parker, M. N., Broadney, M. M., Brady, S. M., Yanovski, S. Z., & Yanovski, J. A. (2021). <scp>Weight‐based</scp> teasing in youth: Associations with metabolic and inflammatory markers. *Pediatric Obesity*, *16*(3). https://doi.org/10.1111/ijpo.12729

Schwartzman, J. M., Kujawa, A., Jeste, S. S., Corbett, B. A., Xu, W., Monachino, A. D., Castro, M. A., Cardenas-Quintero, K., Dao, A., Durazo-Arvizu, R., & Morales, S. (2025). Study protocol for a multimethod investigation of the development of social and nonsocial reward responsivity and depression in autistic adolescents: Reward and Depression in Autism (RDA). *BMC Psychology*, *13*(1), 603. https://doi.org/10.1186/s40359-025-02911-w

Schweinsburg, A. D., McQueeny, T., Nagel, B. J., Eyler, L. T., & Tapert, S. F. (2010). A preliminary study of functional magnetic resonance imaging response during verbal encoding among adolescent binge drinkers. *Alcohol*, *44*(1), 111–117. https://doi.org/10.1016/j.alcohol.2009.09.032

Seelemeyer, H., Gurr, C., Leyhausen, J., Berg, L. M., Pretzsch, C. M., Schäfer, T., Hermila, B., Freitag, C. M., Loth, E., Oakley, B., Mason, L., Buitelaar, J. K., Beckmann, C. F., Floris, D. L., Charman, T., Banaschewski, T., Jones, E., Bourgeron, T., Ahmad, J., … Ecker, C. (2025). Decomposing the Brain in Autism: Linking Behavioral Domains to Neuroanatomical Variation and Genomic Underpinnings. *Biological Psychiatry: Cognitive Neuroscience and Neuroimaging*, *10*(10), 1067–1077. https://doi.org/10.1016/j.bpsc.2024.12.003

Seewoo, B. J., Rodger, J., Demitrack, M. A., Heart, K. L., Port, J. D., Strawn, J. R., & Croarkin, P. E. (2022). Neurostructural Differences in Adolescents With Treatment-Resistant Depression and Treatment Effects of Transcranial Magnetic Stimulation. *International Journal of Neuropsychopharmacology*, *25*(8), 619–630. https://doi.org/10.1093/ijnp/pyac007

Segal, T. Y., Hindmarsh, P. C., & Viner, R. M. (2005). Disturbed Adrenal Function in Adolescents with Chronic Fatigue Syndrome. *Journal of Pediatric Endocrinology and Metabolism*, *18*(3). https://doi.org/10.1515/JPEM.2005.18.3.295

Segura, A.-G., Prohens, L., Julià, L., Amoretti, S., RIbero, M., Pino-Camacho, L., Cano-Escalera, G., Mane, A., Rodriguez-Jimenez, R., Roldan, A., Sarró, S., Ibañez, A., Usall, J., Lobo, A., Garcia-Rizo, C., Cuesta, M. J., Parellada, M., González-Pinto, A., Berrocoso, E., … De-la-Cámara, C. (2025). Methylation profile scores of environmental exposures and risk of relapse after a first episode of schizophrenia. *European Neuropsychopharmacology*, *94*, 4–15. https://doi.org/10.1016/j.euroneuro.2025.02.003

Seidman, L. J., Thermenos, H. W., Poldrack, R. A., Peace, N. K., Koch, J. K., Faraone, S. v., & Tsuang, M. T. (2006). Altered brain activation in dorsolateral prefrontal cortex in adolescents and young adults at genetic risk for schizophrenia: An fMRI study of working memory. *Schizophrenia Research*, *85*(1–3), 58–72. https://doi.org/10.1016/J.SCHRES.2006.03.019

Seidman, S. B., Danzo, S., Patton, E., & Connell, A. M. (2020). Here’s looking at you, kid? Maternal depression and adolescent attention to self- or other-directed emotional faces. *Journal of Affective Disorders*, *272*, 38–45. https://doi.org/10.1016/j.jad.2020.03.149

Seif, E. A., Diab, I. H., Ibrahim, S. A., Hussein, H. A., & Ghitani, S. A. (2019). Association between different parameters of child maltreatment and global DNA methylation. *Alexandria Journal of Medicine*, *55*(1), 82–88. https://doi.org/10.1080/20905068.2019.1681178

Seiger, R., Reggente, N., Majid, D. S.-A., Ly, R., Tadayonnejad, R., Strober, M., & Feusner, J. D. (2023). Neural representations of anxiety in adolescents with anorexia nervosa: a multivariate approach. *Translational Psychiatry*, *13*(1), 283. https://doi.org/10.1038/s41398-023-02581-5

Selim, S., Riesenberg, R., Cassella, J., Kunta, J., Hellriegel, E., Smith, M. A., Vinks, A. A., & Rabinovich‐Guilatt, L. (2017). Pharmacokinetics and Safety of Single‐Dose Inhaled Loxapine in Children and Adolescents. *The Journal of Clinical Pharmacology*, *57*(10), 1244–1257. https://doi.org/10.1002/jcph.932

Shafer, A. T., Benoit, J. R., Brown, M. R. G., Greenshaw, A. J., van Vliet, K. J., Vohra, S., Dolcos, F., & Singhal, A. (2020). Differences in attentional control and white matter microstructure in adolescents with attentional, affective, and behavioral disorders. *Brain Imaging and Behavior*, *14*(2), 599–614. https://doi.org/10.1007/s11682-019-00211-7

Shakeel, M. K., MacQueen, G., Addington, J., Metzak, P. D., Georgopoulos, G., Bray, S., Goldstein, B. I., MacIntosh, B. J., Wang, J., Kennedy, S. H., & Lebel, C. (2020). White Matter Connectivity in Youth at Risk for Serious Mental Illness: A Longitudinal Analysis. *Psychiatry Research: Neuroimaging*, *302*, 111106. https://doi.org/10.1016/j.pscychresns.2020.111106

Shanmugan, S., Wolf, D. H., Calkins, M. E., Moore, T. M., Ruparel, K., Hopson, R. D., Vandekar, S. N., Roalf, D. R., Elliott, M. A., Jackson, C., Gennatas, E. D., Leibenluft, E., Pine, D. S., Shinohara, R. T., Hakonarson, H., Gur, R. C., Gur, R. E., & Satterthwaite, T. D. (2016). Common and Dissociable Mechanisms of Executive System Dysfunction Across Psychiatric Disorders in Youth. *American Journal of Psychiatry*, *173*(5), 517–526. https://doi.org/10.1176/appi.ajp.2015.15060725

Sharma, V., Saini, A. G., Malhi, P., & Singhi, P. (2022). Epilepsy and EEG Abnormalities in Children with Autism Spectrum Disorders. *Indian Journal of Pediatrics*, *89*(10), 975–982. https://doi.org/10.1007/s12098-021-03928-w

Shchelochkov, O. A., Farmer, C. A., Chlebowski, C., Adedipe, D., Ferry, S., Manoli, I., Pass, A., McCoy, S., van Ryzin, C., Sloan, J., Thurm, A., & Venditti, C. P. (2024). Intellectual disability and autism in propionic acidemia: a biomarker-behavioral investigation implicating dysregulated mitochondrial biology. *Molecular Psychiatry*, *29*(4), 974–981. https://doi.org/10.1038/s41380-023-02385-5

Sheerin, C. M., Lancaster, E. E., York, T. P., Walker, J., Danielson, C. K., & Amstadter, A. B. (2021). Epigenome‐Wide Study of Posttraumatic Stress Disorder Symptom Severity in a Treatment‐Seeking Adolescent Sample. *Journal of Traumatic Stress*, *34*(3), 607–615. https://doi.org/10.1002/jts.22655

Shen, M. D., Shih, P., Öttl, B., Keehn, B., Leyden, K. M., Gaffrey, M. S., & Müller, R. A. (2012). Atypical lexicosemantic function of extrastriate cortex in autism spectrum disorder: Evidence from functional and effective connectivity. *NeuroImage*, *62*(3), 1780–1791. https://doi.org/10.1016/J.NEUROIMAGE.2012.06.008

Shen, X., Wang, C., Alimujiang, A., Zhang, C., Zou, S., & Liu, L. (2025). The relationship of methylation and mRNA expression profile of AUTS2 with suicidal ideation in adolescents with bipolar depression. *BMC Psychiatry*, *25*(1), 543. https://doi.org/10.1186/s12888-025-06927-9

Shenk, C. E., O’Donnell, K. J., Pokhvisneva, I., Kobor, M. S., Meaney, M. J., Bensman, H. E., Allen, E. K., & Olson, A. E. (2022). Epigenetic Age Acceleration and Risk for Posttraumatic Stress Disorder following Exposure to Substantiated Child Maltreatment. *Journal of Clinical Child & Adolescent Psychology*, *51*(5), 651–661. https://doi.org/10.1080/15374416.2020.1864738

Shi, X., Wang, H., Zhao, Y., Wang, C., Qi, Y., Gao, M., Wei, M., & Wang, G. (2025). Neural oscillations induced by gamma sensory entrainment in cognitive biotype of depression. *Frontiers in Psychiatry*, *16*. https://doi.org/10.3389/fpsyt.2025.1586075

Sichko, S., Bui, T. Q., Vinograd, M., Shields, G. S., Saha, K., Devkota, S., Olvera-Alvarez, H. A., Carroll, J. E., Cole, S. W., Irwin, M. R., & Slavich, G. M. (2021). Psychobiology of Stress and Adolescent Depression (PSY SAD) Study: Protocol overview for an fMRI-based multi-method investigation. *Brain, Behavior, & Immunity - Health*, *17*, 100334. https://doi.org/10.1016/J.BBIH.2021.100334

Siegel, M., Smith, K. A., Mazefsky, C., Gabriels, R. L., Erickson, C., Kaplan, D., Morrow, E. M., Wink, L., & Santangelo, S. L. (2015). The autism inpatient collection: methods and preliminary sample description. *Molecular Autism*, *6*(1), 61. https://doi.org/10.1186/s13229-015-0054-8

Silk, J. S., Sequeira, S. S., Jones, N. P., Lee, K. H., Dahl, R. E., Forbes, Erika. E., Ryan, N. D., & Ladouceur, C. D. (2023). Subgenual Anterior Cingulate Cortex Reactivity to Rejection Vs. Acceptance Predicts Depressive Symptoms among Adolescents with an Anxiety History. *Journal of Clinical Child & Adolescent Psychology*, *52*(5), 659–674. https://doi.org/10.1080/15374416.2021.2019048

SIMEON, J. G., FERGUSON, H. B., KNOTT, V., ROBERTS, N., GAUTHIER, B., DUBOIS, C., & WIGGINS, D. (1992). Clinical, Cognitive, and Neurophysiological Effects of Alprazolam in Children and Adolescents with Overanxious and Avoidant Disorders. *Journal of the American Academy of Child & Adolescent Psychiatry*, *31*(1), 29–33. https://doi.org/10.1097/00004583-199201000-00006

Singh, M. K., Chang, K. D., Kelley, R. G., Saggar, M., Reiss, A. L., & Gotlib, I. H. (2014). Early signs of anomalous neural functional connectivity in healthy offspring of parents with bipolar disorder. *Bipolar Disorders*, *16*(7), 678–689. https://doi.org/10.1111/bdi.12221

Singh, M. K., Chang, K. D., Mazaika, P., Garrett, A., Adleman, N., Kelley, R., Howe, M., & Reiss, A. (2010). Neural Correlates of Response Inhibition in Pediatric Bipolar Disorder. *Journal of Child and Adolescent Psychopharmacology*, *20*(1), 15–24. https://doi.org/10.1089/cap.2009.0004

Singh, M. K., Nimarko, A. F., Garrett, A. S., Gorelik, A. J., Roybal, D. J., Walshaw, P. D., Chang, K. D., & Miklowitz, D. J. (2021). Changes in Intrinsic Brain Connectivity in Family-Focused Therapy Versus Standard Psychoeducation Among Youths at High Risk for Bipolar Disorder. *Journal of the American Academy of Child & Adolescent Psychiatry*, *60*(4), 458–469. https://doi.org/10.1016/j.jaac.2020.07.892

Smesny, S., Milleit, B., Hipler, U.-C., Milleit, C., Schäfer, M. R., Klier, C. M., Holub, M., Holzer, I., Berger, G. E., Otto, M., Nenadic, I., Berk, M., McGorry, P. D., Sauer, H., & Amminger, G. P. (2014). Omega-3 fatty acid supplementation changes intracellular phospholipase A2 activity and membrane fatty acid profiles in individuals at ultra-high risk for psychosis. *Molecular Psychiatry*, *19*(3), 317–324. https://doi.org/10.1038/mp.2013.7

Smith, A. B., Taylor, E., Brammer, M., Halari, R., & Rubia, K. (2008). Reduced activation in right lateral prefrontal cortex and anterior cingulate gyrus in medication‐naïve adolescents with attention deficit hyperactivity disorder during time discrimination. *Journal of Child Psychology and Psychiatry*, *49*(9), 977–985. https://doi.org/10.1111/j.1469-7610.2008.01870.x

Smith, A. R., White, L. K., Leibenluft, E., McGlade, A. L., Heckelman, A. C., Haller, S. P., Buzzell, G. A., Fox, N. A., & Pine, D. S. (2020). The Heterogeneity of Anxious Phenotypes: Neural Responses to Errors in Treatment-Seeking Anxious and Behaviorally Inhibited Youths. *Journal of the American Academy of Child & Adolescent Psychiatry*, *59*(6), 759–769. https://doi.org/10.1016/j.jaac.2019.05.014

Sokhadze, E. M., El-Baz, A. S., Tasman, A., Sears, L. L., Wang, Y., Lamina, E. v., & Casanova, M. F. (2014). Neuromodulation Integrating rTMS and Neurofeedback for the Treatment of Autism Spectrum Disorder: An Exploratory Study. *Applied Psychophysiology and Biofeedback*, *39*(3–4), 237–257. https://doi.org/10.1007/s10484-014-9264-7

Sollenberger, N. A., Kimbler, A., Cummings, L. R., Pettit, J. W., Hayes, T., Patriarca, G. C., Vázquez, A. L., Shumway, P., Yegüez, C. E., Rey, Y., Mattfeld, A. T., & McMakin, D. L. (2023). Sleep fails to depotentiate amygdala-reactivity to negative emotional stimuli in youth with elevated symptoms of anxiety. *Cognitive, Affective, & Behavioral Neuroscience*, *23*(2), 415–426. https://doi.org/10.3758/s13415-023-01066-8

Sollenberger, N. A., Sequeira, S., Forbes, E. E., Siegle, G. J., Silk, J. S., Ladouceur, C. D., Ryan, N. D., Dahl, R. E., Mattfeld, A. T., & McMakin, D. L. (2023). More time awake after sleep onset is linked to reduced ventral striatum response to rewards in youth with anxiety. *Journal of Child Psychology and Psychiatry*, *64*(1), 83–90. https://doi.org/10.1111/jcpp.13669

Solomon, M., Ozonoff, S. J., Ursu, S., Ravizza, S., Cummings, N., Ly, S., & Carter, C. S. (2009). The neural substrates of cognitive control deficits in autism spectrum disorders. *Neuropsychologia*, *47*(12), 2515–2526. https://doi.org/10.1016/J.NEUROPSYCHOLOGIA.2009.04.019

Solomon, M., Ragland, J. D., Niendam, T. A., Lesh, T. A., Beck, J. S., Matter, J. C., Frank, M. J., & Carter, C. S. (2015). Atypical Learning in Autism Spectrum Disorders: A Functional Magnetic Resonance Imaging Study of Transitive Inference. *Journal of the American Academy of Child & Adolescent Psychiatry*, *54*(11), 947–955. https://doi.org/10.1016/j.jaac.2015.08.010

Song, K., Zhang, J.-L., Zhou, N., Fu, Y., Zou, B., Xu, L.-X., Wang, Z., Li, X., Zhao, Y., Potenza, M., Fang, X., & Zhang, J.-T. (2023). Youth Screen Media Activity Patterns and Associations With Behavioral Developmental Measures and Resting-state Brain Functional Connectivity. *Journal of the American Academy of Child & Adolescent Psychiatry*, *62*(9), 1051–1063. https://doi.org/10.1016/j.jaac.2023.02.014

Song, Y., Zhu, H., Wu, Z., Hu, W., Zhang, S., Zhou, Y., Peng, Y., Yang, Y., Li, W., Shi, H., Yang, G., Zhang, Y., & Lv, L. (2025). Functional connectivity changes in the prefrontal-thalamic-cerebellar circuit in adolescents with first-episode Schizophrenia. *European Child & Adolescent Psychiatry*, *34*(8), 2463–2472. https://doi.org/10.1007/s00787-025-02656-2

Sovatzidi, G., Vasilakakis, M., & Iakovidis, D. K. (2022). *Constructive Fuzzy Cognitive Map for Depression Severity Estimation*. https://doi.org/10.3233/SHTI220506

Speth, T., Rusak, B., Perrot, T., Cote, K., & Corkum, P. (2023). Sleep Architecture and EEG Power Spectrum Following Cumulative Sleep Restriction: A Comparison between Typically Developing Children and Children with ADHD. *Brain Sciences*, *13*(5), 772. https://doi.org/10.3390/brainsci13050772

Spicer, J., Werner, E., Zhao, Y., Choi, C. W., Lopez-Pintado, S., Feng, T., Altemus, M., Gyamfi, C., & Monk, C. (2013). Ambulatory assessments of psychological and peripheral stress-markers predict birth outcomes in teen pregnancy. *Journal of Psychosomatic Research*, *75*(4), 305–313. https://doi.org/10.1016/j.jpsychores.2013.07.001

Spinelli, S., Vasa, R. A., Joel, S., Nelson, T. E., Pekar, J. J., & Mostofsky, S. H. (2011). Variability in post‐error behavioral adjustment is associated with functional abnormalities in the temporal cortex in children with ADHD. *Journal of Child Psychology and Psychiatry*, *52*(7), 808–816. https://doi.org/10.1111/j.1469-7610.2010.02356.x

Srivastava, S., Jo, B., Zhang, B., Frazier, T., Gallagher, A. S., Peck, F., Levin, A. R., Mondal, S., Li, Z., Filip-Dhima, R., Geisel, G., Dies, K. A., Diplock, A., Eng, C., Hanna, R., Sahin, M., Hardan, A., Sahin, M., Eng, C., … Frazier, T. (2022). A randomized controlled trial of everolimus for neurocognitive symptoms in PTEN hamartoma tumor syndrome. *Human Molecular Genetics*, *31*(20), 3393–3404. https://doi.org/10.1093/hmg/ddac111

Srivastava, S., Scherrer, B., Prohl, A. K., Filip-Dhima, R., Kapur, K., Kolevzon, A., Buxbaum, J. D., Berry-Kravis, E., Soorya, L., Thurm, A., Powell, C. M., Bernstein, J. A., Warfield, S. K., & Sahin, M. (2019). Volumetric Analysis of the Basal Ganglia and Cerebellar Structures in Patients with Phelan-McDermid Syndrome. *Pediatric Neurology*, *90*, 37–43. https://doi.org/10.1016/j.pediatrneurol.2018.09.008

Steingard, R. J., Renshaw, P. F., Hennen, J., Lenox, M., Cintron, C. B., Young, A. D., Connor, D. F., Au, T. H., & Yurgelun-Todd, D. A. (2002). Smaller frontal lobe white matter volumes in depressed adolescents. *Biological Psychiatry*, *52*(5), 413–417. https://doi.org/10.1016/S0006-3223(02)01393-8

Steingard, R. J., Yurgelun-Todd, D. A., Hennen, J., Moore, J. C., Moore, C. M., Vakili, K., Young, A. D., Katic, A., Beardslee, W. R., & Renshaw, P. F. (2000). Increased orbitofrontal cortex levels of choline in depressed adolescents as detected by in vivo proton magnetic resonance spectroscopy. *Biological Psychiatry*, *48*(11), 1053–1061. https://doi.org/10.1016/S0006-3223(00)00942-2

Sterzer, P., Stadler, C., Poustka, F., & Kleinschmidt, A. (2007). A structural neural deficit in adolescents with conduct disorder and its association with lack of empathy. *NeuroImage*, *37*(1), 335–342. https://doi.org/10.1016/j.neuroimage.2007.04.043

Stewart Campbell, A., Needham, B. D., Meyer, C. R., Tan, J., Conrad, M., Preston, G. M., Bolognani, F., Rao, S. G., Heussler, H., Griffith, R., Guastella, A. J., Janes, A. C., Frederick, B., Donabedian, D. H., & Mazmanian, S. K. (2022). Safety and target engagement of an oral small-molecule sequestrant in adolescents with autism spectrum disorder: an open-label phase 1b/2a trial. *Nature Medicine*, *28*(3), 528–534. https://doi.org/10.1038/s41591-022-01683-9

Stohr, G. M., Harrewijn, A., Morales, S., Zeytinoglu, S., Laky, Z. E., Khosravi, P., Cardinale, E. M., Lorenzo, N., Stoddard, J., Pine, D. S., Fox, N. A., Brotman, M. A., & Haller, S. P. (2025). Adolescent cognitive control processing is associated with anxiety in young adulthood during the COVID-19 pandemic. *Cognitive, Affective, & Behavioral Neuroscience*, *25*(5), 1485–1495. https://doi.org/10.3758/s13415-025-01293-1

Straub, J., Metzger, C. D., Plener, P. L., Koelch, M. G., Groen, G., & Abler, B. (2017). Successful group psychotherapy of depression in adolescents alters fronto-limbic resting-state connectivity. *Journal of Affective Disorders*, *209*, 135–139. https://doi.org/10.1016/J.JAD.2016.11.024

Stroganova, T. A., Butorina, A. v., Sysoeva, O. v., Prokofyev, A. O., Nikolaeva, A. Yu., Tsetlin, M. M., & Orekhova, E. v. (2015). Altered modulation of gamma oscillation frequency by speed of visual motion in children with autism spectrum disorders. *Journal of Neurodevelopmental Disorders*, *7*(1), 21. https://doi.org/10.1186/s11689-015-9121-x

Stunden, C., Stratton, K., Zakani, S., & Jacob, J. (2021). Comparing a Virtual Reality–Based Simulation App (VR-MRI) With a Standard Preparatory Manual and Child Life Program for Improving Success and Reducing Anxiety During Pediatric Medical Imaging: Randomized Clinical Trial. *Journal of Medical Internet Research*, *23*(9), e22942. https://doi.org/10.2196/22942

Sugranyes, G., de la Serna, E., Borras, R., Sanchez-Gistau, V., Pariente, J. C., Romero, S., Baeza, I., Díaz-Caneja, C. M., Rodriguez-Toscano, E., Moreno, C., Bernardo, M., Moreno, D., Vieta, E., & Castro-Fornieles, J. (2017). Clinical, Cognitive, and Neuroimaging Evidence of a Neurodevelopmental Continuum in Offspring of Probands With Schizophrenia and Bipolar Disorder. *Schizophrenia Bulletin*, *43*(6), 1208–1219. https://doi.org/10.1093/schbul/sbx002

Sulheim, D., Fagermoen, E., Winger, A., Andersen, A. M., Godang, K., Müller, F., Rowe, P. C., Saul, J. P., Skovlund, E., Øie, M. G., & Wyller, V. B. (2014). Disease Mechanisms and Clonidine Treatment in Adolescent Chronic Fatigue Syndrome. *JAMA Pediatrics*, *168*(4), 351. https://doi.org/10.1001/jamapediatrics.2013.4647

Sun, Y., Duan, M., An, L., Liu, S., & Ming, D. (2023). Abnormal attentional bias in individuals with suicidal ideation during an emotional Stroop task: an event-related potential study. *Frontiers in Psychiatry*, *14*. https://doi.org/10.3389/fpsyt.2023.1118602

Sun, Y., Wu, Z., Guo, L., Wang, W., & Ma, X. (2025). Risk factor analysis and establishment of a predictive model for epilepsy comorbid with depression. *PLOS One*, *20*(9), e0331441. https://doi.org/10.1371/journal.pone.0331441

Suo, X., Lei, D., Li, K., Chen, F., Li, F., Li, L., Huang, X., Lui, S., Li, L., Kemp, G. J., & Gong, Q. (2015). Disrupted brain network topology in pediatric posttraumatic stress disorder: A resting‐state fMRI study. *Human Brain Mapping*, *36*(9), 3677–3686. https://doi.org/10.1002/hbm.22871

Suor, J. H., Granros, M., Calentino, A. E., Phan, K. L., & Burkhouse, K. L. (2023). The interplay of childhood maltreatment and maternal depression in relation to the reward positivity in youth. *Development and Psychopathology*, *35*(1), 168–178. https://doi.org/10.1017/S0954579421000857

Swartz, J. R., Hariri, A. R., & Williamson, D. E. (2017). An epigenetic mechanism links socioeconomic status to changes in depression-related brain function in high-risk adolescents. *Molecular Psychiatry*, *22*(2), 209–214. https://doi.org/10.1038/mp.2016.82

Swartz, J. R., Williamson, D. E., & Hariri, A. R. (2015). Developmental Change in Amygdala Reactivity During Adolescence: Effects of Family History of Depression and Stressful Life Events. *American Journal of Psychiatry*, *172*(3), 276–283. https://doi.org/10.1176/appi.ajp.2014.14020195

Syu, G.-D., Cheng, Y., Chen, K., Yang, C.-Y., Sutandy, F. X. R., Liu, C.-C., Chen, C.-S., & Shih, J. C. (2025). Autoantibody profiling of autism spectrum disorder patients with monoamine oxidase a deficiency. *Brain, Behavior, & Immunity - Health*, *47*, 101039. https://doi.org/10.1016/j.bbih.2025.101039

Szobot, C., Roman, T., Cunha, R., Acton, P., Hutz, M., & Rohde, L. A. (2005). Brain perfusion and dopaminergic genes in boys with attention‐deficit/hyperactivity disorder. *American Journal of Medical Genetics Part B: Neuropsychiatric Genetics*, *132B*(1), 53–58. https://doi.org/10.1002/ajmg.b.30096

Tadayonnejad, R., Majid, D.-A., Tsolaki, E., Rane, R., Wang, H., Moody, T. D., Pauli, W. M., Pouratian, N., Bari, A. A., Murray, S. B., O’Doherty, J. P., & Feusner, J. D. (2022). Mesolimbic Neurobehavioral Mechanisms of Reward Motivation in Anorexia Nervosa: A Multimodal Imaging Study. *Frontiers in Psychiatry*, *13*. https://doi.org/10.3389/fpsyt.2022.806327

Tan, J., Zhan, Y., Tang, Y., Bao, W., & Tian, Y. (2024). EEG decoding for effects of visual joint attention training on ASD patients with interpretable and lightweight convolutional neural network. *Cognitive Neurodynamics*, *18*(3), 947–960. https://doi.org/10.1007/s11571-023-09947-x

Tan, S. M. X., Yee, J. Y., Budhraja, S., Singh, B., Doborjeh, Z., Doborjeh, M., Kasabov, N., Lai, E., Sumich, A., Lee, J., & Goh, W. W. bin. (2023). RNA-sequencing of peripheral whole blood of individuals at ultra-high-risk for psychosis – A longitudinal perspective. *Asian Journal of Psychiatry*, *89*, 103796. https://doi.org/10.1016/j.ajp.2023.103796

Tan, S. Y., Poh, B. K., Sekartini, R., Rojroongwasinkul, N., Tran, T. N., Wong, J. E., Novita Chandra, D., Pongcharoen, T., Tran, K. van, Actis-Goretta, L., Vonk, M. M., Ng, S. A., Parikh, P., & Khouw, I. (2024). South East Asian Nutrition Surveys (SEANUTS) II – a multi-country evaluation of nutrition and lifestyle indicators in children aged 12 years and below: rationale and design. *Public Health Nutrition*, *27*(1), e150. https://doi.org/10.1017/S1368980024000910

Tang, A., Miskovic, V., Lahat, A., Tanaka, M., MacMillan, H., van Lieshout, R. J., & Schmidt, L. A. (2018). Trajectories of resting frontal brain activity and psychopathology in female adolescents exposed to child maltreatment. *Developmental Psychobiology*, *60*(1), 67–77. https://doi.org/10.1002/dev.21585

Tarbell, S. E., Millar, A., Laudenslager, M., Palmer, C., & Fortunato, J. E. (2017). Anxiety and physiological responses to the Trier Social Stress Test for Children in adolescents with cyclic vomiting syndrome. *Autonomic Neuroscience*, *202*, 79–85. https://doi.org/10.1016/j.autneu.2016.08.010

Teicher, M. H., Dumont, N. L., Ito, Y., Vaituzis, C., Giedd, J. N., & Andersen, S. L. (2004). Childhood neglect is associated with reduced corpus callosum area. *Biological Psychiatry*, *56*(2), 80–85. https://doi.org/10.1016/j.biopsych.2004.03.016

Teng, Z., Xu, X., Chen, X., Qiu, Y., Li, S., Chen, J., Tang, H., Xiang, H., Wang, B., Tan, Y., & Wu, H. (2024). Increased circulating cell-free mitochondrial DNA in plasma of first-diagnosed drug-naïve bipolar disorder patients: A case-control and 4-week follow-up study. *Journal of Affective Disorders*, *355*, 378–384. https://doi.org/10.1016/J.JAD.2024.03.113

Terczynska, M., Bargiel, W., Grabarczyk, M., Kozlowski, T., Zakowicz, P., Bojarski, D., Wasicka-Przewozna, K., Kapelski, P., Rajewska-Rager, A., & Skibinska, M. (2025). Circulating Growth Factors and Cytokines Correlate with Temperament and Character Dimensions in Adolescents with Mood Disorders. *Brain Sciences*, *15*(2), 121. https://doi.org/10.3390/brainsci15020121

Tervo-Clemmens, B., Quach, A., Luna, B., Foran, W., Chung, T., de Bellis, M. D., & Clark, D. B. (2017). Neural Correlates of Rewarded Response Inhibition in Youth at Risk for Problematic Alcohol Use. *Frontiers in Behavioral Neuroscience*, *11*. https://doi.org/10.3389/fnbeh.2017.00205

Tesler, N., Gerstenberg, M., Franscini, M., Jenni, O. G., Walitza, S., & Huber, R. (2016). Increased frontal sleep slow wave activity in adolescents with major depression. *NeuroImage: Clinical*, *10*, 250–256. https://doi.org/10.1016/J.NICL.2015.10.014

Thai, M., Başgöze, Z., Klimes-Dougan, B., Mueller, B. A., Fiecas, M., Lim, K. O., Albott, C. S., & Cullen, K. R. (2020). Neural and Behavioral Correlates of Clinical Improvement to Ketamine in Adolescents With Treatment Resistant Depression. *Frontiers in Psychiatry*, *11*. https://doi.org/10.3389/fpsyt.2020.00820

Thatcher, , Dawn L, & Clark, , Duncan Β. (2006). Cardiovascular risk factors in adolescents with alcohol use disorders. *International Journal of Adolescent Medicine and Health*, *18*(1), 151–158. https://doi.org/10.1515/IJAMH.2006.18.1.151

Thomas, J. J., Holsen, L., van de Water, A. L., Becker, K. R., Breithaupt, L., Burton-Murray, H., Asanza, E., Gydus, J., Palmer, L. P., Stern, C. M., Freizinger, M., Shrier, L. A., Bern, E. M., Deckersbach, T., Misra, M., Eddy, K. T., Micali, N., & Lawson, E. (2025). Neural Response to Food Cues in Avoidant/Restrictive Food Intake Disorder. *JAMA Network Open*, *8*(2), e2460101. https://doi.org/10.1001/jamanetworkopen.2024.60101

Thompson, D. K., Cai, S., Kelly, C. E., Alexander, B., Matthews, L. G., Mainzer, R., Doyle, L. W., Cheong, J. L. Y., Inder, T. E., Yang, J. Y. M., & Anderson, P. J. (2025). Brain volume and neurodevelopment at 13 years following sepsis in very preterm infants. *Pediatric Research*, *97*(2), 744–750. https://doi.org/10.1038/s41390-024-03407-w

Tobiasova, Z., van der Lingen, K. H. B., Scahill, L., Leckman, J. F., Zhang, Y., Chae, W., McCracken, J. T., McDougle, C. J., Vitiello, B., Tierney, E., Aman, M. G., Arnold, L. E., Katsovich, L., Hoekstra, P. J., Volkmar, F., Bothwell, A. L. M., & Kawikova, I. (2011). Risperidone-Related Improvement of Irritability in Children with Autism Is not Associated with Changes in Serum of Epidermal Growth Factor and Interleukin-13. *Journal of Child and Adolescent Psychopharmacology*, *21*(6), 555–564. https://doi.org/10.1089/cap.2010.0134

Tokatly Latzer, I., Hanson, E., Bertoldi, M., DiBacco, M. L., Aygun, D., Afacan, O., García-Cazorla, À., Juliá-Palacios, N., Opladen, T., Hübschmann, O. K., Jeltsch, K., Aden, P., Oppebøen, M., Rotenberg, A., Tsuboyama, M., Roullet, J.-B., & Pearl, P. L. (2025). The neuropsychological profile of SSADH deficiency, a neurotransmitter disorder of GABA metabolism. *Molecular Genetics and Metabolism*, *144*(3), 109051. https://doi.org/10.1016/j.ymgme.2025.109051

Tokatly Latzer, I., Hanson, E., Bertoldi, M., García‐Cazorla, À., Tsuboyama, M., MacMullin, P., Rotenberg, A., Roullet, J., & Pearl, P. L. (2023). Autism spectrum disorder and <scp>GABA</scp> levels in children with succinic semialdehyde dehydrogenase deficiency. *Developmental Medicine & Child Neurology*, *65*(12), 1596–1606. https://doi.org/10.1111/dmcn.15659

Tonon, A. C., Constantino, D. B., Amando, G. R., Abreu, A. C., Francisco, A. P., de Oliveira, M. A. B., Pilz, L. K., Xavier, N. B., Rohrsetzer, F., Souza, L., Piccin, J., Caye, A., Petresco, S., Manfro, P. H., Pereira, R., Martini, T., Kohrt, B. A., Fisher, H. L., Mondelli, V., … Hidalgo, M. P. L. (2022). Sleep disturbances, circadian activity, and nocturnal light exposure characterize high risk for and current depression in adolescence. *Sleep*, *45*(7). https://doi.org/10.1093/sleep/zsac104

Toyota, E., Mackinley, M., Silva, A. M., Jiang, Y., Dalal, T. C., Nettekoven, C., & Palaniyappan, L. (2025). Cerebellum as a neural substrate for impoverishment in early psychosis. *Neuropsychologia*, *210*, 109094. https://doi.org/10.1016/j.neuropsychologia.2025.109094

Trickett, P. K., Noll, J. G., Susman, E. J., Shenk, C. E., & Putnam, F. W. (2010). Attenuation of cortisol across development for victims of sexual abuse. *Development and Psychopathology*, *22*(1), 165–175. https://doi.org/10.1017/S0954579409990332

Tseng, Y.-L., Lee, C.-H., Chiu, Y.-N., Tsai, W.-C., Wang, J.-S., Wu, W.-C., & Chien, Y.-L. (2024). Characterizing Autism Spectrum Disorder Through Fusion of Local Cortical Activation and Global Functional Connectivity Using Game-Based Stimuli and a Mobile EEG System. *IEEE Transactions on Neural Systems and Rehabilitation Engineering*, *32*, 3026–3035. https://doi.org/10.1109/TNSRE.2024.3417210

Tu, F. F., Hellman, K. M., Darnell, S. E., Harber, K. A., Bohnert, A. M., Singh, L., & Walker, L. S. (2024). A multidimensional appraisal of early menstrual pain experience. *American Journal of Obstetrics and Gynecology*, *230*(5), 550.e1-550.e10. https://doi.org/10.1016/j.ajog.2024.01.017

Tupler, L. A., & de Bellis, M. D. (2006). Segmented Hippocampal Volume in Children and Adolescents with Posttraumatic Stress Disorder. *Biological Psychiatry*, *59*(6), 523–529. https://doi.org/10.1016/j.biopsych.2005.08.007

Tweedie-Cullen, R. Y., Leong, K., Wilson, B. C., Derraik, J. G. B., Albert, B. B., Monk, R., Vatanen, T., Creagh, C., Depczynski, M., Edwards, T., Beck, K., Thabrew, H., O’Sullivan, J. M., & Cutfield, W. S. (2024). Protocol for the Gut Bugs in Autism Trial: a double-blind randomised placebo-controlled trial of faecal microbiome transfer for the treatment of gastrointestinal symptoms in autistic adolescents and adults. *BMJ Open*, *14*(2), e074625. https://doi.org/10.1136/bmjopen-2023-074625

Uddin, L. Q., Supekar, K., Lynch, C. J., Khouzam, A., Phillips, J., Feinstein, C., Ryali, S., & Menon, V. (2013). Salience Network–Based Classification and Prediction of Symptom Severity in Children With Autism. *JAMA Psychiatry*, *70*(8), 869. https://doi.org/10.1001/jamapsychiatry.2013.104

Uddin, L. Q., Supekar, K., & Menon, V. (2013). Reconceptualizing functional brain connectivity in autism from a developmental perspective. *Frontiers in Human Neuroscience*, *7*. https://doi.org/10.3389/fnhum.2013.00458

Uhlhaas, P. J., Gajwani, R., Gross, J., Gumley, A. I., Lawrie, S. M., & Schwannauer, M. (2017). The Youth Mental Health Risk and Resilience Study (YouR-Study). *BMC Psychiatry*, *17*(1), 43. https://doi.org/10.1186/s12888-017-1206-5

Valadez, E. A., Troller‐Renfree, S. v., Buzzell, G. A., Henderson, H. A., Chronis‐Tuscano, A., Pine, D. S., & Fox, N. A. (2021). Behavioral inhibition and dual mechanisms of anxiety risk: Disentangling neural correlates of proactive and reactive control. *JCPP Advances*, *1*(2). https://doi.org/10.1002/jcv2.12022

Valles‐Capetillo, E., Kurtz, M. R., & Kana, R. K. (2025). The Role of the Brain’s Pragmatic Language Network in Reading Comprehension in Autistic Children. *Autism Research*, *18*(8), 1550–1562. https://doi.org/10.1002/aur.70076

van den Bulk, B. G., Meens, P. H. F., van Lang, N. D. J., de Voogd, E. L., van der Wee, N. J. A., Rombouts, S. A. R. B., Crone, E. A., & Vermeiren, R. R. J. M. (2014). Amygdala activation during emotional face processing in adolescents with affective disorders: the role of underlying depression and anxiety symptoms. *Frontiers in Human Neuroscience*, *8*. https://doi.org/10.3389/fnhum.2014.00393

van der Venne, P., Balint, A., Drews, E., Parzer, P., Resch, F., Koenig, J., & Kaess, M. (2021). Pain sensitivity and plasma beta-endorphin in adolescent non-suicidal self-injury. *Journal of Affective Disorders*, *278*, 199–208. https://doi.org/10.1016/j.jad.2020.09.036

van Griensven, F., Naorat, S., Kilmarx, P. H., Jeeyapant, S., Manopaiboon, C., Chaikummao, S., Jenkins, R. A., Uthaivoravit, W., Wasinrapee, P., Mock, P. A., & Tappero, J. W. (2006). Palmtop-assisted Self-Interviewing for the Collection of Sensitive Behavioral Data: Randomized Trial with Drug Use Urine Testing. *American Journal of Epidemiology*, *163*(3), 271–278. https://doi.org/10.1093/aje/kwj038

Vandewouw, M. M., Brian, J., Crosbie, J., Schachar, R. J., Iaboni, A., Georgiades, S., Nicolson, R., Kelley, E., Ayub, M., Jones, J., Taylor, M. J., Lerch, J. P., Anagnostou, E., & Kushki, A. (2023). Identifying Replicable Subgroups in Neurodevelopmental Conditions Using Resting-State Functional Magnetic Resonance Imaging Data. *JAMA Network Open*, *6*(3), e232066. https://doi.org/10.1001/jamanetworkopen.2023.2066

Vandewouw, M. M., Safar, K., Mossad, S. I., Lu, J., Lerch, J. P., Anagnostou, E., & Taylor, M. J. (2021). Do shapes have feelings? Social attribution in children with autism spectrum disorder and attention-deficit/hyperactivity disorder. *Translational Psychiatry*, *11*(1), 493. https://doi.org/10.1038/s41398-021-01625-y

Vaquero, L., Groves, K., Muñoz-Vidal, E. L., James, K., Marlor, J. L., McIntyre, C., Ostia, L., Sirota, S., Shields, L., Degé, F., García-Mingo, E., & Ripollés, P. (2025). Studying the effects of digital arts-based interventions on teenagers’ social media usage, brain connectivity, and mental health: study protocol of the SMART project. *European Journal of Psychotraumatology*, *16*(1). https://doi.org/10.1080/20008066.2025.2550079

Veer, I. M., Jetzschmann, P., Garbusow, M., Nebe, S., Frank, R., Kuitunen-Paul, S., Sebold, M., Ripke, S., Heinz, A., Friedel, E., Smolka, M. N., & Walter, H. (2019). Nucleus accumbens connectivity at rest is associated with alcohol consumption in young male adults. *European Neuropsychopharmacology*, *29*(12), 1476–1485. https://doi.org/10.1016/J.EURONEURO.2019.10.008

Viding, E., Sebastian, C. L., Dadds, M. R., Lockwood, P. L., Cecil, C. A. M., de Brito, S. A., & McCrory, E. J. (2012). Amygdala Response to Preattentive Masked Fear in Children With Conduct Problems: The Role of Callous-Unemotional Traits. *American Journal of Psychiatry*, *169*(10), 1109–1116. https://doi.org/10.1176/appi.ajp.2012.12020191

Villa, L. M., Goodyer, I. M., Tait, R., Kelvin, R., Reynolds, S., Wilkinson, P. O., & Suckling, J. (2020). Cognitive behavioral therapy may have a rehabilitative, not normalizing, effect on functional connectivity in adolescent depression. *Journal of Affective Disorders*, *268*, 1–11. https://doi.org/10.1016/j.jad.2020.01.103

Villemonteix, T., Rogers, J. C., Courbet, O., Gonzalez-Madruga, K., Kohls, G., Raschle, N. M., Stadler, C., Konrad, K., Freitag, C. M., Fairchild, G., & de Brito, S. A. (2022). Sex matters: association between callous-unemotional traits and uncinate fasciculus microstructure in youths with conduct disorder. *Brain Imaging and Behavior*, *16*(1), 263–269. https://doi.org/10.1007/s11682-021-00499-4

Vogan, V. M., Francis, K. E., Morgan, B. R., Smith, M. lou, & Taylor, M. J. (2018). Load matters: neural correlates of verbal working memory in children with autism spectrum disorder. *Journal of Neurodevelopmental Disorders*, *10*(1), 19. https://doi.org/10.1186/s11689-018-9236-y

Volkow, N. D., Koob, G. F., Croyle, R. T., Bianchi, D. W., Gordon, J. A., Koroshetz, W. J., Pérez-Stable, E. J., Riley, W. T., Bloch, M. H., Conway, K., Deeds, B. G., Dowling, G. J., Grant, S., Howlett, K. D., Matochik, J. A., Morgan, G. D., Murray, M. M., Noronha, A., Spong, C. Y., … Weiss, S. R. B. (2018). The conception of the ABCD study: From substance use to a broad NIH collaboration. *Developmental Cognitive Neuroscience*, *32*, 4–7. https://doi.org/10.1016/j.dcn.2017.10.002

von Rhein, D., Mennes, M., van Ewijk, H., Groenman, A. P., Zwiers, M. P., Oosterlaan, J., Heslenfeld, D., Franke, B., Hoekstra, P. J., Faraone, S. v., Hartman, C., & Buitelaar, J. (2015). The NeuroIMAGE study: a prospective phenotypic, cognitive, genetic and MRI study in children with attention-deficit/hyperactivity disorder. Design and descriptives. *European Child & Adolescent Psychiatry*, *24*(3), 265–281. https://doi.org/10.1007/s00787-014-0573-4

Vreeker, A., Horsfall, M., Eikelenboom, M., Beerthuizen, A., Bergink, V., Boks, M. P. M., Hartman, C. A., de Koning, R., de Leeuw, M., Maciejewski, D. F., Penninx, B. W. J. H., & Hillegers, M. H. J. (2024). The Mood and Resilience in Offspring (MARIO) project: a longitudinal cohort study among offspring of parents with and without a mood disorder. *BMC Psychiatry*, *24*(1), 227. https://doi.org/10.1186/s12888-024-05555-z

Vulser, H., Lemaitre, H., Artiges, E., Miranda, R., Penttilä, J., Struve, M., Fadai, T., Kappel, V., Grimmer, Y., Goodman, R., Stringaris, A., Poustka, L., Conrod, P., Frouin, V., Banaschewski, T., Barker, G. J., Bokde, A. L. W., Bromberg, U., Büchel, C., … Stephens, D. (2015). Subthreshold Depression and Regional Brain Volumes in Young Community Adolescents. *Journal of the American Academy of Child & Adolescent Psychiatry*, *54*(10), 832–840. https://doi.org/10.1016/J.JAAC.2015.07.006

WABER, D. P., de MOOR, C., FORBES, P. W., ALMLI, C. R., BOTTERON, K. N., LEONARD, G., MILOVAN, D., PAUS, T., & RUMSEY, J. (2007). The NIH MRI study of normal brain development: Performance of a population based sample of healthy children aged 6 to 18 years on a neuropsychological battery. *Journal of the International Neuropsychological Society*, *13*(05). https://doi.org/10.1017/S1355617707070841

Wade, N. E., Bagot, K. S., Cota, C. I., Fotros, A., Squeglia, L. M., Meredith, L. R., & Jacobus, J. (2019). Orbitofrontal cortex volume prospectively predicts cannabis and other substance use onset in adolescents. *Journal of Psychopharmacology*, *33*(9), 1124–1131. https://doi.org/10.1177/0269881119855971

Wadhera, T., & Kakkar, D. (2020). Multiplex temporal measures reflecting neural underpinnings of brain functional connectivity under cognitive load in Autism Spectrum Disorder. *Neurological Research*, *42*(4), 327–337. https://doi.org/10.1080/01616412.2020.1726586

Walker, J. C., Teresi, G. I., Weisenburger, R. L., Segarra, J. R., Ojha, A., Kulla, A., Sisk, L., Gu, M., Spielman, D. M., Rosenberg-Hasson, Y., Maecker, H. T., Singh, M. K., Gotlib, I. H., & Ho, T. C. (2020). Study Protocol for Teen Inflammation Glutamate Emotion Research (TIGER). *Frontiers in Human Neuroscience*, *14*. https://doi.org/10.3389/fnhum.2020.585512

Waloszek, J. M., Byrne, M. L., Woods, M. J., Nicholas, C. L., Bei, B., Murray, G., Raniti, M., Allen, N. B., & Trinder, J. (2015). Early physiological markers of cardiovascular risk in community based adolescents with a depressive disorder. *Journal of Affective Disorders*, *175*, 403–410. https://doi.org/10.1016/J.JAD.2015.01.008

Walsh, K., Badour, C. L., Zuromski, K. L., Gilmore, A. K., Kilpatrick, D. G., Acierno, R., & Resnick, H. S. (2021). A secondary analysis of a brief video intervention on suicidal ideation among recent rape victims. *Psychological Services*, *18*(4), 703–708. https://doi.org/10.1037/ser0000495

Walsh, N. D., Dalgleish, T., Lombardo, M. v., Dunn, V. J., van Harmelen, A.-L., Ban, M., & Goodyer, I. M. (2014). General and specific effects of early-life psychosocial adversities on adolescent grey matter volume. *NeuroImage: Clinical*, *4*, 308–318. https://doi.org/10.1016/j.nicl.2014.01.001

Wan, X., Wu, Y., Jiang, W., Lu, X., Tang, Y., Yuan, X., Huang, L., & Hu, M. (2024). Cortical functional mechanisms in emotional cognitive tasks in first-episode, drug-naïve with major depressive disorder: A fNIRS study. *Journal of Affective Disorders*, *362*, 698–705. https://doi.org/10.1016/j.jad.2024.07.113

Wang, A. T., Lee, S. S., Sigman, M., & Dapretto, M. (2006). Neural basis of irony comprehension in children with autism: the role of prosody and context. *Brain*, *129*(4), 932–943. https://doi.org/10.1093/brain/awl032

Wang, C., Qu, Y., Shen, X., Tang, X., Tong, G., Wati, M., Naibi, M., Zhang, C., & Zou, S. (2025). Exploration of neurometabolic alterations in adolescent patients with bipolar depression and non-suicidal self-injury based on proton magnetic resonance spectroscopy. *Frontiers in Psychiatry*, *15*. https://doi.org/10.3389/fpsyt.2024.1474170

Wang, J., Zhang, C., Zhang, Y., Liu, Y., Zhang, J., Fang, X., Xia, W., Xie, Y., Lan, Z., Wang, J., Lu, M., & Chen, J. (2025). Protocol for a nested case-control study: identifying neuroimaging biomarkers for the progression of subclinical depression and qi-stagnation constitution to major depressive disorder in adolescents. *Frontiers in Psychiatry*, *15*. https://doi.org/10.3389/fpsyt.2024.1516846

Wang, K., He, Q., Zhu, X., Hu, Y., Yao, Y., Hommel, B., Beste, C., Liu, J., Yang, Y., & Zhang, W. (2022). Smaller putamen volumes are associated with greater problems in external emotional regulation in depressed adolescents with nonsuicidal self-injury. *Journal of Psychiatric Research*, *155*, 338–346. https://doi.org/10.1016/J.JPSYCHIRES.2022.09.014

Wang, K., Liu, S., Huang, D., Guan, X., Chen, N., Xiu, M., Liu, D., & Huang, Y. (2024). Onset age moderates the associations between neutrophil-to-lymphocyte ratio and clinical symptoms in first-episode patients with schizophrenia. *Schizophrenia*, *10*(1), 110. https://doi.org/10.1038/s41537-024-00522-9

Wang, L., Zhao, P., Zhang, J., Zhang, R., Liu, J., Duan, J., Zhang, X., Zhu, R., & Wang, F. (2023). Functional connectivity between the cerebellar vermis and cerebrum distinguishes early treatment response for major depressive episodes in adolescents. *Journal of Affective Disorders*, *339*, 256–263. https://doi.org/10.1016/j.jad.2023.07.054

Wang, L.-J., Li, S.-C., Li, S.-W., Kuo, H.-C., Lee, S.-Y., Huang, L.-H., Chin, C.-Y., & Yang, C.-Y. (2022). Gut microbiota and plasma cytokine levels in patients with attention-deficit/hyperactivity disorder. *Translational Psychiatry*, *12*(1), 76. https://doi.org/10.1038/s41398-022-01844-x

Wang, Q., Gao, Y., Shangguan, Q., Zeng, Y., Jin, W., Wang, P., Hu, Y., & Chen, X. (2026). Morphological and genetic signatures of hypomania: A multisite exploration of reward circuitry and transcriptomic profiles. *Journal of Affective Disorders*, *393*, 120312. https://doi.org/10.1016/j.jad.2025.120312

Wang, S., Fan, S., Gan, Y., Zhang, Y., Gao, Y., Xue, T., Xie, H., Ma, R., Zhang, Q., Zhao, B., Wang, Y., Zhu, G., Yang, A., Jiang, Y., Meng, F., & Zhang, J. (2024). Efficacy and safety of combined deep brain stimulation with capsulotomy for comorbid motor and psychiatric symptoms in Tourette’s syndrome: Experience and evidence. *Asian Journal of Psychiatry*, *94*, 103960. https://doi.org/10.1016/j.ajp.2024.103960

Wang, X., Kong, Y., Xiang, J., Jiang, Z., Wang, Y., Chen, X., Wan, L., Hong, S., & Kuang, L. (2025). Transforming growth factor-β superfamily members as potential biomarkers for adolescent major depressive disorder. *Frontiers in Psychiatry*, *16*. https://doi.org/10.3389/fpsyt.2025.1655332

Wang, Y., Fan, L., He, Y., Yuan, L., Li, Z., Zheng, W., Tang, J., Li, C., Jin, K., Liu, W., Chen, X., Ouyang, L., & Ma, X. (2024). Compensatory thickening of cortical thickness in early stage of schizophrenia. *Cerebral Cortex*, *34*(6). https://doi.org/10.1093/cercor/bhae255

Wang, Y., Li, J., Zeng, L., Wang, H., Yang, T., Shao, Y., & Weng, X. (2022). Open Eyes Increase Neural Oscillation and Enhance Effective Brain Connectivity of the Default Mode Network: Resting-State Electroencephalogram Research. *Frontiers in Neuroscience*, *16*. https://doi.org/10.3389/fnins.2022.861247

Wang, Y., Wang, J., Su, W., Hu, H., Xia, M., Zhang, T., Xu, L., Zhang, X., Taylor, H., Osipowicz, K., Young, I. M., Lin, Y. H., Nicholas, P., Tanglay, O., Sughrue, M. E., Tang, Y., & Doyen, S. (2023). Symptom-circuit mappings of the schizophrenia connectome. *Psychiatry Research*, *323*, 115122. https://doi.org/10.1016/J.PSYCHRES.2023.115122

Wang, Y., Wang, Y., Zhu, Z., Xia, Z., Wang, N., Li, M., Jiao, Z., Pan, N., Zhang, Y., Chen, Q., Ke, X., & Zhang, W. (2025). Fundus Peripapillary Vascular Changes in Autism Spectrum Disorder: A Cross‐Sectional Study. *Autism Research*, *18*(9), 1775–1795. https://doi.org/10.1002/aur.70094

Wang, Y., Xie, M., Zheng, L., Ma, J., Wang, M., & Zhang, L. (2024). Associations between parental rearing style and amygdala and hippocampal subfield abnormalities in drug-naive females with anorexia nervosa. *BMC Psychiatry*, *24*(1), 648. https://doi.org/10.1186/s12888-024-06120-4

Wang, Z., Zhu, Y., Li, C., Xin, X., Wang, G., Chen, J., Karina, S., & Tian, Y. (2025). Correlation between physical exercise levels, depressive symptoms, and sleep quality in college students: Evidence from electroencephalography. *Journal of Affective Disorders*, *369*, 789–799. https://doi.org/10.1016/j.jad.2024.10.043

Wannan, C. M. J., Nelson, B., Addington, J., Allott, K., Anticevic, A., Arango, C., Baker, J. T., Bearden, C. E., Billah, T., Bouix, S., Broome, M. R., Buccilli, K., Cadenhead, K. S., Calkins, M. E., Cannon, T. D., Cecci, G., Chen, E. Y. H., Cho, K. I. K., Choi, J., … Shenton, M. E. (2024). Accelerating Medicines Partnership® Schizophrenia (AMP® SCZ): Rationale and Study Design of the Largest Global Prospective Cohort Study of Clinical High Risk for Psychosis. *Schizophrenia Bulletin*, *50*(3), 496–512. https://doi.org/10.1093/schbul/sbae011

Watts, R., Archibald, T., Hembry, P., Howard, M., Kelly, C., Loomes, R., Markham, L., Moss, H., Munuve, A., Oros, A., Siddall, A., Rhind, C., Uddin, M., Ahmad, Z., Bryant-Waugh, R., & Hübel, C. (2023). The clinical presentation of avoidant restrictive food intake disorder in children and adolescents is largely independent of sex, autism spectrum disorder and anxiety traits. *eClinicalMedicine*, *63*, 102190. https://doi.org/10.1016/j.eclinm.2023.102190

Weeland, C. J., van den Heuvel, O. A., White, T., Tiemeier, H., & Vriend, C. (2022). Obsessive-compulsive symptoms and resting-state functional characteristics in pre-adolescent children from the general population. *Brain Imaging and Behavior*, *16*(6), 2715–2724. https://doi.org/10.1007/s11682-022-00732-8

Wei, H., & Jin, Z. (2024). EEG correlates of trait test anxiety in the flanker task for adolescents. *Neuroscience Letters*, *826*, 137725. https://doi.org/10.1016/j.neulet.2024.137725

Wei, H., Xin, Q., Cheng, Y., Lv, Z., He, W., Tan, M., Lin, M., Zheng, S., Guo, J., & Zhang, B. (2025). Neural responses to social decision-making and their associated factors among nonsuicidal self-injured adolescents: An event-related potentials study. *Journal of Psychiatric Research*, *184*, 350–358. https://doi.org/10.1016/j.jpsychires.2025.03.014

Wei, J., Zhang, Z., Yang, X., Zhao, L., Wang, M., Dou, Y., Yan, Y., Ni, R., Gong, M., Dong, Z., & Ma, X. (2024). Abnormal functional connectivity within the prefrontal cortex is associated with multiple plasma lipid species in major depressive disorder. *Journal of Affective Disorders*, *350*, 713–720. https://doi.org/10.1016/j.jad.2023.12.072

Wei, L., Han, X., Yu, X., Sun, Y., Ding, M., Du, Y., Jiang, W., Zhou, Y., & Wang, H. (2021). Brain controllability and morphometry similarity of internet gaming addiction. *Methods*, *192*, 93–102. https://doi.org/10.1016/J.YMETH.2020.08.005

Wei, Z., Yan, C., Cui, L., Zhao, X., & Liu, L. (2023). Physiological Responses during Parental Conflicts as Potential Biomarkers for Adolescent Depression. *Children*, *10*(7), 1195. https://doi.org/10.3390/children10071195

Weigard, A., McCurry, K. L., Shapiro, Z., Martz, M. E., Angstadt, M., Heitzeg, M. M., Dinov, I. D., & Sripada, C. (2023). Generalizable prediction of childhood ADHD symptoms from neurocognitive testing and youth characteristics. *Translational Psychiatry*, *13*(1), 225. https://doi.org/10.1038/s41398-023-02502-6

Weigensberg, M. J., Spruijt-Metz, D., Wen, C. K. F., Davis, J. N., Ávila, Q., Juarez, M., Brown-Wadé, N., & Lane, C. J. (2018). Protocol for the Imagine HEALTH Study: Guided imagery lifestyle intervention to improve obesity-related behaviors and salivary cortisol patterns in predominantly Latino adolescents. *Contemporary Clinical Trials*, *72*, 103–116. https://doi.org/10.1016/J.CCT.2018.07.009

Weissman, D. G., Jenness, J. L., Colich, N. L., Miller, A. B., Sambrook, K. A., Sheridan, M. A., & McLaughlin, K. A. (2020). Altered Neural Processing of Threat-Related Information in Children and Adolescents Exposed to Violence: A Transdiagnostic Mechanism Contributing to the Emergence of Psychopathology. *Journal of the American Academy of Child & Adolescent Psychiatry*, *59*(11), 1274–1284. https://doi.org/10.1016/j.jaac.2019.08.471

Weissman, D. G., Rosen, M. L., Colich, N. L., Sambrook, K. A., Lengua, L. J., Sheridan, M. A., & McLaughlin, K. A. (2022). Exposure to Violence as an Environmental Pathway Linking Low Socioeconomic Status with Altered Neural Processing of Threat and Adolescent Psychopathology. *Journal of Cognitive Neuroscience*, *34*(10), 1892–1905. https://doi.org/10.1162/jocn_a_01825

Weng, S. J., Wiggins, J. L., Peltier, S. J., Carrasco, M., Risi, S., Lord, C., & Monk, C. S. (2010). Alterations of resting state functional connectivity in the default network in adolescents with autism spectrum disorders. *Brain Research*, *1313*, 202–214. https://doi.org/10.1016/J.BRAINRES.2009.11.057

Westner, B. U., Bosch, E., Utzerath, C., Buitelaar, J., & de Lange, F. P. (2025). Typical neural adaptation for familiar images in autistic adolescents. *Imaging Neuroscience*, *3*. https://doi.org/10.1162/imag_a_00505

White, L. O., Ising, M., von Klitzing, K., Sierau, S., Michel, A., Klein, A. M., Andreas, A., Keil, J., Quintero, L., Müller‐Myhsok, B., Uhr, M., Gausche, R., Manly, J. T., Crowley, M. J., Kirschbaum, C., & Stalder, T. (2017). Reduced hair cortisol after maltreatment mediates externalizing symptoms in middle childhood and adolescence. *Journal of Child Psychology and Psychiatry*, *58*(9), 998–1007. https://doi.org/10.1111/jcpp.12700

White, M. G., Bogdan, R., Fisher, P. M., Muñoz, K. E., Williamson, D. E., & Hariri, A. R. (2012). *<scp>FKBP5</scp>* and emotional neglect interact to predict individual differences in amygdala reactivity. *Genes, Brain and Behavior*, *11*(7), 869–878. https://doi.org/10.1111/j.1601-183X.2012.00837.x

White, S. F., Marsh, A. A., Fowler, K. A., Schechter, J. C., Adalio, C., Pope, K., Sinclair, S., Pine, D. S., & Blair, R. J. R. (2012). Reduced Amygdala Response in Youths With Disruptive Behavior Disorders and Psychopathic Traits: Decreased Emotional Response Versus Increased Top-Down Attention to Nonemotional Features. *American Journal of Psychiatry*, *169*(7), 750–758. https://doi.org/10.1176/appi.ajp.2012.11081270

Whitley, C. B., Cleary, M., Eugen Mengel, K., Harmatz, P., Shapiro, E., Nestrasil, I., Haslett, P., Whiteman, D., & Alexanderian, D. (2018). Observational Prospective Natural History of Patients with Sanfilippo Syndrome Type B. *The Journal of Pediatrics*, *197*, 198-206.e2. https://doi.org/10.1016/J.JPEDS.2018.01.044

Whittle, S., Dennison, M., Vijayakumar, N., Simmons, J. G., Yücel, M., Lubman, D. I., Pantelis, C., & Allen, N. B. (2013). Childhood Maltreatment and Psychopathology Affect Brain Development During Adolescence. *Journal of the American Academy of Child & Adolescent Psychiatry*, *52*(9), 940-952.e1. https://doi.org/10.1016/J.JAAC.2013.06.007

Wiggins, J. L., Brotman, M. A., Adleman, N. E., Kim, P., Wambach, C. G., Reynolds, R. C., Chen, G., Towbin, K., Pine, D. S., & Leibenluft, E. (2017). Neural Markers in Pediatric Bipolar Disorder and Familial Risk for Bipolar Disorder. *Journal of the American Academy of Child & Adolescent Psychiatry*, *56*(1), 67–78. https://doi.org/10.1016/j.jaac.2016.10.009

Wilson, B. C., Derraik, J. G. B., Albert, B. B., Leong, K. S. W., Tweedie-Cullen, R. Y., Creagh, C., Depczynski, M., Edwards, T., Vatanen, T., Thabrew, H., Cutfield, W. S., & O’Sullivan, J. M. (2023). An open-label pilot trial of faecal microbiome transfer to restore the gut microbiome in anorexia nervosa: protocol. *BMJ Open*, *13*(7), e070616. https://doi.org/10.1136/bmjopen-2022-070616

Winsor, A. A., Richards, C., Seri, S., Liew, A., & Bagshaw, A. P. (2023). The contribution of sleep and co-occurring neurodevelopmental conditions to quality of life in children with epilepsy. *Epilepsy Research*, *194*, 107188. https://doi.org/10.1016/J.EPLEPSYRES.2023.107188

Wong, C., Gau, S. S., & Chou, T. (2019). Neural correlates of association strength and categorical relatedness in youths with autism spectrum disorder. *Autism Research*, *12*(10), 1484–1494. https://doi.org/10.1002/aur.2184

Wu, H., Lu, B., Xiang, N., Qiu, M., Da, H., Xiao, Q., Zhang, Y., & Shi, H. (2024). Different activation in dorsolateral prefrontal cortex between anxious depression and non-anxious depression during an autobiographical memory task: A fNIRS study. *Journal of Affective Disorders*, *362*, 585–594. https://doi.org/10.1016/j.jad.2024.07.031

Wu, H., Wang, J., Teng, T., Yin, B., He, Y., Jiang, Y., Liu, X., Yu, Y., Li, X., & Zhou, X. (2023). Biomarkers of intestinal permeability and blood-brain barrier permeability in adolescents with major depressive disorder. *Journal of Affective Disorders*, *323*, 659–666. https://doi.org/10.1016/J.JAD.2022.11.058

Wu, J., Qi, S., Yu, W., Gao, Y., & Ma, J. (2023). Regional Homogeneity of the Left Posterior Cingulate Gyrus May Be a Potential Imaging Biomarker of Manic Episodes in First-Episode, Drug-Naive Bipolar Disorder. *Neuropsychiatric Disease and Treatment*, *Volume 19*, 2775–2785. https://doi.org/10.2147/NDT.S441021

Wu, S., Xue, T., Li, Y., Chen, W., & Ren, Y. (2025). Comprehensive bioinformatics analysis identifies hub genes associated with immune cell infiltration in early-onset schizophrenia. *BMC Psychiatry*, *25*(1), 55. https://doi.org/10.1186/s12888-025-06499-8

Wu, S.-C. J., Hsu, J.-W., Huang, K.-L., Bai, Y.-M., Tu, P.-C., & Chen, M.-H. (2023). Functional dysconnectivity of cerebellum and attention networks in emotional dysregulation shared between attention deficit hyperactivity disorder and major depressive disorder: a multimodal imaging study. *CNS Spectrums*, *28*(4), 470–477. https://doi.org/10.1017/S1092852922000876

Wu, W., Huang, Y., Liu, X., Li, Z., Zheng, W., Wang, W., Kang, C., & Li, Y. (2026). Age-Specific Auditory Event-Related Potential Abnormalities in Children with Attention Deficit Hyperactivity Disorder: A Cross-Sectional Study. *Clinical EEG and Neuroscience*, *57*(2), 115–129. https://doi.org/10.1177/15500594251382654

Wu, X., Niu, Z., Zhu, Y., Shi, Y., Qiu, H., Gu, W., Liu, H., Zhao, J., Yang, L., Wang, Y., Liu, T., Xia, Y., Yang, Y., Chen, J., & Fang, Y. (2022). Peripheral biomarkers to predict the diagnosis of bipolar disorder from major depressive disorder in adolescents. *European Archives of Psychiatry and Clinical Neuroscience*, *272*(5), 817–826. https://doi.org/10.1007/s00406-021-01321-4

Wu, Y.-H., Gau, S. S.-F., Lo, Y.-C., & Tseng, W.-Y. I. (2014). White matter tract integrity of frontostriatal circuit in attention deficit hyperactivity disorder: Association with attention performance and symptoms. *Human Brain Mapping*, *35*(1), 199–212. https://doi.org/10.1002/hbm.22169

Wu, Z.-M., Wang, P., Liu, X.-C., Zhou, Q.-C., Cao, X.-L., Sun, L., Liu, L., Cao, Q.-J., Yang, L., Wang, Y.-F., Qian, Y., & Yang, B.-R. (2025). Functional and structural connectivity of the subregions of the amygdala in ADHD children with or without ODD. *BMC Psychiatry*, *25*(1), 74. https://doi.org/10.1186/s12888-025-06500-4

Wyller, V. B., Nguyen, C. B., Ludviksen, J. A., & Mollnes, T. E. (2017). Transforming growth factor beta (TGF-β) in adolescent chronic fatigue syndrome. *Journal of Translational Medicine*, *15*(1), 245. https://doi.org/10.1186/s12967-017-1350-1

Wyller, V. B., Vitelli, V., Sulheim, D., Fagermoen, E., Winger, A., Godang, K., & Bollerslev, J. (2016). Altered neuroendocrine control and association to clinical symptoms in adolescent chronic fatigue syndrome: a cross-sectional study. *Journal of Translational Medicine*, *14*(1), 121. https://doi.org/10.1186/s12967-016-0873-1

Xiang, J., Qin, Y., Jiang, R., Wang, X., Zhou, Y., Liu, J., & Kuang, L. (2025). Serum HMGB1 as a diagnostic biomarker and mediator of childhood trauma in adolescent depression. *Frontiers in Psychiatry*, *16*. https://doi.org/10.3389/fpsyt.2025.1584320

Xiao, Q., Shen, L., He, H., Wang, X., Fu, Y., Ding, J., Jiang, F., Zhang, J., Zhang, Z., Grecucci, A., Yi, X., & Chen, B. T. (2024). Alteration of prefrontal cortex and its associations with emotional and cognitive dysfunctions in adolescent borderline personality disorder. *European Child & Adolescent Psychiatry*, *33*(11), 3937–3949. https://doi.org/10.1007/s00787-024-02438-2

Xiao, Q., Wang, X., Yi, X., Fu, Y., Ding, J., Jiang, F., Wang, J., Han, Z., & Chen, B. T. (2023). Alteration of surface morphology and core features in adolescents with borderline personality disorder. *Journal of Affective Disorders*, *333*, 86–93. https://doi.org/10.1016/j.jad.2023.04.055

Xiao, Q., Wu, Z., Jiao, Q., Zhong, Y., Zhang, Y., & Lu, G. (2021). Children with euthymic bipolar disorder during an emotional go/nogo task: Insights into the neural circuits of cognitive-emotional regulation. *Journal of Affective Disorders*, *282*, 669–676. https://doi.org/10.1016/j.jad.2020.12.157

Xiao, Q., Yi, X., Fu, Y., Jiang, F., Zhang, Z., Huang, Q., Han, Z., & Chen, B. T. (2023). Altered brain activity and childhood trauma in Chinese adolescents with borderline personality disorder. *Journal of Affective Disorders*, *323*, 435–443. https://doi.org/10.1016/J.JAD.2022.12.003

Xiao, Q., Zhang, G., & Zhong, Y. (2024). Abnormal functional connectivity of the intrinsic networks in adolescent bipolar I versus bipolar II disorder. *Psychiatry Research: Neuroimaging*, *340*, 111802. https://doi.org/10.1016/j.pscychresns.2024.111802

Xiao, X., Hou, X., Zhang, Z., Li, Y., Yu, X., Wang, Y., Tian, J., & Xu, K. (2020). Efficacy and brain mechanism of transcutaneous auricular vagus nerve stimulation for adolescents with mild to moderate depression: Study protocol for a randomized controlled trial. *Pediatric Investigation*, *4*(2), 109–117. https://doi.org/10.1002/ped4.12198

Xie, Y., Wu, S., Li, J., Zhang, C., Zhang, Y., Hang, Y., Lang, N., Lv, Z., Zhang, P., Liang, M., Yu, B., Long, J., Liu, Y., Wang, S., Ouyang, L., Zhang, L., Wu, Y., & Wang, C. (2025). Impulse control deficits among patients with nonsuicidal self-injury: a mediation analysis based on structural imaging. *Journal of Psychiatry and Neuroscience*, *50*(2), E73–E84. https://doi.org/10.1503/jpn.240129

Xiong, Y., Chen, R.-S., Wang, X.-Y., Li, X., Dai, L.-Q., & Yu, R.-Q. (2022). Cerebral blood flow in adolescents with drug-naive, first-episode major depressive disorder: An arterial spin labeling study based on voxel-level whole-brain analysis. *Frontiers in Neuroscience*, *16*. https://doi.org/10.3389/fnins.2022.966087

Xu, H., Li, J., Xu, J., & Li, D. (2025). Machine learning-derived multimodal Neurobiological profiles of behavioral activation traits in adolescents. *European Child & Adolescent Psychiatry*, *34*(10), 3059–3070. https://doi.org/10.1007/s00787-025-02714-9

Xu, H., Lu, L., Jones, C. R., Patino, L. R., Li, X., Blom, T. J., Zhang, L., Gong, Q., Singh, M. K., & DelBello, M. P. (2025). Aberrant White Matter Microstructure in Youth at High Risk for Bipolar Disorder. *Biological Psychiatry: Cognitive Neuroscience and Neuroimaging*. https://doi.org/10.1016/j.bpsc.2025.08.017

Xu, L., Hong, X., Tang, Y., Cui, H., Wei, Y., Qian, Z., Su, W., Tang, X., Hu, Y., Zhang, D., Zheng, W., Wang, Y., Hu, H., Zhu, J., Zhang, T., & Wang, J. (2024). Direct and indirect effects of error monitoring on social functioning in a cohort with high-risk and first-episode psychosis. *Progress in Neuro-Psychopharmacology and Biological Psychiatry*, *129*, 110904. https://doi.org/10.1016/j.pnpbp.2023.110904

Xu, L., Xue, R., Ai, Z., Huang, Y., Liu, X., Wang, L., Liang, D., & Wang, Z. (2024). Resting-State Functional Magnetic Resonance Imaging as an Indicator of Neuropsychological Changes in Type 1 Narcolepsy. *Academic Radiology*, *31*(1), 69–81. https://doi.org/10.1016/j.acra.2023.08.026

Xu, M., Li, X., Teng, T., Huang, Y., Liu, M., Long, Y., Lv, F., Zhi, D., Li, X., Feng, A., Yu, S., Calhoun, V., Zhou, X., & Sui, J. (2024). Reconfiguration of Structural and Functional Connectivity Coupling in Patient Subgroups With Adolescent Depression. *JAMA Network Open*, *7*(3), e241933. https://doi.org/10.1001/jamanetworkopen.2024.1933

Xue, A. M., Foerde, K., Walsh, B. T., Steinglass, J. E., Shohamy, D., & Bakkour, A. (2022). Neural Representations of Food-Related Attributes in the Human Orbitofrontal Cortex during Choice Deliberation in Anorexia Nervosa. *The Journal of Neuroscience*, *42*(1), 109–120. https://doi.org/10.1523/JNEUROSCI.0958-21.2021

Yamada, Y., Mishima, K., Ohnishi, T., Suzuki, M., Nemoto, T., Mizuno, M., Kishimoto, T., Tomita, H., Ozone, M., Kitamura, S., Hashimoto, K., Nakagome, K., & Sumiyoshi, T. (2025). Identification of Factors to Predict Transition to Schizophrenia in Subjects with Ultra-high Risk for Psychosis: A Protocol for a Multicenter, Longitudinal Study of Sleep Parameters and Cytokine Levels. *Clinical Psychopharmacology and Neuroscience*, *23*(2), 266–277. https://doi.org/10.9758/cpn.24.1239

Yan, H., Siegel, L., Breitbart, S., Gorodetsky, C., Fasano, A., Rahim, A., Loh, A., Kulkarni, A. v., & Ibrahim, G. M. (2022). An open-label prospective pilot trial of nucleus accumbens deep brain stimulation for children with autism spectrum disorder and severe, refractory self-injurious behavior: study protocol. *Pilot and Feasibility Studies*, *8*(1), 24. https://doi.org/10.1186/s40814-022-00988-3

Yan, J., Han, V. X., Jones, H. F., Couttas, T. A., Jieu, B., Leweke, F. M., Lee, J., Loi, C., Webster, R., Kothur, K., Menezes, M. P., Antony, J., Kandula, T., Cardamone, M., Patel, S., Bandodkar, S., & Dale, R. C. (2025). Cerebrospinal fluid metabolomics in autistic regression reveals dysregulation of sphingolipids and decreased β-hydroxybutyrate. *eBioMedicine*, *114*, 105664. https://doi.org/10.1016/j.ebiom.2025.105664

Yang, B., Wang, M., Zhou, W., Wang, X., Chen, S., Potenza, M. N., Yuan, L., & Dong, G.-H. (2023). Disrupted network integration and segregation involving the default mode network in autism spectrum disorder. *Journal of Affective Disorders*, *323*, 309–319. https://doi.org/10.1016/j.jad.2022.11.083

Yang, F., Zhang, J., Fan, L., Liao, M., Wang, Y., Chen, C., Zhai, T., Zhang, Y., Li, L., Su, L., & Dai, Z. (2020). White matter structural network disturbances in first-episode, drug-naïve adolescents with generalized anxiety disorder. *Journal of Psychiatric Research*, *130*, 394–404. https://doi.org/10.1016/j.jpsychires.2020.08.004

Yang, G., Ullah, H. M. A., Parker, E., Gorsi, B., Libowitz, M., Maguire, C., King, J. B., Coon, H., Lopez-Larson, M., Anderson, J. S., Yandell, M., & Shcheglovitov, A. (2023). Neurite outgrowth deficits caused by rare PLXNB1 mutation in pediatric bipolar disorder. *Molecular Psychiatry*, *28*(6), 2525–2539. https://doi.org/10.1038/s41380-023-02035-w

Yang, H., Chen, Y., Tao, Q., Shi, W., Tian, Y., Wei, Y., Li, S., Zhang, Y., Han, S., & Cheng, J. (2024). Integrative molecular and structural neuroimaging analyses of the interaction between depression and age of onset: A multimodal magnetic resonance imaging study. *Progress in Neuro-Psychopharmacology and Biological Psychiatry*, *134*, 111052. https://doi.org/10.1016/j.pnpbp.2024.111052

Yang, J., Ma, S., Cheng, A., Zhang, L., Li, B., Cui, W., Li, K., Zhang, Y., & Liu, X. (2024). Analysis of functional network asymmetry in major depressive disorder under four fNIRS tasks. *Journal of Affective Disorders*, *365*, 303–312. https://doi.org/10.1016/j.jad.2024.08.022

Yang, Q., Huang, P., Li, C., Fang, P., Zhao, N., Nan, J., Wang, B., Gao, W., & Cui, L.-B. (2018). Mapping alterations of gray matter volume and white matter integrity in children with autism spectrum disorder. *NeuroReport*, *29*(14), 1188–1192. https://doi.org/10.1097/WNR.0000000000001094

Yang, T. T., Simmons, A. N., Matthews, S. C., Tapert, S. F., Frank, G. K., Bischoff-Grethe, A., Lansing, A. E., Wu, J., Brown, G. G., & Paulus, M. P. (2009). Depressed adolescents demonstrate greater subgenual anterior cingulate activity. *NeuroReport*, *20*(4), 440–444. https://doi.org/10.1097/WNR.0b013e3283262e10

Yap, M. B. H., Whittle, S., Yücel, M., Sheeber, L., Pantelis, C., Simmons, J. G., & Allen, N. B. (2008). Interaction of Parenting Experiences and Brain Structure in the Prediction of Depressive Symptoms in Adolescents. *Archives of General Psychiatry*, *65*(12), 1377. https://doi.org/10.1001/archpsyc.65.12.1377

Yarger, H. A., Redcay, E., Herrington, J., Kerns, C. M., & Thomas, S. B. (2025). Assessing anxiety in adolescents with autism spectrum disorder (ADORA): developing a health equity protocol for non-invasive biomarkers. *BMJ Open*, *15*(9), e107684. https://doi.org/10.1136/bmjopen-2025-107684

Yates, T., Sigwebela, S., Seedat, S., Milham, M., du Plessis, S., Abramson, L., Niemiec, E., Worthman, C., Rotheram-Borus, M. J., Salum, G., Franco, A., Zuanazzi, A., Ahmed, F., Gemmell, K., Christodoulou, J., Mhlaba, N., Mqhele, N., Ngalimane, N., Sambudla, A., … Tomlinson, M. (2025). Investigative Approaches to Resilient Emotion Regulation Neurodevelopment in a South African Birth Cohort. *Biological Psychiatry Global Open Science*, *5*(3), 100457. https://doi.org/10.1016/j.bpsgos.2025.100457

Yatzkar, U., Amir, E., Tamir, S., & Armon-Omer, A. (2024). Omega-3 Fatty Acid Supplementation Improves Attention Deficit-Hyperactivity Disorder Symptoms in Children. *Current Psychiatry Research and Reviews*, *20*(1), 48–58. https://doi.org/10.2174/2666082219666230103113402

Yee, J. Y., Lee, T.-S., & Lee, J. (2018). Levels of Serum Brain-Derived Neurotropic Factor in Individuals at Ultra-High Risk for Psychosis—Findings from the Longitudinal Youth at Risk Study (LYRIKS). *International Journal of Neuropsychopharmacology*, *21*(8), 734–739. https://doi.org/10.1093/ijnp/pyy036

Yeh, C.-H., Tseng, R.-Y., Ni, H.-C., Cocchi, L., Chang, J.-C., Hsu, M.-Y., Tu, E.-N., Wu, Y.-Y., Chou, T.-L., Gau, S. S.-F., & Lin, H.-Y. (2022). White matter microstructural and morphometric alterations in autism: implications for intellectual capabilities. *Molecular Autism*, *13*(1), 21. https://doi.org/10.1186/s13229-022-00499-1

Yerys, B. E., Gordon, E. M., Abrams, D. N., Satterthwaite, T. D., Weinblatt, R., Jankowski, K. F., Strang, J., Kenworthy, L., Gaillard, W. D., & Vaidya, C. J. (2015). Default mode network segregation and social deficits in autism spectrum disorder: Evidence from non-medicated children. *NeuroImage: Clinical*, *9*, 223–232. https://doi.org/10.1016/j.nicl.2015.07.018

Yeung, M. K., Lee, T. L., & Chan, A. S. (2019). Frontal lobe dysfunction underlies the differential word retrieval impairment in adolescents with high‐functioning autism. *Autism Research*, *12*(4), 600–613. https://doi.org/10.1002/aur.2082

Yi, X., Fu, Y., Ding, J., Jiang, F., Han, Z., Zhang, Y., Zhang, Z., Xiao, Q., & Chen, B. T. (2024). Altered gray matter volume and functional connectivity in adolescent borderline personality disorder with non-suicidal self-injury behavior. *European Child & Adolescent Psychiatry*, *33*(1), 193–202. https://doi.org/10.1007/s00787-023-02161-4

Yi, X., Fu, Y., Zhang, Z., Jiang, F., Xiao, Q., & Chen, B. T. (2023). Altered regional homogeneity and its association with cognitive function in adolescents with borderline personality disorder. *Journal of Psychiatry and Neuroscience*, *48*(1), E1–E10. https://doi.org/10.1503/jpn.220144

Yi, X., Wang, X., Fu, Y., Jiang, F., Zhang, Z., Wang, J., Han, Z., Xiao, Q., & Chen, B. T. (2024). Altered resting-state functional connectivity and its association with executive function in adolescents with borderline personality disorder. *European Child & Adolescent Psychiatry*, *33*(6), 1721–1730. https://doi.org/10.1007/s00787-023-02277-7

Yi, X., Wang, X., Shen, L., Fu, Y., Xiao, Q., Zhang, J., Ding, J., Jiang, F., Zhang, Z., Grecucci, A., & Chen, B. T. (2025). White matter microstructural abnormalities related to emotional dysfunction and childhood trauma characterize adolescents with borderline personality disorder. *Brain Imaging and Behavior*, *19*(6), 1213–1223. https://doi.org/10.1007/s11682-025-01046-1

Yip, S. W., Lacadie, C. M., Sinha, R., Mayes, L. C., & Potenza, M. N. (2016). Prenatal cocaine exposure, illicit-substance use and stress and craving processes during adolescence. *Drug and Alcohol Dependence*, *158*, 76–85. https://doi.org/10.1016/J.DRUGALCDEP.2015.11.012

Yoon, L., Rohrsetzer, F., Battel, L., Anés, M., Manfro, P. H., Rohde, L. A., Viduani, A., Zajkowska, Z., Mondelli, V., Kieling, C., & Swartz, J. R. (2022). Reward‐ and threat‐related neural function associated with risk and presence of depression in adolescents: a study using a composite risk score in Brazil. *Journal of Child Psychology and Psychiatry*, *63*(5), 579–590. https://doi.org/10.1111/jcpp.13496

Yoon, S.-H., Kim, J. S., Lee, H.-A., Lee, W.-S., Song, Y. W., & Shim, S.-H. (2025). Electrophysiological characteristics in adolescents with non-suicidal self-injury: an event-related potential study and source analysis. *Frontiers in Psychiatry*, *16*. https://doi.org/10.3389/fpsyt.2025.1596035

Yu, R.-Q., Zhang, Z.-J., Chen, R., Wang, X.-Y., Li, X., Dai, L.-Q., & Li, Z.-L. (2023). Electroconvulsive therapy-induced neuroimaging alterations measured by cerebral blood flow in adolescents with major depressive disorder. *Journal of Affective Disorders*, *327*, 385–390. https://doi.org/10.1016/j.jad.2023.02.027

Yu, T., Jia, T., Zhu, L., Desrivières, S., Macare, C., Bi, Y., Bokde, A. L. W., Quinlan, E. B., Heinz, A., Ittermann, B., Liu, C. X., Ji, L., Banaschewski, T., Ren, D., Du, L., Hou, B., Flor, H., Frouin, V., Garavan, H., … Stedman, A. (2020). Cannabis-Associated Psychotic-like Experiences Are Mediated by Developmental Changes in the Parahippocampal Gyrus. *Journal of the American Academy of Child & Adolescent Psychiatry*, *59*(5), 642–649. https://doi.org/10.1016/J.JAAC.2019.05.034

Yu, X., Xiang, J., Epstein, J. N., Tamm, L., Foster, J. A., & Becker, S. P. (2025). Neural Correlates of Cognitive Disengagement Syndrome Symptoms in Children: A Magnetoencephalography Study. *Brain Sciences*, *15*(6), 624. https://doi.org/10.3390/brainsci15060624

Yuan, H., Xu, B., Wang, Y., Ou, Y., Qiu, Y., Teng, Z., Xu, X., Wu, G., Wang, B., Mao, W., Chen, J., Wu, H., & Li, S. (2025). Identifying bipolar disorder and predicting its therapeutic response of cognitive impairment. *Journal of Affective Disorders*, *391*, 119991. https://doi.org/10.1016/j.jad.2025.119991

Zafarullah, M., Angkustsiri, K., Quach, A., Yeo, S., Durbin-Johnson, B. P., Bowling, H., & Tassone, F. (2024). Untargeted metabolomic, and proteomic analysis identifies metabolic biomarkers and pathway alterations in individuals with 22q11.2 deletion syndrome. *Metabolomics*, *20*(2), 31. https://doi.org/10.1007/s11306-024-02088-0

Zarr, R., Han, B., Estrada, E., & Cohen, D. A. (2022). The Park Rx trial to increase physical activity among low-income youth. *Contemporary Clinical Trials*, *122*, 106930. https://doi.org/10.1016/J.CCT.2022.106930

Zeni-Graiff, M., Rios, A. C., Maurya, P. K., Rizzo, L. B., Sethi, S., Yamagata, A. S., Mansur, R. B., Pan, P. M., Asevedo, E., Cunha, G. R., Zugman, A., Bressan, R. A., Gadelha, A., & Brietzke, E. (2019). Peripheral levels of superoxide dismutase and glutathione peroxidase in youths in ultra-high risk for psychosis: a pilot study. *CNS Spectrums*, *24*(03), 333–337. https://doi.org/10.1017/S1092852917000803

Zhai, S., Qu, Y., Zhang, D., Li, T., Xie, Y., Wu, X., Zou, L., Yang, Y., Tao, F., & Tao, S. (2023). Depressive symptoms predict longitudinal changes of chronic inflammation at the transition to adulthood. *Frontiers in Immunology*, *13*. https://doi.org/10.3389/fimmu.2022.1036739

Zhang, D.-W., Li, H., Wu, Z., Zhao, Q., Song, Y., Liu, L., Qian, Q., Wang, Y., Roodenrys, S., Johnstone, S. J., de Blasio, F. M., & Sun, L. (2019). Electroencephalogram Theta/Beta Ratio and Spectral Power Correlates of Executive Functions in Children and Adolescents With AD/HD. *Journal of Attention Disorders*, *23*(7), 721–732. https://doi.org/10.1177/1087054717718263

Zhang, F., Liu, L., Peng, J., Ding, G., Li, Y., Biswal, B. B., & Wang, P. (2025). Transdiagnostic and Diagnosis-Specific Morphological Similarity Related Transcriptional Profile in Attention-Deficit/Hyperactivity Disorder and Autism Spectrum Disorder. *Journal of the American Academy of Child & Adolescent Psychiatry*, *64*(9), 1068–1079. https://doi.org/10.1016/j.jaac.2024.11.015

Zhang, K., Yuan, J., Pei, X., Fu, Z., Zhao, Y., Hu, N., Wang, Y., Yang, L., & Cao, Q. (2023). Cerebral blood flow characteristics of drug-naïve attention-deficit/hyperactivity disorder with social impairment: Evidence for region–symptom specificity. *Frontiers in Neuroscience*, *17*. https://doi.org/10.3389/fnins.2023.1149703

Zhang, L., Geier, C., House, E., & Oshri, A. (2025). Latent Default Mode Network Connectivity Patterns: Associations With Sleep Health and Adolescent Psychopathology. *Brain and Behavior*, *15*(5). https://doi.org/10.1002/brb3.70579

Zhang, M., Palaniyappan, L., Deng, M., Zhang, W., Pan, Y., Fan, Z., Tan, W., Wu, G., Liu, Z., & Pu, W. (2021). Abnormal Thalamocortical Circuit in Adolescents With Early-Onset Schizophrenia. *Journal of the American Academy of Child & Adolescent Psychiatry*, *60*(4), 479–489. https://doi.org/10.1016/J.JAAC.2020.07.903

Zhang, Q., Hong, S., Cao, J., Zhou, Y., Xu, X., Ai, M., & Kuang, L. (2021). Hippocampal Subfield Volumes in Major Depressive Disorder Adolescents with a History of Suicide Attempt. *BioMed Research International*, *2021*(1). https://doi.org/10.1155/2021/5524846

Zhang, S., Tang, H., Gong, C., Liu, J., & Chen, J. (2017). Assessment of Serum Cx3Cl1/Fractalkine Level in Han Chinese Girls with Anorexia Nervosa and its Correlation with Nutritional Status: A Preliminary Cross-Sectional Study. *Journal of Investigative Medicine*, *65*(2), 333–337. https://doi.org/10.1136/jim-2016-000209

Zhang, W., Zhang, C., Zhao, J., Cui, J., Bai, J., Deng, X., Ji, J., Li, T., Wang, Y., Li, K., Qu, Y., & Li, J. (2025). Microstructure Abnormalities of Diffusion Tensor Imaging Measures in First‐Episode, Treatment‐Naïve Adolescents With Major Depressive Disorder: An Integrated AFQ and TBSS Study. *Brain and Behavior*, *15*(3). https://doi.org/10.1002/brb3.70416

Zhang, X., Gao, W., Cao, W., Kuang, L., Niu, J., Guo, Y., Cui, D., Jiao, Q., Qiu, J., Su, L., & Lu, G. (2022). Pallidal volume reduction and prefrontal-striatal-thalamic functional connectivity disruption in pediatric bipolar disorders. *Journal of Affective Disorders*, *301*, 281–288. https://doi.org/10.1016/j.jad.2022.01.049

Zhang, X., Zhou, Y., Sun, J., Yang, R., Chen, J., Cheng, X., Li, Z., Chen, X., Yang, C., Zhu, X., & Cao, L. (2022). A cohort study of adolescents with depression in China: tracking multidimensional outcomes and early biomarkers for intervention. *General Psychiatry*, *35*(4). https://doi.org/10.1136/gpsych-2022-100782

Zhang, Y., Yang, T., Cui, X., Huang, C., Xie, G., Luo, X., & He, Y. (2025). EEG microstates as indicators and predictors of response through 4 weeks of treatment in adolescents with major depressive disorder. *Journal of Affective Disorders*, *391*, 119511. https://doi.org/10.1016/j.jad.2025.119511

Zhang, Z. Z., Moeckel, C., Mustafa, M., Pham, H., Olson, A. E., Mehta, D., Dorn, L. D., Engeland, C. G., & Shenk, C. E. (2023). The association of epigenetic age acceleration and depressive and anxiety symptom severity among children recently exposed to substantiated maltreatment. *Journal of Psychiatric Research*, *165*, 7–13. https://doi.org/10.1016/j.jpsychires.2023.07.007

Zhang-James, Y., Helminen, E. C., Liu, J., Busatto, G. F., Calvo, A., Cercignani, M., Chaim-Avancini, T. M., Gabel, M. C., Harrison, N. A., Lazaro, L., Lera-Miguel, S., Louza, M. R., Nicolau, R., Rosa, P. G. P., Schulte-Rutte, M., Zanetti, M. v., Ambrosino, S., Asherson, P., Banaschewski, T., … Faraone, S. v. (2021). Evidence for similar structural brain anomalies in youth and adult attention-deficit/hyperactivity disorder: a machine learning analysis. *Translational Psychiatry*, *11*(1), 82. https://doi.org/10.1038/s41398-021-01201-4

Zhao, L., Hong, S., Peng, X., He, X., Hu, J., Ma, L., Liu, X., Tao, W., Chen, R., Jiang, Z., Zhang, C., Liao, J., Xiang, J., Zeng, Q., Dai, L., Zhang, Q., Wang, W., & Kuang, L. (2025). Impaired response inhibition to negative emotional stimuli in depressed adolescents with non-suicidal self-injury: a neurophysiological perspective. *Frontiers in Psychiatry*, *16*. https://doi.org/10.3389/fpsyt.2025.1559068

Zhao, L., Zhou, D., He, X., Peng, X., Hu, J., Ma, L., Liu, X., Tao, W., Chen, R., Jiang, Z., Zhang, C., Liao, J., Xiang, J., Zeng, Q., Dai, L., Zhang, Q., Hong, S., Wang, W., & Kuang, L. (2024). Changes in P300 amplitude to negative emotional stimuli correlate with treatment responsiveness to sertraline in adolescents with depression. *Brain Research*, *1845*, 149272. https://doi.org/10.1016/j.brainres.2024.149272

Zhao, L., Zhou, D., Hu, J., He, X., Peng, X., Ma, L., Liu, X., Tao, W., Chen, R., Jiang, Z., Zhang, C., Liao, J., Xiang, J., Zeng, Q., Dai, L., Zhang, Q., Hong, S., Wang, W., & Kuang, L. (2023). Changes in microstates of first-episode untreated nonsuicidal self-injury adolescents exposed to negative emotional stimuli and after receiving rTMS intervention. *Frontiers in Psychiatry*, *14*. https://doi.org/10.3389/fpsyt.2023.1151114

Zhao, T., Guo, H., Yang, J., Cai, A., Liu, J., Zheng, J., Xiao, Y., Zhao, P., Li, Y., Luo, X., Zhang, X., Zhu, R., Wang, J., & Wang, F. (2024). Repetitive Transcranial Magnetic Stimulation Reversing Abnormal Brain Function in Mood Disorders with Early Life Stress: from preclinical models to clinical applications. *Asian Journal of Psychiatry*, *97*, 104092. https://doi.org/10.1016/j.ajp.2024.104092

Zhao, T., Zhang, Y., Li, Y., Wu, J., Wang, R., Lv, Q., Li, D., & Lang, Y. (2024). Resting-state brain networks alterations in adolescents with Internet Gaming Disorder associate with cognitive control impairments. *Frontiers in Psychiatry*, *15*. https://doi.org/10.3389/fpsyt.2024.1404050

Zhao, X., Zhu, S., Cao, Y., Cheng, P., Lin, Y., Zhai, T., Yan, W., Li, Y., Jiang, W., Hong, W., & Du, Y. (2025). Investigating Resting-State Brain Activity in Adolescents with High-Functioning Autism Spectrum Disorder: Linking Fractional Amplitude of Low-Frequency Fluctuation to Clinical Symptoms. *Journal of Autism and Developmental Disorders*. https://doi.org/10.1007/s10803-025-06867-z

Zhao, Z., Xu, G., Sun, B., Li, X., Shen, Z., Li, S., Xu, Y., Huang, M., & Xu, D. (2019). Altered coupling of spontaneous brain activities and brain temperature in patients with adolescent-onset, first-episode, drug-naïve schizophrenia. *Neuroradiology*, *61*(5), 575–584. https://doi.org/10.1007/s00234-019-02181-5

Zheng, Q., Liu, J., Ji, Y., Zhang, Y., Chen, X., & Liu, B. (2022). Elevated levels of monocyte-lymphocyte ratio and platelet-lymphocyte ratio in adolescents with non-suicidal self-injury. *BMC Psychiatry*, *22*(1), 618. https://doi.org/10.1186/s12888-022-04260-z

Zhong, H., Zhang, J., Mo, D., Zheng, H., Li, M., Liu, W., Shen, X., Cao, X., & Jia, Y. (2024). Functional connectivity density of different suicidal behaviors in adolescents with major depressive disorders. *Frontiers in Psychiatry*, *15*. https://doi.org/10.3389/fpsyt.2024.1491042

Zhou, D., Hua, T., Tang, H., Yang, R., Huang, L., Gong, Y., Zhang, L., & Tang, G. (2024). Gender and age related brain structural and functional alterations in children with autism spectrum disorder. *Cerebral Cortex*, *34*(7). https://doi.org/10.1093/cercor/bhae283

Zhou, H., Zhou, L., Zheng, T., Ma, L., Fan, M., Liu, L., Zhao, X., & Yan, C. (2025). Unraveling the link between childhood maltreatment and depression: Insights from the role of ventral striatum and middle cingulate cortex in hedonic experience and emotion regulation. *Development and Psychopathology*, *37*(1), 292–302. https://doi.org/10.1017/S0954579423001591

Zhou, J., Yao, N., Fairchild, G., Cao, X., Zhang, Y., Xiang, Y.-T., Zhang, L., & Wang, X. (2016). Disrupted default mode network connectivity in male adolescents with conduct disorder. *Brain Imaging and Behavior*, *10*(4), 995–1003. https://doi.org/10.1007/s11682-015-9465-6

Zhou, X., Fernando, S. M., Pan, A. Y., Laposa, R., Cullen, K. R., Klimes-Dougan, B., & Andreazza, A. C. (2021). Characterizing the NLRP3 Inflammasome in Mood Disorders: Overview, Technical Development, and Measures of Peripheral Activation in Adolescent Patients. *International Journal of Molecular Sciences*, *22*(22), 12513. https://doi.org/10.3390/ijms222212513

Zhou, Y., Zhou, D., Zhang, Z., Jia, H., Chen, F., Ran, L., Chen, X., Wan, L., Wang, Y., & Wang, W. (2025). Study of pain perception under negative emotions and its large-scale brain network dynamics in adolescents with non-suicidal self-injury. *Frontiers in Psychiatry*, *16*. https://doi.org/10.3389/fpsyt.2025.1582971

Zhu, Z., Lei, D., Qin, K., Li, X., Li, W., Tallman, M. J., Patino, L. R., Fleck, D. E., Aghera, V., Gong, Q., Sweeney, J. A., McNamara, R. K., & DelBello, M. P. (2023). Brain network structural connectome abnormalities among youth with attention-deficit/hyperactivity disorder at varying risk for bipolar I disorder: a cross-sectional graph-based magnetic resonance imaging study. *Journal of Psychiatry and Neuroscience*, *48*(4), E315–E324. https://doi.org/10.1503/jpn.220209

Zilcha-Mano, S., Orbach, M., Malka, M., & Lebowitz, E. R. (2025). Oxytocin as a Biomarker of Differential Effects to SPACE vs. CBT Treatment of Child Anxiety Disorders. *Journal of Clinical Child & Adolescent Psychology*, *54*(2), 220–228. https://doi.org/10.1080/15374416.2023.2188557
